# White matter disturbances in major depressive disorder: a coordinated analysis across 20 international cohorts in the ENIGMA MDD working group

**DOI:** 10.1038/s41380-019-0477-2

**Published:** 2019-08-30

**Authors:** Laura S. van Velzen, Sinead Kelly, Dmitry Isaev, Andre Aleman, Lyubomir I. Aftanas, Jochen Bauer, Bernhard T. Baune, Ivan V. Brak, Angela Carballedo, Colm G. Connolly, Baptiste Couvy-Duchesne, Kathryn R. Cullen, Konstantin V. Danilenko, Udo Dannlowski, Verena Enneking, Elena Filimonova, Katharina Förster, Thomas Frodl, Ian H. Gotlib, Nynke A. Groenewold, Dominik Grotegerd, Mathew A. Harris, Sean N. Hatton, Emma L. Hawkins, Ian B. Hickie, Tiffany C. Ho, Andreas Jansen, Tilo Kircher, Bonnie Klimes-Dougan, Peter Kochunov, Axel Krug, Jim Lagopoulos, Renick Lee, Tristram A. Lett, Meng Li, Frank P. MacMaster, Nicholas G. Martin, Andrew M. McIntosh, Quinn McLellan, Susanne Meinert, Igor Nenadić, Evgeny Osipov, Brenda W. J. H. Penninx, Maria J. Portella, Jonathan Repple, Annerine Roos, Matthew D. Sacchet, Philipp G. Sämann, Knut Schnell, Xueyi Shen, Kang Sim, Dan J. Stein, Marie-Jose van Tol, Alexander S. Tomyshev, Leonardo Tozzi, Ilya M. Veer, Robert Vermeiren, Yolanda Vives-Gilabert, Henrik Walter, Martin Walter, Nic J. A. van der Wee, Steven J. A. van der Werff, Melinda Westlund Schreiner, Heather C. Whalley, Margaret J. Wright, Tony T. Yang, Alyssa Zhu, Dick J. Veltman, Paul M. Thompson, Neda Jahanshad, Lianne Schmaal

**Affiliations:** 1 Department of Psychiatry, Amsterdam UMC, The Netherlands; 2 Orygen, The National Centre of Excellence in Youth Mental, Health, Parkville, Australia; 3 Centre for Youth Mental Health, The University of Melbourne, Melbourne, Australia; 4 Department of Psychiatry, Beth Israel Deaconess Medical Center, Harvard Medical School, Boston, MA, USA; 5 Psychiatry Neuroimaging Laboratory, Brigham and Women’s Hospital, Harvard Medical School, Boston, MA, USA; 6 Imaging Genetics Center, Mark and Mary Stevens Neuroimaging and Informatics Institute, Keck School of Medicine, University of Southern California, Marina del Rey, CA, USA; 7 Cognitive Neuroscience Center, University Medical Center, Groningen, University of Groningen, Groningen, The Netherlands; 8 FSSBI “Scientific Research Institute of Physiology & Basic Medicine”, Laboratory of Affective, Cognitive & Translational Neuroscience, Novosibirsk, Russia; 9 Department of Neuroscience, Novosibirsk State University, Novosibirsk, Russia; 10 University of Münster, Institute of Clinical Radiology, Münster, Germany; 11 Department of Psychiatry, University of Münster, Münster, Germany; 12 Department of Psychiatry, The University of Melbourne, Melbourne, VIC, Australia; 13 The Florey Institute of Neuroscience and Mental Heatlh, The University of Melbourne, Melbourne, VIC, Australia; 14 Lab. of Experimental & Translational Neuroscience, Novosibirsk, State University, Novosibirsk, Russia; 15 Department of Psychiatry and Trinity Institute of Neuroscience, Trinity College Dublin, Dublin, Ireland; 16 North Dublin Mental Health Services, Dublin, Ireland; 17 Department of Psychiatry, University of California, San Francisco, CA, USA; 18 Department of Biomedical Sciences, Florida State University, Tallahassee, FL, USA; 19 Institute for Molecular Bioscience, The University of Queensland, Brisbane, QLD, Australia; 20 Department of Psychiatry and Behavioral Sciences, The University of Minnesota, Minneapolis, MN, USA; 21 Department of Psychiatry and Psychotherapy, Otto von Guericke University, Madgeburg, Germany; 22 German Center for Neurodegenerative Disease, Magdeburg, Germany; 23 Department of Psychology, Stanford University, Stanford, CA, USA; 24 Department of Psychiatry, University of Cape Town, Cape Town, South Africa; 25 Department of Psychiatry, Interdisciplinary Center Psychopathology and Emotion regulation (ICPE), University, Medical Center Groningen, University of Groningen, Groningen, The Netherlands; 26 Division of Psychiatry, University of Edinburgh, Edinburgh, UK; 27 Youth Mental Health Team, Brain and Mind Centre, University of Sydney, Camperdown, Australia; 28 Department of Psychiatry & Behavioral Sciences, Stanford University, Stanford, CA, USA; 29 Department of Psychiatry, University of Marburg, Marburg, Germany; 30 Department of Psychology, The University of Minnesota, Minneapolis, MN, USA; 31 Maryland Psychiatric Research Center, Department of Psychiatry, University of Maryland School of Medicine, Baltimore, MD, USA; 32 Sunshine Coast Mind and Neuroscience—Thompson Institute, Birtinya, QLD, Australia; 33 Research Division, Institute of Mental Health, Singapore, Singapore; 34 Division of Mind and Brain Research, Department of Psychiatry and Psychotherapy CCM, Charité—Universitätsmedizin Berlin, corporate member of Freie Universität Berlin, Humboldt-Universität zu Berlin, and Berlin Institute of Health, Berlin, Germany; 35 Department of Neurology, University of Magdeburg, Magdeburg, Germany; 36 Psychiatry and Paediatrics, University of Calgary, Calgary, Canada; 37 Strategic Clinical Network for Addictions and Mental Health, Calgary, Canada; 38 QIMR Berghofer Medical Research Institute, Brisbane, QLD, Australia; 39 Centre for Cognitive Ageing and Cognitive Epidemiology, University of Edinburgh, Edinburgh, UK; 40 Department of Neuroscience, University of Calgary, Calgary, Canada; 41 Alberta Children’s Hospital Research Institute, Calgary, Canada; 42 Department of Psychiatry, Institute of Biomedical Research Sant Pau, Barcelona, Spain; 43 CIBERSAM, Madrid, Spain; 44 Universitat Autònoma de Barcelona, Barcelona, Spain; 45 SAMRC Unit on Risk & Resilience in Mental Disorders, Department of Psychiatry, Stellenbosch University, Cape Town, South Africa; 46 Center for Depression, Anxiety, and Stress Research, McLean Hospital, Harvard Medical School, Belmont, MA, USA; 47 Max Planck Institute of Psychiatry, Munich, Germany; 48 Department of Psychiatry, University of Heidelberg, Heidelberg, Germany; 49 West Region and Research Division, Institute of Mental Health, Singapore, Singapore; 50 Yong Loo Lin School of Medicine, National University of Singapore, Singapore, Singapore; 51 Lee Kong Chian School of Medicine, Nanyang Technological University, Singapore, Singapore; 52 SAMRC Unit on Risk & Resilience in Mental Disorders, Department of Psychiatry & Neuroscience Institute, University of Cape Town, South Africa; 53 Mental Health Research Center, Moscow, Russia; 54 Curium-LUMC Child and Adolescent Psychiatry, Leiden University Medical Center, Leiden, The Netherlands; 55 Leiden Institute for Brain and Cognition, Leiden, The Netherlands; 56 Institute of Psychology, Leiden University, Leiden, The Netherlands; 57 Instituto ITACA, Universitat Politècnica de València, València, Spain; 58 Department of Psychiatry and Psychotherapy, University of Tübingen, Tubingen, Germany; 59 Department of Psychiatry, Leiden University Medical Center, Leiden, The Netherlands; 60 Queensland Brain Institute, The University of Queensland, Brisbane, QLD, Australia; 61 Centre for Advanced Imaging, The University of Queensland, Brisbane, QLD, Australia; 62 Amsterdam Neuroscience, Amsterdam, The Netherlands

## Abstract

Alterations in white matter (WM) microstructure have been implicated in the pathophysiology of major depressive disorder (MDD). However, previous findings have been inconsistent, partially due to low statistical power and the heterogeneity of depression. In the largest multi-site study to date, we examined WM anisotropy and diffusivity in 1305 MDD patients and 1602 healthy controls (age range 12–88 years) from 20 samples worldwide, which included both adults and adolescents, within the MDD Working Group of the Enhancing Neuroimaging Genetics through Meta-Analysis (ENIGMA) consortium. Processing of diffusion tensor imaging (DTI) data and statistical analyses were harmonized across sites and effects were meta-analyzed across studies. We observed subtle, but widespread, lower fractional anisotropy (FA) in adult MDD patients compared with controls in 16 out of 25 WM tracts of interest (Cohen’s *d* between 0.12 and 0.26). The largest differences were observed in the corpus callosum and corona radiata. Widespread higher radial diffusivity (RD) was also observed (all Cohen’s *d* between 0.12 and 0.18). Findings appeared to be driven by patients with recurrent MDD and an adult age of onset of depression. White matter microstructural differences in a smaller sample of adolescent MDD patients and controls did not survive correction for multiple testing. In this coordinated and harmonized multisite DTI study, we showed subtle, but widespread differences in WM microstructure in adult MDD, which may suggest structural disconnectivity in MDD.

## Introduction

Major depressive disorder (MDD) is a debilitating and highly prevalent psychiatric disorder, characterized by depressed mood and loss of interest in daily activities [1]. Although MDD is one of the leading causes of disability worldwide [2], our understanding of the pathophysiological basis of the disorder remains incomplete. In recent years, neuroimaging analyses have helped to characterize the neuroanatomical basis of MDD; however, consistent patterns of brain alterations have been difficult to identify due to limited power in previous studies and heterogeneity in data analysis. To address this issue, the MDD working group within the Enhancing Neuro Imaging Genetics through Meta-Analysis (ENIGMA) consortium (http://enigma.usc.edu/) initiated the largest coordinated meta-analyses of brain structure in MDD to date to investigate the robustness or consistency of neuroimaging findings across many samples worldwide. Our recent studies revealed lower hippocampal volume and altered cortical structure in MDD patients [3, 4]. Lower integrity of white matter (WM) tracts connecting these cortical and subcortical regions may suggest a ‘disconnection-syndrome’ in MDD [5]. Identifying patterns of alterations in WM in MDD could lead to the discovery of pathogenic processes and thereby guide development of new treatment targets for MDD; it could also help provide ways of monitoring or predicting response to currently available treatments.

Diffusion tensor imaging (DTI) characterizes the directionality of water diffusion in the brain and allows for the in vivo study of WM microstructural properties that cannot be measured with standard anatomical MRI. Fractional anisotropy (FA) is a common measure derived from DTI ranging from 0 to 1, where higher values typically represent directionally constrained diffusion within the WM, likely due to more intact myelin, and greater uniformity and compactness of fiber bundles. MDD patients have been reported to show lower FA, on average, in numerous WM tracts, including callosal, association, and commissural fibers; yet the pattern of deficits, and the degree of disruption is highly variable across studies [6–10]. Some studies report lower FA in the uncinate fasciculus [7] and internal capsule [8, 9], yet others did not replicate these findings [6]. Inconsistent findings across studies may be due, in part, to limited statistical power related to small sample sizes, as well as differences in analytical techniques between studies. Variations in demographic (e.g. age [11]) or clinical characteristics of participants (e.g. age of onset [12, 13] and illness duration [14]) across studies may also contribute to conflicting results in the literature as increasing age, longer illness duration and early age of onset have been associated with lower FA [13–15]. Of note, adolescent MDD may show distinct patterns of WM changes compared to adult MDD, as childhood and adolescence is a peak period for WM maturation [16].

To date, four retrospective meta-analyses of DTI studies have been performed in 2011 [17], 2013 [5] and 2016 [18, 19]. These have all reported lower FA in the corpus callosum in adult MDD patients. Other meta-analytic results included lower FA in the anterior limb of the internal capsule [19], inferior longitudinal fasciculus, posterior thalamic radiation [5] and the superior longitudinal fasciculus [17], while both higher and lower FA have been reported in the fronto-occipital fasciculus [5, 17]. One major limitation of this form of literature-based meta-analysis is its dependency on published data and therefore susceptibility to publication bias. Moreover, results of the different retrospective meta-analyses highlight different WM tracts and do not confirm findings from other meta-analyses, perhaps as they include studies with different processing protocols and different statistical analyses.

In the DTI project of the ENIGMA-MDD Working Group, we aimed to address these methodological issues and increase statistical power by initiating a worldwide effort to perform the largest coordinated multi-cohort analysis on WM alterations in MDD to date. Standardized protocols for image processing, quality assurance, and statistical analyses were applied using the ENIGMA-DTI protocols for multi-site DTI harmonization [20–22], and distributed to sites around the world. Harmonized effectsize estimates calculated across sites were then meta-analyzed.

Our primary goal was to identify and rank the most robust associations between MDD diagnosis and WM microstructure in a large sample of 1305 MDD patients and 1602 healthy controls across 20 samples from North America, Europe, Asia, and Australia. In contrast to most previous studies and meta-analyses that only examined FA, we also characterized axial diffusivity (AD), which is considered to represent a measure of axonal number, caliber, and organization, radial diffusivity (RD), which may give more insight into myelination, and mean diffusivity (MD), often considered a measure of membrane density [23]. Due to the continued maturation of WM tracts throughout adolescence, we analyzed adolescent (age ≤ 21 years) and adult (age > 21 years) patients and controls separately. Furthermore, we explored the modulating effects of clinical characteristics of MDD including age of onset, recurrence of depression, antidepressant use, severity of depressive symptoms, and number of episodes.

## Materials and methods

### Study sample

The ENIGMA-MDD DTI Working Group consists of 20 cohorts from 11 different countries and includes DTI scans from 1602 healthy controls and 1305 adults and adolescents). Demographic and clinical characteristics for each sample are presented in Tables 1 and 2. Diagnostic assessment measures and exclusion criteria for every site are presented in Supplementary Table S1. All study participants provided written informed consent and the local institutional review boards and ethics committees approved each included cohort study.

### Image processing and analysis

Scanner and acquisition parameters for all sites are provided in Supplementary Table S2. Preprocessing of diffusion weighted images, including eddy current correction, echoplanar imaging (EPI)-induced distortion correction, and tensor fitting, were performed at each site. After tensor estimation, DTI images were processed using the ENIGMA-DTI protocols (see Supplemental Note 1). Protocols with image processing as well as quality control procedures are freely available as part of the ENIGMA-DTI webpage (http://enigma.ini.usc.edu/ongoing/dti-workinggroup/) and NITRC (https://www.nitrc.org/projects/enigma_dti/). Measures of FA, MD, RD, and AD were obtained for 25 regions of interest (please see Supplementary Table S3 for a list of and description of the regions of interest). In all analyses, we combined ROIs across both hemispheres by taking the mean of the left and right hemisphere regions weighted by the number of voxels; as we did not hypothesize any lateralized effect of the disorder, our primary analysis included bilaterally averaged measures to avoid potential issues of left/right flipping between sites.

### Statistical analysis

First, differences between patients and controls in FA, MD, AD, and RD were examined within adult (age > 21 years) and adolescent (age ≤ 21 years) samples separately by linear regression analysis, and Cohen’s *d* effect size estimates were calculated. This was done for all 25 regions of interest. All analyses were corrected for age and sex and linear and nonlinear age and sex interactions (age-by-sex interaction, age^2^ and age^2^-by-sex interaction). To examine whether case-control effects were regional effects beyond a global effect, these analyses were performed again while including average FA, MD, RD, or AD as an additional covariate.

We further performed diagnosis-by-sex and diagnosis-by-age interaction analyses. Case-control differences were also examined in different age categories (10–<20 years; 20–<30 years; 30–<40 years; 40–<50 years, and 50–<60 years; please see Supplemental Note 4 and Supplemental Tables). Separately within adult (age > 21) and adolescent (≤21 years) samples, we performed stratified meta-analyses to compare age of onset (adolescent onset ≤ 21 years of age, adult onset > 21 years; in adult samples only), antidepressant use at the time of scanning (antidepressant users and antidepressant non-users) and MDD stage (first and recurrent episode patients) and to investigate associations with symptom severity (Beck Depression Inventory (BDI-II) and the 17-item Hamilton Depression Rating Scale (HDRS-17)) and the number of depressive episodes. For the latter two analyses with continuous measures and interaction analyses, we extracted the beta regression coefficient as the effect size measure.

Analyses scripts are available on the ENIGMA-GitHub (https://github.com/ENIGMA-git/). All regression models and effect sizes were computed for each site separately and regression outputs were then meta-analyzed across sites.

### Meta-analysis

A random-effects inverse-variance weighted meta-analysis was conducted in R (metaphor package, version 1.9–118) to combine effect sizes from all sites. Heterogeneity scores (*I*^2^), indicating the percentage of total variance explained by heterogeneity of the effects alone, were also computed for each test, with lower values indicating lower effect size estimate variance across sites. The false discovery rate (FDR) multiple comparisons correction was applied to correct for the number of WM tracts. In addition, in the supplemental tables, we show which findings survive FDR-correction across all four diffusion measures and the 25 WM tracts (100 measures; see supplements). All reported *p*-values are FDR-corrected *p*-values.

### Secondary analyses

#### Moderator analysis

We performed moderator analyses using meta-regression to examine whether characteristics of individual sites explained variation in effect sizes across sites. Methods and results of these analyses are presented in Supplemental Note 2.

#### UK Biobank

To examine whether our findings generalize to a population study, we analyzed a large population cohort of adults (age range: 43–78 years) from the UK Biobank (1st and 2nd release) a large-scale population health study of adults in the United Kingdom. 2096 individuals were identified to have ‘probable lifetime MDD’, as previously described [24], based on hospital admissions data and self-report of depressive symptoms. DTI measures from patients were compared to DTI measures from 3275 controls without mental disorders. Image acquisition and processing of UK Biobank data is described in Supplemental Note 3. In order to examine overlap between the UK Biobank sample and the meta-analysis, the case-control analyses in the meta-analysis were also performed including only subjects within the UK Biobank age range (43–78 years).

## Results

### Adults

**Fig. 1:**
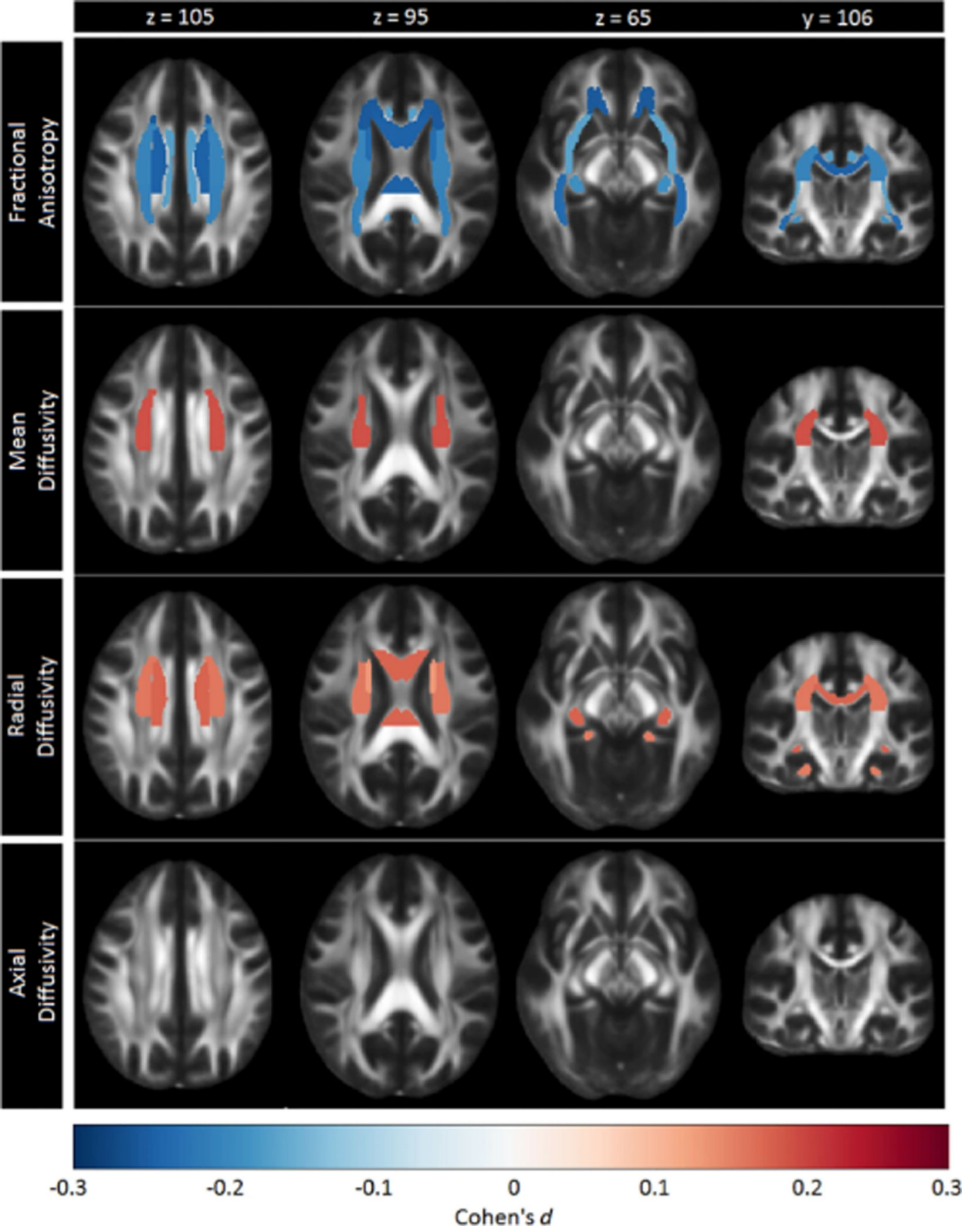
Cohen’s *d* effect sizes for case-control differences in fractional anisotropy, mean diffusivity, radial diffusivity, and axial diffusivity across adults and adolescents

**Fig. 2:**
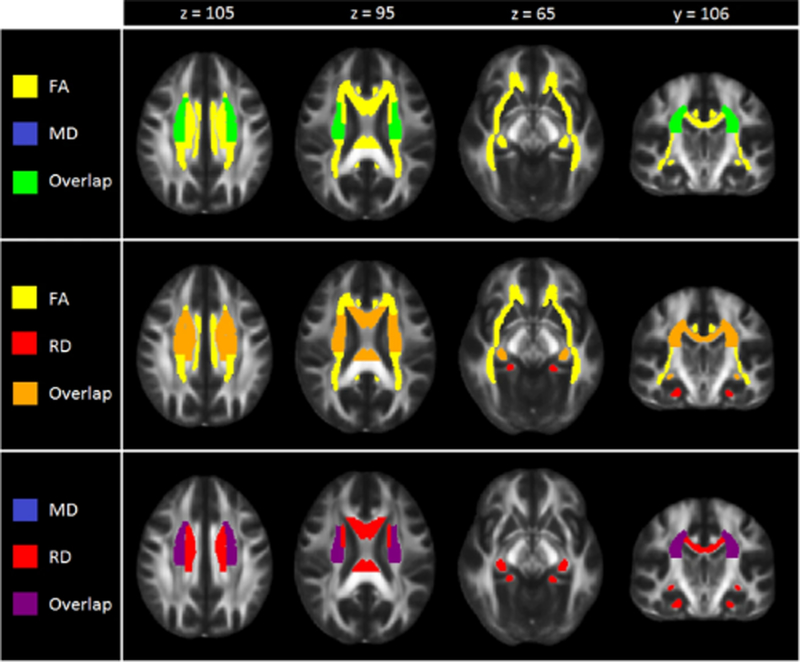
Regional overlap in case-control differences in white matter integrity across adults and adolescents

After FDR correction, significantly lower FA was observed for adult MDD patients (*N* = 921; age range 22–88) compared to healthy controls (*N* = 1265) in 16 of the 25 ROIs, with the largest effects observed for the full WM skeleton, followed by the anterior corona radiata (ACR), corona radiata (CR), corpus callosum (CC), genu of the corpus callosum (GCC), body of the corpus callosum (BCC) and anterior limb of the internal capsule (ALIC). Significantly lower FA was also observed in the superior fronto-occipital fasciculus (SFO), sagittal stratum (SS), internal capsule (IC), posterior corona radiata (PCR), superior corona radiata (SCR), inferior fronto-occipital fasciculus (IFO), fornix/stria terminalis (FXST), external capsule (EC), and cingulate gyrus of the cingulum bundle (CGC) (Fig. 1, Supplementary Table S4 and Supplementary Fig. 1). No significant effects were observed for AD or MD differences in adults (Tables S4 and S6 and Supplementary Fig. 1). Higher RD for the adult sample was observed across seven ROIs, including the FXST, BCC, SCR, hippocampal part of the cingulum bundle (CGH), the full WM skeleton, CR, and SFO (Fig. 2, Table S7 and Supplementary Fig. 1).

### Adolescents

After FDR correction, no significant differences were observed for FA, AD, MD, or RD between adolescent MDD patients (*N* = 372) and healthy controls (*N* = 290) (Tables S8–11 and Supplementary Figures. 2 and 3).

### Correction for average anisotropy/diffusivity

No significant differences between MDD patients and controls were observed after correcting for average anisotropy and diffusivity across the WM skeleton in the adult samples (Tables S12–15) and adolescent samples (Tables S16–19). These results indicate that no region showed additional effects beyond the global effect.

### Diagnosis by sex interaction

#### Adults

There were no significant diagnosis-by-sex interaction effects in adult subjects (Tables S20–23).

#### Adolescents

A diagnosis-by-sex interaction for adolescents was observed for RD in the uncinate fasciculus (UNC; Table S27), post-hoc tests showed that this was driven by higher RD in patients compared to controls in males only (*d* = 0.760, *p* = 0.011).

### Diagnosis by age interaction

#### Full group

Diagnosis-by-age interaction analyses were performed across adults and adolescents to obtain the largest age range possible. Interaction effects were observed for FA in the BCC, CC, FXST, GCC, SS, and average FA (Table S28), post-hoc tests showed that case-control differences increased with age. There were no interaction effects for AD, MD, and RD (Tables S29–31).

### First and recurrent episodes

#### Adults

Lower FA was observed for recurrent MDD patients (*N* = 645) compared to controls (*N* = 1053) across 15 ROIs, with the largest effects observed in the ACR, GCC, CR, ALIC, full WM skeleton, IC and the whole CC. Significant effects were also observed in the BCC, SFO, SS, PCR, superior longitudinal fasciculus (SLF), retrolenticular part of the internal capsule (RLIC), PTR, and IFO (Table S32). No significant effects were observed for MD or AD (Tables S33 and S34). Significantly higher RD was observed for MDD patients in the SCR (Table S35). No significant differences were observed between first-episode MDD patients (*N* = 169) and controls (*N* = 816) or between first-episode MDD patients and recurrent MDD patients (Tables S36–43).

#### Adolescents

No significant differences were observed between first episode adolescent patients (*N* = 98) and controls (*N* = 130) or between recurrent adolescents (*N* = 148), and controls (*N* = 146) (Tables S44-55).

### Age of onset

#### Adults

No significant differences were observed between adolescent age of onset MDD (≤21 years (*N* = 251) and controls (*n* = 869) (Tables S56–59). MDD patients with an adult age of onset (>21years) (*N* = 399) had significantly lower FA compared to controls (*N* = 853) across 10 ROIs, with the largest effects observed for the EC, SS, and the IFO. Significant effects were also observed in the CC, GCC, CGC, PCR, CR, SLF, and the FA across the full WM skeleton (Supplementary Table S60). No significant differences were observed between adult onset (*N* = 334) and adolescent onset MDD (*N* = 197) (Tables S64–67).

### Antidepressant use at the time of scanning

#### Adults

No differences were observed between antidepressant users (*N* = 406) and controls (*N* = 848) (Tables S68–71). Significantly lower FA was observed for antidepressant non-users (*N* = 288) compared to controls (*N* = 962) in the FXST, the entire WM skeleton, FX, CGH, SS, and EC (Table S72). No significant effects were observed for AD or MD (Tables S73 and S74). Antidepressant non-users had higher RD in the FXST compared to controls (Table S75). No differences were observed between antidepressant users (*N* = 335) and non-users (*N* = 164) (Tables S76–79).

#### Adolescents

No significant differences were observed between adolescent antidepressant users (*N* = 128) and controls (*N* = 138), antidepressant non-users (*N* = 188) and controls (*N* = 156) or between antidepressant non-users (N = 104) and antidepressant users (*N* = 112) (Tables S80–S91).

### WM associations with symptom severity

#### Adults

No significant associations were observed between anisotropy or diffusivity and symptom severity scores assessed with the BDI (*N*= 477) or HDRS (*N*= 603) (Tables S92–99).

#### Adolescents

No significant associations were observed between anisotropy or diffusivity and BDI (*N* = 168) or HDRS (*N* = 191) scores (Tables S100–107).

#### Moderator analysis

Results of the moderator analyses are presented in Supplemental Note 2.

#### UK Biobank

Significantly lower FA in the SFO was observed in patients with probable MDD (*N* = 2096) compared to healthy controls (*N* = 3275) in the UK Biobank sample (Table S108). Higher AD in patients was observed in the SCR (Table S109). Higher MD in patients was observed in the ACR, ALIC, CR, SCR, and SFO (Table S110). Finally, RD was higher in patients in the ACR, ALIC, CR, SCR, and SFO (Table S111).

## Discussion

A coordinated approach was used to perform the largest multi-site DTI study in MDD to date. Differences in FA and diffusivity measures (AD, RD, and MD) were examined between depressed patients and healthy controls, as well as associations with clinical characteristics of the disorder. We included DTI data from 20 cohorts, including a total of 1305 patients with MDD and 1602 healthy controls. Separate analyses were performed for adolescent and adult MDD, and we found evidence for globally lower FA and higher RD across the full WM skeleton in adult MDD patients. These effects were mostly driven by patients with recurrent episodes, patients with an adult (older than 21 years) age of onset of depression, and patients not taking antidepressants at the time of scanning.

In adult patients with MDD, we found evidence of subtle, but widespread lower FA in 16 out of 25 tract-based ROIs including the corpus callosum, internal capsule, inferior fronto-occipital fasciculus, corona radiata, cingulum, fornix, superior fronto-occipital fasciculus (SFO), and sagittal stratum. These effects appear to be global, as these significant associations were no longer significant after controlling for average/global FA.

The regions that most strongly contributed to the global effect of lower FA were regions of the corona radiata and corpus callosum. Lower FA in the CC is consistent with previous meta-analyses of WM in MDD [5, 18, 19]. The CC is the largest interhemispheric commissure in the human brain [25], and connects amongst others the anterior cingulate cortex and orbitofrontal cortex in both hemispheres. These regions play an important role in mood regulation [26], show cortical thinning in MDD [4] and show functional abnormalities in relation to cognitive control, working memory and emotion processing in MDD [27–29]. Changes in FA in the CR have been observed in single site studies of adult and geriatric depression [30–32]. This tract is part of the limbic-thalamo-cortical circuitry, and contains thalamic projections to cortical regions and also plays an important role in emotion regulation [33, 34].

In adults, global lower FA in some WM tracts in MDD was accompanied by global higher RD, yet no differences found in MD or AD. Previous work suggests that changes in RD reflect changes in myelination or morphology of glial cells [35, 36]. In line with this, a prior quantitative MRI study of individuals with MDD reported global reductions of R1, an MRI parameter that is thought to reflect myelin content [37]. Furthermore, previous post-mortem studies have reported lower oligodendrocyte density in the amygdala and prefrontal cortex in MDD patients [38–40] and lower expression of genes related to oligodendrocyte function [41]. Overall, we provided evidence that adult MDD is associated with subtle widespread differences in WM microstructure that are robust across many samples worldwide.

WM abnormalities in adult MDD appear to be driven by patients with more than one episode of MDD. Abnormalities in these WM tracts may be related to cumulative effects of stress on brain structure. Psychological stress is associated with increased glucocorticoid release and stress-related neuroinflammation, which have been shown to negatively impact WM microstructure [42, 43]. Our results suggest that exposure to multiple episodes of MDD may have a neuroprogressive effect on WM microstructure. We did not observe any associations between FA or diffusivity measures and the number of MDD episodes, suggesting that the relationship with the number of episodes may not be linear. It is, however, also possible that WM abnormalities in these tracts are a risk factor for an unfavorable course of MDD or that the study is underpowered to detect effects in first-episode patients.

WM differences were also primarily found in patients with an adult age of onset (>21 years). The findings may be related to age, as the mean age at the time of scanning was higher in adults with an adult-onset compared to adults with an adolescent-onset depression. We speculate that depression may interact with the normal aging process, as results of diagnosis-by-age interaction analysis showed that MDD is associated with increased decline in overall and interhemispheric FA with age. These results suggest that MDD may be associated with accelerated brain aging, which has been shown for regional gray matter volume [44, 45], although longitudinal studies are required to confirm this hypothesis. A previous study did not observe a diagnosis-by-age interaction effect on WM, but this study was performed in a smaller sample of patients and controls and may have been underpowered to detect small effects [46].

Identifying neuroimaging biomarkers related to antidepressant treatment is important, as it not only increases our understanding of the pathophysiology of MDD, but may also reveal neural mechanisms through which antidepressants improve mood. In this study we observed lower FA in the cingulum, sagittal stratum, external capsule, and fornix in patients who were not taking antidepressant medication at the time of scanning. No such differences were observed between patients who were taking antidepressants and healthy subjects, which could suggest that WM deficits may normalize with antidepressant use. In line with this, selective serotonin reuptake inhibitors (SSRIs) have previously been associated with increased myelination, plasticity and myelin repair [47]. SSRIs also increase release of brain-derived neurotrophic factor (BDNF), a neuroprotective protein that impacts oligodendrocytes and increases myelination in the central nervous system [48]. However, caution is warranted when interpreting these findings as medication effects, as we have only examined antidepressant use at the time of scanning and do not have data on past antidepressant use. Furthermore, the cross-sectional nature of this study and possible confounding effect of disease severity also limit our ability to conclude that these findings are medication effects. While we acknowledge these limitations, this study is the largest study to date to examine associations between current AD use and WM microstructure. Our findings are in line with a study by Zeng et al. [49], which is to our knowledge the only study to date that has examined alterations in WM between *medication-naive* MDD patients and MDD patients using antidepressants. Although their sample was small, the authors conclude that antidepressant use normalizes WM deficits in MDD. Ultimately, to fully elucidate the direct effect of antidepressant use on WM microstructure, DTI measures should be incorporated in randomized longitudinal clinical trials with antidepressant medication in medication-naive MDD patients.

In secondary analyses, we ran the same analyses on a large dataset from UK Biobank to examine how our findings generalize to an adult population sample. Effect sizes were generally lower and most significant findings in the meta-analysis were not significant in the UK Biobank sample. Including only subjects in the meta-analysis within the age range of UK Biobank did not increase the consistency in findings in the meta-analysis and UK Biobank (see Tables S140–143). We speculate that this different pattern of results across regions and in effect sizes between the meta-analysis and UK Biobank may be related to the fact that UK Biobank is a community cohort study (of relatively well individuals) with a lifetime diagnosis of MDD, while most studies in the meta-analysis have specifically recruited participants with a current diagnosis MDD and may have more severe symptoms at time of scanning. Finally, differences in the processing of images between the ENIGMA-DTI protocol and the UK Biobank protocol may be important, although most regions of interest show good agreement between protocols (please see supplements).

In adolescents with MDD (21 years or younger), differences in WM microstructure of the corpus callosum (i.e., lower FA and higher MD and RD) were also observed compared to age-matched healthy controls, however, these findings did not survive FDR-correction for multiple testing. The effect sizes of case-control comparisons in adolescents were comparable to adults in the corpus callosum, but were lower in other tracts. It is possible that the effects are smaller in adolescent MDD compared to adult MDD, due to the shorter disease duration and lower recurrence of MDD episodes in adolescents. As the sample size in adolescent MDD was smaller than in adult MDD, we may not have been able to detect similar small differences in WM in adolescent MDD as we observed in adult MDD. The effect sizes for differences in FA in the corpus callosum were similar to the effect sizes observed in adult MDD, suggesting that larger studies in adolescent MDD are needed to confirm whether subtle differences in FA of the corpus callosum can also be detected in adolescent MDD patients.

The reported abnormalities in WM microstructure are likely not specific to MDD, as previous work from the ENIGMA schizophrenia working group has also revealed lower global FA and higher RD in schizophrenia patients compared to healthy controls [50]. The strongest effects were observed in the same regions, namely the corona radiata and corpus callosum, although the effect sizes were higher in patients with schizophrenia (maximum Cohen’s *d* = 0.42). WM microstructure studies are currently underway in other ENIGMA disease working groups, allowing future comparisons across multiple mental disorders.

We observed significant differences in adult MDD patients in WM tracts that lead to the hippocampus, including the fornix/stria terminalis and the hippocampal portion of the cingulum bundle. Smaller hippocampal volumes (on average) is one of the most consistently reported neuroimaging findings in the depression literature (for meta-analyses please see [3, 51]). Similar mechanisms may underlie both findings. Stress-related decreases in hippocampal gliogenesis, a reduction in the number of hippocampal oligodendrocytes or stress-related morphological changes to hippocampal neurons (i.e. decreased dendritic length and number of dendritic spines), could affect both hippocampal volume and WM microstructure [52, 53]. It is also possible, however, that hippocampal volume and WM microstructure are independently impacted by stress-related mechanisms including glutamate excitotoxicity, inflammation, oxidative stress or an increase in glucocorticoids. Recent imaging genetics studies also suggest that a genetic predisposition to such neuroanatomical variations may also be related to the genetic underpinnings of mental illness [54].

### Limitations

While the sample size and use of harmonized protocols in this multi-site study are strengths, we also acknowledge certain limitations. First, we used a cutoff of 21 years for adolescent and adult MDD and for categorizing adolescent and adult age of onset. While this is consistent with our previous work [3, 4], alternative definitions may affect our findings. We also performed the case-control analysis using a cutoff of 25 years, with adolescents being 25 years or younger and adults older than 25 years. Results in adults were very similar to the findings using the cutoff at 21 and there were still no significant case-control differences in adolescents. Second, analyses of effects of age and age of onset on WM microstructure were performed at the site-level prior to meta-analysis, and are therefore hampered by the limited range of age and age of onset within each site. Future studies harmonizing individual level data across studies may allow for data to be pooled and for the effects of age and age of onset to be examined across the total age range across studies (12–88 years). Third, while several limitations of TBSS have been recognized (please see [55]), it remains the most widely used method to examine group differences in WM microstructure. Fourth, while DTI analysis was harmonized using standardized protocols, DTI acquisition was not harmonized, which may have affected our findings. Still, our moderator analyses showed no effect of DTI acquisition parameters on effect sizes. Including DTI data that was acquired with different acquisition parameters helps ensure that the findings are not specific to any specific acquisition scheme. Finally, in this study we were only able to examine antidepressant use at time of scanning, but future studies may want to examine associations between duration or (cumulative) dose of antidepressants and WM microstructure.

## Conclusion

In this first coordinated multi-site study of WM tract integrity in MDD, we provide evidence for subtle, global differences in FA and RD in multiple WM tracts in adult MDD, with the strongest regional effects being observed in tracts that have been implicated in emotion regulation. Future studies with a larger sample of adolescents are needed to examine whether more subtle WM alterations already exist at this age. Our results suggest that widespread structural dysconnectivity may play a role in the pathophysiology of MDD and may become more pronounced with recurrent episodes of MDD.

## Supplementary Material

Figures

Tables

Notes

## Figures and Tables

**Table 1:** Demographic data for all samples included in the ENIGMA-MDD-DTI project

Sample	Adult samples (age >21)						Adolescent samples (age ≤21)					
	Age controls (mean ± SD)	Age MDD (mean ± SD)	% Female controls	%Female MDD	Total no. of controls	Total MDD	Age controls (mean ± SD)	Age MDD(mean ± SD)	% Female controls	%Female MDD	Total no. controls	Total of no. of MDD
1 Barcelona	46.41 ± 7.82	47.29 ± 7.88	72.40	78.20	29	55						
2 Bipolar family study	24.70 ± 1.88	25.22 ± 2.21	53.40	55.60	73	18						
3 CODE	38.45 ± 13.29	35.41 ± 11.76	50.00	59.30	22	27						
4 DIP	44.50 ± 14.71	43.38 ± 15.75	65.00	62.50	20	16						
5 S expect	33.75 ± 7.20	39.21 ± 11.11	15.00	42.10	20	19						
6 EPISCA							14.60 ± 1.64	15.53 ± 1.55	85.00	82.40	20	17
7 MOTAR	34.65 ± 12.14	33.77 ± 8.53	57.70	61.30	26	31						
8 MPIP	52.01 ± 12.38	50.98 ± 12.86	63.80	56.90	130	137						
9 Munster neuroimaging cohort	38.37 ± 11.03	38.82 ±11.36	57.30	56.80	386	125	19.68 ± 1.67	18.92 ± 1.44	59.50	69.20	37	13
10 NESDA	54.91 ± 8.59	48.37 ± 10.05	45.50	69.50	22	57						
11 Novosibirsk	40.13 ± 8.82	46.33 ± 12.23	46.70	77.10	30	48						
12 QTIM	24.82 ± 1.89	25.15 ± 2.22	71.40	41.43	84	13	20.14 ± 1.16	19.71 ± 1.29	62.07	84.61	87	13
13 Sydney	25.86 ± 3.37	24.87 ± 3.94	54.90	55.20	71	67	19.00 ± 2.67	17.73 ± 2.10	75.90	67.70	29	155
14 Imaging Genetics Dublin	38.26 ± 12.40	41.81 ± 10.76	52.20	62.30	46	53						
15 UCSF							15.38 ± 1.35	15.74 ± 1.28	57.77	60.66	45	61
16 Child and Adolescent Imaging Research Calgary							19.40 ± 1.51	17.80 ± 1.44	80.00	55.00	10	20
17 FQR2017	34.73 ± 12.27	39.37 ± 12.92	60.90	59.60	274	218	19.54 ± 1.36	19.76 ± 1.05	73.10	64.00	26	25
18 University of Minnesota							15.67 ± 2.07	15.40 ± 1.82	66.70	76.50	36	68
19 Stanford	32.00 ± 10.13	35.00 ± 8.49	100.00	100.00	15	14						
20 IMH MDD Singapore	38.53 ± 4.64	39.30 ± 8.34	52.90	43.50	17	23						

Age (in years) and sex for patients and controls, presented separately for adult and adolescent samples

**Table 2:** Clinical data for all samples included in the ENIGMA-MDD-DTI project: percentage of MDD patients using antidepressants, percentage of acute versus remitted MDD, age of onset of MDD, percentage of MDD patients with a comorbid anxiety disorder and severity of MDD symptoms, presented separately for adult (age >21) and adolescent (age ≤21) samples

Study	Sample	% AD use	% first episode/recurrent MDD	% Acute/remitted MDD	Age of onset MDD (mean± SD)	% comorbid anxiety disorder	HDRS-17 severity MDD (mean ± SD)^a^	BDI-II severity MDD (mean±SD)^b^
*Adult samples*								
1	Barcelona	94.50	32.70/67.30	63.60/36.40	32.91 ±11.45	0.0	13.51 ±9.04	NA
2	Bipolar family study	16.70	NA	NA	22.80 ±2.70	NA	4.94 ±4.72	NA
3	CODE	0.00	0.00/100.00	100.00/0.00	NA	NA	NA	NA
4	DIP	50.00	31.25/68.75	100.00/0.00	26.00 ±13.46	31.3	NA	25.94 ±10.03
5	S expect	100.00	20.00/80.00	100.00/0.00	30.87 ±11.24	0.1	11.90 ± 5.39	20.86 ±12.40
6	MOTAR	0.00	71.00/29.00	100.00/0.00	24.52 ±9.93	45.2	NA	NA
7	MPIP	NA	0.00/100.00	70.00/30.00	32.20 ±12.76	17.9	NA	14.25 ±10.87
8	FOR2017	60.60	22.50/75.70	73.90/21.10	27.34± 13.12	12.0	8.85 ± 8.00	17.58 ± 11.16
9	Munster neuroimaging cohort	88.80	22.40/77.60	100.00/0.00	30.15 ±12.13	NA	23.09 ±5.15	28.56±9.16
10	NESDA	33.90	6.80/93.20	37.30/62.70	25.44 ±10.60	23.7	NA	NA
11	Novosibirsk	18.80	60.40/39.60	100.00/0.00	38.60± 14.16	0.0	18.41 ±4.75	31.73 ±10.87
12	QTIM	NA	NA	NA	19.54 ±4.74	30.8	NA	NA
13	Stanford	21.40	0.00/100.00	100.00/0.00	16.79 ±6.34	64.3	18.86 ±4.22	29.71 ±6.41
14	Sydney	40.30	31.30/31.30	40.30/35.80	14.11 ±7.39	26.9	12.48 ±7.26	4.00 ±3.39
15	Imaging Genetics Dublin	75.50	0.00/100.00	100.00/0.00	25.26 ±12.67	NA	23.53 ±5.00	33.17 ± 11.60
16	IMH MDD Singapore	100.00	34.80/65.20	21.70/78.30	33.39 ±9.83	NA	6.30 ±6.23	NA
*Adolescent samples*								
1	Child and Adolescent Imaging Research Calgary	65.00	0.00/100.00	100.00/0.00	14.10 ±2.29	79.3	20.40 ±6.31	30.50± 10.51
2	EPISCA	11.80	100.00/0.00	100.00/0.00	NA	29.4	NA	NA
3	University of Minnesota	23.50	23.59/32.40	NA	12.46 ±2.35	63.3	NA	26.35 ±12.06
4	FOR2017	60.00	24.00/76.00	80.00/20.00	14.21 ±2.96	17.6	11.45 ±9.17	19.13 ±12.02
5	Munster neuroimaging cohort	84.60	53.80/46.20	100.00/0.00	16.77 ±2.89	NA	22.38 ±3.86	30.08 ±10.40
6	QTIM	NA	NA	NA	17.92 ±2.10	46.2	NA	NA
7	Sydney	41.90	29.00/32.30	46.50/31.00	11.57 ± 5.52	23.2	12.65 ±7.86	2.31 ±0.95
8	UCSF	0.00	35.09/64.91	92.99/7.01	13.40 ±2.34	61.0	NA	28.33 ± 11.12

a Hamilton Depression Rating Scale (HDRS-17; range: 0–52)

b Beck Depression Inventory (BDI-II; range: 0–63)
