# Supplementary material for: White matter disturbances in major depressive disorder: a coordinated analysis across 20 international cohorts in the ENIGMA MDD working group
Source: Mol Psychiatry. 2019 Aug 30;25(7):1511–25. doi: 10.1038/s41380-019-0477-2 (PMC7055351; doi:10.1038/s41380-019-0477-2)
Supplement: Supplementary file 1 — Supplementary Figures [file 41380_2019_477_MOESM1_ESM.docx]

**Supplementary Figures**


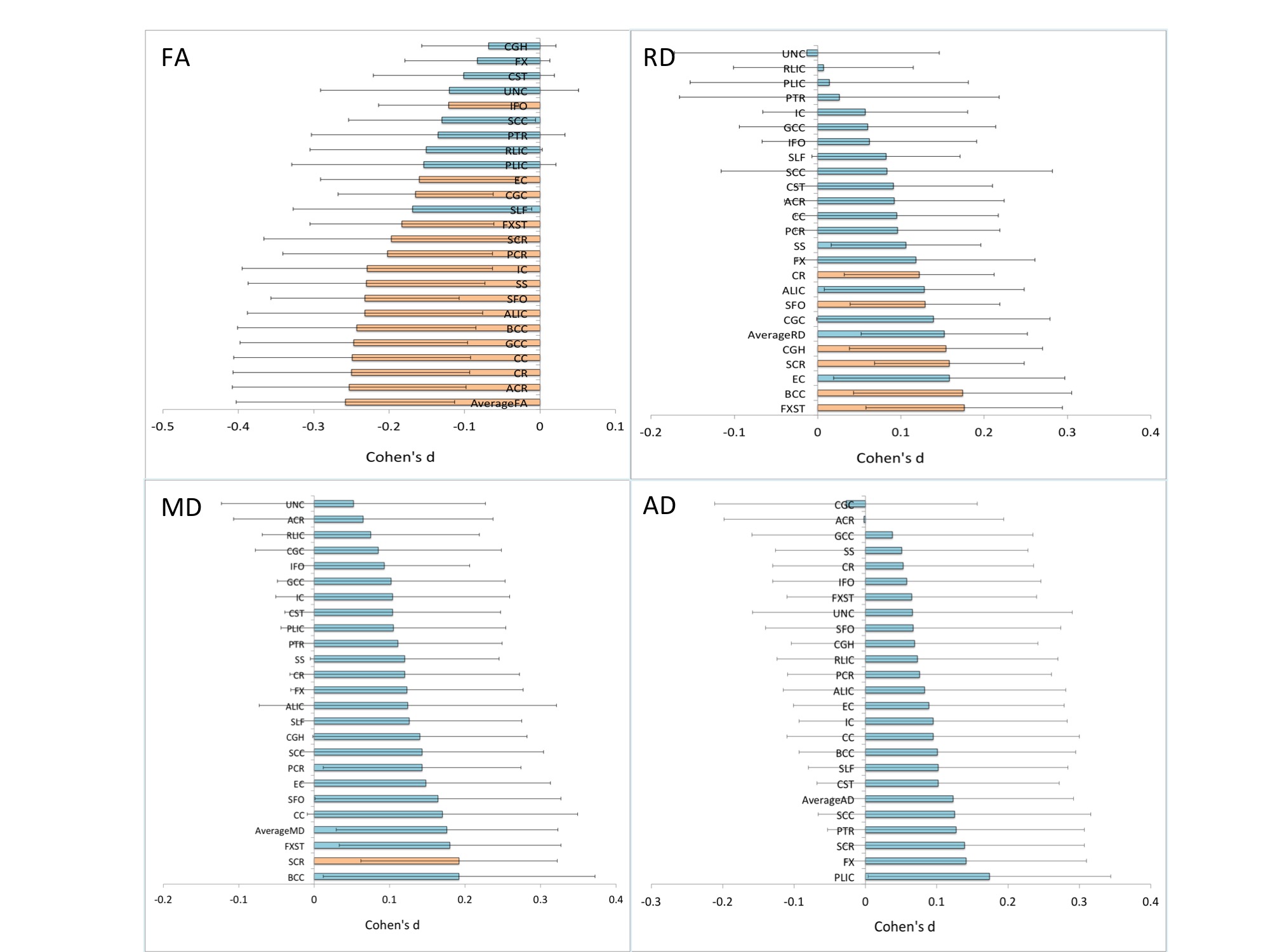


**Supplementary figure 1.** Cohen’s *d* effect sizes, after meta-analysis, for FA, AD, MD and RD differences between adult MDD patients and healthy controls, including age, sex, age×sex, age^2^ and age^2^×sex, as covariates. Error bars represent the 95% confidence interval. Significant regions after FDR correction in orange.

**
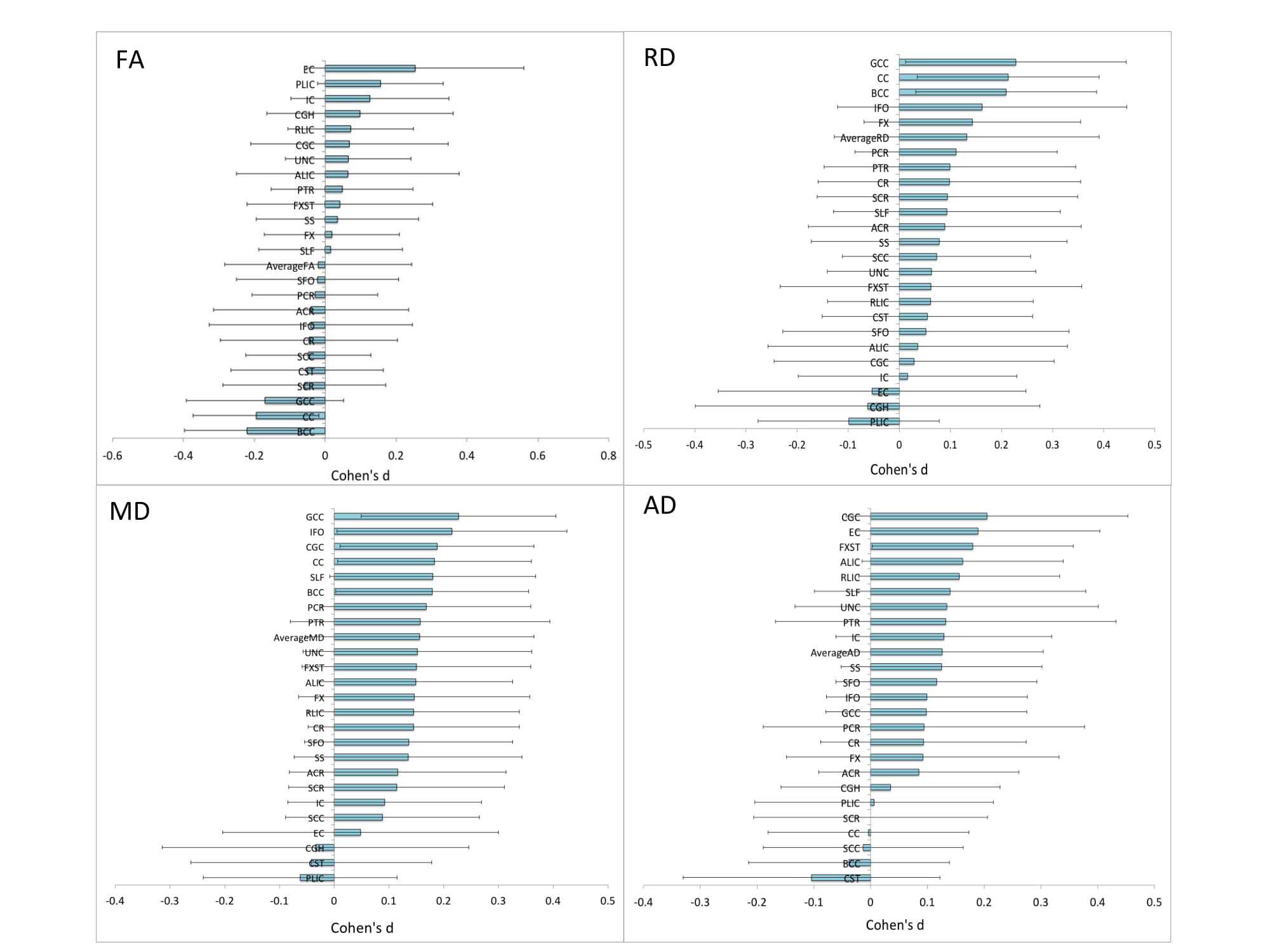
**

**Supplementary figure 2.** Cohen’s *d* effect sizes, after meta-analysis, for FA, AD, MD and RD differences between adolescent MDD patients and healthy controls, including age, sex, age×sex, age^2^ and age^2^×sex, as covariates. Error bars represent the 95% confidence interval. Significant regions after FDR correction in orange.

**
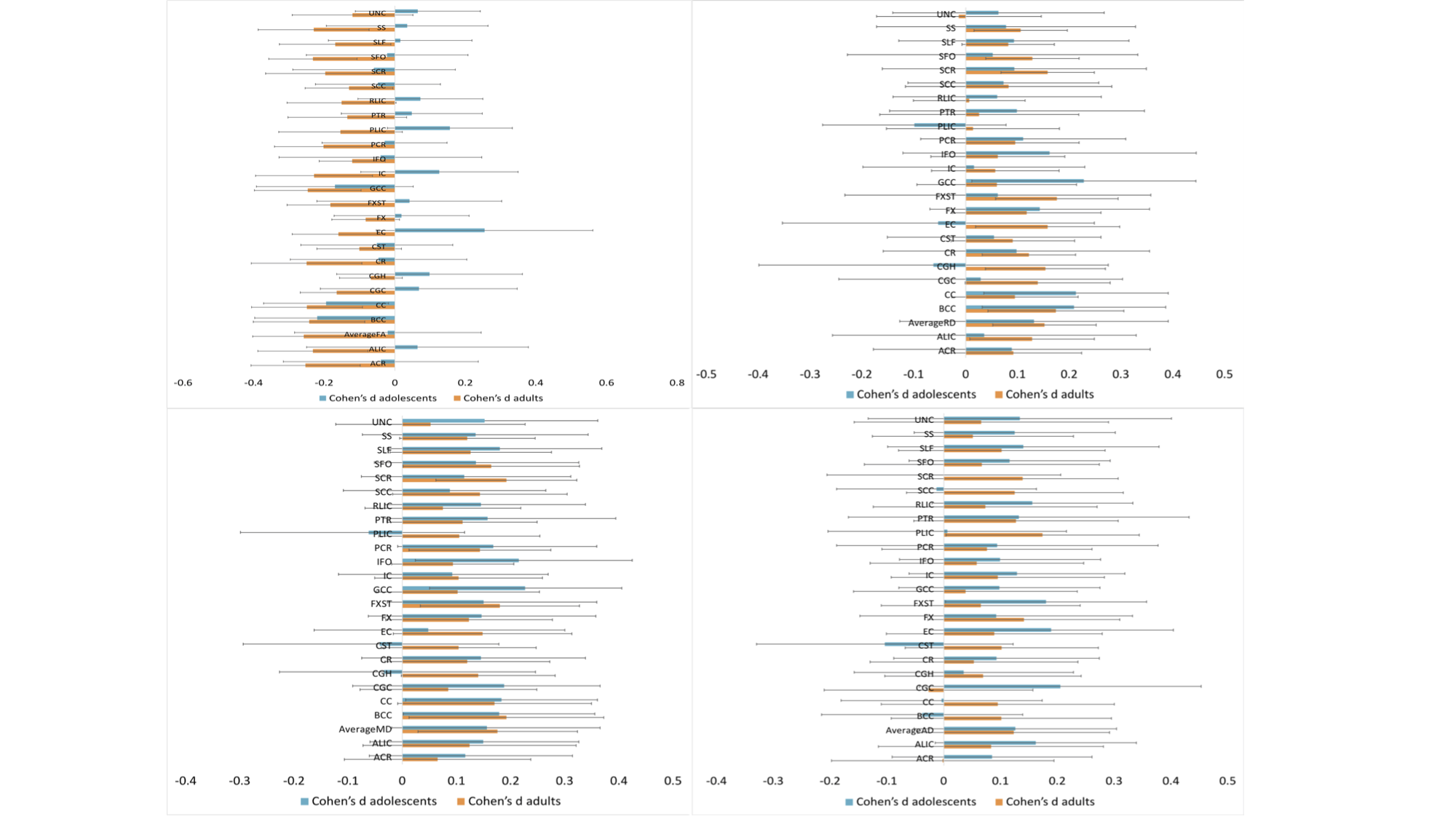
**

**Supplementary figure 3.** Cohen’s *d* effect sizes, after meta-analysis, for FA, AD, MD and RD differences between MDD patients and healthy controls in adults and adolescents, including age, sex, age×sex, age^2^ and age^2^×sex, as covariates. Error bars represent the 95% confidence interval.
