# Supplementary material for: White matter disturbances in major depressive disorder: a coordinated analysis across 20 international cohorts in the ENIGMA MDD working group
Source: Mol Psychiatry. Author manuscript; Available in PMC 2021 Jul 1. (PMC7055351; doi:10.1038/s41380-019-0477-2)
Supplement: Supplementary file 2 [file NIHMS1064748-supplement-Notes.docx]

**Supplementary notes**

**Supplementary Note 1: ENIGMA-DTI protocols**

Image pre-processing

Scanner and acquisition parameters for each site are provided in supplementary table S2. Each site performed pre-processing steps, including eddy current correction, EPI induced distortion correction, and tensor fitting. Tract-based spatial statistics (TBSS), which is part of FSL software (Smith et al., 2006), was used to analyse the images.

Analysis pipeline

First, subject FA maps were aligned to the custom ENIGMA-DTI FA template, which was derived from 400 adults scanned at four sites for optimal multisite harmonization (Jahanshad et al., 2013). Then, FA voxels were projected on the ENIGMA-DTI template skeleton, which creates a unique FA skeleton in the same space for all individuals per site. The same projection was then used for the MD, AD and RD images onto the skeleton. Voxels were then averaged across white matter regions of interest. Twenty-five bilateral ROIs were delineated based on the JHU white matter atlas, which were derived using deterministic tractography (Hua et al., 2008). The whole-brain WM skeleton was defined using TBSS and each of the diffusion measures was averaged over all skeleton voxels for each ROI. Voxels at the periphery of WM bundles were therefore excluded from the ROI average.

Regions of interest

The JHU white matter atlas (Mori et al., 2008) was used to parcellate regions of interest from the ENIGMA template in MNI space. 25 ROIs were extracted from the skeletonized images and averaged (see Table  for the 25 ROIs, some overlapping). These regions include 5 midsaggital regions and 19 lateralized regions (left and right are averaged to obtain bilateral FA). Overall FA values were calculated by averaging values for the entire WM skeleton.

Quality assurance protocol

The quality assuarance (QA) protocol consists of visual inspection of the images before and after registration to the template. The average skeleton projection distance was also calculated, which helps assess the registration quality between images and the ENIGMA-DTI template. After ROI extraction, histograms of FA and diffusivity measures were computed for each region of interest.

 

**Supplementary Note 2: Moderator analyses**

We performed moderator analyses using meta-regression analysis to examine whether site characteristics (percentage of acutely depressed patients and percentage of patients with comorbid anxiety disorders) or diffusion MRI acquisition parameters (field strength and number of directions) significantly explained the variance in effect sizes across sites in the MDD vs. healthy controls comparison. These analyses were performed separately for adults and adolescent samples. A moderator variable was included as a fixed-effect predictor in this meta-regression model. Results were considered significant if they survived FDR-correction (*p*<0.05).

Results show that clinical characteristics (percentage of acutely depressed patients and percentage of patients with a comorbid anxiety disorder) and diffusion MRI acquisition parameters (field strength and number of directions) were not associated with effect sizes of FA, MD, RD and AD differences in adult or adolescent patients compared to healthy controls (data not shown).  

**Supplementary Note 3: UK Biobank image acquisition and processing** 

Diffusion MRI data was acquired using a (“monopolar”) Stejskal-Tanner pulse sequence (Resolution = 2×2×2 mm, FoV = 104×104×72 matrix, TR = 3600ms, TE = 92.00ms, SE-EPI with x3 multislice acceleration, in-plane acceleration = off, fat saturation = on). Ten baseline volumes were collected (b = 0 s/mm^2^), with 50 b=1000 s/mm^2^ and 50 b=2000s/mm^2^. The overall duration was 7 minutes. Standard protocols were used for DTI acquisition, pre-processing and imaging analysis to obtain FA measures of white matter tracts (Miller et al., 2016). Details regarding the processing steps can be found at [https://biobank.ctsu.ox.ac.uk/crystal/docs/brain_mri.pdf](https://biobank.ctsu.ox.ac.uk/crystal/docs/brain_mri.pdf). FA images were alligned onto a white matter skeleton by high-dimensional FNIRT-based warping (de Groot et al., 2013). This warp is then applied to all DTI-based FA, AD and RD maps. The skeletonised images were then averaged across 48 standard-space masks of the different tracts (Mori et al., 2005; Wakana et al., 2007). 

UK biobank diffusion measures were used directly from the UK biobank release, and not re-analyzed with the ENIGMA-DTI protocol. However, there is substantial overlap in steps between both processing protocols (please see the technical report here: [https://github.com/USC-IGC/enigma_ukb_comparison/blob/master/diffusion_comparison.ipynb](https://webmail.geestgronden.nl/owa/redir.aspx?C=IY22TJirP0SeXRBmCRaRsGk7VELJhdZIbOmDWBm1E1Sh4hU3-fgFt7xn0JhJAh5oL64Z-eYbS3w.&URL=https%3a%2f%2furldefense.proofpoint.com%2fv2%2furl%3fu%3dhttps-3A__github.com_USC-2DIGC_enigma-5Fukb-5Fcomparison_blob_master_diffusion-5Fcomparison.ipynb%26d%3dDwMFaQ%26c%3dclK7kQUTWtAVEOVIgvi0NU5BOUHhpN0H8p7CSfnc_gI%26r%3dxwt118oaBd_6H2oWuWYNqZjg7YU5Rgb6jyddci9s5lE%26m%3d4lfflKzwrC3eQLhu0mITzV_LMcTvFTd4FvutrlYW8Rw%26s%3dj1xDdQKgJwjhatpRjwcTWebjAFaNcMpNMJPZSg4Sqow%26e%3d)). DTI and diffusivity measures of most regions of interest show great consistency across pipelines (please see the R coefficients per region below).

R^2^ values of all metrics and ROIs  (adapted from technical report Zhu et al., 2018)

| **ROIs** | **MD** | **FA** | **AD** | **RD** |
| --- | --- | --- | --- | --- |
| PLIC | 0.954 | 0.964 | 0.962 | 0.964 |
| SLF | 0.974 | 0.911 | 0.933 | 0.956 |
| RLIC | 0.957 | 0.918 | 0.947 | 0.932 |
| EC | 0.963 | 0.947 | 0.929 | 0.964 |
| GCC | 0.969 | 0.909 | 0.902 | 0.927 |
| CGC | 0.975 | 0.9 | 0.914 | 0.958 |
| SS | 0.962 | 0.893 | 0.889 | 0.946 |
| ALIC | 0.936 | 0.868 | 0.906 | 0.93 |
| BCC | 0.957 | 0.855 | 0.859 | 0.927 |
| SCR | 0.976 | 0.767 | 0.926 | 0.916 |
| SCC | 0.935 | 0.812 | 0.953 | 0.876 |
| ACR | 0.965 | 0.792 | 0.921 | 0.906 |
| PTR | 0.907 | 0.886 | 0.866 | 0.893 |
| SCP | 0.875 | 0.921 | 0.908 | 0.885 |
| PCR | 0.905 | 0.76 | 0.873 | 0.873 |
| IFO | 0.922 | 0.884 | 0.908 | 0.888 |
| CGH | 0.696 | 0.855 | 0.733 | 0.698 |
| CR | 0.5 | 0.673 | 0.798 | 0.48 |
| FX/ST | 0.353 | 0.908 | 0.181 | 0.674 |
| SFO | 0.617 | 0.382 | 0.636 | 0.512 |
| FX | 0.634 | 0.561 | 0.617 | 0.614 |
| CST | 0.525 | 0.233 | 0.51 | 0.419 |
| UNC | 0.468 | 0.423 | 0.284 | 0.51 |

**Supplementary Note 4: Case-control differences in different ages**

Differences between patients and controls were also analyzed in separate age categories (10-<20 years; 20-<30 years; 30-<40 years; 40-<50 years and 50-<60 years). Due to small sample sizes, data from participants between 60-<70 and 70-<80 were not analysed. In these analysis, only sites were included that had more than 10 patients and 10 controls in this age range. The results are presented in Supplemental Tables S120-S139. 

Results show no significant differences in FA, AD, MD or RD between patients and controls between age 10-20 and 20-30.  FA was lower in the anterior limb of the internal capsule and the posterior corona radiata in patients than in controls between 30 and 40 years of age. In the 40-50 year age range, FA was lower the posterior thalamic radiation in patients compared to controls. Finally, in the 50-60 year age range, MD and RD was higher in patients in the external capsule, and MD was higher in the genu of the corpus callosum in patients compared to controls. However, these results should be interpreted with caution, as the sample size in each group are small and we may have limited power to detect small effects. Furthermore, the sample sizes differ between age bins, which limits the ability to compare results between age ranges. 

**Supplementary Note 5: Age in early and late age of onset patients and controls**

| Site | EAO | LAO | HC |
| --- | --- | --- | --- |
| Barcelona | 46.17 (9.87) | 47.60 (7.34) | 46.41 (7.82) |
| FOR2107 | 29.43 (8.19) | 46.03 (10.88) | 34.73 (12.27) |
| MOTAR | 28.75 (5.72) | 36.95 (8.59) | 34.65 (12.14) |
| Munster | 30.32 (9.12) | 42.40 (10.30) | 38.37 (11.03) |
| NESDA | 46.14 (9.71) | 50.09 (9.95) | 54.91 (8.59) |
| Stanford | 33.82 (8.64) | NA | 32.00 (10.13) |
| Sydney | 24.05 (3.19) | NA | 25.86 (3.37) |
| Trinity | 39.18 (10.12) | 44.76 (10.88) | 38.26 (12.40) |
| DIP | NA | 41.70 (15.13) | 44.50 (14.71) |
| Sexpect | NA | 38.08 (10.69) | 33.75 (7.20) |
| Novosibirsk_site1 | NA | 44.33 (12.57) | 39.17 (8.55) |
| Novosibirsk_site2 | NA | 52.67 (9.26) | 41.58 (9.39) |

Mean age in patients with an early or late age of onset and healthy controls. EAO: early age of onset; LAO; late age of onset; HC: healthy controls
