# Supplementary material for: White matter disturbances in major depressive disorder: a coordinated analysis across 20 international cohorts in the ENIGMA MDD working group
Source: Mol Psychiatry. Author manuscript; Available in PMC 2021 Jul 1. (PMC7055351; doi:10.1038/s41380-019-0477-2)
Supplement: Supplementary file 3 [file NIHMS1064748-supplement-Tables.docx]

**Supplementary tables**

- **Exclusion criteria and image acquisition**

Supplementary Table S1: Instruments used to diagnose MDD and exclusion criteria per site

Supplementary Table S2: Image acquisition parameters and pre-processing steps per site

- **White matter tracts of interest**

Supplementary Table S3: List of white matter tracts of interest

- **Exclusion criteria and image acquisition**

Supplementary Table S2: Instruments used to diagnose MDD and exclusion criteria per site

Supplementary Table S3: Image acquisition parameters and pre-processing steps per site

- **Meta-analysis results for case/control comparisons**

Adults

Supplementary Tables S4-7: Cohen’s d effect sizes after meta-analysis, for FA, AD, MD and RD differences between patients and controls **in adults only**. Age, sex, agexsex, age^2^, age^2^xsex and scansite included as covariates.

Adolescents

Supplementary Tables S8-11: Cohen’s d effect sizes after meta-analysis, for FA, AD, MD and RD differences between patients and controls **in adolescents only**. Age, sex, agexsex, age^2^, age^2^xsex and scansite included as covariates.

- **Meta-analysis results for case/control comparisons after correction for average diffusivity**

Adults

Supplementary Tables S12-15: Cohen’s d effect sizes after meta-analysis, for FA, AD, MD and RD differences between **adult** patients and controls after correction for average FA. Age, sex, agexsex, age^2^, age^2^xsex, averageFA and scansite included as covariates.

Adolescents

Supplementary Tables S16-19: Cohen’s d effect sizes after meta-analysis, for FA, AD, MD and RD differences between **adolescent** patients and controls after correction for average FA. Age, sex, agexsex, age^2^, age^2^xsex, averageFA and scansite included as covariates.

- **Meta-analysis results for the Diagnosis-by-Sex interaction effect**

Adults

Supplementary Tables S20-23: Full meta-analytic results for the Diagnosis by Sex interaction for FA, AD, MD and RD **in adults only**. Age, sex, age^2^ and scansite included as covariates.

Adolescents

Supplementary Tables S24-27: Full meta-analytic results for the Diagnosis by Sex interaction for FA, AD, MD and RD **in adolescents only**. Age, sex, age^2^ and scansite included as covariates.

- **Meta-analysis results for the Diagnosis-by-Age interaction effect**

Supplementary Tables S28-31: Full meta-analytic results for the Diagnosis by Age interaction for FA, AD, MD and RD. Age, sex, age^2^ and scansite included as covariates.

- **Meta-analysis results for the first episode vs. recurrent episodes comparison**

Adults

Supplementary Tables S32-43: Cohen’s d effect sizes after meta-analysis, for FA, AD, MD and RD differences between patients with recurrent MDD, first episode patients and controls **in adults only**. Age, sex, agexsex, age^2^, age^2^xsex and scansite included as covariates.

Adolescents

Supplementary Tables S44-55: Cohen’s d effect sizes after meta-analysis, for FA, AD, MD and RD differences between patients with recurrent MDD, first episode patients and controls **in adolescents only**. Age, sex, agexsex, age^2^, age^2^xsex and scansite included as covariates.

- **Meta-analysis results for the early versus late onset of MDD comparison**

Supplementary Tables S56-67: Cohen’s d effect sizes after meta-analysis, for FA, AD, MD and RD differences between adolescent age of onset MDD patients (age<22 years), adult age of onset patients (age ≥ 22 years) and healthy controls **in adults only**. Age, sex, agexsex, age^2^, age^2^xsex and scansite included as covariates.

- **Meta-analysis results for patients taking antidepressants and patients not taking antidepressants at the time of scanning comparison**

Adults

Supplementary Tables S68-79: Cohen’s d effect sizes after meta-analysis, for FA, AD, MD and RD differences between non-antidepressant users, antidepressant users and healthy controls **in adults only**. Age, sex, agexsex, age^2^, age^2^xsex and scansite included as covariates.

Adolescents

Supplementary Tables S80-91: Cohen’s d effect sizes after meta-analysis, for FA, AD, MD and RD differences between non-antidepressant users, antidepressant users and healthy controls **in adolescents only**. Age, sex, agexsex, age^2^, age^2^xsex and scansite included as covariates.

- **Meta-analysis results for association with severity of symptoms**

Adults

Supplementary Tables S92-95: Beta’s from linear regression analyses examining the association between FA, AD, MD and RD values and severity of symptoms at study inclusion measured by the BDI-II **in adults only**. Age, sex, agexsex, age^2^, age^2^xsex and scansite included as covariates

Supplementary Tables S96-99: Beta’s from linear regression analyses examining the association between FA, AD, MD and RD values and severity of symptoms at study inclusion measured by the HDRS **in adults only**. Age, sex, agexsex, age^2^, age^2^xsex and scansite included as covariates.

Adolescents

Supplementary Tables S100-103: Beta’s from linear regression analyses examining the association between FA, AD, MD and RD values and severity of symptoms at study inclusion measured by the BDI-II **in adolescents only**. Age, sex, agexsex, age^2^, age^2^xsex and scansite included as covariates

Supplementary Tables S104-107: Beta’s from linear regression analyses examining the association between FA, AD, MD and RD values and severity of symptoms at study inclusion measured by the HDRS **in adolescents only**. Age, sex, agexsex, age^2^, age^2^xsex and scansite included as covariates

- **Results for case/control comparisons in UK biobank**

Supplementary Tables S108-111: Cohen’s d effect sizes for FA, AD, MD and RD differences between patients and controls for UK biobank. Age, sex, agexsex, age^2^, age^2^xsex, included as covariates.

- **Meta-analysis results for case/control comparisons after FDR correction for the total number of tests (4 measures * 25 regions)**

Adults

Supplementary Tables S112-115: Cohen’s d effect sizes after meta-analysis, for FA, AD, MD and RD differences between patients and controls **in adults only**. Results are FDR-corrected for the total number of tests (4 metrics x 25 ROIs). Age, sex, agexsex, age^2^, age^2^xsex and scansite included as covariates.

Adolescents

Supplementary Tables S116-119: Cohen’s d effect sizes after meta-analysis, for FA, AD, MD and RD differences between patients and controls **in adolescents only**. Results are FDR-corrected for the total number of tests (4 metrics x 25 ROIs). Age, sex, agexsex, age^2^, age^2^xsex and scansite included as covariates.

- **Meta-analysis results for case/control comparisons in different age bins**

Supplementary Tables S120-S139: Cohen’s d effect sizes after meta-analysis, for FA, AD, MD and RD differences between patients and controls in different age bins: 10-20, 20-30, 30-40, 40-50, 50-60 years of age. Age, sex, agexsex, age^2^, age^2^xsex, included as covariates.

- **Meta-analysis results for case/control comparisons in the age range of UK biobank patients only**

Supplementary Tables S140-S143: Cohen’s d effect sizes after meta-analysis, for FA, AD, MD and RD differences between patients and controls in the age range of UK biobank (> 42 years of age). Age, sex, agexsex, age^2^, age^2^xsex, included as covariates.

Table S1. Instruments used to diagnose MDD and exclusion criteria per site

| Study | Sample | Instrument for diagnosis of MDD | Exclusion criteria |
| --- | --- | --- | --- |
| 1 | Barcelona | DSM-IV-TR | Left-handedness, previous or current history of medical or neurologic illness, substance abuse, aged above 65, IQ<85. For controls also, lifetime psychiatric diagnoses, first-degree relatives with psychiatric diagnoses and clinically important physical or neurologic illness and psychotropic medication. |
| 2 | Bipolar family study | SCID | Individuals were initially recruited as unaffected familial risk participants for a longitudinal study. Initial exclusion criteria for both high risk and control groups included a personal history of major depression, mania or hypomania, psychosis, or any major neurological or psychiatric disorder, a history of substance dependence, learning disability, or any history of head injury that included loss of consciousness and any contraindications to magnetic resonance imaging. Those who were subsequently diagnosed as MDD at second assessment with scanning data are included here as MDD cases. |
| 3 | CODE | SCID | MDD: Presence of any other Axis-1 diagnosis; Acute risk for suicide (in contrast to suicidal ideation); History of psychotic symptoms, bipolar disorder, or dementia; Schizotypal, antisocial or borderline personality disorder; Use of psychotropic medication within two weeks prior to the start of the study; No current psychotherapeutic treatment. Control subjects: No history of or current Axis-1 or 2 disorders. All subjects: History of or current neurological disorder or brain injury; Serious medical condition; Severe cognitive impairment; Substance-related abuse or dependence disorder; Use of psychotropic medication; Use of central-acting medication; Pregnancy; General MRI contraindications. |
| 4 | DIP | MINI-SCAN | Age younger than 18 years, neurological problems, end-stage renal disease, cerebro-, and cardiovascular disease, inadequate language comprehension, concrete suicidal plans, general MRI-contraindications, current use of psychotropic medication other than SSRI/SNRI/TCA or infrequent benzodiazepine use. For patients: lifetime psychiatric diagnosis other than a depressive or anxiety disorder. For controls: no lifetime psychiatric diagnosis (history of moderate substance use, history of or current nicotine dependence allowed in patient and control groups if no gross anatomical abnormalities). |
| 5 | Sexpect |  | Exclusion criteria were major medical illness, history of seizures, prior electroconvulsive therapy treatments as well as all contraindications against MRI. Speciﬁc psychiatric exclusion criteria consisted of atypical forms of depression, any additional psychiatric disorder and a history of substance abuse or dependence. |
| 6 | EPISCA | ADIS | Left-handedness, current substance abuse, history of neurological disorders or severe head injury, age below 12 or higher than 21, pregnancy, intelligence scores below 80, general MRI contraindications. For patients, diagnosis of ADHD, PDD, PTSD, Tourette’s, OCD, bipolar disorder or psychotic disorders and current use of psychotropic medication, other than stable SSRI use. |
| 7 | MOTAR | CIDI | Severe internal or neurological disorders, MRI contraindications, use of antidepressants or other psychoactive medication (with the exception of stable benzodiazepine use for patients), lifetime diagnosis of psychotic disorders, bipolar disorder or personality disporders and dependence on drugs or alcohol. |
| 8 | MPIP | SCAN | Incidental MR findings that prevent image processing. Presence of manic episodes, mood incongruent psychotic symptoms, the presence of a lifetime diagnosis of intravenous drug abuse and depressive symptoms only secondary to alcohol or substance abuse or to medical illness or medication for patients. Presence of severe somatic diseases or life-time history of anxiety and affective disorders according to the Composite International Diagnostic-Screener (CIDI-S) for healthy controls. |
| 9 | Muenster cohort | SCID | Inclusion criteria: age 17-65 years; Exclusion criteria all: any MRI contraindications; Exclusion criteria controls: any current or former psychiatric disorder; Exclusion criteria patients: any neurological abnormalities, substance-related disorders or current benzodiazepine treatment (wash out of at least three half-lives before study participation), and former electroconvulsive therapy, bipolar disorder |
| 10 | NESDA | CIDI | Severe internal or neurological disorders, insulin dependence, neurological trauma with loss of consciousness, general MRI contraindications, a history of drug or alcohol dependence. Exclusion criteria for patients include use of psychotroic medication, with the exception of stable use (>4 weeks daily intake) of antidepressants (TCA & SSRI), low dose of atypical antipsychotic medication, lithium use or infrequent (<3 days per week) use of benzodiazepines. Exclusion criteria for patients were use of psychoactive medication, with the exception of infrequent benzodiazepine use (<1 day per week). All participants were asked not to use benzodiazepines, soft- or harddrugs in the 48 hours preceding scanning. |
| 11 | Novosibirsk | MINI, SCID, ICD-10 interviews | Presence of axis-I disorders other than MDD, panic disorder, social anxiety disorder, or generalized anxiety disorder and any use of psychotropic medication other than stable use of SSRIs or infrequent benzodiazepine use; age 18 or below; alcohol or substance abuse/dependence within 6 months of study participation; current major medical problems for patients. Age over 65; any current or former psychiatric disorder were exclusion criteria for patients. |
| 12 | QTIM | CIDI | Presence of axis-I disorders other than MDD and anxiety disorders for all patients Use of antidepressant use or presence of a psychiatric disorder for controls All subjects, left handedness, history of neurological or other severe medical illness, head injury or current or past diagnosis of substance abuse, use of cognition affecting medication and general MRI contraindications |
| 13 | Sydney | DSM-IV | Neurological diseases, medical illness known to impact cognitive and brain function, medical instability (as determined by a psychiatrist), intellectual and/or developmental disability, insufficient English, current substance dependence. Hazardous alcohol consumption (determined by the AUDIT) or a history of excessive substance abuse were also exclusion criteria. |
| 14 | Imaging Genetics Dublin | SCID | Axis-I disorders other than depression for patients, use of antipsychotic medication, alcohol or drug dependency, axis II disorders, severe medical diseases, commotion cerebri and head injuries in the past, glucocorticoid therapy |
| 15 | UCSF | KSADS | Wechsler Abbreviated Scale of Intelligence (WASI) scores of less than 70, contraindications for MRI, prepubertal status (Tanner stage 1 or 2), substance abuse, history of neurological disorders, misuse of prescription drugs, drinking more than two alcoholic drinks per week, use of medication with effect on the CNS in the two weeks preceding scanning. Diagnosis of psychosis, bipolar disorder or substance abuse for patients. Axis I disorders or a family history of mood or psychotic disorders in controls. |
| 16 | Child and Adolescent Imaging Research Calgary | KSADS | Left handedness, history of seizures, epilepsy or other neurological disorders, pregnancy. For patients, diagnosis of bipolar disorders, psychosis, pervasive developmental disorders, eating disorders and PTSD were exclusion criteria. |
| 17 | FOR2017 | SCID | Age under 18 and over 65, no current or previous neurological or cardiovascular disorders, no current or previous severe physical disorders (such as cancer, autoimmune diseases etc), no substance-dependence (abuse is ok), or polytoxicomania, no current benzodiazepine use, no history of head injury (+ all other conditions that might affect MRI quality or safety) |
| 18 | University of Minnesota | KSADS | Age under 12 and over 18, history of intellectual disability, neurological or chronic medical condition, meeting DSM-IV-R criteria for pervasive developmental disorder, bipolar disorder, or psychotic disorder, current substance use disorder, IQ < 80 |
| 19 | Stanford | SCID | Cardiovascular or neurological disorders, history of head injury, hyper- or hypothyroidism, history of any psychiatric disorder or substance abuse/dependence for controls and diagnosis of bipolar disorder, psychosis or substance abuse/dependence in the past six months for patients. |
| 20 | IMH MDD Singapore | SCID | History of significant head injury, neurological diseases such as epilepsy, cerebrovascular accident, impaired thyroid function, steroid use, DSM IV alcohol or substance use or dependence, contraindications to MRI (eg pacemaker, orbital foreign body, recent surgery or procedures with metallic devices/implants inserted) and pregnant women |

Table S2. Image acquisition parameters and pre-processing steps per site

| Study | Sample | Scanner type | Field strength | Voxel size | Slice thickness | Number of directions & b-factor | Preprocessing software & steps |
| --- | --- | --- | --- | --- | --- | --- | --- |
| 1 | Barcelona | Philips Achieva | 3T | 1.75 x 1.75  x 2 mm | 2 mm | 16 directions; 1 b0 image; b-factor = 800 | Eddy current & motion correction FDT (FSL) |
| 2 | Bipolar family study | GE Signa Horizon | 1.5T | 2.5 x 2.5 mm | 2.5 mm | 64 directions; b-factor = 1000 | Eddy current and motion correction in FSL |
| 3 | CODE | Siemens Trio (3 Sites), Philips Achieva (1 site) | 3T | 1.7 X 1.7 mm (1 site) 2.5 x 2.5 mm (3 sites) | 1.7 mm (1 site) 2.5 mm (3 sites) | 60 directions; 6 b0 images; b-factor = 1000 (1 site) 64 directions; 4 b0 images; b-factor = 1000 (3 sites) | Eddy current and motion correction in FSL |
| 4 | DIP | Philips Intera | 3T | 2.5 x 2.5 mm | 2.5 mm | 32 directions; 1 b0 image; b-factor = 1000 | Eddy current and motion correction in FSL |
| 5 | Sexpect | Siemens Trio | 3T | 2 x 2 mm | 2 mm | 12 directions; 1 b0 image; b-factor = 1000 | Eddy current and motion correction in FSL |
| 6 | EPISCA | Philips Achieva | 3T | 2.3 x 2.3 mm | 2.3 mm | 32 directions; 1 b0 image; b-factor = 1000 | Eddy current and motion correction in FSL |
| 7 | MOTAR | Philips Achieva | 3T | 2 x 2 mm | 2 mm | 32 directions; 1 b0 image; b-factor = 1000 | Eddy current. motion and EPI correction and matrix rotation in ExploreDTI |
| 8 | MPIP | GE Signa Excite | 1.5 T | 1.875 x 1.875 mm 1.964 x 1.964 mm | 3 mm | 6 directions 15 directions |  |
| 9 | Muenster cohort | Philips Gyroscan Intera | 3T | 1.8 x1.8 mm | 3.6 mm | 25 directions; 5 b0 images; b-factor = 1000 | Eddy current & motion correction in ACID Toolbox |
| 10 | NESDA | Philips Achieva | 3T | 1.88 x 2.35 mm | 2 mm | 30 directions; 1 b0 image; b-factor = 1000 | TOPUP,  eddy current and motion correction and matrix rotation in FSL |
| 11 | Novosibirsk | GE Discovery MR750w | 3T | 2 x 2 mm | 2 mm | 64 directions; 7 b0 images; b-factor = 1000 | eddy_correct, EPI correction in FSL and ANTs |
| 12 | QTIM |  |  |  |  |  |  |
| 13 | Sydney | GE Discovery MR750 | 3T | 0.859 x 0.859 mm | 2 mm | 69 directions; 8 b0 images; b-factor = 1159 | Eddy current & motion correction in FSL |
| 14 | Imaging Genetics Dublin | Philips Achieva | 3T | 1.8 x1.8 mm | 2.1 mm | 61 directions; 1 b0 image;  b-factor = 1200 | Eddy current, motion & EPI correction in Explore DTI |
| 15 | UCSF | GE Discovery MR750 | 3T | 1.875 x 1.875 mm | 2.5 mm | 30 directions; 1 b0 image; b-factor = 1500 | Fieldmap correction, eddy current correction, matrix rotation in FSL & AFNI |
| 16 | Child and Adolescent Imaging Research Calgary | GE Discovery MR750 | 3T | 2.2 x 2.2 mm | 2.2 mm | 26 directions; 3 b0 images; b-factor = 900 | eddy current and motion correction and matrix rotation in FSL |
| 17 | FOR2017 | Siemens Magnetom Trio, a trio system | 3T | 2.5 x 2.5 mm | 2.5 mm | 30 directions; 4b images; b-factor = 1000 |  |
| 18 | University of Minnesota | Siemens Trio | 3T | 2 x 2 mm | 2 mm | 64 directions; b-factor=1000 | Eddy current and distortion correction in FSL |
| 19 | Stanford | GE Discovery MR750 | 3T | 0.859 x 0.859 mm | 2.0 mm | 96 directions; 9 b0 images; b-factor=2000 | Eddy current and motion correction & bvec rotation in FSL |
| 20 | IMH MDD Singapore | Philips Achieva | 3T | 1 x 1 x 1 mm | 3.0 mm | 32 directions; 1 b0 image; b-factor=800 | Eddy current and motion correction in FSL |

Table S3: List of white matter tracts of interest

| Abbreviation | Full tract name |
| --- | --- |
| AverageFA | Full skeleton average FA |
| ACR (L+R) | Anterior corona radiata |
| ALIC (L+R) | Anterior limb of internal capsule |
| BCC | Body of corpus callosum |
| CC (BCC+GCC+SCC) | Corpus callosum |
| CGC (L+R) | Cingulum (cingulate gyrus) |
| CGH (L+R) | Cingulum (hippocampal portion) |
| CR (L+R) | Corona radiata |
| CST (L+R) | Corticospinal tract |
| EC (L+R) | External capsule |
| FX | Fornix |
| FXST (L+R) | Fornix (cres) / Stria terminalis |
| GCC | Genu of corpus callosum |
| IC (L+R) | Internal capsule |
| IFO (L+R) | Inferior fronto-occipital fasciculus |
| PCR (L+R) | Posterior corona radiata |
| PLIC (L+R) | Posterior limb of internal capsule |
| PTR (L+R) | Posterior thalamic radiation |
| RLIC (L+R) | Retrolenticular part of internal capsule |
| SCC | Splenium of corpus callosum |
| SCR (L+R) | Superior corona radiata |
| SFO (L+R) | Superior fronto-occipital fasciculus |
| SLF (L+R) | Superior longitudinal fasciculus |
| SS (L+R) | Sagittal stratum |
| UNC (L+R) | Uncinate fasciculus |

**1: Effect of MDD diagnosis**

Table S4. Cohen’s d effect sizes after meta-analysis, for FA differences between patients and controls **in adults only**. Age, sex, agexsex, age^2^, age^2^xsex and scansite included as covariates.

| Region | Cohen’s d | SE | CI LB | CI UB | P-value | FDR P-value | I^2^ | Controls | Patients |
| --- | --- | --- | --- | --- | --- | --- | --- | --- | --- |
| ACR | -0.253 | 0.079 | -0.408 | -0.098 | 0.001 | 0.006 | 54.984 | 1265 | 921 |
| ALIC | -0.232 | 0.080 | -0.388 | -0.076 | 0.004 | 0.008 | 55.406 | 1265 | 921 |
| BCC | -0.243 | 0.080 | -0.401 | -0.086 | 0.002 | 0.006 | 56.195 | 1265 | 921 |
| CC | -0.249 | 0.080 | -0.406 | -0.092 | 0.002 | 0.006 | 55.820 | 1265 | 921 |
| CGC | -0.165 | 0.053 | -0.268 | -0.062 | 0.002 | 0.006 | 11.516 | 1265 | 921 |
| CGH | -0.068 | 0.046 | -0.157 | 0.022 | 0.138 | 0.144 | 0.000 | 1265 | 921 |
| CR | -0.250 | 0.080 | -0.407 | -0.093 | 0.002 | 0.006 | 55.811 | 1265 | 921 |
| CST | -0.101 | 0.062 | -0.221 | 0.020 | 0.101 | 0.115 | 28.658 | 1265 | 921 |
| EC | -0.160 | 0.067 | -0.291 | -0.029 | 0.016 | 0.027 | 37.605 | 1265 | 921 |
| FX | -0.083 | 0.049 | -0.179 | 0.012 | 0.087 | 0.104 | 5.409 | 1265 | 921 |
| FXST | -0.183 | 0.062 | -0.305 | -0.061 | 0.003 | 0.008 | 29.984 | 1265 | 921 |
| GCC | -0.247 | 0.077 | -0.398 | -0.095 | 0.001 | 0.006 | 52.666 | 1265 | 921 |
| IC | -0.229 | 0.085 | -0.395 | -0.063 | 0.007 | 0.013 | 60.650 | 1265 | 921 |
| IFO | -0.121 | 0.048 | -0.214 | -0.027 | 0.012 | 0.021 | 3.601 | 1265 | 921 |
| PCR | -0.202 | 0.071 | -0.341 | -0.063 | 0.004 | 0.008 | 44.138 | 1265 | 921 |
| PLIC | -0.154 | 0.089 | -0.329 | 0.021 | 0.084 | 0.104 | 64.810 | 1265 | 921 |
| PTR | -0.135 | 0.086 | -0.303 | 0.033 | 0.116 | 0.126 | 61.851 | 1265 | 921 |
| RLIC | -0.151 | 0.079 | -0.305 | 0.003 | 0.054 | 0.071 | 54.283 | 1265 | 921 |
| SCC | -0.130 | 0.063 | -0.254 | -0.006 | 0.039 | 0.054 | 31.342 | 1265 | 921 |
| SCR | -0.197 | 0.086 | -0.366 | -0.029 | 0.022 | 0.034 | 61.930 | 1265 | 921 |
| SFO | -0.232 | 0.064 | -0.357 | -0.107 | 0.000 | 0.000 | 32.299 | 1265 | 921 |
| SLF | -0.169 | 0.081 | -0.327 | -0.011 | 0.037 | 0.054 | 56.865 | 1265 | 921 |
| SS | -0.230 | 0.080 | -0.387 | -0.073 | 0.004 | 0.008 | 56.069 | 1265 | 921 |
| UNC | -0.120 | 0.088 | -0.291 | 0.052 | 0.172 | 0.172 | 63.560 | 1265 | 921 |
| AverageFA | -0.258 | 0.073 | -0.403 | -0.114 | 0.000 | 0.000 | 47.560 | 1265 | 921 |

Table S5. Cohen’s d effect sizes after meta-analysis, for AD differences between patients and controls **in adults only**. Age, sex, agexsex, age^2^, age^2^xsex and scansite included as covariates.

| Region | Cohen’s d | SE | CI LB | CI UB | P-value | FDR P-value | I^2^ | Controls | Patients |
| --- | --- | --- | --- | --- | --- | --- | --- | --- | --- |
| ACR | -0.002 | 0.100 | -0.198 | 0.194 | 0.984 | 0.984 | 72.857 | 1265 | 921 |
| ALIC | 0.083 | 0.101 | -0.115 | 0.281 | 0.411 | 0.650 | 73.242 | 1265 | 921 |
| BCC | 0.101 | 0.099 | -0.093 | 0.296 | 0.306 | 0.650 | 72.078 | 1265 | 921 |
| CC | 0.095 | 0.104 | -0.110 | 0.299 | 0.366 | 0.650 | 75.131 | 1265 | 921 |
| CGC | -0.027 | 0.094 | -0.211 | 0.158 | 0.778 | 0.810 | 68.886 | 1265 | 921 |
| CGH | 0.069 | 0.088 | -0.104 | 0.241 | 0.433 | 0.650 | 63.992 | 1265 | 921 |
| CR | 0.053 | 0.093 | -0.130 | 0.236 | 0.572 | 0.650 | 68.391 | 1265 | 921 |
| CST | 0.102 | 0.087 | -0.068 | 0.272 | 0.240 | 0.650 | 62.841 | 1265 | 921 |
| EC | 0.089 | 0.097 | -0.101 | 0.278 | 0.358 | 0.650 | 70.597 | 1265 | 921 |
| FX | 0.141 | 0.086 | -0.028 | 0.309 | 0.102 | 0.650 | 61.952 | 1265 | 921 |
| FXST | 0.065 | 0.089 | -0.110 | 0.240 | 0.466 | 0.650 | 65.066 | 1265 | 921 |
| GCC | 0.038 | 0.101 | -0.159 | 0.235 | 0.706 | 0.767 | 73.030 | 1265 | 921 |
| IC | 0.095 | 0.096 | -0.093 | 0.284 | 0.321 | 0.650 | 70.213 | 1265 | 921 |
| IFO | 0.058 | 0.096 | -0.130 | 0.245 | 0.545 | 0.650 | 69.753 | 1265 | 921 |
| PCR | 0.076 | 0.095 | -0.109 | 0.262 | 0.421 | 0.650 | 69.284 | 1265 | 921 |
| PLIC | 0.174 | 0.087 | 0.004 | 0.344 | 0.045 | 0.650 | 62.797 | 1265 | 921 |
| PTR | 0.127 | 0.092 | -0.053 | 0.308 | 0.167 | 0.650 | 67.504 | 1265 | 921 |
| RLIC | 0.073 | 0.101 | -0.124 | 0.271 | 0.467 | 0.650 | 73.152 | 1265 | 921 |
| SCC | 0.125 | 0.097 | -0.066 | 0.315 | 0.200 | 0.650 | 70.877 | 1265 | 921 |
| SCR | 0.139 | 0.085 | -0.029 | 0.306 | 0.104 | 0.650 | 61.516 | 1265 | 921 |
| SFO | 0.067 | 0.105 | -0.140 | 0.273 | 0.526 | 0.650 | 75.530 | 1265 | 921 |
| SLF | 0.102 | 0.093 | -0.080 | 0.284 | 0.272 | 0.650 | 67.946 | 1265 | 921 |
| SS | 0.051 | 0.090 | -0.126 | 0.228 | 0.572 | 0.650 | 65.983 | 1265 | 921 |
| UNC | 0.066 | 0.114 | -0.158 | 0.290 | 0.564 | 0.650 | 79.609 | 1265 | 921 |
| AverageAD | 0.123 | 0.086 | -0.046 | 0.293 | 0.154 | 0.650 | 62.693 | 1265 | 921 |

Table S6. Cohen’s d effect sizes after meta-analysis, for MD differences between patients and controls **in adults only**. Age, sex, agexsex, age^2^, age^2^xsex and scansite included as covariates.

| Region | Cohen’s d | SE | CI LB | CI UB | P-value | FDR P-value | I^2^ | Controls | Patients |
| --- | --- | --- | --- | --- | --- | --- | --- | --- | --- |
| ACR | 0.065 | 0.088 | -0.107 | 0.236 | 0.459 | 0.478 | 63.620 | 1265 | 921 |
| ALIC | 0.124 | 0.100 | -0.073 | 0.320 | 0.217 | 0.258 | 72.639 | 1265 | 921 |
| BCC | 0.192 | 0.092 | 0.012 | 0.371 | 0.036 | 0.172 | 66.786 | 1265 | 921 |
| CC | 0.170 | 0.091 | -0.009 | 0.349 | 0.062 | 0.172 | 66.547 | 1265 | 921 |
| CGC | 0.085 | 0.083 | -0.078 | 0.248 | 0.306 | 0.333 | 59.337 | 1265 | 921 |
| CGH | 0.140 | 0.073 | -0.002 | 0.283 | 0.054 | 0.172 | 47.001 | 1265 | 921 |
| CR | 0.120 | 0.078 | -0.032 | 0.272 | 0.122 | 0.191 | 53.301 | 1265 | 921 |
| CST | 0.104 | 0.073 | -0.039 | 0.246 | 0.154 | 0.226 | 46.829 | 1265 | 921 |
| EC | 0.148 | 0.084 | -0.017 | 0.313 | 0.078 | 0.189 | 60.384 | 1265 | 921 |
| FX | 0.123 | 0.078 | -0.031 | 0.277 | 0.116 | 0.191 | 54.034 | 1265 | 921 |
| FXST | 0.180 | 0.075 | 0.033 | 0.327 | 0.017 | 0.158 | 50.051 | 1265 | 921 |
| GCC | 0.102 | 0.077 | -0.049 | 0.253 | 0.184 | 0.236 | 52.506 | 1265 | 921 |
| IC | 0.104 | 0.079 | -0.051 | 0.258 | 0.189 | 0.236 | 54.668 | 1265 | 921 |
| IFO | 0.093 | 0.058 | -0.020 | 0.206 | 0.106 | 0.191 | 21.769 | 1265 | 921 |
| PCR | 0.143 | 0.067 | 0.012 | 0.275 | 0.033 | 0.172 | 38.082 | 1265 | 921 |
| PLIC | 0.105 | 0.076 | -0.044 | 0.255 | 0.166 | 0.231 | 51.329 | 1265 | 921 |
| PTR | 0.111 | 0.071 | -0.027 | 0.250 | 0.115 | 0.191 | 43.877 | 1265 | 921 |
| RLIC | 0.075 | 0.073 | -0.069 | 0.218 | 0.306 | 0.333 | 47.523 | 1265 | 921 |
| SCC | 0.143 | 0.082 | -0.018 | 0.304 | 0.083 | 0.189 | 58.472 | 1265 | 921 |
| SCR | 0.192 | 0.067 | 0.062 | 0.323 | 0.004 | 0.100 | 37.170 | 1265 | 921 |
| SFO | 0.164 | 0.083 | 0.001 | 0.328 | 0.048 | 0.172 | 59.449 | 1265 | 921 |
| SLF | 0.126 | 0.076 | -0.023 | 0.276 | 0.098 | 0.191 | 51.613 | 1265 | 921 |
| SS | 0.120 | 0.064 | -0.005 | 0.246 | 0.061 | 0.172 | 33.189 | 1265 | 921 |
| UNC | 0.052 | 0.089 | -0.123 | 0.226 | 0.563 | 0.563 | 64.948 | 1265 | 921 |
| AverageMD | 0.176 | 0.075 | 0.029 | 0.324 | 0.019 | 0.158 | 50.183 | 1265 | 921 |

Table S7. Cohen’s d effect sizes after meta-analysis, for RD differences between patients and controls **in adults only**. Age, sex, agexsex, age^2^, age^2^xsex and scansite included as covariates.

| Region | Cohen’s d | SE | CI LB | CI UB | P-value | FDR P-value | I^2^ | Controls | Patients |
| --- | --- | --- | --- | --- | --- | --- | --- | --- | --- |
| ACR | 0.092 | 0.068 | -0.040 | 0.225 | 0.172 | 0.253 | 0.172 | 1265 | 921 |
| ALIC | 0.128 | 0.061 | 0.008 | 0.249 | 0.036 | 0.090 | 0.036 | 1265 | 921 |
| BCC | 0.174 | 0.067 | 0.043 | 0.304 | 0.009 | 0.032 | 0.009 | 1265 | 921 |
| CC | 0.095 | 0.062 | -0.027 | 0.216 | 0.127 | 0.212 | 0.127 | 1265 | 921 |
| CGC | 0.139 | 0.071 | -0.001 | 0.279 | 0.051 | 0.116 | 0.051 | 1265 | 921 |
| CGH | 0.154 | 0.059 | 0.038 | 0.271 | 0.009 | 0.032 | 0.009 | 1265 | 921 |
| CR | 0.122 | 0.046 | 0.032 | 0.211 | 0.008 | 0.032 | 0.008 | 1265 | 921 |
| CST | 0.091 | 0.061 | -0.028 | 0.210 | 0.136 | 0.213 | 0.136 | 1265 | 921 |
| EC | 0.158 | 0.071 | 0.019 | 0.297 | 0.025 | 0.069 | 0.025 | 1265 | 921 |
| FX | 0.118 | 0.073 | -0.025 | 0.260 | 0.106 | 0.204 | 0.106 | 1265 | 921 |
| FXST | 0.176 | 0.061 | 0.058 | 0.295 | 0.004 | 0.031 | 0.004 | 1265 | 921 |
| GCC | 0.060 | 0.078 | -0.094 | 0.213 | 0.445 | 0.530 | 0.445 | 1265 | 921 |
| IC | 0.057 | 0.063 | -0.066 | 0.179 | 0.366 | 0.482 | 0.366 | 1265 | 921 |
| IFO | 0.062 | 0.066 | -0.067 | 0.191 | 0.345 | 0.479 | 0.345 | 1265 | 921 |
| PCR | 0.096 | 0.063 | -0.027 | 0.220 | 0.127 | 0.212 | 0.127 | 1265 | 921 |
| PLIC | 0.014 | 0.085 | -0.153 | 0.181 | 0.873 | 0.905 | 0.873 | 1265 | 921 |
| PTR | 0.026 | 0.098 | -0.166 | 0.217 | 0.793 | 0.901 | 0.793 | 1265 | 921 |
| RLIC | 0.007 | 0.055 | -0.101 | 0.114 | 0.905 | 0.905 | 0.905 | 1265 | 921 |
| SCC | 0.083 | 0.101 | -0.116 | 0.281 | 0.414 | 0.518 | 0.414 | 1265 | 921 |
| SCR | 0.158 | 0.046 | 0.068 | 0.247 | 0.001 | 0.025 | 0.001 | 1265 | 921 |
| SFO | 0.129 | 0.046 | 0.039 | 0.218 | 0.005 | 0.031 | 0.005 | 1265 | 921 |
| SLF | 0.082 | 0.046 | -0.007 | 0.172 | 0.072 | 0.150 | 0.072 | 1265 | 921 |
| SS | 0.106 | 0.046 | 0.016 | 0.195 | 0.020 | 0.063 | 0.020 | 1265 | 921 |
| UNC | -0.013 | 0.081 | -0.172 | 0.145 | 0.868 | 0.905 | 0.868 | 1265 | 921 |
| AverageRD | 0.152 | 0.051 | 0.052 | 0.253 | 0.003 | 0.031 | 0.003 | 1265 | 921 |

Table S8. Cohen’s d effect sizes after meta-analysis, for FA differences between patients and controls **in adolescents only**. Age, sex, agexsex, age^2^, age^2^xsex and scansite included as covariates.

| Region | Cohen’s d | SE | CI LB | CI UB | P-value | FDR P-value | I^2^ | Controls | Patients |
| --- | --- | --- | --- | --- | --- | --- | --- | --- | --- |
| ACR | -0.040 | 0.141 | -0.316 | 0.235 | 0.773 | 0.885 | 55.139 | 290 | 372 |
| ALIC | 0.064 | 0.160 | -0.250 | 0.377 | 0.691 | 0.885 | 65.133 | 290 | 372 |
| BCC | -0.220 | 0.090 | -0.397 | -0.043 | 0.015 | 0.375 | 0.000 | 290 | 372 |
| CC | -0.195 | 0.090 | -0.372 | -0.018 | 0.031 | 0.388 | 0.000 | 290 | 372 |
| CGC | 0.068 | 0.143 | -0.211 | 0.347 | 0.634 | 0.885 | 56.353 | 290 | 372 |
| CGH | 0.098 | 0.135 | -0.165 | 0.362 | 0.465 | 0.885 | 51.107 | 290 | 372 |
| CR | -0.046 | 0.127 | -0.296 | 0.203 | 0.716 | 0.885 | 45.724 | 290 | 372 |
| CST | -0.051 | 0.110 | -0.266 | 0.165 | 0.645 | 0.885 | 28.635 | 290 | 372 |
| EC | 0.254 | 0.157 | -0.053 | 0.561 | 0.104 | 0.650 | 63.436 | 290 | 372 |
| FX | 0.019 | 0.098 | -0.172 | 0.210 | 0.847 | 0.885 | 11.427 | 290 | 372 |
| FXST | 0.041 | 0.134 | -0.221 | 0.303 | 0.760 | 0.885 | 50.207 | 290 | 372 |
| GCC | -0.170 | 0.113 | -0.392 | 0.053 | 0.134 | 0.670 | 32.256 | 290 | 372 |
| IC | 0.126 | 0.113 | -0.097 | 0.348 | 0.268 | 0.885 | 32.091 | 290 | 372 |
| IFO | -0.041 | 0.147 | -0.328 | 0.247 | 0.782 | 0.885 | 58.632 | 290 | 372 |
| PCR | -0.029 | 0.090 | -0.206 | 0.148 | 0.750 | 0.885 | 0.000 | 290 | 372 |
| PLIC | 0.156 | 0.090 | -0.021 | 0.333 | 0.084 | 0.650 | 0.000 | 290 | 372 |
| PTR | 0.048 | 0.102 | -0.152 | 0.248 | 0.636 | 0.885 | 17.924 | 290 | 372 |
| RLIC | 0.072 | 0.090 | -0.105 | 0.249 | 0.426 | 0.885 | 0.000 | 290 | 372 |
| SCC | -0.048 | 0.090 | -0.225 | 0.128 | 0.592 | 0.885 | 0.000 | 290 | 372 |
| SCR | -0.059 | 0.118 | -0.289 | 0.172 | 0.617 | 0.885 | 37.020 | 290 | 372 |
| SFO | -0.022 | 0.117 | -0.251 | 0.208 | 0.854 | 0.885 | 36.151 | 290 | 372 |
| SLF | 0.015 | 0.103 | -0.188 | 0.218 | 0.885 | 0.885 | 20.215 | 290 | 372 |
| SS | 0.035 | 0.117 | -0.194 | 0.264 | 0.767 | 0.885 | 36.024 | 290 | 372 |
| UNC | 0.065 | 0.090 | -0.112 | 0.242 | 0.473 | 0.885 | 0.000 | 290 | 372 |
| AverageFA | -0.020 | 0.135 | -0.284 | 0.244 | 0.882 | 0.885 | 51.141 | 290 | 372 |

Table S9. Cohen’s d effect sizes after meta-analysis, for AD differences between patients and controls **in adolescents only**. Age, sex, agexsex, age^2^, age^2^xsex and scansite included as covariates.

| Region | Cohen’s d | SE | CI LB | CI UB | P-value | FDR P-value | I^2^ | Controls | Patients |
| --- | --- | --- | --- | --- | --- | --- | --- | --- | --- |
| ACR | 0.085 | 0.090 | -0.091 | 0.262 | 0.343 | 0.572 | 0.000 | 290 | 372 |
| ALIC | 0.162 | 0.091 | -0.015 | 0.340 | 0.073 | 0.530 | 0.007 | 290 | 372 |
| BCC | -0.038 | 0.090 | -0.215 | 0.139 | 0.672 | 0.840 | 0.002 | 290 | 372 |
| CC | -0.004 | 0.090 | -0.181 | 0.173 | 0.967 | 1.000 | 0.007 | 290 | 372 |
| CGC | 0.205 | 0.127 | -0.043 | 0.454 | 0.106 | 0.530 | 44.994 | 290 | 372 |
| CGH | 0.035 | 0.099 | -0.158 | 0.229 | 0.722 | 0.860 | 13.828 | 290 | 372 |
| CR | 0.093 | 0.093 | -0.088 | 0.274 | 0.315 | 0.572 | 3.681 | 290 | 372 |
| CST | -0.104 | 0.115 | -0.330 | 0.122 | 0.368 | 0.574 | 34.420 | 290 | 372 |
| EC | 0.189 | 0.110 | -0.026 | 0.404 | 0.085 | 0.530 | 27.886 | 290 | 372 |
| FX | 0.092 | 0.122 | -0.148 | 0.332 | 0.453 | 0.629 | 40.931 | 290 | 372 |
| FXST | 0.180 | 0.090 | 0.003 | 0.358 | 0.046 | 0.530 | 0.000 | 290 | 372 |
| GCC | 0.098 | 0.090 | -0.079 | 0.275 | 0.278 | 0.572 | 0.000 | 290 | 372 |
| IC | 0.129 | 0.097 | -0.061 | 0.318 | 0.183 | 0.547 | 9.860 | 290 | 372 |
| IFO | 0.099 | 0.090 | -0.078 | 0.276 | 0.273 | 0.572 | 0.000 | 290 | 372 |
| PCR | 0.094 | 0.144 | -0.189 | 0.377 | 0.515 | 0.678 | 57.539 | 290 | 372 |
| PLIC | 0.006 | 0.107 | -0.204 | 0.215 | 0.957 | 1.000 | 24.671 | 290 | 372 |
| PTR | 0.132 | 0.153 | -0.168 | 0.432 | 0.390 | 0.574 | 61.882 | 290 | 372 |
| RLIC | 0.156 | 0.090 | -0.021 | 0.333 | 0.084 | 0.530 | 0.008 | 290 | 372 |
| SCC | -0.013 | 0.090 | -0.189 | 0.164 | 0.890 | 1.000 | 0.006 | 290 | 372 |
| SCR | 0.000 | 0.105 | -0.206 | 0.206 | 1.000 | 1.000 | 22.566 | 290 | 372 |
| SFO | 0.116 | 0.090 | -0.061 | 0.294 | 0.197 | 0.547 | 0.007 | 290 | 372 |
| SLF | 0.140 | 0.122 | -0.099 | 0.379 | 0.251 | 0.572 | 40.776 | 290 | 372 |
| SS | 0.125 | 0.090 | -0.052 | 0.302 | 0.165 | 0.547 | 0.000 | 290 | 372 |
| UNC | 0.134 | 0.136 | -0.133 | 0.400 | 0.326 | 0.572 | 51.848 | 290 | 372 |
| AverageAD | 0.126 | 0.090 | -0.052 | 0.303 | 0.165 | 0.547 | 0.005 | 290 | 372 |

Table S10. Cohen’s d effect sizes after meta-analysis, for MD differences between patients and controls **in adolescents only**. Age, sex, agexsex, age^2^, age^2^xsex and scansite included as covariates.

| Region | Cohen’s d | SE | CI LB | CI UB | P-value | FDR P-value | I^2^ | Controls | Patients |
| --- | --- | --- | --- | --- | --- | --- | --- | --- | --- |
| ACR | 0.116 | 0.101 | -0.082 | 0.315 | 0.251 | 0.339 | 17.086 | 290 | 372 |
| ALIC | 0.149 | 0.090 | -0.028 | 0.326 | 0.099 | 0.288 | 0.003 | 290 | 372 |
| BCC | 0.179 | 0.090 | 0.003 | 0.356 | 0.047 | 0.235 | 0.000 | 290 | 372 |
| CC | 0.183 | 0.090 | 0.006 | 0.360 | 0.043 | 0.235 | 0.000 | 290 | 372 |
| CGC | 0.188 | 0.090 | 0.011 | 0.365 | 0.038 | 0.235 | 0.000 | 290 | 372 |
| CGH | -0.034 | 0.143 | -0.314 | 0.246 | 0.811 | 0.811 | 56.466 | 290 | 372 |
| CR | 0.145 | 0.098 | -0.048 | 0.338 | 0.140 | 0.288 | 12.856 | 290 | 372 |
| CST | -0.042 | 0.112 | -0.262 | 0.177 | 0.705 | 0.736 | 31.227 | 290 | 372 |
| EC | 0.048 | 0.129 | -0.204 | 0.301 | 0.707 | 0.736 | 46.763 | 290 | 372 |
| FX | 0.146 | 0.108 | -0.065 | 0.358 | 0.175 | 0.292 | 25.630 | 290 | 372 |
| FXST | 0.150 | 0.107 | -0.059 | 0.359 | 0.160 | 0.288 | 24.180 | 290 | 372 |
| GCC | 0.227 | 0.090 | 0.049 | 0.404 | 0.012 | 0.235 | 0.000 | 290 | 372 |
| IC | 0.092 | 0.090 | -0.085 | 0.269 | 0.309 | 0.386 | 0.000 | 290 | 372 |
| IFO | 0.215 | 0.107 | 0.005 | 0.425 | 0.044 | 0.235 | 24.527 | 290 | 372 |
| PCR | 0.168 | 0.097 | -0.023 | 0.359 | 0.084 | 0.288 | 11.216 | 290 | 372 |
| PLIC | -0.062 | 0.090 | -0.239 | 0.115 | 0.492 | 0.559 | 0.000 | 290 | 372 |
| PTR | 0.157 | 0.121 | -0.080 | 0.395 | 0.194 | 0.300 | 40.113 | 290 | 372 |
| RLIC | 0.145 | 0.098 | -0.048 | 0.337 | 0.141 | 0.288 | 12.613 | 290 | 372 |
| SCC | 0.088 | 0.090 | -0.089 | 0.265 | 0.328 | 0.390 | 0.000 | 290 | 372 |
| SCR | 0.114 | 0.101 | -0.083 | 0.311 | 0.258 | 0.339 | 16.273 | 290 | 372 |
| SFO | 0.136 | 0.097 | -0.054 | 0.326 | 0.161 | 0.288 | 10.933 | 290 | 372 |
| SLF | 0.180 | 0.096 | -0.008 | 0.367 | 0.061 | 0.254 | 8.582 | 290 | 372 |
| SS | 0.135 | 0.106 | -0.073 | 0.343 | 0.204 | 0.300 | 23.629 | 290 | 372 |
| UNC | 0.152 | 0.106 | -0.057 | 0.360 | 0.154 | 0.288 | 24.077 | 290 | 372 |
| AverageMD | 0.156 | 0.107 | -0.053 | 0.365 | 0.144 | 0.288 | 24.469 | 290 | 372 |

Table S11. Cohen’s d effect sizes after meta-analysis, for RD differences between patients and controls **in adolescents only**. Age, sex, agexsex, age^2^, age^2^xsex and scansite included as covariates.

| Region | Cohen’s d | SE | CI LB | CI UB | P-value | FDR P-value | I^2^ | Controls | Patients |
| --- | --- | --- | --- | --- | --- | --- | --- | --- | --- |
| ACR | 0.089 | 0.136 | -0.178 | 0.355 | 0.514 | 0.812 | 52.055 | 290 | 372 |
| ALIC | 0.036 | 0.150 | -0.257 | 0.329 | 0.808 | 0.873 | 60.163 | 290 | 372 |
| BCC | 0.209 | 0.090 | 0.032 | 0.386 | 0.020 | 0.250 | 0.000 | 290 | 372 |
| CC | 0.213 | 0.090 | 0.035 | 0.390 | 0.019 | 0.250 | 0.000 | 290 | 372 |
| CGC | 0.029 | 0.139 | -0.245 | 0.302 | 0.838 | 0.873 | 54.434 | 290 | 372 |
| CGH | -0.062 | 0.172 | -0.399 | 0.275 | 0.719 | 0.832 | 69.874 | 290 | 372 |
| CR | 0.098 | 0.131 | -0.159 | 0.355 | 0.456 | 0.812 | 48.693 | 290 | 372 |
| CST | 0.055 | 0.105 | -0.151 | 0.261 | 0.600 | 0.832 | 22.509 | 290 | 372 |
| EC | -0.053 | 0.154 | -0.354 | 0.249 | 0.732 | 0.832 | 62.377 | 290 | 372 |
| FX | 0.143 | 0.108 | -0.069 | 0.354 | 0.186 | 0.812 | 25.529 | 290 | 372 |
| FXST | 0.062 | 0.150 | -0.233 | 0.356 | 0.681 | 0.832 | 60.500 | 290 | 372 |
| GCC | 0.228 | 0.110 | 0.012 | 0.444 | 0.038 | 0.317 | 28.512 | 290 | 372 |
| IC | 0.016 | 0.109 | -0.198 | 0.231 | 0.880 | 0.880 | 27.611 | 290 | 372 |
| IFO | 0.162 | 0.145 | -0.121 | 0.446 | 0.262 | 0.812 | 57.429 | 290 | 372 |
| PCR | 0.111 | 0.101 | -0.087 | 0.310 | 0.272 | 0.812 | 17.317 | 290 | 372 |
| PLIC | -0.099 | 0.090 | -0.276 | 0.078 | 0.272 | 0.812 | 0.000 | 290 | 372 |
| PTR | 0.099 | 0.126 | -0.147 | 0.346 | 0.429 | 0.812 | 44.262 | 290 | 372 |
| RLIC | 0.061 | 0.103 | -0.140 | 0.263 | 0.552 | 0.812 | 19.498 | 290 | 372 |
| SCC | 0.073 | 0.094 | -0.111 | 0.256 | 0.438 | 0.812 | 5.877 | 290 | 372 |
| SCR | 0.094 | 0.130 | -0.161 | 0.349 | 0.470 | 0.812 | 47.825 | 290 | 372 |
| SFO | 0.052 | 0.143 | -0.228 | 0.332 | 0.716 | 0.832 | 56.368 | 290 | 372 |
| SLF | 0.093 | 0.114 | -0.129 | 0.316 | 0.411 | 0.812 | 32.349 | 290 | 372 |
| SS | 0.078 | 0.127 | -0.172 | 0.327 | 0.540 | 0.812 | 45.583 | 290 | 372 |
| UNC | 0.063 | 0.104 | -0.141 | 0.266 | 0.545 | 0.812 | 20.647 | 290 | 372 |
| AverageRD | 0.132 | 0.132 | -0.127 | 0.392 | 0.318 | 0.812 | 49.624 | 290 | 372 |

**2: Effect of MDD diagnosis: corrected for average diffusivity**

Table S12. Cohen’s d effect sizes after meta-analysis, for FA differences between **adult** patients and controls after correction for average FA. Age, sex, agexsex, age^2^, age^2^xsex, averageFA and scansite included as covariates.

| Region | Cohen’s d | SE | CI LB | CI UB | P-value | FDR P-value | I^2^ | Controls | Patients |
| --- | --- | --- | --- | --- | --- | --- | --- | --- | --- |
| ACR | -0.114 | 0.060 | -0.232 | 0.004 | 0.058 | 0.852 | 26.382 | 1265 | 921 |
| ALIC | -0.067 | 0.056 | -0.177 | 0.044 | 0.236 | 0.852 | 19.363 | 1265 | 921 |
| BCC | -0.075 | 0.064 | -0.201 | 0.051 | 0.245 | 0.852 | 33.437 | 1265 | 921 |
| CC | -0.064 | 0.076 | -0.214 | 0.086 | 0.406 | 0.852 | 51.733 | 1265 | 921 |
| CGC | -0.024 | 0.046 | -0.114 | 0.065 | 0.596 | 0.853 | 0.000 | 1265 | 921 |
| CGH | -0.009 | 0.046 | -0.099 | 0.080 | 0.838 | 0.860 | 0.001 | 1265 | 921 |
| CR | -0.077 | 0.064 | -0.203 | 0.049 | 0.229 | 0.852 | 33.641 | 1265 | 921 |
| CST | -0.014 | 0.046 | -0.104 | 0.075 | 0.758 | 0.855 | 0.002 | 1265 | 921 |
| EC | -0.064 | 0.069 | -0.198 | 0.071 | 0.354 | 0.852 | 40.781 | 1265 | 921 |
| FX | -0.048 | 0.050 | -0.146 | 0.049 | 0.333 | 0.852 | 7.102 | 1265 | 921 |
| FXST | -0.064 | 0.063 | -0.188 | 0.060 | 0.309 | 0.852 | 31.865 | 1265 | 921 |
| GCC | -0.073 | 0.086 | -0.242 | 0.095 | 0.394 | 0.852 | 62.052 | 1265 | 921 |
| IC | -0.051 | 0.063 | -0.175 | 0.074 | 0.426 | 0.852 | 32.241 | 1265 | 921 |
| IFO | -0.032 | 0.055 | -0.140 | 0.076 | 0.563 | 0.853 | 16.713 | 1265 | 921 |
| PCR | -0.030 | 0.059 | -0.144 | 0.085 | 0.613 | 0.853 | 23.274 | 1265 | 921 |
| PLIC | -0.012 | 0.066 | -0.140 | 0.117 | 0.860 | 0.860 | 35.923 | 1265 | 921 |
| PTR | 0.045 | 0.097 | -0.145 | 0.235 | 0.641 | 0.853 | 70.723 | 1265 | 921 |
| RLIC | 0.031 | 0.066 | -0.099 | 0.161 | 0.637 | 0.853 | 37.031 | 1265 | 921 |
| SCC | 0.072 | 0.046 | -0.018 | 0.161 | 0.115 | 0.852 | 0.005 | 1265 | 921 |
| SCR | -0.029 | 0.069 | -0.163 | 0.106 | 0.675 | 0.853 | 40.652 | 1265 | 921 |
| SFO | -0.086 | 0.048 | -0.181 | 0.008 | 0.073 | 0.852 | 4.206 | 1265 | 921 |
| SLF | 0.023 | 0.071 | -0.116 | 0.162 | 0.746 | 0.855 | 44.183 | 1265 | 921 |
| SS | -0.036 | 0.085 | -0.203 | 0.131 | 0.672 | 0.853 | 61.391 | 1265 | 921 |
| UNC | 0.024 | 0.086 | -0.145 | 0.193 | 0.784 | 0.855 | 62.381 | 1265 | 921 |

Table S13. Cohen’s d effect sizes after meta-analysis, for AD differences between **adult** patients and controls after correction for average AD. Age, sex, agexsex, age^2^, age^2^xsex, averageAD and scansite included as covariates.

| Region | Cohen’s d | SE | CI LB | CI UB | P-value | FDR P-value | I^2^ | Controls | Patients |
| --- | --- | --- | --- | --- | --- | --- | --- | --- | --- |
| ACR | -0.130 | 0.077 | -0.281 | 0.021 | 0.092 | 0.736 | 52.343 | 1265 | 921 |
| ALIC | 0.020 | 0.048 | -0.074 | 0.113 | 0.683 | 0.997 | 3.591 | 1265 | 921 |
| BCC | 0.011 | 0.072 | -0.129 | 0.152 | 0.875 | 0.997 | 45.241 | 1265 | 921 |
| CC | 0.010 | 0.073 | -0.133 | 0.153 | 0.889 | 0.997 | 47.168 | 1265 | 921 |
| CGC | -0.046 | 0.046 | -0.135 | 0.043 | 0.314 | 0.997 | 0.003 | 1265 | 921 |
| CGH | 0.015 | 0.056 | -0.095 | 0.124 | 0.794 | 0.997 | 18.192 | 1265 | 921 |
| CR | -0.012 | 0.052 | -0.114 | 0.089 | 0.812 | 0.997 | 10.717 | 1265 | 921 |
| CST | 0.080 | 0.055 | -0.027 | 0.187 | 0.143 | 0.858 | 15.718 | 1265 | 921 |
| EC | -0.024 | 0.072 | -0.165 | 0.118 | 0.743 | 0.997 | 46.152 | 1265 | 921 |
| FX | 0.060 | 0.046 | -0.030 | 0.149 | 0.192 | 0.922 | 0.001 | 1265 | 921 |
| FXST | 0.002 | 0.047 | -0.091 | 0.095 | 0.968 | 1.000 | 2.944 | 1265 | 921 |
| GCC | -0.050 | 0.046 | -0.139 | 0.040 | 0.275 | 0.997 | 0.001 | 1265 | 921 |
| IC | 0.025 | 0.060 | -0.093 | 0.143 | 0.679 | 0.997 | 26.319 | 1265 | 921 |
| IFO | 0.034 | 0.046 | -0.057 | 0.124 | 0.468 | 0.997 | 0.885 | 1265 | 921 |
| PCR | -0.019 | 0.072 | -0.160 | 0.121 | 0.787 | 0.997 | 45.410 | 1265 | 921 |
| PLIC | 0.107 | 0.054 | 0.000 | 0.214 | 0.049 | 0.588 | 15.598 | 1265 | 921 |
| PTR | 0.023 | 0.078 | -0.131 | 0.177 | 0.771 | 0.997 | 54.255 | 1265 | 921 |
| RLIC | -0.039 | 0.065 | -0.167 | 0.089 | 0.549 | 0.997 | 35.145 | 1265 | 921 |
| SCC | 0.065 | 0.075 | -0.083 | 0.212 | 0.389 | 0.997 | 50.243 | 1265 | 921 |
| SCR | 0.094 | 0.046 | 0.004 | 0.183 | 0.040 | 0.588 | 0.000 | 1265 | 921 |
| SFO | -0.010 | 0.075 | -0.158 | 0.138 | 0.894 | 0.997 | 50.171 | 1265 | 921 |
| SLF | 0.000 | 0.073 | -0.144 | 0.144 | 1.000 | 1.000 | 47.562 | 1265 | 921 |
| SS | -0.048 | 0.067 | -0.179 | 0.083 | 0.474 | 0.997 | 37.917 | 1265 | 921 |
| UNC | 0.011 | 0.097 | -0.180 | 0.201 | 0.914 | 0.997 | 70.789 | 1265 | 921 |

Table S14. Cohen’s d effect sizes after meta-analysis, for MD differences between **adult** patients and controls after correction for average MD. Age, sex, agexsex, age^2^, age^2^xsex, averageMD and scansite included as covariates.

| Region | Cohen’s d | SE | CI LB | CI UB | P-value | FDR P-value | I^2^ | Controls | Patients |
| --- | --- | --- | --- | --- | --- | --- | --- | --- | --- |
| ACR | -0.154 | 0.079 | -0.309 | 0.001 | 0.052 | 0.416 | 54.774 | 1265 | 921 |
| ALIC | -0.022 | 0.046 | -0.112 | 0.067 | 0.626 | 0.693 | 0.006 | 1265 | 921 |
| BCC | 0.107 | 0.063 | -0.016 | 0.230 | 0.087 | 0.522 | 30.716 | 1265 | 921 |
| CC | 0.044 | 0.046 | -0.045 | 0.134 | 0.330 | 0.602 | 0.003 | 1265 | 921 |
| CGC | -0.048 | 0.055 | -0.156 | 0.060 | 0.383 | 0.613 | 16.890 | 1265 | 921 |
| CGH | 0.026 | 0.053 | -0.078 | 0.129 | 0.628 | 0.693 | 12.583 | 1265 | 921 |
| CR | -0.071 | 0.068 | -0.204 | 0.062 | 0.295 | 0.602 | 39.490 | 1265 | 921 |
| CST | 0.043 | 0.058 | -0.070 | 0.157 | 0.453 | 0.665 | 22.064 | 1265 | 921 |
| EC | 0.034 | 0.062 | -0.088 | 0.155 | 0.588 | 0.693 | 29.819 | 1265 | 921 |
| FX | 0.033 | 0.046 | -0.057 | 0.124 | 0.471 | 0.665 | 1.170 | 1265 | 921 |
| FXST | 0.046 | 0.046 | -0.043 | 0.136 | 0.309 | 0.602 | 0.000 | 1265 | 921 |
| GCC | -0.069 | 0.058 | -0.183 | 0.045 | 0.234 | 0.602 | 22.452 | 1265 | 921 |
| IC | -0.088 | 0.046 | -0.178 | 0.001 | 0.052 | 0.416 | 0.001 | 1265 | 921 |
| IFO | -0.016 | 0.053 | -0.120 | 0.088 | 0.762 | 0.795 | 13.269 | 1265 | 921 |
| PCR | -0.001 | 0.060 | -0.119 | 0.117 | 0.993 | 0.993 | 26.279 | 1265 | 921 |
| PLIC | -0.061 | 0.051 | -0.161 | 0.039 | 0.230 | 0.602 | 9.436 | 1265 | 921 |
| PTR | -0.045 | 0.094 | -0.229 | 0.138 | 0.628 | 0.693 | 68.653 | 1265 | 921 |
| RLIC | -0.128 | 0.047 | -0.220 | -0.037 | 0.006 | 0.144 | 1.874 | 1265 | 921 |
| SCC | -0.049 | 0.052 | -0.151 | 0.054 | 0.351 | 0.602 | 11.484 | 1265 | 921 |
| SCR | 0.065 | 0.046 | -0.024 | 0.155 | 0.151 | 0.602 | 0.000 | 1265 | 921 |
| SFO | 0.053 | 0.046 | -0.036 | 0.143 | 0.246 | 0.602 | 0.002 | 1265 | 921 |
| SLF | -0.058 | 0.046 | -0.147 | 0.032 | 0.205 | 0.602 | 0.002 | 1265 | 921 |
| SS | -0.075 | 0.075 | -0.222 | 0.073 | 0.321 | 0.602 | 50.226 | 1265 | 921 |
| UNC | -0.043 | 0.091 | -0.222 | 0.135 | 0.635 | 0.693 | 66.457 | 1265 | 921 |

Table S15. Cohen’s d effect sizes after meta-analysis, for RD differences between **adult** patients and controls after correction for average RD. Age, sex, agexsex, age^2^, age^2^xsex, averageRD and scansite included as covariates.

| Region | Cohen’s d | SE | CI LB | CI UB | P-value | FDR P-value | I^2^ | Controls | Patients |
| --- | --- | --- | --- | --- | --- | --- | --- | --- | --- |
| ACR | -0.086 | 0.085 | -0.253 | 0.081 | 0.313 | 0.702 | 61.344 | 1265 | 921 |
| ALIC | 0.039 | 0.066 | -0.089 | 0.168 | 0.547 | 0.702 | 35.231 | 1265 | 921 |
| BCC | 0.089 | 0.050 | -0.009 | 0.187 | 0.075 | 0.702 | 7.124 | 1265 | 921 |
| CC | -0.001 | 0.083 | -0.164 | 0.163 | 0.995 | 0.995 | 59.551 | 1265 | 921 |
| CGC | 0.027 | 0.049 | -0.069 | 0.122 | 0.585 | 0.702 | 5.042 | 1265 | 921 |
| CGH | 0.062 | 0.052 | -0.041 | 0.164 | 0.239 | 0.702 | 11.759 | 1265 | 921 |
| CR | -0.034 | 0.076 | -0.183 | 0.115 | 0.653 | 0.746 | 51.283 | 1265 | 921 |
| CST | 0.042 | 0.057 | -0.069 | 0.154 | 0.459 | 0.702 | 20.180 | 1265 | 921 |
| EC | 0.058 | 0.072 | -0.082 | 0.199 | 0.416 | 0.702 | 45.321 | 1265 | 921 |
| FX | 0.069 | 0.065 | -0.059 | 0.197 | 0.293 | 0.702 | 35.401 | 1265 | 921 |
| FXST | 0.109 | 0.073 | -0.034 | 0.252 | 0.134 | 0.702 | 46.785 | 1265 | 921 |
| GCC | -0.059 | 0.090 | -0.235 | 0.116 | 0.509 | 0.702 | 65.378 | 1265 | 921 |
| IC | -0.056 | 0.085 | -0.221 | 0.110 | 0.510 | 0.702 | 60.698 | 1265 | 921 |
| IFO | -0.041 | 0.068 | -0.174 | 0.093 | 0.551 | 0.702 | 39.810 | 1265 | 921 |
| PCR | -0.017 | 0.085 | -0.184 | 0.149 | 0.837 | 0.913 | 61.076 | 1265 | 921 |
| PLIC | -0.085 | 0.092 | -0.265 | 0.095 | 0.353 | 0.702 | 67.057 | 1265 | 921 |
| PTR | -0.108 | 0.122 | -0.347 | 0.131 | 0.375 | 0.702 | 82.136 | 1265 | 921 |
| RLIC | -0.131 | 0.075 | -0.278 | 0.016 | 0.080 | 0.702 | 49.763 | 1265 | 921 |
| SCC | 0.001 | 0.098 | -0.192 | 0.194 | 0.992 | 0.995 | 71.558 | 1265 | 921 |
| SCR | 0.046 | 0.055 | -0.061 | 0.154 | 0.396 | 0.702 | 16.314 | 1265 | 921 |
| SFO | 0.091 | 0.074 | -0.054 | 0.236 | 0.218 | 0.702 | 48.664 | 1265 | 921 |
| SLF | -0.069 | 0.058 | -0.183 | 0.046 | 0.239 | 0.702 | 22.690 | 1265 | 921 |
| SS | -0.050 | 0.087 | -0.221 | 0.120 | 0.563 | 0.702 | 63.174 | 1265 | 921 |
| UNC | -0.093 | 0.085 | -0.259 | 0.073 | 0.272 | 0.702 | 60.784 | 1265 | 921 |

Table S16. Cohen’s d effect sizes after meta-analysis, for FA differences between **adolescent** patients and controls after correction for average FA. Age, sex, agexsex, age^2^, age^2^xsex, averageFA and scansite included as covariates.

| Region | Cohen’s d | SE | CI LB | CI UB | P-value | FDR P-value | I^2^ | Controls | Patients |
| --- | --- | --- | --- | --- | --- | --- | --- | --- | --- |
| ACR | -0.033 | 0.093 | -0.215 | 0.149 | 0.719 | 0.784 | 4.389 | 290 | 372 |
| ALIC | 0.055 | 0.105 | -0.150 | 0.260 | 0.598 | 0.784 | 21.558 | 290 | 372 |
| BCC | -0.211 | 0.110 | -0.427 | 0.004 | 0.055 | 0.252 | 28.262 | 290 | 372 |
| CC | -0.203 | 0.102 | -0.402 | -0.003 | 0.047 | 0.252 | 17.894 | 290 | 372 |
| CGC | 0.091 | 0.166 | -0.235 | 0.418 | 0.583 | 0.784 | 67.865 | 290 | 372 |
| CGH | 0.097 | 0.100 | -0.100 | 0.294 | 0.334 | 0.784 | 15.891 | 290 | 372 |
| CR | -0.033 | 0.090 | -0.210 | 0.143 | 0.713 | 0.784 | 0.000 | 290 | 372 |
| CST | -0.046 | 0.121 | -0.284 | 0.192 | 0.705 | 0.784 | 40.360 | 290 | 372 |
| EC | 0.342 | 0.135 | 0.078 | 0.607 | 0.011 | 0.088 | 50.840 | 290 | 372 |
| FX | 0.055 | 0.090 | -0.123 | 0.232 | 0.545 | 0.784 | 0.001 | 290 | 372 |
| FXST | 0.032 | 0.144 | -0.250 | 0.314 | 0.825 | 0.825 | 57.014 | 290 | 372 |
| GCC | -0.168 | 0.090 | -0.346 | 0.009 | 0.063 | 0.252 | 0.000 | 290 | 372 |
| IC | 0.247 | 0.092 | 0.066 | 0.428 | 0.007 | 0.084 | 3.011 | 290 | 372 |
| IFO | -0.051 | 0.118 | -0.282 | 0.180 | 0.665 | 0.784 | 36.799 | 290 | 372 |
| PCR | 0.044 | 0.117 | -0.186 | 0.274 | 0.711 | 0.784 | 36.325 | 290 | 372 |
| PLIC | 0.254 | 0.091 | 0.076 | 0.431 | 0.005 | 0.084 | 0.000 | 290 | 372 |
| PTR | 0.103 | 0.108 | -0.109 | 0.314 | 0.341 | 0.784 | 25.778 | 290 | 372 |
| RLIC | 0.137 | 0.115 | -0.089 | 0.363 | 0.233 | 0.784 | 34.375 | 290 | 372 |
| SCC | 0.027 | 0.113 | -0.195 | 0.249 | 0.812 | 0.825 | 32.430 | 290 | 372 |
| SCR | -0.043 | 0.090 | -0.219 | 0.134 | 0.637 | 0.784 | 0.000 | 290 | 372 |
| SFO | -0.039 | 0.090 | -0.216 | 0.138 | 0.664 | 0.784 | 0.000 | 290 | 372 |
| SLF | 0.102 | 0.101 | -0.096 | 0.301 | 0.312 | 0.784 | 17.059 | 290 | 372 |
| SS | 0.069 | 0.140 | -0.204 | 0.343 | 0.619 | 0.784 | 54.654 | 290 | 372 |
| UNC | 0.082 | 0.146 | -0.205 | 0.368 | 0.577 | 0.784 | 58.310 | 290 | 372 |

Table S17. Cohen’s d effect sizes after meta-analysis, for AD differences between **adolescent** patients and controls after correction for average AD. Age, sex, agexsex, age^2^, age^2^xsex, averageAD and scansite included as covariates.

| Region | Cohen’s d | SE | CI LB | CI UB | P-value | FDR P-value | I^2^ | Controls | Patients |
| --- | --- | --- | --- | --- | --- | --- | --- | --- | --- |
| ACR | 0.032 | 0.090 | -0.144 | 0.209 | 0.720 | 0.916 | 0.000 | 290 | 372 |
| ALIC | 0.107 | 0.106 | -0.100 | 0.313 | 0.313 | 0.770 | 22.930 | 290 | 372 |
| BCC | -0.151 | 0.090 | -0.328 | 0.026 | 0.095 | 0.770 | 0.000 | 290 | 372 |
| CC | -0.098 | 0.090 | -0.275 | 0.079 | 0.278 | 0.770 | 0.001 | 290 | 372 |
| CGC | 0.158 | 0.126 | -0.088 | 0.404 | 0.209 | 0.770 | 44.001 | 290 | 372 |
| CGH | -0.029 | 0.108 | -0.241 | 0.184 | 0.792 | 0.943 | 26.762 | 290 | 372 |
| CR | 0.006 | 0.095 | -0.179 | 0.191 | 0.948 | 0.952 | 7.088 | 290 | 372 |
| CST | -0.134 | 0.124 | -0.378 | 0.110 | 0.282 | 0.770 | 43.050 | 290 | 372 |
| EC | 0.123 | 0.110 | -0.094 | 0.339 | 0.267 | 0.770 | 29.124 | 290 | 372 |
| FX | 0.011 | 0.115 | -0.213 | 0.236 | 0.920 | 0.952 | 33.183 | 290 | 372 |
| FXST | 0.101 | 0.092 | -0.079 | 0.281 | 0.272 | 0.770 | 2.693 | 290 | 372 |
| GCC | 0.054 | 0.090 | -0.123 | 0.230 | 0.552 | 0.828 | 0.000 | 290 | 372 |
| IC | 0.039 | 0.090 | -0.138 | 0.216 | 0.669 | 0.916 | 0.000 | 290 | 372 |
| IFO | 0.032 | 0.090 | -0.145 | 0.209 | 0.725 | 0.916 | 0.000 | 290 | 372 |
| PCR | 0.010 | 0.166 | -0.315 | 0.335 | 0.952 | 0.952 | 67.710 | 290 | 372 |
| PLIC | -0.092 | 0.099 | -0.285 | 0.102 | 0.353 | 0.770 | 13.363 | 290 | 372 |
| PTR | 0.107 | 0.110 | -0.109 | 0.323 | 0.332 | 0.770 | 28.481 | 290 | 372 |
| RLIC | 0.060 | 0.090 | -0.117 | 0.237 | 0.505 | 0.808 | 0.000 | 290 | 372 |
| SCC | -0.109 | 0.090 | -0.285 | 0.068 | 0.229 | 0.770 | 0.000 | 290 | 372 |
| SCR | -0.092 | 0.095 | -0.279 | 0.095 | 0.336 | 0.770 | 8.440 | 290 | 372 |
| SFO | 0.020 | 0.090 | -0.157 | 0.197 | 0.825 | 0.943 | 0.000 | 290 | 372 |
| SLF | 0.096 | 0.127 | -0.152 | 0.344 | 0.448 | 0.778 | 45.002 | 290 | 372 |
| SS | 0.068 | 0.090 | -0.109 | 0.244 | 0.454 | 0.778 | 0.001 | 290 | 372 |
| UNC | 0.096 | 0.125 | -0.148 | 0.341 | 0.439 | 0.778 | 43.161 | 290 | 372 |

Table S18. Cohen’s d effect sizes after meta-analysis, for MD differences between **adolescent** patients and controls after correction for average MD. Age, sex, agexsex, age^2^, age^2^xsex, averageMD and scansite included as covariates.

| Region | Cohen’s d | SE | CI LB | CI UB | P-value | FDR P-value | I^2^ | Controls | Patients |
| --- | --- | --- | --- | --- | --- | --- | --- | --- | --- |
| ACR | 0.041 | 0.090 | -0.136 | 0.218 | 0.648 | 0.819 | 0.007 | 290 | 372 |
| ALIC | 0.012 | 0.103 | -0.191 | 0.215 | 0.907 | 0.978 | 20.143 | 290 | 372 |
| BCC | 0.088 | 0.090 | -0.089 | 0.264 | 0.331 | 0.809 | 0.000 | 290 | 372 |
| CC | 0.099 | 0.090 | -0.078 | 0.276 | 0.272 | 0.809 | 0.000 | 290 | 372 |
| CGC | 0.092 | 0.090 | -0.084 | 0.269 | 0.306 | 0.809 | 0.000 | 290 | 372 |
| CGH | -0.128 | 0.134 | -0.391 | 0.134 | 0.337 | 0.809 | 50.524 | 290 | 372 |
| CR | 0.050 | 0.090 | -0.127 | 0.227 | 0.583 | 0.819 | 0.001 | 290 | 372 |
| CST | -0.090 | 0.112 | -0.310 | 0.129 | 0.420 | 0.809 | 31.132 | 290 | 372 |
| EC | -0.103 | 0.132 | -0.361 | 0.156 | 0.437 | 0.809 | 48.999 | 290 | 372 |
| FX | 0.067 | 0.097 | -0.123 | 0.257 | 0.489 | 0.819 | 10.426 | 290 | 372 |
| FXST | 0.012 | 0.147 | -0.276 | 0.299 | 0.937 | 0.978 | 58.586 | 290 | 372 |
| GCC | 0.163 | 0.090 | -0.014 | 0.340 | 0.070 | 0.809 | 0.000 | 290 | 372 |
| IC | -0.086 | 0.096 | -0.275 | 0.104 | 0.375 | 0.809 | 10.119 | 290 | 372 |
| IFO | 0.103 | 0.117 | -0.126 | 0.332 | 0.378 | 0.809 | 35.619 | 290 | 372 |
| PCR | 0.070 | 0.090 | -0.107 | 0.247 | 0.438 | 0.809 | 0.000 | 290 | 372 |
| PLIC | -0.216 | 0.098 | -0.408 | -0.024 | 0.028 | 0.672 | 12.092 | 290 | 372 |
| PTR | 0.092 | 0.090 | -0.085 | 0.269 | 0.308 | 0.809 | 0.000 | 290 | 372 |
| RLIC | -0.009 | 0.090 | -0.186 | 0.167 | 0.917 | 0.978 | 0.007 | 290 | 372 |
| SCC | -0.044 | 0.090 | -0.220 | 0.133 | 0.628 | 0.819 | 0.000 | 290 | 372 |
| SCR | 0.001 | 0.090 | -0.175 | 0.178 | 0.988 | 0.988 | 0.000 | 290 | 372 |
| SFO | 0.055 | 0.094 | -0.130 | 0.240 | 0.561 | 0.819 | 6.772 | 290 | 372 |
| SLF | 0.034 | 0.147 | -0.255 | 0.323 | 0.819 | 0.978 | 58.868 | 290 | 372 |
| SS | 0.051 | 0.091 | -0.126 | 0.229 | 0.570 | 0.819 | 0.003 | 290 | 372 |
| UNC | 0.083 | 0.090 | -0.094 | 0.260 | 0.359 | 0.809 | 0.000 | 290 | 372 |

Table S19. Cohen’s d effect sizes after meta-analysis, for RD differences between **adolescent** patients and controls after correction for average RD. Age, sex, agexsex, age^2^, age^2^xsex, averageRD and scansite included as covariates.

| Region | Cohen’s d | SE | CI LB | CI UB | P-value | FDR P-value | I^2^ | Controls | Patients |
| --- | --- | --- | --- | --- | --- | --- | --- | --- | --- |
| ACR | 0.045 | 0.090 | -0.132 | 0.222 | 0.619 | 0.961 | 0.000 | 290 | 372 |
| ALIC | -0.040 | 0.146 | -0.325 | 0.246 | 0.785 | 0.961 | 57.813 | 290 | 372 |
| BCC | 0.156 | 0.090 | -0.021 | 0.333 | 0.084 | 0.504 | 0.000 | 290 | 372 |
| CC | 0.171 | 0.090 | -0.006 | 0.348 | 0.058 | 0.464 | 0.000 | 290 | 372 |
| CGC | -0.031 | 0.164 | -0.353 | 0.291 | 0.850 | 0.961 | 67.020 | 290 | 372 |
| CGH | -0.119 | 0.166 | -0.444 | 0.205 | 0.471 | 0.961 | 67.518 | 290 | 372 |
| CR | 0.021 | 0.090 | -0.156 | 0.198 | 0.813 | 0.961 | 0.000 | 290 | 372 |
| CST | 0.005 | 0.097 | -0.185 | 0.194 | 0.961 | 0.961 | 10.513 | 290 | 372 |
| EC | -0.207 | 0.155 | -0.509 | 0.096 | 0.181 | 0.776 | 62.354 | 290 | 372 |
| FX | 0.083 | 0.099 | -0.112 | 0.278 | 0.405 | 0.961 | 14.121 | 290 | 372 |
| FXST | -0.021 | 0.184 | -0.382 | 0.340 | 0.910 | 0.961 | 73.743 | 290 | 372 |
| GCC | 0.195 | 0.091 | 0.018 | 0.372 | 0.031 | 0.372 | 0.000 | 290 | 372 |
| IC | -0.132 | 0.102 | -0.331 | 0.067 | 0.194 | 0.776 | 17.473 | 290 | 372 |
| IFO | 0.102 | 0.134 | -0.161 | 0.365 | 0.447 | 0.961 | 50.694 | 290 | 372 |
| PCR | -0.014 | 0.090 | -0.191 | 0.162 | 0.873 | 0.961 | 0.000 | 290 | 372 |
| PLIC | -0.223 | 0.090 | -0.401 | -0.046 | 0.014 | 0.336 | 0.000 | 290 | 372 |
| PTR | 0.015 | 0.124 | -0.228 | 0.258 | 0.902 | 0.961 | 42.870 | 290 | 372 |
| RLIC | -0.067 | 0.116 | -0.294 | 0.160 | 0.561 | 0.961 | 34.891 | 290 | 372 |
| SCC | -0.027 | 0.090 | -0.203 | 0.150 | 0.769 | 0.961 | 0.000 | 290 | 372 |
| SCR | 0.020 | 0.090 | -0.156 | 0.197 | 0.821 | 0.961 | 0.000 | 290 | 372 |
| SFO | 0.048 | 0.101 | -0.151 | 0.246 | 0.639 | 0.961 | 17.166 | 290 | 372 |
| SLF | -0.047 | 0.112 | -0.267 | 0.173 | 0.675 | 0.961 | 30.889 | 290 | 372 |
| SS | 0.009 | 0.151 | -0.288 | 0.305 | 0.954 | 0.961 | 61.094 | 290 | 372 |
| UNC | 0.023 | 0.090 | -0.154 | 0.199 | 0.803 | 0.961 | 0.001 | 290 | 372 |

**3: Diagnosis-by-sex interaction effect**

Table S20. Full meta-analytic results for the Diagnosis by Sex interaction for FA **in adults only**. Age, sex, age^2^ and scansite included as covariates.

| Region | Beta | SE | CI LB | CI UB | P-value | FDR P-value | I^2^ | Controls | Patients |
| --- | --- | --- | --- | --- | --- | --- | --- | --- | --- |
| ACR | -0.00069658 | 0.00151024 | -0.0036566 | 0.00226343 | 0.645 | 0.790 | 0.000 | 1256 | 924 |
| ALIC | -0.00168227 | 0.00265693 | -0.00688976 | 0.00352522 | 0.527 | 0.730 | 24.221 | 1256 | 924 |
| BCC | -0.00183887 | 0.00193394 | -0.00562931 | 0.00195157 | 0.342 | 0.658 | 0.943 | 1256 | 924 |
| CC | -0.00296819 | 0.00203949 | -0.00696551 | 0.00102913 | 0.146 | 0.484 | 9.689 | 1256 | 924 |
| CGC | -0.00581972 | 0.00408895 | -0.01383391 | 0.00219447 | 0.155 | 0.484 | 35.741 | 1256 | 924 |
| CGH | -0.0082493 | 0.00490953 | -0.01787181 | 0.00137321 | 0.093 | 0.484 | 40.850 | 1256 | 924 |
| CR | -0.00049927 | 0.00124501 | -0.00293944 | 0.0019409 | 0.688 | 0.790 | 0.173 | 1256 | 924 |
| CST | 0.00382401 | 0.00460012 | -0.00519205 | 0.01284008 | 0.406 | 0.679 | 18.029 | 1256 | 924 |
| EC | -0.00166683 | 0.00160738 | -0.00481724 | 0.00148358 | 0.300 | 0.625 | 8.012 | 1256 | 924 |
| FX | -0.00446325 | 0.00595386 | -0.0161326 | 0.00720609 | 0.453 | 0.679 | 23.454 | 1256 | 924 |
| FXST | -0.00468043 | 0.00347291 | -0.01148721 | 0.00212636 | 0.178 | 0.494 | 30.656 | 1256 | 924 |
| GCC | -0.00316487 | 0.00206616 | -0.00721447 | 0.00088473 | 0.126 | 0.484 | 5.109 | 1256 | 924 |
| IC | -0.00091611 | 0.00233632 | -0.0054952 | 0.00366299 | 0.695 | 0.790 | 28.573 | 1256 | 924 |
| IFO | -0.00946824 | 0.00416296 | -0.01762749 | -0.00130899 | 0.023 | 0.288 | 16.001 | 1256 | 924 |
| PCR | 0.0004875 | 0.00149731 | -0.00244717 | 0.00342217 | 0.745 | 0.810 | 0.000 | 1256 | 924 |
| PLIC | 0.00024411 | 0.00335434 | -0.00633029 | 0.0068185 | 0.942 | 0.942 | 49.750 | 1256 | 924 |
| PTR | -0.0028936 | 0.00117628 | -0.00519906 | -0.00058815 | 0.014 | 0.288 | 0.000 | 1256 | 924 |
| RLIC | -0.00169756 | 0.00143285 | -0.0045059 | 0.00111078 | 0.236 | 0.564 | 0.000 | 1256 | 924 |
| SCC | -0.00288931 | 0.00185139 | -0.00651798 | 0.00073936 | 0.119 | 0.484 | 11.689 | 1256 | 924 |
| SCR | -0.00211667 | 0.00287623 | -0.00775397 | 0.00352064 | 0.462 | 0.679 | 40.398 | 1256 | 924 |
| SFO | 0.00024252 | 0.00218849 | -0.00404684 | 0.00453189 | 0.912 | 0.942 | 0.000 | 1256 | 924 |
| SLF | -0.00166262 | 0.00144025 | -0.00448546 | 0.00116022 | 0.248 | 0.564 | 0.000 | 1256 | 924 |
| SS | 0.00100283 | 0.0013002 | -0.00154551 | 0.00355117 | 0.441 | 0.679 | 0.000 | 1256 | 924 |
| UNC | -0.00215918 | 0.0036606 | -0.00933382 | 0.00501545 | 0.555 | 0.730 | 1.258 | 1256 | 924 |
| AverageFA | -0.00152557 | 0.00102541 | -0.00353534 | 0.0004842 | 0.137 | 0.484 | 4.684 | 1256 | 924 |

Table S21. Full meta-analytic results for the Diagnosis by Sex interaction for AD **in adults only**. Age, sex, age^2^ and scansite included as covariates.

| Region | Beta | SE | CI LB | CI UB | P-value | FDR P-value | I^2^ | Controls | Patients |
| --- | --- | --- | --- | --- | --- | --- | --- | --- | --- |
| ACR | 0.00001073 | 0.000099 | -0.00018331 | 0.00020477 | 0.914 | 1.000 | 99.753 | 1256 | 924 |
| ALIC | 0.00000439 | 0.00000419 | -0.00000382 | 0.0000126 | 0.295 | 1.000 | 0.000 | 1256 | 924 |
| BCC | -0.00000959 | 0.00000579 | -0.00002095 | 0.00000176 | 0.098 | 1.000 | 8.442 | 1256 | 924 |
| CC | -0.00000626 | 0.0000055 | -0.00001704 | 0.00000453 | 0.256 | 1.000 | 15.631 | 1256 | 924 |
| CGC | -0.00000634 | 0.00000637 | -0.00001882 | 0.00000614 | 0.319 | 1.000 | 7.801 | 1256 | 924 |
| CGH | -0.00004009 | 0.00076345 | -0.00153644 | 0.00145625 | 0.958 | 1.000 | 99.991 | 1256 | 924 |
| CR | 0.00000214 | 0.00000393 | -0.00000557 | 0.00000984 | 0.586 | 1.000 | 3.638 | 1256 | 924 |
| CST | 0.00001089 | 0.00000683 | -0.00000249 | 0.00002428 | 0.111 | 1.000 | 0.000 | 1256 | 924 |
| EC | -0.00001033 | 0.00022021 | -0.00044193 | 0.00042126 | 0.963 | 1.000 | 99.972 | 1256 | 924 |
| FX | 0.00002894 | 0.00002481 | -0.0000197 | 0.00007757 | 0.244 | 1.000 | 7.744 | 1256 | 924 |
| FXST | 0.00002634 | 0.00056637 | -0.00108373 | 0.0011364 | 0.963 | 1.000 | 99.990 | 1256 | 924 |
| GCC | -0.00000379 | 0.00000552 | -0.00001461 | 0.00000703 | 0.493 | 1.000 | 0.000 | 1256 | 924 |
| IC | 0.00000109 | 0.00000375 | -0.00000626 | 0.00000845 | 0.770 | 1.000 | 0.000 | 1256 | 924 |
| IFO | -0.00006899 | 0.00061201 | -0.00126851 | 0.00113054 | 0.910 | 1.000 | 99.989 | 1256 | 924 |
| PCR | 0.00000237 | 0.00000431 | -0.00000607 | 0.00001081 | 0.582 | 1.000 | 0.745 | 1256 | 924 |
| PLIC | 0.0000005 | 0.0000051 | -0.00000949 | 0.0000105 | 0.921 | 1.000 | 0.000 | 1256 | 924 |
| PTR | -0.0000047 | 0.00000479 | -0.00001408 | 0.00000468 | 0.326 | 1.000 | 0.000 | 1256 | 924 |
| RLIC | -0.00000112 | 0.00000513 | -0.00001118 | 0.00000894 | 0.827 | 1.000 | 5.919 | 1256 | 924 |
| SCC | -0.00000598 | 0.00000752 | -0.00002071 | 0.00000875 | 0.426 | 1.000 | 26.567 | 1256 | 924 |
| SCR | 0.00000136 | 0.00000437 | -0.00000721 | 0.00000993 | 0.756 | 1.000 | 4.320 | 1256 | 924 |
| SFO | 0.00000002 | 0.0002554 | -0.00050055 | 0.00050059 | 1.000 | 1.000 | 99.952 | 1256 | 924 |
| SLF | -0.00000493 | 0.00000349 | -0.00001177 | 0.00000191 | 0.158 | 1.000 | 0.026 | 1256 | 924 |
| SS | 0.00000984 | 0.0000691 | -0.00012559 | 0.00014526 | 0.887 | 1.000 | 99.441 | 1256 | 924 |
| UNC | 0.00000322 | 0.00000867 | -0.00001378 | 0.00002021 | 0.711 | 1.000 | 2.444 | 1256 | 924 |
| AverageAD | -0.00000067 | 0.00000309 | -0.00000673 | 0.00000538 | 0.827 | 1.000 | 18.104 | 1256 | 924 |

Table S22. Full meta-analytic results for the Diagnosis by Sex interaction for MD **in adults only**. Age, sex, age^2^ and scansite included as covariates.

| Region | Beta | SE | CI LB | CI UB | P-value | FDR P-value | I^2^ | Controls | Patients |
| --- | --- | --- | --- | --- | --- | --- | --- | --- | --- |
| ACR | 0.00000813 | 0.00009829 | -0.00018452 | 0.00020078 | 0.934 | 0.997 | 99.865 | 1256 | 924 |
| ALIC | 0.00000156 | 0.00000274 | -0.00000381 | 0.00000693 | 0.568 | 0.997 | 5.612 | 1256 | 924 |
| BCC | -0.00000063 | 0.00000399 | -0.00000845 | 0.00000719 | 0.875 | 0.997 | 0.000 | 1256 | 924 |
| CC | 0.00000044 | 0.00000375 | -0.00000692 | 0.00000779 | 0.907 | 0.997 | 7.517 | 1256 | 924 |
| CGC | 0.00000074 | 0.00000309 | -0.00000531 | 0.0000068 | 0.810 | 0.997 | 0.000 | 1256 | 924 |
| CGH | -0.00003364 | 0.0007638 | -0.00153067 | 0.00146338 | 0.965 | 0.997 | 99.997 | 1256 | 924 |
| CR | 0.0000009 | 0.00000306 | -0.00000509 | 0.00000689 | 0.768 | 0.997 | 7.887 | 1256 | 924 |
| CST | 0.0000038 | 0.00000434 | -0.00000471 | 0.00001231 | 0.381 | 0.997 | 0.000 | 1256 | 924 |
| EC | -0.00000526 | 0.00022067 | -0.00043776 | 0.00042723 | 0.981 | 0.997 | 99.985 | 1256 | 924 |
| FX | 0.00003052 | 0.00003038 | -0.00002902 | 0.00009005 | 0.315 | 0.997 | 30.103 | 1256 | 924 |
| FXST | 0.00003227 | 0.0005659 | -0.00107687 | 0.00114142 | 0.955 | 0.997 | 99.997 | 1256 | 924 |
| GCC | 0.00000283 | 0.00000436 | -0.00000572 | 0.00001138 | 0.517 | 0.997 | 5.038 | 1256 | 924 |
| IC | -0.00000065 | 0.00000259 | -0.00000573 | 0.00000443 | 0.802 | 0.997 | 11.470 | 1256 | 924 |
| IFO | -0.00005219 | 0.00061228 | -0.00125224 | 0.00114786 | 0.932 | 0.997 | 99.997 | 1256 | 924 |
| PCR | 0.00000001 | 0.00000301 | -0.00000589 | 0.00000592 | 0.997 | 0.997 | 0.000 | 1256 | 924 |
| PLIC | -0.00000106 | 0.00000312 | -0.00000718 | 0.00000507 | 0.735 | 0.997 | 15.227 | 1256 | 924 |
| PTR | 0.00000045 | 0.00000343 | -0.00000627 | 0.00000718 | 0.895 | 0.997 | 3.086 | 1256 | 924 |
| RLIC | -0.000002 | 0.00000289 | -0.00000767 | 0.00000367 | 0.489 | 0.997 | 5.685 | 1256 | 924 |
| SCC | -0.00000123 | 0.00000359 | -0.00000827 | 0.0000058 | 0.731 | 0.997 | 11.518 | 1256 | 924 |
| SCR | 0.00000032 | 0.00000273 | -0.00000504 | 0.00000567 | 0.908 | 0.997 | 9.946 | 1256 | 924 |
| SFO | -0.00000174 | 0.00025521 | -0.00050193 | 0.00049846 | 0.995 | 0.997 | 99.982 | 1256 | 924 |
| SLF | -0.00000055 | 0.00000249 | -0.00000543 | 0.00000434 | 0.826 | 0.997 | 2.958 | 1256 | 924 |
| SS | 0.0000054 | 0.00007059 | -0.00013295 | 0.00014375 | 0.939 | 0.997 | 99.766 | 1256 | 924 |
| UNC | 0.00000188 | 0.00000554 | -0.00000899 | 0.00001274 | 0.735 | 0.997 | 0.000 | 1256 | 924 |
| AverageMD | 0.00000194 | 0.00000308 | -0.0000041 | 0.00000799 | 0.529 | 0.997 | 24.336 | 1256 | 924 |

Table S23. Full meta-analytic results for the Diagnosis by Sex interaction for RD **in adults only**. Age, sex, age^2^ and scansite included as covariates.

| Region | Beta | SE | CI LB | CI UB | P-value | FDR P-value | I^2^ | Controls | Patients |
| --- | --- | --- | --- | --- | --- | --- | --- | --- | --- |
| ACR | 0.00000734 | 0.00009815 | -0.00018503 | 0.00019971 | 0.940 | 0.990 | 99.848 | 1241 | 910 |
| ALIC | 0.00000254 | 0.00000348 | -0.00000427 | 0.00000936 | 0.464 | 0.990 | 19.992 | 1241 | 910 |
| BCC | 0.00000353 | 0.00000475 | -0.00000577 | 0.00001284 | 0.457 | 0.990 | 0.000 | 1241 | 910 |
| CC | 0.00000514 | 0.0000039 | -0.00000251 | 0.00001278 | 0.188 | 0.990 | 5.872 | 1241 | 910 |
| CGC | 0.00000728 | 0.00000406 | -0.00000068 | 0.00001524 | 0.073 | 0.990 | 7.538 | 1241 | 910 |
| CGH | -0.00003042 | 0.00076401 | -0.00152785 | 0.00146701 | 0.968 | 0.990 | 99.996 | 1241 | 910 |
| CR | 0.000002 | 0.00000349 | -0.00000484 | 0.00000884 | 0.567 | 0.990 | 13.047 | 1241 | 910 |
| CST | -0.00000051 | 0.00000461 | -0.00000955 | 0.00000852 | 0.911 | 0.990 | 0.000 | 1241 | 910 |
| EC | -0.00000286 | 0.0002209 | -0.00043582 | 0.0004301 | 0.990 | 0.990 | 99.981 | 1241 | 910 |
| FX | 0.00003356 | 0.00003296 | -0.00003105 | 0.00009817 | 0.309 | 0.990 | 36.326 | 1241 | 910 |
| FXST | 0.00003553 | 0.00056568 | -0.00107319 | 0.00114425 | 0.950 | 0.990 | 99.996 | 1241 | 910 |
| GCC | 0.00000325 | 0.000004 | -0.00000459 | 0.0000111 | 0.416 | 0.990 | 0.000 | 1241 | 910 |
| IC | -0.00000063 | 0.00000289 | -0.0000063 | 0.00000504 | 0.828 | 0.990 | 16.067 | 1241 | 910 |
| IFO | -0.00004441 | 0.00061244 | -0.00124477 | 0.00115595 | 0.942 | 0.990 | 99.995 | 1241 | 910 |
| PCR | -0.00000035 | 0.00000335 | -0.00000692 | 0.00000622 | 0.917 | 0.990 | 0.000 | 1241 | 910 |
| PLIC | -0.00000233 | 0.00000311 | -0.00000843 | 0.00000377 | 0.454 | 0.990 | 13.542 | 1241 | 910 |
| PTR | 0.00000161 | 0.00000358 | -0.0000054 | 0.00000863 | 0.652 | 0.990 | 0.000 | 1241 | 910 |
| RLIC | 0.00000077 | 0.00000347 | -0.00000603 | 0.00000756 | 0.825 | 0.990 | 9.693 | 1241 | 910 |
| SCC | 0.00000696 | 0.00000412 | -0.00000112 | 0.00001503 | 0.092 | 0.990 | 21.862 | 1241 | 910 |
| SCR | 0.00000077 | 0.00000305 | -0.0000052 | 0.00000674 | 0.801 | 0.990 | 12.290 | 1241 | 910 |
| SFO | -0.00000344 | 0.00025502 | -0.00050328 | 0.00049639 | 0.989 | 0.990 | 99.979 | 1241 | 910 |
| SLF | 0.00000104 | 0.00000267 | -0.00000419 | 0.00000627 | 0.696 | 0.990 | 0.000 | 1241 | 910 |
| SS | 0.00000363 | 0.00007127 | -0.00013606 | 0.00014332 | 0.959 | 0.990 | 99.718 | 1241 | 910 |
| UNC | 0.00000046 | 0.00000643 | -0.00001214 | 0.00001306 | 0.943 | 0.990 | 0.000 | 1241 | 910 |
| AverageRD | 0.000004 | 0.00000342 | -0.00000271 | 0.00001071 | 0.242 | 0.990 | 28.665 | 1241 | 910 |

Table S24. Full meta-analytic results for the Diagnosis by Sex interaction for FA **in adolescents only**. Age, sex, age^2^ and scansite included as covariates.

| Region | Beta | SE | CI LB | CI UB | P-value | FDR P-value | I^2^ | Controls | Patients |
| --- | --- | --- | --- | --- | --- | --- | --- | --- | --- |
| ACR | -0.00734714 | 0.00665401 | -0.02038875 | 0.00569447 | 0.270 | 0.731 | 36.490 | 323 | 379 |
| ALIC | 0.00044967 | 0.00570351 | -0.010729 | 0.01162833 | 0.937 | 0.937 | 28.834 | 323 | 379 |
| BCC | 0.00815565 | 0.00803948 | -0.00760144 | 0.02391275 | 0.310 | 0.731 | 26.245 | 323 | 379 |
| CC | 0.00518115 | 0.00542613 | -0.00545387 | 0.01581617 | 0.340 | 0.731 | 14.546 | 323 | 379 |
| CGC | 0.00067412 | 0.00660594 | -0.01227329 | 0.01362153 | 0.919 | 0.937 | 0.288 | 323 | 379 |
| CGH | 0.00157859 | 0.0076105 | -0.01333771 | 0.01649489 | 0.836 | 0.937 | 0.000 | 323 | 379 |
| CR | 0.00438538 | 0.00473009 | -0.00488544 | 0.01365619 | 0.354 | 0.731 | 18.120 | 323 | 379 |
| CST | -0.00315399 | 0.00761594 | -0.01808095 | 0.01177298 | 0.679 | 0.893 | 0.000 | 323 | 379 |
| EC | 0.00419467 | 0.00462169 | -0.00486367 | 0.013253 | 0.364 | 0.731 | 17.567 | 323 | 379 |
| FX | -0.00461992 | 0.00919794 | -0.02264755 | 0.01340772 | 0.615 | 0.854 | 0.000 | 323 | 379 |
| FXST | 0.00346457 | 0.00631487 | -0.00891235 | 0.0158415 | 0.583 | 0.854 | 0.000 | 323 | 379 |
| GCC | 0.00047059 | 0.00588688 | -0.01106749 | 0.01200866 | 0.936 | 0.937 | 9.746 | 323 | 379 |
| IC | 0.00410327 | 0.00467002 | -0.0050498 | 0.01325634 | 0.380 | 0.731 | 23.279 | 323 | 379 |
| IFO | 0.00097188 | 0.0075967 | -0.01391739 | 0.01586115 | 0.898 | 0.937 | 0.000 | 323 | 379 |
| PCR | 0.01564171 | 0.00518969 | 0.00547009 | 0.02581332 | 0.003 | 0.075 | 0.000 | 323 | 379 |
| PLIC | 0.00626673 | 0.00585022 | -0.00519948 | 0.01773295 | 0.284 | 0.731 | 22.643 | 323 | 379 |
| PTR | 0.00389093 | 0.00665186 | -0.00914647 | 0.01692833 | 0.559 | 0.854 | 16.007 | 323 | 379 |
| RLIC | 0.00573915 | 0.00522343 | -0.00449859 | 0.01597689 | 0.272 | 0.731 | 0.870 | 323 | 379 |
| SCC | 0.00521575 | 0.00492076 | -0.00442876 | 0.01486027 | 0.289 | 0.731 | 12.847 | 323 | 379 |
| SCR | 0.01259063 | 0.00457255 | 0.00362858 | 0.02155267 | 0.006 | 0.075 | 0.000 | 323 | 379 |
| SFO | -0.00327533 | 0.00544712 | -0.01395149 | 0.00740083 | 0.548 | 0.854 | 0.000 | 323 | 379 |
| SLF | 0.00291195 | 0.00486842 | -0.00662999 | 0.01245388 | 0.550 | 0.854 | 0.000 | 323 | 379 |
| SS | 0.00084907 | 0.00546843 | -0.00986885 | 0.01156699 | 0.877 | 0.937 | 0.000 | 323 | 379 |
| UNC | 0.01975038 | 0.01139878 | -0.00259083 | 0.04209159 | 0.083 | 0.692 | 36.407 | 323 | 379 |
| AverageFA | 0.00284112 | 0.00285969 | -0.00276376 | 0.00844601 | 0.320 | 0.731 | 0.000 | 323 | 379 |

Table S25. Full meta-analytic results for the Diagnosis by Sex interaction for AD **in adolescents only**. Age, sex, age^2^ and scansite included as covariates.

| Region | Beta | SE | CI LB | CI UB | P-value | FDR P-value | I^2^ | Controls | Patients |
| --- | --- | --- | --- | --- | --- | --- | --- | --- | --- |
| ACR | 0.0000035 | 0.000009 | -0.00001417 | 0.00002123 | 0.696 | 0.986 | 10.242 | 323 | 379 |
| ALIC | 0.0000103 | 0.000010 | -0.00000906 | 0.00002973 | 0.296 | 0.740 | 22.295 | 323 | 379 |
| BCC | 0.0000106 | 0.000014 | -0.00001687 | 0.00003802 | 0.450 | 0.758 | 33.972 | 323 | 379 |
| CC | 0.0000022 | 0.000011 | -0.00001909 | 0.00002357 | 0.837 | 0.986 | 14.135 | 323 | 379 |
| CGC | 0.0000156 | 0.000017 | -0.00001808 | 0.00004927 | 0.364 | 0.758 | 59.952 | 323 | 379 |
| CGH | 0.0000021 | 0.000013 | -0.00002333 | 0.00002743 | 0.874 | 0.986 | 0.352 | 323 | 379 |
| CR | 0.0000073 | 0.000007 | -0.00000584 | 0.0000205 | 0.276 | 0.740 | 0.000 | 323 | 379 |
| CST | 0.0000002 | 0.000013 | -0.00002521 | 0.00002566 | 0.986 | 0.986 | 0.000 | 323 | 379 |
| EC | 0.0000077 | 0.000006 | -0.00000478 | 0.00002024 | 0.226 | 0.740 | 3.402 | 323 | 379 |
| FX | 0.0000365 | 0.000034 | -0.00003059 | 0.00010363 | 0.286 | 0.740 | 0.000 | 323 | 379 |
| FXST | 0.0000014 | 0.000012 | -0.00002214 | 0.00002489 | 0.909 | 0.986 | 11.217 | 323 | 379 |
| GCC | 0.0000088 | 0.000012 | -0.00001424 | 0.00003177 | 0.455 | 0.758 | 0.000 | 323 | 379 |
| IC | 0.0000128 | 0.000007 | -0.00000121 | 0.00002683 | 0.073 | 0.740 | 0.401 | 323 | 379 |
| IFO | 0.0000003 | 0.000011 | -0.00002107 | 0.00002171 | 0.977 | 0.986 | 1.464 | 323 | 379 |
| PCR | 0.0000129 | 0.000009 | -0.00000448 | 0.00003034 | 0.146 | 0.740 | 0.000 | 323 | 379 |
| PLIC | 0.0000152 | 0.000009 | -0.00000267 | 0.000033 | 0.096 | 0.740 | 8.576 | 323 | 379 |
| PTR | 0.0000019 | 0.000011 | -0.00002022 | 0.00002396 | 0.868 | 0.986 | 0.000 | 323 | 379 |
| RLIC | 0.0000104 | 0.000009 | -0.0000082 | 0.00002897 | 0.273 | 0.740 | 0.000 | 323 | 379 |
| SCC | -0.0000090 | 0.000011 | -0.0000315 | 0.00001346 | 0.432 | 0.758 | 0.000 | 323 | 379 |
| SCR | 0.0000109 | 0.000008 | -0.00000455 | 0.00002627 | 0.167 | 0.740 | 11.213 | 323 | 379 |
| SFO | 0.0000010 | 0.000013 | -0.00002411 | 0.00002617 | 0.936 | 0.986 | 33.890 | 323 | 379 |
| SLF | 0.0000057 | 0.000007 | -0.00000706 | 0.0000185 | 0.381 | 0.758 | 0.000 | 323 | 379 |
| SS | -0.0000018 | 0.000010 | -0.00002116 | 0.00001766 | 0.860 | 0.986 | 0.000 | 323 | 379 |
| UNC | 0.0000015 | 0.000021 | -0.00003906 | 0.00004206 | 0.942 | 0.986 | 29.917 | 323 | 379 |
| AverageAD | 0.0000066 | 0.000005 | -0.00000279 | 0.00001596 | 0.169 | 0.740 | 0.000 | 323 | 379 |

Table S26. Full meta-analytic results for the Diagnosis by Sex interaction for MD **in adolescents only**. Age, sex, age^2^ and scansite included as covariates.

| Region | Beta | SE | CI LB | CI UB | P-value | FDR P-value | I^2^ | Controls | Patients |
| --- | --- | --- | --- | --- | --- | --- | --- | --- | --- |
| ACR | 0.00000185 | 0.00000638 | -0.00001066 | 0.00001437 | 0.772 | 0.941 | 13.183 | 323 | 379 |
| ALIC | 0.00000172 | 0.00000423 | -0.00000657 | 0.00001 | 0.685 | 0.941 | 0.502 | 323 | 379 |
| BCC | -0.00000171 | 0.00000675 | -0.00001494 | 0.00001152 | 0.800 | 0.941 | 0.000 | 323 | 379 |
| CC | -0.00000375 | 0.00000566 | -0.00001485 | 0.00000734 | 0.507 | 0.941 | 0.000 | 323 | 379 |
| CGC | 0.00000154 | 0.00000546 | -0.00000916 | 0.00001224 | 0.777 | 0.941 | 0.000 | 323 | 379 |
| CGH | 0.00000141 | 0.00000671 | -0.00001174 | 0.00001455 | 0.834 | 0.941 | 0.000 | 323 | 379 |
| CR | -0.000003 | 0.00000486 | -0.00001252 | 0.00000652 | 0.537 | 0.941 | 3.494 | 323 | 379 |
| CST | -0.00000704 | 0.00000822 | -0.00002314 | 0.00000906 | 0.391 | 0.941 | 0.000 | 323 | 379 |
| EC | 0.00000169 | 0.00000387 | -0.00000591 | 0.00000928 | 0.663 | 0.941 | 0.000 | 323 | 379 |
| FX | 0.00005281 | 0.00002932 | -0.00000466 | 0.00011027 | 0.072 | 0.600 | 0.000 | 323 | 379 |
| FXST | -0.00000731 | 0.00000519 | -0.00001749 | 0.00000286 | 0.159 | 0.663 | 0.560 | 323 | 379 |
| GCC | 0.00000357 | 0.00000699 | -0.00001014 | 0.00001727 | 0.610 | 0.941 | 0.000 | 323 | 379 |
| IC | 0.00000056 | 0.00000421 | -0.0000077 | 0.00000881 | 0.895 | 0.941 | 3.888 | 323 | 379 |
| IFO | 0.00000029 | 0.00000516 | -0.00000983 | 0.0000104 | 0.956 | 0.956 | 0.000 | 323 | 379 |
| PCR | -0.00000872 | 0.00000565 | -0.0000198 | 0.00000236 | 0.123 | 0.615 | 0.000 | 323 | 379 |
| PLIC | 0.00000093 | 0.00000461 | -0.00000811 | 0.00000998 | 0.839 | 0.941 | 0.000 | 323 | 379 |
| PTR | -0.00000765 | 0.00000657 | -0.00002054 | 0.00000523 | 0.244 | 0.871 | 6.249 | 323 | 379 |
| RLIC | -0.00000477 | 0.00000496 | -0.00001448 | 0.00000494 | 0.336 | 0.941 | 0.000 | 323 | 379 |
| SCC | -0.00001165 | 0.00000551 | -0.00002244 | -0.00000085 | 0.034 | 0.600 | 0.000 | 323 | 379 |
| SCR | -0.00000337 | 0.00000451 | -0.00001222 | 0.00000547 | 0.455 | 0.941 | 6.684 | 323 | 379 |
| SFO | -0.00000228 | 0.00000534 | -0.00001274 | 0.00000818 | 0.669 | 0.941 | 0.000 | 323 | 379 |
| SLF | -0.00000052 | 0.00000432 | -0.00000899 | 0.00000794 | 0.903 | 0.941 | 0.000 | 323 | 379 |
| SS | -0.00000921 | 0.00000592 | -0.00002081 | 0.0000024 | 0.120 | 0.615 | 0.000 | 323 | 379 |
| UNC | -0.00001724 | 0.00000929 | -0.00003545 | 0.00000097 | 0.064 | 0.600 | 0.000 | 323 | 379 |
| AverageMD | 0.00000066 | 0.00000376 | -0.00000671 | 0.00000802 | 0.862 | 0.941 | 0.000 | 323 | 379 |

Table S27. Full meta-analytic results for the Diagnosis by Sex interaction for RD **in adolescents only**. Age, sex, age^2^ and scansite included as covariates.

| Region | Beta | SE | CI LB | CI UB | P-value | FDR P-value | I^2^ | Controls | Patients |
| --- | --- | --- | --- | --- | --- | --- | --- | --- | --- |
| ACR | 0.00000529 | 0.00000736 | -0.00000914 | 0.00001972 | 0.472 | 0.716 | 21.868 | 323 | 379 |
| ALIC | -0.00000295 | 0.00000424 | -0.00001126 | 0.00000537 | 0.487 | 0.716 | 0.000 | 323 | 379 |
| BCC | -0.00000635 | 0.00000887 | -0.00002374 | 0.00001104 | 0.474 | 0.716 | 0.000 | 323 | 379 |
| CC | -0.00000656 | 0.00000658 | -0.00001945 | 0.00000634 | 0.319 | 0.665 | 0.000 | 323 | 379 |
| CGC | 0.00000182 | 0.00000721 | -0.0000123 | 0.00001594 | 0.801 | 0.859 | 9.312 | 323 | 379 |
| CGH | 0.00000269 | 0.00000697 | -0.00001096 | 0.00001635 | 0.699 | 0.859 | 0.000 | 323 | 379 |
| CR | -0.00000477 | 0.00000545 | -0.00001545 | 0.00000591 | 0.382 | 0.716 | 8.692 | 323 | 379 |
| CST | -0.00000588 | 0.00000836 | -0.00002227 | 0.00001051 | 0.482 | 0.716 | 0.000 | 323 | 379 |
| EC | 0.00000091 | 0.00000415 | -0.00000721 | 0.00000904 | 0.825 | 0.859 | 0.000 | 323 | 379 |
| FX | 0.00005333 | 0.00002813 | -0.0000018 | 0.00010846 | 0.058 | 0.242 | 0.000 | 323 | 379 |
| FXST | -0.00000994 | 0.00000578 | -0.00002126 | 0.00000139 | 0.085 | 0.286 | 0.000 | 323 | 379 |
| GCC | -0.00000217 | 0.0000074 | -0.00001667 | 0.00001233 | 0.769 | 0.859 | 0.000 | 323 | 379 |
| IC | -0.00000675 | 0.00000414 | -0.00001486 | 0.00000136 | 0.103 | 0.286 | 0.000 | 323 | 379 |
| IFO | 0.00000193 | 0.00000692 | -0.00001164 | 0.0000155 | 0.780 | 0.859 | 0.000 | 323 | 379 |
| PCR | -0.00001557 | 0.00000602 | -0.00002737 | -0.00000378 | 0.010 | 0.125 | 0.000 | 323 | 379 |
| PLIC | -0.00000665 | 0.00000512 | -0.00001668 | 0.00000338 | 0.194 | 0.441 | 0.000 | 323 | 379 |
| PTR | -0.00001279 | 0.00000599 | -0.00002453 | -0.00000106 | 0.033 | 0.180 | 0.000 | 323 | 379 |
| RLIC | -0.00000901 | 0.00000547 | -0.00001974 | 0.00000172 | 0.100 | 0.286 | 0.000 | 323 | 379 |
| SCC | -0.00001195 | 0.0000057 | -0.00002312 | -0.00000078 | 0.036 | 0.180 | 0.000 | 323 | 379 |
| SCR | -0.00000977 | 0.00000458 | -0.00001875 | -0.0000008 | 0.033 | 0.180 | 0.000 | 323 | 379 |
| SFO | -0.00000062 | 0.00000514 | -0.0000107 | 0.00000946 | 0.904 | 0.904 | 0.000 | 323 | 379 |
| SLF | -0.00000234 | 0.00000496 | -0.00001205 | 0.00000737 | 0.637 | 0.859 | 0.000 | 323 | 379 |
| SS | -0.00000905 | 0.00000625 | -0.00002131 | 0.00000321 | 0.148 | 0.370 | 0.000 | 323 | 379 |
| UNC | -0.00003468 | 0.00001022 | -0.0000547 | -0.00001465 | 0.001 | 0.025 | 0.000 | 323 | 379 |
| AverageRD | -0.00000122 | 0.00000382 | -0.00000871 | 0.00000626 | 0.749 | 0.859 | 0.000 | 323 | 379 |

**4: Diagnosis-by-age interaction effects**

Table S28. Full meta-analytic results for the Diagnosis by Age interaction for FA. Age, sex, age^2^ and scansite included as covariates.

| Region | Beta | SE | CI LB | CI UB | P-value | FDR P-value | I^2^ | Controls | Patients |
| --- | --- | --- | --- | --- | --- | --- | --- | --- | --- |
| ACR | -0.0000675 | 0.0000832 | -0.0002306 | 0.0000956 | 0.417 | 0.549 | 4.966 | 1595 | 1308 |
| ALIC | -0.0000515 | 0.0000972 | -0.0002419 | 0.0001389 | 0.596 | 0.710 | 10.302 | 1595 | 1308 |
| BCC | -0.0001921 | 0.0000679 | -0.0003251 | -0.0000591 | 0.005 | 0.033 | 0.000 | 1595 | 1308 |
| CC | -0.0001586 | 0.0000597 | -0.0002757 | -0.0000415 | 0.008 | 0.033 | 0.969 | 1595 | 1308 |
| CGC | -0.0001145 | 0.0000797 | -0.0002707 | 0.0000418 | 0.151 | 0.342 | 0.000 | 1595 | 1308 |
| CGH | -0.0000544 | 0.0000441 | -0.0001408 | 0.0000321 | 0.218 | 0.389 | 0.000 | 1595 | 1308 |
| CR | -0.0000955 | 0.0000686 | -0.0002300 | 0.0000389 | 0.164 | 0.342 | 5.207 | 1595 | 1308 |
| CST | -0.0000047 | 0.0001514 | -0.0003014 | 0.0002920 | 0.975 | 0.975 | 0.000 | 1595 | 1308 |
| EC | -0.0000466 | 0.0000604 | -0.0001651 | 0.0000719 | 0.441 | 0.551 | 4.101 | 1595 | 1308 |
| FX | -0.0001268 | 0.0001274 | -0.0003764 | 0.0001229 | 0.320 | 0.479 | 0.000 | 1595 | 1308 |
| FXST | -0.0001499 | 0.0000540 | -0.0002557 | -0.0000441 | 0.005 | 0.033 | 0.000 | 1595 | 1308 |
| GCC | -0.0001890 | 0.0000646 | -0.0003156 | -0.0000625 | 0.003 | 0.033 | 0.665 | 1595 | 1308 |
| IC | -0.0000954 | 0.0000719 | -0.0002363 | 0.0000455 | 0.185 | 0.356 | 5.313 | 1595 | 1308 |
| IFO | 0.0000280 | 0.0001273 | -0.0002215 | 0.0002775 | 0.826 | 0.860 | 0.000 | 1595 | 1308 |
| PCR | -0.0004169 | 0.0004246 | -0.0012490 | 0.0004152 | 0.326 | 0.479 | 94.724 | 1595 | 1308 |
| PLIC | -0.0000763 | 0.0000856 | -0.0002440 | 0.0000915 | 0.373 | 0.518 | 5.964 | 1595 | 1308 |
| PTR | -0.0000688 | 0.0000449 | -0.0001567 | 0.0000192 | 0.125 | 0.313 | 0.000 | 1595 | 1308 |
| RLIC | -0.0001318 | 0.0000542 | -0.0002380 | -0.0000255 | 0.015 | 0.054 | 0.000 | 1595 | 1308 |
| SCC | -0.0001077 | 0.0000492 | -0.0002040 | -0.0000113 | 0.029 | 0.091 | 0.000 | 1595 | 1308 |
| SCR | -0.0001092 | 0.0000531 | -0.0002132 | -0.0000052 | 0.040 | 0.111 | 1.343 | 1595 | 1308 |
| SFO | -0.0000392 | 0.0000857 | -0.0002072 | 0.0001287 | 0.647 | 0.735 | 0.001 | 1595 | 1308 |
| SLF | -0.0000871 | 0.0000796 | -0.0002430 | 0.0000689 | 0.274 | 0.457 | 5.827 | 1595 | 1308 |
| SS | -0.0001521 | 0.0000559 | -0.0002617 | -0.0000425 | 0.007 | 0.033 | 0.662 | 1595 | 1308 |
| UNC | 0.0001353 | 0.0005860 | -0.0010132 | 0.0012838 | 0.817 | 0.860 | 88.100 | 1595 | 1308 |
| AverageFA | -0.0000834 | 0.0000308 | -0.0001437 | -0.0000230 | 0.007 | 0.033 | 0.712 | 1595 | 1308 |

Table S29. Full meta-analytic results for the Diagnosis by Age interaction for AD. Age, sex, age^2^ and scansite included as covariates.

| Region | Beta | SE | CI LB | CI UB | P-value | FDR P-value | I^2^ | Controls | Patients |
| --- | --- | --- | --- | --- | --- | --- | --- | --- | --- |
| ACR | 0.0000004 | 0.0000002 | 0.0000000 | 0.0000007 | 0.037 | 0.185 | 0.000 | 1595 | 1308 |
| ALIC | -0.0000005 | 0.0000161 | -0.0000320 | 0.0000310 | 0.976 | 0.997 | 99.986 | 1595 | 1308 |
| BCC | 0.0000005 | 0.0000129 | -0.0000249 | 0.0000258 | 0.971 | 0.997 | 99.969 | 1595 | 1308 |
| CC | -0.0000012 | 0.0000256 | -0.0000512 | 0.0000489 | 0.964 | 0.997 | 99.994 | 1595 | 1308 |
| CGC | -0.0000097 | 0.0000633 | -0.0001337 | 0.0001143 | 0.878 | 0.997 | 99.998 | 1595 | 1308 |
| CGH | 0.0000001 | 0.0000003 | -0.0000004 | 0.0000007 | 0.611 | 0.997 | 0.000 | 1595 | 1308 |
| CR | 0.0000003 | 0.0000001 | 0.0000001 | 0.0000006 | 0.020 | 0.144 | 0.000 | 1595 | 1308 |
| CST | 0.0000000 | 0.0000003 | -0.0000006 | 0.0000007 | 0.904 | 0.997 | 6.960 | 1595 | 1308 |
| EC | -0.0000014 | 0.0000243 | -0.0000490 | 0.0000462 | 0.954 | 0.997 | 99.996 | 1595 | 1308 |
| FX | 0.0000008 | 0.0000009 | -0.0000010 | 0.0000026 | 0.373 | 0.997 | 1.595 | 1595 | 1308 |
| FXST | -0.0000002 | 0.0000002 | -0.0000006 | 0.0000003 | 0.454 | 0.997 | 5.017 | 1595 | 1308 |
| GCC | -0.0000014 | 0.0000283 | -0.0000568 | 0.0000541 | 0.962 | 0.997 | 99.992 | 1595 | 1308 |
| IC | -0.0000003 | 0.0000116 | -0.0000230 | 0.0000225 | 0.982 | 0.997 | 99.978 | 1595 | 1308 |
| IFO | 0.0000002 | 0.0000002 | -0.0000003 | 0.0000006 | 0.467 | 0.997 | 0.226 | 1595 | 1308 |
| PCR | 0.0000004 | 0.0000002 | 0.0000001 | 0.0000007 | 0.021 | 0.144 | 0.000 | 1595 | 1308 |
| PLIC | 0.0000002 | 0.0000002 | -0.0000002 | 0.0000006 | 0.312 | 0.997 | 0.000 | 1595 | 1308 |
| PTR | 0.0000001 | 0.0000002 | -0.0000003 | 0.0000004 | 0.711 | 0.997 | 0.000 | 1595 | 1308 |
| RLIC | -0.0000012 | 0.0000204 | -0.0000412 | 0.0000387 | 0.952 | 0.997 | 99.990 | 1595 | 1308 |
| SCC | -0.0000022 | 0.0000272 | -0.0000555 | 0.0000511 | 0.935 | 0.997 | 99.993 | 1595 | 1308 |
| SCR | 0.0000004 | 0.0000002 | 0.0000001 | 0.0000007 | 0.023 | 0.144 | 0.000 | 1595 | 1308 |
| SFO | 0.0000006 | 0.0000002 | 0.0000001 | 0.0000010 | 0.012 | 0.144 | 5.150 | 1595 | 1308 |
| SLF | 0.0000000 | 0.0000072 | -0.0000140 | 0.0000140 | 0.997 | 0.997 | 99.949 | 1595 | 1308 |
| SS | -0.0000008 | 0.0000175 | -0.0000352 | 0.0000336 | 0.964 | 0.997 | 99.986 | 1595 | 1308 |
| UNC | 0.0000006 | 0.0000003 | 0.0000000 | 0.0000012 | 0.049 | 0.204 | 0.000 | 1595 | 1308 |
| AverageAD | -0.0000001 | 0.0000069 | -0.0000136 | 0.0000134 | 0.990 | 0.997 | 99.978 | 1595 | 1308 |

Table S30. Full meta-analytic results for the Diagnosis by Age interaction for MD. Age, sex, age^2^ and scansite included as covariates.

| Region | Beta | SE | CI LB | CI UB | P-value | FDR P-value | I^2^ | Controls | Patients |
| --- | --- | --- | --- | --- | --- | --- | --- | --- | --- |
| ACR | 0.0000003 | 0.0000001 | 0.0000001 | 0.0000005 | 0.019 | 0.200 | 0.000 | 1595 | 1308 |
| ALIC | -0.0000002 | 0.0000161 | -0.0000317 | 0.0000313 | 0.991 | 0.994 | 99.996 | 1595 | 1308 |
| BCC | 0.0000008 | 0.0000130 | -0.0000246 | 0.0000262 | 0.950 | 0.994 | 99.982 | 1595 | 1308 |
| CC | -0.0000007 | 0.0000256 | -0.0000508 | 0.0000494 | 0.978 | 0.994 | 99.997 | 1595 | 1308 |
| CGC | -0.0000088 | 0.0000633 | -0.0001329 | 0.0001152 | 0.889 | 0.994 | 100.000 | 1595 | 1308 |
| CGH | 0.0000001 | 0.0000002 | -0.0000002 | 0.0000004 | 0.477 | 0.994 | 0.803 | 1595 | 1308 |
| CR | 0.0000003 | 0.0000001 | 0.0000000 | 0.0000005 | 0.022 | 0.200 | 2.177 | 1595 | 1308 |
| CST | 0.0000000 | 0.0000002 | -0.0000004 | 0.0000003 | 0.826 | 0.994 | 3.818 | 1595 | 1308 |
| EC | -0.0000012 | 0.0000243 | -0.0000488 | 0.0000465 | 0.962 | 0.994 | 99.998 | 1595 | 1308 |
| FX | 0.0000006 | 0.0000009 | -0.0000011 | 0.0000024 | 0.473 | 0.994 | 2.425 | 1595 | 1308 |
| FXST | -0.0000001 | 0.0000001 | -0.0000003 | 0.0000002 | 0.510 | 0.994 | 6.377 | 1595 | 1308 |
| GCC | -0.0000011 | 0.0000283 | -0.0000566 | 0.0000543 | 0.969 | 0.994 | 99.996 | 1595 | 1308 |
| IC | -0.0000002 | 0.0000116 | -0.0000229 | 0.0000226 | 0.989 | 0.994 | 99.993 | 1595 | 1308 |
| IFO | 0.0000001 | 0.0000001 | -0.0000002 | 0.0000003 | 0.631 | 0.994 | 6.418 | 1595 | 1308 |
| PCR | 0.0000003 | 0.0000001 | 0.0000000 | 0.0000005 | 0.024 | 0.200 | 0.000 | 1595 | 1308 |
| PLIC | 0.0000000 | 0.0000001 | -0.0000002 | 0.0000002 | 0.968 | 0.994 | 0.000 | 1595 | 1308 |
| PTR | 0.0000001 | 0.0000001 | -0.0000001 | 0.0000004 | 0.327 | 0.994 | 0.000 | 1595 | 1308 |
| RLIC | -0.0000008 | 0.0000204 | -0.0000408 | 0.0000391 | 0.967 | 0.994 | 99.997 | 1595 | 1308 |
| SCC | -0.0000014 | 0.0000272 | -0.0000547 | 0.0000519 | 0.959 | 0.994 | 99.998 | 1595 | 1308 |
| SCR | 0.0000002 | 0.0000001 | 0.0000000 | 0.0000005 | 0.056 | 0.350 | 8.997 | 1595 | 1308 |
| SFO | 0.0000002 | 0.0000001 | -0.0000001 | 0.0000004 | 0.174 | 0.870 | 3.006 | 1595 | 1308 |
| SLF | -0.0000001 | 0.0000071 | -0.0000141 | 0.0000139 | 0.994 | 0.994 | 99.978 | 1595 | 1308 |
| SS | -0.0000004 | 0.0000175 | -0.0000347 | 0.0000340 | 0.984 | 0.994 | 99.994 | 1595 | 1308 |
| UNC | 0.0000001 | 0.0000002 | -0.0000003 | 0.0000005 | 0.651 | 0.994 | 0.000 | 1595 | 1308 |
| AverageMD | 0.0000001 | 0.0000069 | -0.0000134 | 0.0000137 | 0.987 | 0.994 | 99.981 | 1595 | 1308 |

Table S31. Full meta-analytic results for the Diagnosis by Age interaction for RD. Age, sex, age^2^ and scansite included as covariates.

| Region | Beta | SE | CI LB | CI UB | P-value | FDR P-value | I^2^ | Controls | Patients |
| --- | --- | --- | --- | --- | --- | --- | --- | --- | --- |
| ACR | 0.0000001 | 0.0000001 | -0.0000001 | 0.0000004 | 0.279 | 0.999 | 0.000 | 1577 | 1294 |
| ALIC | 0.0000000 | 0.0000168 | -0.0000330 | 0.0000329 | 1.000 | 0.999 | 99.995 | 1577 | 1294 |
| BCC | 0.0000010 | 0.0000136 | -0.0000255 | 0.0000276 | 0.939 | 0.999 | 99.978 | 1577 | 1294 |
| CC | -0.0000007 | 0.0000267 | -0.0000530 | 0.0000517 | 0.981 | 0.999 | 99.997 | 1577 | 1294 |
| CGC | -0.0000091 | 0.0000662 | -0.0001388 | 0.0001207 | 0.891 | 0.999 | 99.999 | 1577 | 1294 |
| CGH | 0.0000002 | 0.0000002 | -0.0000002 | 0.0000005 | 0.293 | 0.999 | 0.142 | 1577 | 1294 |
| CR | 0.0000002 | 0.0000001 | -0.0000001 | 0.0000004 | 0.198 | 0.999 | 4.527 | 1577 | 1294 |
| CST | 0.0000001 | 0.0000002 | -0.0000003 | 0.0000005 | 0.649 | 0.999 | 2.227 | 1577 | 1294 |
| EC | -0.0000011 | 0.0000254 | -0.0000508 | 0.0000487 | 0.966 | 0.999 | 99.998 | 1577 | 1294 |
| FX | 0.0000007 | 0.0000009 | -0.0000012 | 0.0000025 | 0.485 | 0.999 | 2.143 | 1577 | 1294 |
| FXST | 0.0000001 | 0.0000001 | -0.0000002 | 0.0000003 | 0.507 | 0.999 | 0.000 | 1577 | 1294 |
| GCC | -0.0000011 | 0.0000296 | -0.0000591 | 0.0000568 | 0.970 | 0.999 | 99.997 | 1577 | 1294 |
| IC | -0.0000001 | 0.0000121 | -0.0000238 | 0.0000237 | 0.997 | 0.999 | 99.993 | 1577 | 1294 |
| IFO | 0.0000001 | 0.0000002 | -0.0000003 | 0.0000004 | 0.701 | 0.999 | 7.587 | 1577 | 1294 |
| PCR | 0.0000002 | 0.0000002 | -0.0000001 | 0.0000005 | 0.210 | 0.999 | 4.381 | 1577 | 1294 |
| PLIC | 0.0000000 | 0.0000001 | -0.0000002 | 0.0000003 | 0.749 | 0.999 | 4.761 | 1577 | 1294 |
| PTR | 0.0000001 | 0.0000001 | -0.0000002 | 0.0000003 | 0.624 | 0.999 | 0.830 | 1577 | 1294 |
| RLIC | -0.0000006 | 0.0000213 | -0.0000424 | 0.0000412 | 0.978 | 0.999 | 99.996 | 1577 | 1294 |
| SCC | -0.0000014 | 0.0000284 | -0.0000570 | 0.0000544 | 0.962 | 0.999 | 99.998 | 1577 | 1294 |
| SCR | 0.0000002 | 0.0000001 | -0.0000001 | 0.0000004 | 0.197 | 0.999 | 10.467 | 1577 | 1294 |
| SFO | 0.0000000 | 0.0000001 | -0.0000002 | 0.0000003 | 0.767 | 0.999 | 0.653 | 1577 | 1294 |
| SLF | 0.0000000 | 0.0000075 | -0.0000146 | 0.0000147 | 0.998 | 0.999 | 99.973 | 1577 | 1294 |
| SS | -0.0000001 | 0.0000183 | -0.0000361 | 0.0000358 | 0.995 | 0.999 | 99.993 | 1577 | 1294 |
| UNC | -0.0000001 | 0.0000003 | -0.0000007 | 0.0000004 | 0.626 | 0.999 | 2.512 | 1577 | 1294 |
| AverageRD | 0.0000002 | 0.0000072 | -0.0000139 | 0.0000144 | 0.974 | 0.999 | 99.979 | 1577 | 1294 |

**5: First and recurrent MDD**

Table S32. Cohen’s d effect sizes after meta-analysis, for FA differences between patients with recurrent MDD and controls **in adults only**. Age, sex, agexsex, age^2^, age^2^xsex and scansite included as covariates.

| Region | Cohen’s d | SE | CI LB | CI UB | P-value | FDR P-value | I^2^ | Controls | Patients |
| --- | --- | --- | --- | --- | --- | --- | --- | --- | --- |
| ACR | -0.329 | 0.096 | -0.518 | -0.141 | 0.001 | 0.010 | 58.053 | 1053 | 645 |
| ALIC | -0.294 | 0.113 | -0.515 | -0.074 | 0.009 | 0.025 | 69.683 | 1053 | 645 |
| BCC | -0.263 | 0.094 | -0.448 | -0.078 | 0.005 | 0.018 | 56.608 | 1053 | 645 |
| CC | -0.280 | 0.093 | -0.462 | -0.098 | 0.003 | 0.013 | 54.868 | 1053 | 645 |
| CGC | -0.217 | 0.108 | -0.428 | -0.006 | 0.043 | 0.063 | 66.441 | 1053 | 645 |
| CGH | -0.059 | 0.066 | -0.188 | 0.069 | 0.367 | 0.382 | 20.317 | 1053 | 645 |
| CR | -0.317 | 0.101 | -0.514 | -0.120 | 0.002 | 0.010 | 61.644 | 1053 | 645 |
| CST | -0.052 | 0.100 | -0.247 | 0.143 | 0.599 | 0.599 | 61.220 | 1053 | 645 |
| EC | -0.207 | 0.105 | -0.412 | -0.002 | 0.048 | 0.066 | 64.838 | 1053 | 645 |
| FX | -0.052 | 0.053 | -0.155 | 0.051 | 0.322 | 0.350 | 0.000 | 1053 | 645 |
| FXST | -0.161 | 0.086 | -0.330 | 0.009 | 0.063 | 0.075 | 48.755 | 1053 | 645 |
| GCC | -0.319 | 0.090 | -0.496 | -0.142 | 0.000 | 0.000 | 52.437 | 1053 | 645 |
| IC | -0.284 | 0.116 | -0.512 | -0.056 | 0.015 | 0.034 | 71.795 | 1053 | 645 |
| IFO | -0.200 | 0.088 | -0.372 | -0.028 | 0.023 | 0.043 | 49.995 | 1053 | 645 |
| PCR | -0.221 | 0.085 | -0.387 | -0.054 | 0.009 | 0.025 | 46.787 | 1053 | 645 |
| PLIC | -0.215 | 0.122 | -0.454 | 0.024 | 0.078 | 0.089 | 74.513 | 1053 | 645 |
| PTR | -0.212 | 0.089 | -0.387 | -0.037 | 0.017 | 0.035 | 51.499 | 1053 | 645 |
| RLIC | -0.209 | 0.093 | -0.390 | -0.028 | 0.024 | 0.043 | 54.972 | 1053 | 645 |
| SCC | -0.140 | 0.071 | -0.279 | 0.000 | 0.050 | 0.066 | 28.757 | 1053 | 645 |
| SCR | -0.235 | 0.110 | -0.451 | -0.018 | 0.034 | 0.053 | 68.579 | 1053 | 645 |
| SFO | -0.242 | 0.079 | -0.398 | -0.086 | 0.002 | 0.010 | 39.976 | 1053 | 645 |
| SLF | -0.219 | 0.100 | -0.414 | -0.024 | 0.028 | 0.047 | 61.120 | 1053 | 645 |
| SS | -0.260 | 0.101 | -0.458 | -0.063 | 0.010 | 0.025 | 61.850 | 1053 | 645 |
| UNC | -0.197 | 0.105 | -0.403 | 0.009 | 0.061 | 0.075 | 65.219 | 1053 | 645 |
| AverageFA | -0.298 | 0.094 | -0.483 | -0.114 | 0.002 | 0.010 | 56.067 | 1053 | 645 |

Table S33. Cohen’s d effect sizes after meta-analysis, for AD differences between patients with recurrent MDD and controls **in adults only**. Age, sex, agexsex, age^2^, age^2^xsex and scansite included as covariates.

| Region | Cohen’s d | SE | CI LB | CI UB | P-value | FDR P-value | I^2^ | Controls | Patients |
| --- | --- | --- | --- | --- | --- | --- | --- | --- | --- |
| ACR | -0.010 | 0.120 | -0.245 | 0.225 | 0.934 | 0.934 | 74.001 | 1053 | 645 |
| ALIC | 0.099 | 0.119 | -0.133 | 0.331 | 0.404 | 0.760 | 73.191 | 1053 | 645 |
| BCC | 0.086 | 0.118 | -0.145 | 0.316 | 0.467 | 0.760 | 72.814 | 1053 | 645 |
| CC | 0.070 | 0.116 | -0.157 | 0.297 | 0.547 | 0.760 | 71.851 | 1053 | 645 |
| CGC | -0.048 | 0.118 | -0.278 | 0.183 | 0.686 | 0.808 | 72.829 | 1053 | 645 |
| CGH | 0.064 | 0.107 | -0.145 | 0.274 | 0.546 | 0.760 | 66.518 | 1053 | 645 |
| CR | 0.056 | 0.113 | -0.166 | 0.278 | 0.622 | 0.778 | 70.461 | 1053 | 645 |
| CST | 0.148 | 0.118 | -0.084 | 0.379 | 0.212 | 0.760 | 72.970 | 1053 | 645 |
| EC | 0.112 | 0.117 | -0.118 | 0.342 | 0.338 | 0.760 | 72.653 | 1053 | 645 |
| FX | 0.076 | 0.106 | -0.132 | 0.284 | 0.474 | 0.760 | 66.093 | 1053 | 645 |
| FXST | 0.128 | 0.118 | -0.104 | 0.360 | 0.278 | 0.760 | 73.031 | 1053 | 645 |
| GCC | 0.031 | 0.115 | -0.195 | 0.257 | 0.787 | 0.820 | 71.712 | 1053 | 645 |
| IC | 0.103 | 0.118 | -0.128 | 0.333 | 0.383 | 0.760 | 72.766 | 1053 | 645 |
| IFO | -0.052 | 0.144 | -0.336 | 0.231 | 0.717 | 0.808 | 82.481 | 1053 | 645 |
| PCR | 0.078 | 0.124 | -0.164 | 0.320 | 0.527 | 0.760 | 75.514 | 1053 | 645 |
| PLIC | 0.145 | 0.112 | -0.075 | 0.366 | 0.196 | 0.760 | 69.958 | 1053 | 645 |
| PTR | 0.130 | 0.135 | -0.135 | 0.394 | 0.336 | 0.760 | 79.743 | 1053 | 645 |
| RLIC | 0.087 | 0.121 | -0.149 | 0.324 | 0.469 | 0.760 | 74.278 | 1053 | 645 |
| SCC | 0.092 | 0.116 | -0.135 | 0.319 | 0.428 | 0.760 | 72.018 | 1053 | 645 |
| SCR | 0.134 | 0.107 | -0.076 | 0.345 | 0.210 | 0.760 | 66.863 | 1053 | 645 |
| SFO | 0.065 | 0.128 | -0.186 | 0.315 | 0.612 | 0.778 | 77.169 | 1053 | 645 |
| SLF | 0.118 | 0.119 | -0.115 | 0.352 | 0.320 | 0.760 | 73.489 | 1053 | 645 |
| SS | 0.090 | 0.119 | -0.143 | 0.322 | 0.451 | 0.760 | 73.322 | 1053 | 645 |
| UNC | 0.046 | 0.139 | -0.226 | 0.317 | 0.743 | 0.808 | 80.867 | 1053 | 645 |
| AverageAD | 0.117 | 0.109 | -0.096 | 0.330 | 0.281 | 0.760 | 67.713 | 1053 | 645 |

Table S34. Cohen’s d effect sizes after meta-analysis, for MD differences between patients with recurrent MDD and controls **in adults only**. Age, sex, agexsex, age^2^, age^2^xsex and scansite included as covariates.

| Region | Cohen’s d | SE | CI LB | CI UB | P-value | FDR P-value | I^2^ | Controls | Patients |
| --- | --- | --- | --- | --- | --- | --- | --- | --- | --- |
| ACR | 0.103 | 0.100 | -0.092 | 0.299 | 0.300 | 0.375 | 61.276 | 1053 | 645 |
| ALIC | 0.168 | 0.120 | -0.068 | 0.404 | 0.163 | 0.291 | 73.851 | 1053 | 645 |
| BCC | 0.178 | 0.108 | -0.033 | 0.390 | 0.098 | 0.235 | 67.030 | 1053 | 645 |
| CC | 0.161 | 0.099 | -0.033 | 0.354 | 0.104 | 0.235 | 60.549 | 1053 | 645 |
| CGC | 0.063 | 0.110 | -0.153 | 0.280 | 0.566 | 0.590 | 68.754 | 1053 | 645 |
| CGH | 0.118 | 0.090 | -0.059 | 0.294 | 0.192 | 0.317 | 52.716 | 1053 | 645 |
| CR | 0.153 | 0.089 | -0.022 | 0.327 | 0.086 | 0.235 | 51.387 | 1053 | 645 |
| CST | 0.116 | 0.104 | -0.088 | 0.320 | 0.263 | 0.365 | 64.645 | 1053 | 645 |
| EC | 0.186 | 0.111 | -0.031 | 0.403 | 0.093 | 0.235 | 68.793 | 1053 | 645 |
| FX | 0.059 | 0.094 | -0.125 | 0.243 | 0.531 | 0.577 | 56.375 | 1053 | 645 |
| FXST | 0.228 | 0.102 | 0.028 | 0.428 | 0.026 | 0.235 | 62.843 | 1053 | 645 |
| GCC | 0.122 | 0.078 | -0.031 | 0.274 | 0.117 | 0.235 | 37.971 | 1053 | 645 |
| IC | 0.124 | 0.102 | -0.075 | 0.323 | 0.224 | 0.329 | 62.680 | 1053 | 645 |
| IFO | 0.007 | 0.086 | -0.162 | 0.176 | 0.936 | 0.936 | 48.468 | 1053 | 645 |
| PCR | 0.158 | 0.079 | 0.002 | 0.313 | 0.047 | 0.235 | 40.106 | 1053 | 645 |
| PLIC | 0.089 | 0.101 | -0.109 | 0.288 | 0.379 | 0.431 | 62.589 | 1053 | 645 |
| PTR | 0.157 | 0.102 | -0.042 | 0.356 | 0.122 | 0.235 | 62.839 | 1053 | 645 |
| RLIC | 0.101 | 0.094 | -0.084 | 0.286 | 0.284 | 0.374 | 56.596 | 1053 | 645 |
| SCC | 0.115 | 0.090 | -0.062 | 0.292 | 0.203 | 0.317 | 52.818 | 1053 | 645 |
| SCR | 0.213 | 0.079 | 0.058 | 0.368 | 0.007 | 0.175 | 39.586 | 1053 | 645 |
| SFO | 0.168 | 0.095 | -0.019 | 0.355 | 0.077 | 0.235 | 57.522 | 1053 | 645 |
| SLF | 0.165 | 0.093 | -0.016 | 0.347 | 0.075 | 0.235 | 55.052 | 1053 | 645 |
| SS | 0.128 | 0.082 | -0.033 | 0.290 | 0.120 | 0.235 | 44.056 | 1053 | 645 |
| UNC | 0.089 | 0.092 | -0.091 | 0.269 | 0.333 | 0.396 | 54.401 | 1053 | 645 |
| AverageMD | 0.194 | 0.095 | 0.008 | 0.381 | 0.041 | 0.235 | 57.186 | 1053 | 645 |

Table S35. Cohen’s d effect sizes after meta-analysis, for RD differences between patients with recurrent MDD and controls **in adults only**. Age, sex, agexsex, age^2^, age^2^xsex and scansite included as covariates.

| Region | Cohen’s d | SE | CI LB | CI UB | P-value | FDR P-value | I^2^ | Controls | Patients |
| --- | --- | --- | --- | --- | --- | --- | --- | --- | --- |
| ACR | 0.130 | 0.059 | 0.015 | 0.245 | 0.026 | 0.117 | 8.929 | 1053 | 645 |
| ALIC | 0.183 | 0.088 | 0.011 | 0.355 | 0.037 | 0.119 | 49.557 | 1053 | 645 |
| BCC | 0.158 | 0.076 | 0.009 | 0.308 | 0.038 | 0.119 | 35.708 | 1053 | 645 |
| CC | 0.047 | 0.053 | -0.056 | 0.150 | 0.372 | 0.547 | 0.013 | 1053 | 645 |
| CGC | 0.125 | 0.126 | -0.122 | 0.372 | 0.322 | 0.508 | 76.369 | 1053 | 645 |
| CGH | 0.118 | 0.072 | -0.022 | 0.259 | 0.100 | 0.227 | 29.668 | 1053 | 645 |
| CR | 0.143 | 0.053 | 0.040 | 0.247 | 0.006 | 0.075 | 0.016 | 1053 | 645 |
| CST | 0.096 | 0.097 | -0.094 | 0.287 | 0.323 | 0.508 | 59.250 | 1053 | 645 |
| EC | 0.184 | 0.107 | -0.025 | 0.394 | 0.085 | 0.213 | 66.417 | 1053 | 645 |
| FX | 0.062 | 0.087 | -0.108 | 0.232 | 0.474 | 0.658 | 49.012 | 1053 | 645 |
| FXST | 0.152 | 0.063 | 0.029 | 0.275 | 0.015 | 0.094 | 15.474 | 1053 | 645 |
| GCC | 0.041 | 0.086 | -0.128 | 0.211 | 0.632 | 0.790 | 48.617 | 1053 | 645 |
| IC | 0.092 | 0.094 | -0.091 | 0.276 | 0.325 | 0.508 | 55.729 | 1053 | 645 |
| IFO | 0.007 | 0.096 | -0.182 | 0.195 | 0.945 | 0.964 | 58.481 | 1053 | 645 |
| PCR | 0.083 | 0.076 | -0.066 | 0.233 | 0.274 | 0.508 | 35.986 | 1053 | 645 |
| PLIC | 0.023 | 0.115 | -0.202 | 0.248 | 0.840 | 0.932 | 71.205 | 1053 | 645 |
| PTR | 0.065 | 0.128 | -0.185 | 0.316 | 0.609 | 0.790 | 77.318 | 1053 | 645 |
| RLIC | -0.012 | 0.064 | -0.137 | 0.114 | 0.857 | 0.932 | 18.023 | 1053 | 645 |
| SCC | 0.025 | 0.118 | -0.207 | 0.256 | 0.834 | 0.932 | 73.007 | 1053 | 645 |
| SCR | 0.175 | 0.053 | 0.072 | 0.278 | 0.001 | 0.025 | 0.001 | 1053 | 645 |
| SFO | 0.116 | 0.053 | 0.013 | 0.219 | 0.028 | 0.117 | 0.000 | 1053 | 645 |
| SLF | 0.096 | 0.053 | -0.007 | 0.199 | 0.068 | 0.189 | 0.000 | 1053 | 645 |
| SS | 0.081 | 0.053 | -0.022 | 0.184 | 0.123 | 0.256 | 0.015 | 1053 | 645 |
| UNC | -0.004 | 0.096 | -0.193 | 0.185 | 0.964 | 0.964 | 58.712 | 1053 | 645 |
| AverageRD | 0.165 | 0.064 | 0.039 | 0.291 | 0.010 | 0.083 | 17.937 | 1053 | 645 |

Table S36. Cohen’s d effect sizes after meta-analysis, for FA differences between patients with first episode MDD and controls **in adults only**. Age, sex, agexsex, age^2^, age^2^xsex and scansite included as covariates.

| Region | Cohen’s d | SE | CI LB | CI UB | P-value | FDR P-value | I^2^ | Controls | Patients |
| --- | --- | --- | --- | --- | --- | --- | --- | --- | --- |
| ACR | -0.061 | 0.090 | -0.238 | 0.116 | 0.497 | 0.592 | 0.000 | 816 | 169 |
| ALIC | -0.050 | 0.090 | -0.227 | 0.127 | 0.578 | 0.657 | 0.000 | 816 | 169 |
| BCC | -0.160 | 0.121 | -0.397 | 0.076 | 0.183 | 0.307 | 35.221 | 816 | 169 |
| CC | -0.125 | 0.112 | -0.344 | 0.094 | 0.264 | 0.388 | 26.085 | 816 | 169 |
| CGC | -0.199 | 0.091 | -0.377 | -0.021 | 0.029 | 0.215 | 0.007 | 816 | 169 |
| CGH | -0.184 | 0.090 | -0.362 | -0.007 | 0.042 | 0.215 | 0.000 | 816 | 169 |
| CR | -0.088 | 0.090 | -0.265 | 0.089 | 0.331 | 0.436 | 0.000 | 816 | 169 |
| CST | -0.122 | 0.090 | -0.299 | 0.055 | 0.178 | 0.307 | 0.000 | 816 | 169 |
| EC | -0.258 | 0.090 | -0.435 | -0.080 | 0.004 | 0.100 | 0.000 | 816 | 169 |
| FX | -0.159 | 0.091 | -0.336 | 0.018 | 0.079 | 0.267 | 0.001 | 816 | 169 |
| FXST | -0.176 | 0.106 | -0.383 | 0.031 | 0.096 | 0.267 | 19.728 | 816 | 169 |
| GCC | -0.066 | 0.090 | -0.243 | 0.111 | 0.464 | 0.580 | 0.002 | 816 | 169 |
| IC | -0.120 | 0.090 | -0.297 | 0.057 | 0.184 | 0.307 | 0.000 | 816 | 169 |
| IFO | -0.183 | 0.090 | -0.360 | -0.006 | 0.043 | 0.215 | 0.000 | 816 | 169 |
| PCR | -0.172 | 0.090 | -0.349 | 0.005 | 0.057 | 0.238 | 0.000 | 816 | 169 |
| PLIC | -0.088 | 0.090 | -0.265 | 0.089 | 0.328 | 0.436 | 0.000 | 816 | 169 |
| PTR | -0.127 | 0.091 | -0.305 | 0.050 | 0.160 | 0.307 | 0.012 | 816 | 169 |
| RLIC | -0.140 | 0.090 | -0.317 | 0.037 | 0.121 | 0.303 | 0.000 | 816 | 169 |
| SCC | -0.042 | 0.111 | -0.261 | 0.176 | 0.705 | 0.734 | 26.014 | 816 | 169 |
| SCR | -0.038 | 0.090 | -0.215 | 0.139 | 0.671 | 0.729 | 0.000 | 816 | 169 |
| SFO | -0.134 | 0.110 | -0.350 | 0.082 | 0.223 | 0.348 | 24.703 | 816 | 169 |
| SLF | -0.128 | 0.090 | -0.305 | 0.049 | 0.158 | 0.307 | 0.000 | 816 | 169 |
| SS | -0.208 | 0.090 | -0.386 | -0.031 | 0.021 | 0.215 | 0.000 | 816 | 169 |
| UNC | -0.008 | 0.195 | -0.390 | 0.373 | 0.966 | 0.966 | 74.464 | 816 | 169 |
| AverageFA | -0.154 | 0.090 | -0.331 | 0.023 | 0.089 | 0.267 | 0.000 | 816 | 169 |

Table S37. Cohen’s d effect sizes after meta-analysis, for AD differences between patients with first episode MDD and controls **in adults only**. Age, sex, agexsex, age^2^, age^2^xsex and scansite included as covariates.

| Region | Cohen’s d | SE | CI LB | CI UB | P-value | FDR P-value | I^2^ | Controls | Patients |
| --- | --- | --- | --- | --- | --- | --- | --- | --- | --- |
| ACR | 0.167 | 0.118 | -0.065 | 0.399 | 0.158 | 0.586 | 34.157 | 816 | 169 |
| ALIC | 0.088 | 0.112 | -0.132 | 0.308 | 0.434 | 0.723 | 27.674 | 816 | 169 |
| BCC | 0.128 | 0.091 | -0.049 | 0.306 | 0.156 | 0.586 | 0.000 | 816 | 169 |
| CC | 0.123 | 0.090 | -0.055 | 0.300 | 0.175 | 0.586 | 0.000 | 816 | 169 |
| CGC | 0.002 | 0.102 | -0.198 | 0.203 | 0.981 | 0.981 | 15.523 | 816 | 169 |
| CGH | 0.034 | 0.116 | -0.192 | 0.260 | 0.769 | 0.961 | 31.003 | 816 | 169 |
| CR | 0.208 | 0.111 | -0.009 | 0.424 | 0.060 | 0.500 | 25.774 | 816 | 169 |
| CST | 0.106 | 0.090 | -0.071 | 0.283 | 0.239 | 0.586 | 0.000 | 816 | 169 |
| EC | 0.108 | 0.095 | -0.077 | 0.294 | 0.253 | 0.586 | 6.054 | 816 | 169 |
| FX | 0.054 | 0.090 | -0.123 | 0.231 | 0.550 | 0.859 | 0.002 | 816 | 169 |
| FXST | -0.005 | 0.115 | -0.231 | 0.221 | 0.964 | 0.981 | 30.989 | 816 | 169 |
| GCC | 0.030 | 0.090 | -0.147 | 0.207 | 0.738 | 0.961 | 0.000 | 816 | 169 |
| IC | 0.134 | 0.145 | -0.150 | 0.418 | 0.356 | 0.668 | 54.836 | 816 | 169 |
| IFO | -0.013 | 0.090 | -0.190 | 0.164 | 0.887 | 0.981 | 0.000 | 816 | 169 |
| PCR | 0.132 | 0.124 | -0.111 | 0.374 | 0.287 | 0.598 | 38.967 | 816 | 169 |
| PLIC | 0.183 | 0.119 | -0.052 | 0.417 | 0.126 | 0.586 | 35.142 | 816 | 169 |
| PTR | 0.112 | 0.099 | -0.083 | 0.307 | 0.258 | 0.586 | 12.238 | 816 | 169 |
| RLIC | 0.063 | 0.187 | -0.303 | 0.430 | 0.734 | 0.961 | 72.616 | 816 | 169 |
| SCC | 0.112 | 0.090 | -0.066 | 0.289 | 0.217 | 0.586 | 0.000 | 816 | 169 |
| SCR | 0.232 | 0.095 | 0.047 | 0.418 | 0.014 | 0.338 | 6.054 | 816 | 169 |
| SFO | 0.007 | 0.090 | -0.170 | 0.184 | 0.937 | 0.981 | 0.000 | 816 | 169 |
| SLF | 0.083 | 0.093 | -0.100 | 0.265 | 0.374 | 0.668 | 3.823 | 816 | 169 |
| SS | 0.074 | 0.136 | -0.193 | 0.340 | 0.588 | 0.865 | 49.044 | 816 | 169 |
| UNC | -0.008 | 0.169 | -0.340 | 0.324 | 0.964 | 0.981 | 66.494 | 816 | 169 |
| AverageAD | 0.217 | 0.098 | 0.024 | 0.409 | 0.027 | 0.338 | 10.473 | 816 | 169 |

Table S38. Cohen’s d effect sizes after meta-analysis, for MD differences between patients with first episode MDD and controls **in adults only**. Age, sex, agexsex, age^2^, age^2^xsex and scansite included as covariates.

| Region | Cohen’s d | SE | CI LB | CI UB | P-value | FDR P-value | I^2^ | Controls | Patients |
| --- | --- | --- | --- | --- | --- | --- | --- | --- | --- |
| ACR | 0.054 | 0.090 | -0.123 | 0.231 | 0.551 | 0.676 | 0.000 | 816 | 169 |
| ALIC | 0.015 | 0.090 | -0.162 | 0.192 | 0.870 | 0.870 | 0.000 | 816 | 169 |
| BCC | 0.247 | 0.091 | 0.069 | 0.425 | 0.007 | 0.175 | 0.005 | 816 | 169 |
| CC | 0.178 | 0.091 | 0.000 | 0.355 | 0.050 | 0.290 | 0.007 | 816 | 169 |
| CGC | 0.162 | 0.090 | -0.015 | 0.339 | 0.073 | 0.304 | 0.000 | 816 | 169 |
| CGH | 0.058 | 0.101 | -0.139 | 0.256 | 0.563 | 0.676 | 13.843 | 816 | 169 |
| CR | 0.111 | 0.090 | -0.066 | 0.288 | 0.220 | 0.550 | 0.000 | 816 | 169 |
| CST | 0.089 | 0.090 | -0.088 | 0.266 | 0.326 | 0.627 | 0.000 | 816 | 169 |
| EC | 0.172 | 0.090 | -0.006 | 0.349 | 0.058 | 0.290 | 0.000 | 816 | 169 |
| FX | 0.065 | 0.090 | -0.113 | 0.242 | 0.474 | 0.676 | 0.003 | 816 | 169 |
| FXST | 0.162 | 0.127 | -0.087 | 0.410 | 0.202 | 0.550 | 41.149 | 816 | 169 |
| GCC | 0.065 | 0.090 | -0.112 | 0.242 | 0.471 | 0.676 | 0.000 | 816 | 169 |
| IC | 0.049 | 0.090 | -0.128 | 0.226 | 0.590 | 0.676 | 0.000 | 816 | 169 |
| IFO | 0.187 | 0.090 | 0.009 | 0.364 | 0.039 | 0.290 | 0.000 | 816 | 169 |
| PCR | 0.141 | 0.090 | -0.037 | 0.318 | 0.120 | 0.375 | 0.000 | 816 | 169 |
| PLIC | 0.067 | 0.090 | -0.110 | 0.244 | 0.455 | 0.676 | 0.000 | 816 | 169 |
| PTR | 0.048 | 0.090 | -0.129 | 0.225 | 0.595 | 0.676 | 0.000 | 816 | 169 |
| RLIC | 0.034 | 0.102 | -0.166 | 0.233 | 0.741 | 0.805 | 14.914 | 816 | 169 |
| SCC | 0.097 | 0.090 | -0.081 | 0.274 | 0.285 | 0.594 | 0.000 | 816 | 169 |
| SCR | 0.144 | 0.090 | -0.033 | 0.321 | 0.110 | 0.375 | 0.000 | 816 | 169 |
| SFO | 0.048 | 0.090 | -0.129 | 0.225 | 0.595 | 0.676 | 0.000 | 816 | 169 |
| SLF | 0.080 | 0.090 | -0.097 | 0.257 | 0.374 | 0.668 | 0.000 | 816 | 169 |
| SS | 0.104 | 0.090 | -0.073 | 0.281 | 0.251 | 0.570 | 0.000 | 816 | 169 |
| UNC | -0.046 | 0.178 | -0.395 | 0.302 | 0.794 | 0.827 | 69.421 | 816 | 169 |
| AverageMD | 0.176 | 0.091 | -0.001 | 0.354 | 0.052 | 0.290 | 0.000 | 816 | 169 |

Table S39. Cohen’s d effect sizes after meta-analysis, for RD differences between patients with first episode MDD and controls **in adults only**. Age, sex, agexsex, age^2^, age^2^xsex and scansite included as covariates.

| Region | Cohen’s d | SE | CI LB | CI UB | P-value | FDR P-value | I^2^ | Controls | Patients |
| --- | --- | --- | --- | --- | --- | --- | --- | --- | --- |
| ACR | 0.038 | 0.090 | -0.139 | 0.215 | 0.676 | 0.676 | 0.000 | 816 | 169 |
| ALIC | 0.038 | 0.090 | -0.139 | 0.215 | 0.676 | 0.676 | 0.000 | 816 | 169 |
| BCC | 0.198 | 0.091 | 0.020 | 0.375 | 0.029 | 0.146 | 0.001 | 816 | 169 |
| CC | 0.150 | 0.091 | -0.028 | 0.327 | 0.099 | 0.309 | 0.001 | 816 | 169 |
| CGC | 0.226 | 0.091 | 0.048 | 0.404 | 0.013 | 0.108 | 0.000 | 816 | 169 |
| CGH | 0.126 | 0.091 | -0.052 | 0.303 | 0.165 | 0.375 | 0.001 | 816 | 169 |
| CR | 0.113 | 0.090 | -0.064 | 0.290 | 0.212 | 0.408 | 0.000 | 816 | 169 |
| CST | 0.106 | 0.090 | -0.072 | 0.283 | 0.243 | 0.434 | 0.000 | 816 | 169 |
| EC | 0.226 | 0.091 | 0.049 | 0.403 | 0.013 | 0.108 | 0.000 | 816 | 169 |
| FX | 0.075 | 0.090 | -0.103 | 0.252 | 0.409 | 0.499 | 0.011 | 816 | 169 |
| FXST | 0.226 | 0.144 | -0.056 | 0.509 | 0.116 | 0.322 | 53.562 | 816 | 169 |
| GCC | 0.081 | 0.090 | -0.096 | 0.258 | 0.368 | 0.499 | 0.010 | 816 | 169 |
| IC | 0.078 | 0.090 | -0.099 | 0.255 | 0.389 | 0.499 | 0.000 | 816 | 169 |
| IFO | 0.252 | 0.091 | 0.074 | 0.429 | 0.005 | 0.108 | 0.000 | 816 | 169 |
| PCR | 0.175 | 0.090 | -0.002 | 0.353 | 0.052 | 0.186 | 0.000 | 816 | 169 |
| PLIC | 0.069 | 0.090 | -0.108 | 0.246 | 0.447 | 0.508 | 0.000 | 816 | 169 |
| PTR | 0.084 | 0.090 | -0.093 | 0.262 | 0.351 | 0.499 | 0.008 | 816 | 169 |
| RLIC | 0.073 | 0.090 | -0.104 | 0.250 | 0.419 | 0.499 | 0.000 | 816 | 169 |
| SCC | 0.123 | 0.120 | -0.113 | 0.359 | 0.307 | 0.499 | 35.065 | 816 | 169 |
| SCR | 0.133 | 0.090 | -0.044 | 0.310 | 0.141 | 0.353 | 0.000 | 816 | 169 |
| SFO | 0.089 | 0.090 | -0.088 | 0.266 | 0.326 | 0.499 | 0.000 | 816 | 169 |
| SLF | 0.118 | 0.090 | -0.059 | 0.296 | 0.190 | 0.396 | 0.000 | 816 | 169 |
| SS | 0.190 | 0.090 | 0.013 | 0.367 | 0.035 | 0.146 | 0.000 | 816 | 169 |
| UNC | -0.078 | 0.174 | -0.420 | 0.263 | 0.652 | 0.676 | 68.104 | 816 | 169 |
| AverageRD | 0.193 | 0.091 | 0.016 | 0.371 | 0.033 | 0.146 | 0.000 | 816 | 169 |

Table S40. Cohen’s d effect sizes after meta-analysis, for FA differences between patients with first episode MDD and patients with recurrent MDD **in adults only**. Age, sex, agexsex, age^2^, age^2^xsex and scansite included as covariates.

| Region | Cohen’s d | SE | CI LB | CI UB | P-value | FDR P-value | I^2^ | Controls | Patients |
| --- | --- | --- | --- | --- | --- | --- | --- | --- | --- |
| ACR | -0.159 | 0.106 | -0.366 | 0.049 | 0.135 | 0.841 | 0.000 | 135 | 332 |
| ALIC | -0.126 | 0.127 | -0.375 | 0.123 | 0.322 | 0.841 | 23.150 | 135 | 332 |
| BCC | -0.108 | 0.106 | -0.316 | 0.099 | 0.307 | 0.841 | 0.000 | 135 | 332 |
| CC | -0.159 | 0.106 | -0.367 | 0.049 | 0.134 | 0.841 | 0.000 | 135 | 332 |
| CGC | -0.020 | 0.106 | -0.228 | 0.187 | 0.847 | 0.882 | 0.000 | 135 | 332 |
| CGH | 0.227 | 0.237 | -0.238 | 0.691 | 0.339 | 0.841 | 76.314 | 135 | 332 |
| CR | -0.128 | 0.106 | -0.336 | 0.080 | 0.226 | 0.841 | 0.000 | 135 | 332 |
| CST | 0.126 | 0.185 | -0.236 | 0.489 | 0.495 | 0.841 | 61.582 | 135 | 332 |
| EC | 0.057 | 0.106 | -0.151 | 0.265 | 0.589 | 0.863 | 0.001 | 135 | 332 |
| FX | 0.065 | 0.106 | -0.142 | 0.273 | 0.538 | 0.841 | 0.000 | 135 | 332 |
| FXST | -0.059 | 0.173 | -0.398 | 0.280 | 0.734 | 0.863 | 56.081 | 135 | 332 |
| GCC | -0.184 | 0.106 | -0.392 | 0.024 | 0.083 | 0.841 | 0.000 | 135 | 332 |
| IC | -0.147 | 0.183 | -0.506 | 0.212 | 0.422 | 0.841 | 60.511 | 135 | 332 |
| IFO | -0.093 | 0.140 | -0.368 | 0.181 | 0.506 | 0.841 | 35.083 | 135 | 332 |
| PCR | -0.028 | 0.106 | -0.235 | 0.180 | 0.794 | 0.863 | 0.000 | 135 | 332 |
| PLIC | -0.029 | 0.106 | -0.237 | 0.179 | 0.786 | 0.863 | 0.005 | 135 | 332 |
| PTR | -0.034 | 0.106 | -0.242 | 0.173 | 0.746 | 0.863 | 0.000 | 135 | 332 |
| RLIC | -0.138 | 0.211 | -0.552 | 0.275 | 0.512 | 0.841 | 70.239 | 135 | 332 |
| SCC | -0.123 | 0.106 | -0.331 | 0.084 | 0.245 | 0.841 | 0.000 | 135 | 332 |
| SCR | -0.081 | 0.106 | -0.290 | 0.127 | 0.444 | 0.841 | 0.003 | 135 | 332 |
| SFO | -0.215 | 0.106 | -0.423 | -0.007 | 0.043 | 0.841 | 0.001 | 135 | 332 |
| SLF | -0.050 | 0.106 | -0.257 | 0.158 | 0.640 | 0.863 | 0.000 | 135 | 332 |
| SS | 0.006 | 0.132 | -0.253 | 0.266 | 0.962 | 0.962 | 28.595 | 135 | 332 |
| UNC | -0.031 | 0.106 | -0.239 | 0.177 | 0.769 | 0.863 | 0.013 | 135 | 332 |
| AverageFA | -0.093 | 0.106 | -0.301 | 0.115 | 0.380 | 0.841 | 0.000 | 135 | 332 |

Table S41. Cohen’s d effect sizes after meta-analysis, for AD differences between patients with first episode MDD and patients with recurrent MDD **in adults only**. Age, sex, agexsex, age^2^, age^2^xsex and scansite included as covariates.

| Region | Cohen’s d | SE | CI LB | CI UB | P-value | FDR P-value | I^2^ | Controls | Patients |
| --- | --- | --- | --- | --- | --- | --- | --- | --- | --- |
| ACR | -0.111 | 0.191 | -0.486 | 0.264 | 0.561 | 0.841 | 63.889 | 135 | 332 |
| ALIC | 0.069 | 0.160 | -0.245 | 0.383 | 0.666 | 0.841 | 48.989 | 135 | 332 |
| BCC | -0.071 | 0.106 | -0.279 | 0.136 | 0.500 | 0.841 | 0.000 | 135 | 332 |
| CC | -0.116 | 0.106 | -0.324 | 0.091 | 0.272 | 0.841 | 0.000 | 135 | 332 |
| CGC | -0.109 | 0.106 | -0.317 | 0.098 | 0.302 | 0.882 | 0.001 | 135 | 332 |
| CGH | 0.157 | 0.212 | -0.258 | 0.571 | 0.459 | 0.841 | 70.369 | 135 | 332 |
| CR | -0.133 | 0.178 | -0.482 | 0.215 | 0.454 | 0.841 | 58.435 | 135 | 332 |
| CST | 0.072 | 0.198 | -0.316 | 0.460 | 0.716 | 0.841 | 66.345 | 135 | 332 |
| EC | 0.088 | 0.160 | -0.226 | 0.402 | 0.584 | 0.863 | 48.962 | 135 | 332 |
| FX | -0.021 | 0.106 | -0.228 | 0.186 | 0.843 | 0.841 | 0.000 | 135 | 332 |
| FXST | 0.031 | 0.197 | -0.355 | 0.417 | 0.875 | 0.863 | 66.096 | 135 | 332 |
| GCC | -0.061 | 0.106 | -0.269 | 0.147 | 0.564 | 0.841 | 0.006 | 135 | 332 |
| IC | -0.019 | 0.152 | -0.316 | 0.278 | 0.899 | 0.841 | 43.793 | 135 | 332 |
| IFO | 0.107 | 0.106 | -0.100 | 0.315 | 0.310 | 0.841 | 0.000 | 135 | 332 |
| PCR | -0.165 | 0.215 | -0.587 | 0.256 | 0.442 | 0.863 | 71.553 | 135 | 332 |
| PLIC | -0.061 | 0.106 | -0.269 | 0.147 | 0.566 | 0.863 | 0.006 | 135 | 332 |
| PTR | -0.060 | 0.220 | -0.490 | 0.371 | 0.786 | 0.863 | 72.731 | 135 | 332 |
| RLIC | -0.084 | 0.150 | -0.378 | 0.210 | 0.574 | 0.841 | 42.997 | 135 | 332 |
| SCC | -0.189 | 0.106 | -0.397 | 0.019 | 0.074 | 0.841 | 0.000 | 135 | 332 |
| SCR | -0.106 | 0.106 | -0.314 | 0.101 | 0.316 | 0.841 | 0.000 | 135 | 332 |
| SFO | -0.084 | 0.106 | -0.292 | 0.124 | 0.427 | 0.841 | 0.000 | 135 | 332 |
| SLF | -0.009 | 0.196 | -0.393 | 0.376 | 0.965 | 0.863 | 65.794 | 135 | 332 |
| SS | -0.017 | 0.189 | -0.388 | 0.353 | 0.927 | 0.962 | 63.429 | 135 | 332 |
| UNC | 0.054 | 0.229 | -0.395 | 0.503 | 0.813 | 0.863 | 74.751 | 135 | 332 |
| AverageAD | -0.063 | 0.146 | -0.348 | 0.223 | 0.666 | 0.841 | 39.358 | 135 | 332 |

Table S42. Cohen’s d effect sizes after meta-analysis, for MD differences between patients with first episode MDD and patients with recurrent MDD **in adults only**. Age, sex, agexsex, age^2^, age^2^xsex and scansite included as covariates.

| Region | Cohen’s d | SE | CI LB | CI UB | P-value | FDR P-value | I^2^ | Controls | Patients |
| --- | --- | --- | --- | --- | --- | --- | --- | --- | --- |
| ACR | 0.051 | 0.106 | -0.158 | 0.259 | 0.634 | 0.865 | 0.003 | 135 | 332 |
| ALIC | 0.147 | 0.106 | -0.062 | 0.355 | 0.167 | 0.865 | 0.006 | 135 | 332 |
| BCC | -0.075 | 0.106 | -0.283 | 0.132 | 0.477 | 0.865 | 0.000 | 135 | 332 |
| CC | -0.065 | 0.106 | -0.272 | 0.143 | 0.541 | 0.865 | 0.000 | 135 | 332 |
| CGC | -0.106 | 0.106 | -0.314 | 0.102 | 0.316 | 0.865 | 0.001 | 135 | 332 |
| CGH | 0.082 | 0.155 | -0.222 | 0.387 | 0.596 | 0.865 | 46.374 | 135 | 332 |
| CR | 0.074 | 0.106 | -0.134 | 0.282 | 0.485 | 0.865 | 0.014 | 135 | 332 |
| CST | 0.072 | 0.205 | -0.331 | 0.474 | 0.727 | 0.865 | 68.782 | 135 | 332 |
| EC | 0.081 | 0.140 | -0.193 | 0.355 | 0.562 | 0.865 | 34.316 | 135 | 332 |
| FX | -0.062 | 0.106 | -0.269 | 0.146 | 0.559 | 0.865 | 0.000 | 135 | 332 |
| FXST | 0.158 | 0.145 | -0.126 | 0.443 | 0.276 | 0.865 | 38.983 | 135 | 332 |
| GCC | -0.010 | 0.106 | -0.218 | 0.197 | 0.922 | 0.931 | 0.001 | 135 | 332 |
| IC | 0.042 | 0.106 | -0.166 | 0.250 | 0.691 | 0.865 | 0.002 | 135 | 332 |
| IFO | -0.059 | 0.128 | -0.310 | 0.191 | 0.641 | 0.865 | 24.083 | 135 | 332 |
| PCR | 0.042 | 0.106 | -0.165 | 0.250 | 0.690 | 0.865 | 0.001 | 135 | 332 |
| PLIC | -0.026 | 0.106 | -0.234 | 0.182 | 0.805 | 0.915 | 0.001 | 135 | 332 |
| PTR | 0.118 | 0.128 | -0.133 | 0.370 | 0.357 | 0.865 | 24.505 | 135 | 332 |
| RLIC | 0.019 | 0.106 | -0.190 | 0.227 | 0.861 | 0.931 | 0.007 | 135 | 332 |
| SCC | -0.074 | 0.106 | -0.281 | 0.133 | 0.484 | 0.865 | 0.000 | 135 | 332 |
| SCR | 0.127 | 0.106 | -0.081 | 0.335 | 0.233 | 0.865 | 0.001 | 135 | 332 |
| SFO | 0.089 | 0.112 | -0.130 | 0.307 | 0.427 | 0.865 | 6.339 | 135 | 332 |
| SLF | 0.104 | 0.145 | -0.180 | 0.387 | 0.474 | 0.865 | 38.426 | 135 | 332 |
| SS | 0.056 | 0.146 | -0.230 | 0.343 | 0.700 | 0.865 | 39.321 | 135 | 332 |
| UNC | 0.098 | 0.106 | -0.110 | 0.306 | 0.355 | 0.865 | 0.000 | 135 | 332 |
| AverageMD | 0.009 | 0.106 | -0.199 | 0.217 | 0.931 | 0.931 | 0.001 | 135 | 332 |

Table S43. Cohen’s d effect sizes after meta-analysis, for RD differences between patients with first episode MDD and patients with recurrent MDD **in adults only**. Age, sex, agexsex, age^2^, age^2^xsex and scansite included as covariates.

| Region | Cohen’s d | SE | CI LB | CI UB | P-value | FDR P-value | I^2^ | Controls | Patients |
| --- | --- | --- | --- | --- | --- | --- | --- | --- | --- |
| ACR | 0.106 | 0.106 | -0.103 | 0.314 | 0.320 | 0.965 | 0.000 | 135 | 332 |
| ALIC | 0.172 | 0.106 | -0.037 | 0.380 | 0.106 | 0.965 | 0.002 | 135 | 332 |
| BCC | -0.046 | 0.106 | -0.253 | 0.162 | 0.665 | 0.965 | 0.000 | 135 | 332 |
| CC | -0.027 | 0.106 | -0.235 | 0.180 | 0.798 | 0.965 | 0.000 | 135 | 332 |
| CGC | -0.100 | 0.106 | -0.307 | 0.108 | 0.346 | 0.965 | 0.000 | 135 | 332 |
| CGH | -0.051 | 0.148 | -0.341 | 0.238 | 0.728 | 0.965 | 40.922 | 135 | 332 |
| CR | 0.112 | 0.106 | -0.097 | 0.320 | 0.293 | 0.965 | 0.006 | 135 | 332 |
| CST | 0.066 | 0.210 | -0.345 | 0.477 | 0.752 | 0.965 | 70.086 | 135 | 332 |
| EC | 0.031 | 0.155 | -0.272 | 0.335 | 0.839 | 0.965 | 45.527 | 135 | 332 |
| FX | -0.070 | 0.106 | -0.278 | 0.137 | 0.506 | 0.965 | 0.000 | 135 | 332 |
| FXST | 0.143 | 0.126 | -0.104 | 0.391 | 0.256 | 0.965 | 22.286 | 135 | 332 |
| GCC | 0.020 | 0.106 | -0.188 | 0.228 | 0.849 | 0.965 | 0.004 | 135 | 332 |
| IC | 0.105 | 0.149 | -0.186 | 0.397 | 0.478 | 0.965 | 41.160 | 135 | 332 |
| IFO | -0.107 | 0.106 | -0.315 | 0.101 | 0.315 | 0.965 | 0.005 | 135 | 332 |
| PCR | 0.042 | 0.106 | -0.166 | 0.250 | 0.692 | 0.965 | 0.002 | 135 | 332 |
| PLIC | 0.000 | 0.106 | -0.208 | 0.208 | 1.000 | 1.000 | 0.001 | 135 | 332 |
| PTR | 0.034 | 0.106 | -0.174 | 0.242 | 0.751 | 0.965 | 0.003 | 135 | 332 |
| RLIC | 0.124 | 0.221 | -0.310 | 0.558 | 0.575 | 0.965 | 72.913 | 135 | 332 |
| SCC | -0.007 | 0.106 | -0.214 | 0.201 | 0.950 | 0.990 | 0.000 | 135 | 332 |
| SCR | 0.220 | 0.162 | -0.098 | 0.538 | 0.176 | 0.965 | 49.824 | 135 | 332 |
| SFO | 0.115 | 0.156 | -0.192 | 0.421 | 0.463 | 0.965 | 46.582 | 135 | 332 |
| SLF | 0.031 | 0.106 | -0.177 | 0.239 | 0.773 | 0.965 | 0.003 | 135 | 332 |
| SS | -0.032 | 0.165 | -0.355 | 0.290 | 0.844 | 0.965 | 51.472 | 135 | 332 |
| UNC | 0.102 | 0.106 | -0.105 | 0.309 | 0.334 | 0.965 | 0.000 | 135 | 332 |
| AverageRD | 0.008 | 0.106 | -0.200 | 0.215 | 0.941 | 0.990 | 0.006 | 135 | 332 |

Table S44. Cohen’s d effect sizes after meta-analysis, for FA differences between patients with recurrent MDD and controls **in adolescents only**. Age, sex, agexsex, age^2^, age^2^xsex and scansite included as covariates.

| Region | Cohen’s d | SE | CI LB | CI UB | P-value | FDR P-value | I^2^ | Controls | Patients |
| --- | --- | --- | --- | --- | --- | --- | --- | --- | --- |
| ACR | -0.132 | 0.156 | -0.439 | 0.174 | 0.398 | 0.805 | 38.193 | 146 | 148 |
| ALIC | -0.130 | 0.120 | -0.365 | 0.106 | 0.280 | 0.805 | 0.000 | 146 | 148 |
| BCC | -0.292 | 0.120 | -0.528 | -0.056 | 0.015 | 0.350 | 0.000 | 146 | 148 |
| CC | -0.263 | 0.120 | -0.499 | -0.028 | 0.028 | 0.350 | 0.000 | 146 | 148 |
| CGC | 0.113 | 0.186 | -0.252 | 0.479 | 0.543 | 0.805 | 55.910 | 146 | 148 |
| CGH | -0.082 | 0.167 | -0.409 | 0.244 | 0.621 | 0.805 | 45.375 | 146 | 148 |
| CR | -0.133 | 0.152 | -0.431 | 0.165 | 0.381 | 0.805 | 34.869 | 146 | 148 |
| CST | -0.058 | 0.125 | -0.303 | 0.187 | 0.644 | 0.805 | 7.063 | 146 | 148 |
| EC | 0.112 | 0.188 | -0.257 | 0.481 | 0.552 | 0.805 | 56.756 | 146 | 148 |
| FX | -0.111 | 0.204 | -0.511 | 0.289 | 0.586 | 0.805 | 62.889 | 146 | 148 |
| FXST | -0.127 | 0.130 | -0.383 | 0.128 | 0.328 | 0.805 | 13.253 | 146 | 148 |
| GCC | -0.240 | 0.159 | -0.552 | 0.072 | 0.132 | 0.805 | 39.926 | 146 | 148 |
| IC | -0.008 | 0.137 | -0.276 | 0.260 | 0.953 | 0.953 | 20.548 | 146 | 148 |
| IFO | -0.239 | 0.192 | -0.615 | 0.138 | 0.214 | 0.805 | 58.139 | 146 | 148 |
| PCR | -0.021 | 0.120 | -0.255 | 0.214 | 0.862 | 0.898 | 0.000 | 146 | 148 |
| PLIC | 0.068 | 0.133 | -0.192 | 0.327 | 0.610 | 0.805 | 15.923 | 146 | 148 |
| PTR | 0.030 | 0.120 | -0.206 | 0.265 | 0.805 | 0.898 | 0.006 | 146 | 148 |
| RLIC | 0.025 | 0.125 | -0.221 | 0.271 | 0.843 | 0.898 | 7.493 | 146 | 148 |
| SCC | -0.095 | 0.120 | -0.330 | 0.140 | 0.429 | 0.805 | 0.000 | 146 | 148 |
| SCR | -0.166 | 0.142 | -0.445 | 0.113 | 0.243 | 0.805 | 26.166 | 146 | 148 |
| SFO | -0.092 | 0.120 | -0.327 | 0.144 | 0.445 | 0.805 | 0.000 | 146 | 148 |
| SLF | -0.032 | 0.179 | -0.382 | 0.318 | 0.857 | 0.898 | 52.036 | 146 | 148 |
| SS | -0.096 | 0.165 | -0.420 | 0.228 | 0.563 | 0.805 | 44.435 | 146 | 148 |
| UNC | 0.097 | 0.134 | -0.166 | 0.359 | 0.471 | 0.805 | 17.475 | 146 | 148 |
| AverageFA | -0.185 | 0.160 | -0.498 | 0.128 | 0.247 | 0.805 | 40.650 | 146 | 148 |

Table S45. Cohen’s d effect sizes after meta-analysis, for AD differences between patients with recurrent MDD and controls **in adolescents only**. Age, sex, agexsex, age^2^, age^2^xsex and scansite included as covariates.

| Region | Cohen’s d | SE | CI LB | CI UB | P-value | FDR P-value | I^2^ | Controls | Patients |
| --- | --- | --- | --- | --- | --- | --- | --- | --- | --- |
| ACR | 0.026 | 0.121 | -0.211 | 0.263 | 0.828 | 0.973 | 1.562 | 146 | 148 |
| ALIC | 0.048 | 0.120 | -0.187 | 0.282 | 0.692 | 0.973 | 0.000 | 146 | 148 |
| BCC | -0.178 | 0.150 | -0.472 | 0.116 | 0.235 | 0.653 | 32.896 | 146 | 148 |
| CC | -0.095 | 0.131 | -0.351 | 0.162 | 0.470 | 0.925 | 13.945 | 146 | 148 |
| CGC | 0.254 | 0.120 | 0.018 | 0.490 | 0.035 | 0.653 | 0.000 | 146 | 148 |
| CGH | -0.004 | 0.122 | -0.244 | 0.235 | 0.974 | 0.974 | 3.241 | 146 | 148 |
| CR | 0.048 | 0.120 | -0.187 | 0.284 | 0.686 | 0.973 | 0.005 | 146 | 148 |
| CST | 0.074 | 0.120 | -0.161 | 0.309 | 0.538 | 0.961 | 0.004 | 146 | 148 |
| EC | 0.208 | 0.120 | -0.028 | 0.443 | 0.085 | 0.653 | 0.008 | 146 | 148 |
| FX | -0.007 | 0.120 | -0.242 | 0.228 | 0.955 | 0.974 | 0.005 | 146 | 148 |
| FXST | 0.188 | 0.120 | -0.047 | 0.424 | 0.117 | 0.653 | 0.001 | 146 | 148 |
| GCC | 0.023 | 0.120 | -0.212 | 0.258 | 0.849 | 0.973 | 0.000 | 146 | 148 |
| IC | 0.027 | 0.148 | -0.264 | 0.317 | 0.856 | 0.973 | 31.443 | 146 | 148 |
| IFO | 0.161 | 0.120 | -0.074 | 0.396 | 0.180 | 0.653 | 0.000 | 146 | 148 |
| PCR | 0.151 | 0.171 | -0.184 | 0.485 | 0.377 | 0.857 | 47.608 | 146 | 148 |
| PLIC | -0.088 | 0.185 | -0.449 | 0.274 | 0.635 | 0.973 | 54.952 | 146 | 148 |
| PTR | 0.115 | 0.163 | -0.205 | 0.434 | 0.481 | 0.925 | 42.767 | 146 | 148 |
| RLIC | 0.147 | 0.120 | -0.089 | 0.382 | 0.222 | 0.653 | 0.000 | 146 | 148 |
| SCC | -0.026 | 0.120 | -0.261 | 0.209 | 0.827 | 0.973 | 0.000 | 146 | 148 |
| SCR | -0.043 | 0.120 | -0.278 | 0.193 | 0.723 | 0.973 | 0.001 | 146 | 148 |
| SFO | 0.178 | 0.120 | -0.058 | 0.413 | 0.139 | 0.653 | 0.000 | 146 | 148 |
| SLF | 0.152 | 0.120 | -0.083 | 0.388 | 0.205 | 0.653 | 0.001 | 146 | 148 |
| SS | 0.197 | 0.120 | -0.038 | 0.432 | 0.100 | 0.653 | 0.000 | 146 | 148 |
| UNC | 0.176 | 0.175 | -0.168 | 0.520 | 0.317 | 0.793 | 50.232 | 146 | 148 |
| AverageAD | 0.020 | 0.160 | -0.293 | 0.334 | 0.898 | 0.974 | 40.568 | 146 | 148 |

Table S46. Cohen’s d effect sizes after meta-analysis, for MD differences between patients with recurrent MDD and controls **in adolescents only**. Age, sex, agexsex, age^2^, age^2^xsex and scansite included as covariates.

| Region | Cohen’s d | SE | CI LB | CI UB | P-value | FDR P-value | I^2^ | Controls | Patients |
| --- | --- | --- | --- | --- | --- | --- | --- | --- | --- |
| ACR | 0.091 | 0.207 | -0.314 | 0.497 | 0.659 | 0.805 | 64.038 | 146 | 148 |
| ALIC | 0.207 | 0.120 | -0.029 | 0.443 | 0.086 | 0.707 | 0.004 | 146 | 148 |
| BCC | 0.125 | 0.120 | -0.109 | 0.360 | 0.295 | 0.707 | 0.000 | 146 | 148 |
| CC | 0.148 | 0.137 | -0.120 | 0.416 | 0.278 | 0.707 | 20.447 | 146 | 148 |
| CGC | 0.251 | 0.178 | -0.097 | 0.599 | 0.157 | 0.707 | 51.316 | 146 | 148 |
| CGH | 0.100 | 0.200 | -0.293 | 0.492 | 0.619 | 0.805 | 61.758 | 146 | 148 |
| CR | 0.073 | 0.207 | -0.334 | 0.479 | 0.725 | 0.805 | 64.195 | 146 | 148 |
| CST | 0.124 | 0.122 | -0.116 | 0.363 | 0.311 | 0.707 | 2.908 | 146 | 148 |
| EC | 0.088 | 0.228 | -0.358 | 0.535 | 0.699 | 0.805 | 70.167 | 146 | 148 |
| FX | 0.093 | 0.120 | -0.142 | 0.328 | 0.437 | 0.805 | 0.000 | 146 | 148 |
| FXST | 0.316 | 0.121 | 0.079 | 0.552 | 0.009 | 0.225 | 0.000 | 146 | 148 |
| GCC | 0.194 | 0.156 | -0.112 | 0.501 | 0.213 | 0.707 | 37.911 | 146 | 148 |
| IC | 0.137 | 0.120 | -0.098 | 0.373 | 0.254 | 0.707 | 0.005 | 146 | 148 |
| IFO | 0.360 | 0.203 | -0.038 | 0.758 | 0.076 | 0.707 | 62.213 | 146 | 148 |
| PCR | 0.074 | 0.176 | -0.271 | 0.419 | 0.673 | 0.805 | 50.744 | 146 | 148 |
| PLIC | -0.005 | 0.120 | -0.241 | 0.230 | 0.964 | 0.964 | 0.000 | 146 | 148 |
| PTR | 0.080 | 0.213 | -0.337 | 0.498 | 0.707 | 0.805 | 66.011 | 146 | 148 |
| RLIC | 0.119 | 0.156 | -0.187 | 0.425 | 0.447 | 0.805 | 38.042 | 146 | 148 |
| SCC | 0.107 | 0.155 | -0.197 | 0.411 | 0.490 | 0.805 | 37.075 | 146 | 148 |
| SCR | 0.046 | 0.211 | -0.368 | 0.460 | 0.827 | 0.861 | 65.404 | 146 | 148 |
| SFO | 0.200 | 0.167 | -0.128 | 0.527 | 0.232 | 0.707 | 45.413 | 146 | 148 |
| SLF | 0.095 | 0.221 | -0.339 | 0.529 | 0.669 | 0.805 | 68.391 | 146 | 148 |
| SS | 0.224 | 0.170 | -0.109 | 0.557 | 0.187 | 0.707 | 47.065 | 146 | 148 |
| UNC | 0.064 | 0.194 | -0.317 | 0.445 | 0.741 | 0.805 | 59.445 | 146 | 148 |
| AverageMD | 0.147 | 0.208 | -0.260 | 0.554 | 0.479 | 0.805 | 64.243 | 146 | 148 |

Table S47. Cohen’s d effect sizes after meta-analysis, for RD differences between patients with recurrent MDD and controls **in adolescents only**. Age, sex, agexsex, age^2^, age^2^xsex and scansite included as covariates.

| Region | Cohen’s d | SE | CI LB | CI UB | P-value | FDR P-value | I^2^ | Controls | Patients |
| --- | --- | --- | --- | --- | --- | --- | --- | --- | --- |
| ACR | 0.119 | 0.225 | -0.322 | 0.559 | 0.597 | 0.811 | 69.420 | 146 | 148 |
| ALIC | 0.264 | 0.135 | 0.000 | 0.528 | 0.050 | 0.319 | 17.829 | 146 | 148 |
| BCC | 0.234 | 0.120 | -0.001 | 0.469 | 0.051 | 0.319 | 0.000 | 146 | 148 |
| CC | 0.273 | 0.120 | 0.037 | 0.509 | 0.023 | 0.319 | 0.000 | 146 | 148 |
| CGC | 0.096 | 0.257 | -0.406 | 0.599 | 0.707 | 0.842 | 76.398 | 146 | 148 |
| CGH | 0.146 | 0.253 | -0.349 | 0.641 | 0.563 | 0.811 | 75.685 | 146 | 148 |
| CR | 0.111 | 0.225 | -0.331 | 0.553 | 0.621 | 0.811 | 69.608 | 146 | 148 |
| CST | 0.169 | 0.133 | -0.091 | 0.430 | 0.203 | 0.633 | 16.338 | 146 | 148 |
| EC | 0.072 | 0.254 | -0.426 | 0.570 | 0.776 | 0.861 | 75.979 | 146 | 148 |
| FX | 0.137 | 0.120 | -0.098 | 0.372 | 0.253 | 0.633 | 0.000 | 146 | 148 |
| FXST | 0.305 | 0.141 | 0.029 | 0.581 | 0.030 | 0.319 | 24.102 | 146 | 148 |
| GCC | 0.277 | 0.163 | -0.042 | 0.595 | 0.089 | 0.445 | 42.294 | 146 | 148 |
| IC | 0.165 | 0.139 | -0.108 | 0.437 | 0.237 | 0.633 | 22.769 | 146 | 148 |
| IFO | 0.285 | 0.237 | -0.180 | 0.749 | 0.230 | 0.633 | 72.181 | 146 | 148 |
| PCR | 0.072 | 0.157 | -0.237 | 0.380 | 0.649 | 0.811 | 38.831 | 146 | 148 |
| PLIC | 0.018 | 0.120 | -0.217 | 0.253 | 0.881 | 0.896 | 0.007 | 146 | 148 |
| PTR | 0.100 | 0.199 | -0.289 | 0.490 | 0.614 | 0.811 | 61.080 | 146 | 148 |
| RLIC | 0.093 | 0.162 | -0.225 | 0.411 | 0.567 | 0.811 | 42.565 | 146 | 148 |
| SCC | 0.183 | 0.142 | -0.095 | 0.461 | 0.197 | 0.633 | 25.687 | 146 | 148 |
| SCR | 0.144 | 0.220 | -0.288 | 0.576 | 0.513 | 0.811 | 68.193 | 146 | 148 |
| SFO | 0.128 | 0.207 | -0.277 | 0.533 | 0.536 | 0.811 | 63.915 | 146 | 148 |
| SLF | 0.063 | 0.237 | -0.402 | 0.527 | 0.792 | 0.861 | 72.425 | 146 | 148 |
| SS | 0.187 | 0.211 | -0.227 | 0.602 | 0.375 | 0.781 | 65.449 | 146 | 148 |
| UNC | -0.022 | 0.172 | -0.359 | 0.314 | 0.896 | 0.896 | 48.282 | 146 | 148 |
| AverageRD | 0.226 | 0.221 | -0.207 | 0.659 | 0.306 | 0.695 | 68.177 | 146 | 148 |

Table S48. Cohen’s d effect sizes after meta-analysis, for FA differences between patients with first episode MDD and controls **in adolescents only**. Age, sex, agexsex, age^2^, age^2^xsex and scansite included as covariates.

| Region | Cohen’s d | SE | CI LB | CI UB | P-value | FDR P-value | I^2^ | Controls | Patients |
| --- | --- | --- | --- | --- | --- | --- | --- | --- | --- |
| ACR | 0.000 | 0.350 | -0.686 | 0.687 | 1.000 | 1.000 | 83.167 | 130 | 98 |
| ALIC | -0.018 | 0.430 | -0.860 | 0.825 | 0.967 | 1.000 | 88.589 | 130 | 98 |
| BCC | -0.234 | 0.140 | -0.508 | 0.040 | 0.095 | 0.788 | 0.000 | 130 | 98 |
| CC | -0.214 | 0.140 | -0.488 | 0.060 | 0.126 | 0.788 | 0.000 | 130 | 98 |
| CGC | 0.244 | 0.251 | -0.248 | 0.735 | 0.331 | 0.798 | 67.545 | 130 | 98 |
| CGH | 0.176 | 0.300 | -0.413 | 0.764 | 0.559 | 0.822 | 77.162 | 130 | 98 |
| CR | -0.020 | 0.338 | -0.682 | 0.642 | 0.952 | 1.000 | 81.942 | 130 | 98 |
| CST | -0.113 | 0.174 | -0.453 | 0.227 | 0.516 | 0.822 | 33.747 | 130 | 98 |
| EC | 0.435 | 0.348 | -0.248 | 1.117 | 0.212 | 0.798 | 82.571 | 130 | 98 |
| FX | 0.108 | 0.172 | -0.229 | 0.445 | 0.528 | 0.822 | 32.511 | 130 | 98 |
| FXST | 0.255 | 0.238 | -0.211 | 0.721 | 0.283 | 0.798 | 63.771 | 130 | 98 |
| GCC | -0.101 | 0.201 | -0.494 | 0.292 | 0.615 | 0.854 | 50.092 | 130 | 98 |
| IC | 0.193 | 0.228 | -0.254 | 0.639 | 0.398 | 0.798 | 60.799 | 130 | 98 |
| IFO | 0.011 | 0.198 | -0.378 | 0.400 | 0.955 | 1.000 | 48.998 | 130 | 98 |
| PCR | -0.144 | 0.172 | -0.482 | 0.193 | 0.403 | 0.798 | 32.686 | 130 | 98 |
| PLIC | 0.217 | 0.140 | -0.057 | 0.491 | 0.121 | 0.788 | 0.000 | 130 | 98 |
| PTR | 0.155 | 0.221 | -0.278 | 0.589 | 0.482 | 0.822 | 58.442 | 130 | 98 |
| RLIC | 0.134 | 0.139 | -0.139 | 0.408 | 0.335 | 0.798 | 0.000 | 130 | 98 |
| SCC | -0.142 | 0.140 | -0.415 | 0.132 | 0.310 | 0.798 | 0.000 | 130 | 98 |
| SCR | -0.009 | 0.316 | -0.629 | 0.611 | 0.978 | 1.000 | 79.497 | 130 | 98 |
| SFO | -0.074 | 0.235 | -0.535 | 0.387 | 0.752 | 0.989 | 63.372 | 130 | 98 |
| SLF | 0.114 | 0.140 | -0.160 | 0.387 | 0.415 | 0.798 | 0.000 | 130 | 98 |
| SS | 0.189 | 0.140 | -0.085 | 0.463 | 0.176 | 0.798 | 0.009 | 130 | 98 |
| UNC | -0.261 | 0.140 | -0.536 | 0.013 | 0.062 | 0.788 | 0.000 | 130 | 98 |
| AverageFA | 0.051 | 0.307 | -0.551 | 0.653 | 0.867 | 1.000 | 78.282 | 130 | 98 |

Table S49. Cohen’s d effect sizes after meta-analysis, for AD differences between patients with first episode MDD and controls **in adolescents only**. Age, sex, agexsex, age^2^, age^2^xsex and scansite included as covariates.

| Region | Cohen’s d | SE | CI LB | CI UB | P-value | FDR P-value | I^2^ | Controls | Patients |
| --- | --- | --- | --- | --- | --- | --- | --- | --- | --- |
| ACR | -0.004 | 0.172 | -0.342 | 0.334 | 0.980 | 0.980 | 33.068 | 130 | 98 |
| ALIC | 0.033 | 0.292 | -0.539 | 0.605 | 0.909 | 0.947 | 75.919 | 130 | 98 |
| BCC | -0.122 | 0.168 | -0.450 | 0.207 | 0.467 | 0.929 | 29.088 | 130 | 98 |
| CC | -0.059 | 0.139 | -0.332 | 0.214 | 0.673 | 0.947 | 0.000 | 130 | 98 |
| CGC | 0.366 | 0.144 | 0.084 | 0.649 | 0.011 | 0.138 | 4.392 | 130 | 98 |
| CGH | -0.027 | 0.139 | -0.300 | 0.246 | 0.844 | 0.947 | 0.000 | 130 | 98 |
| CR | 0.108 | 0.168 | -0.221 | 0.437 | 0.520 | 0.929 | 29.439 | 130 | 98 |
| CST | -0.335 | 0.332 | -0.985 | 0.316 | 0.314 | 0.782 | 81.135 | 130 | 98 |
| EC | 0.219 | 0.140 | -0.055 | 0.493 | 0.118 | 0.450 | 0.000 | 130 | 98 |
| FX | 0.345 | 0.209 | -0.065 | 0.755 | 0.099 | 0.450 | 52.960 | 130 | 98 |
| FXST | 0.197 | 0.140 | -0.077 | 0.471 | 0.158 | 0.494 | 0.000 | 130 | 98 |
| GCC | 0.056 | 0.139 | -0.217 | 0.329 | 0.687 | 0.947 | 0.000 | 130 | 98 |
| IC | 0.166 | 0.167 | -0.162 | 0.494 | 0.321 | 0.782 | 28.511 | 130 | 98 |
| IFO | 0.036 | 0.152 | -0.263 | 0.335 | 0.812 | 0.947 | 15.217 | 130 | 98 |
| PCR | 0.237 | 0.140 | -0.037 | 0.511 | 0.090 | 0.450 | 0.000 | 130 | 98 |
| PLIC | -0.039 | 0.155 | -0.344 | 0.265 | 0.802 | 0.947 | 18.222 | 130 | 98 |
| PTR | 0.369 | 0.142 | 0.090 | 0.647 | 0.010 | 0.138 | 1.562 | 130 | 98 |
| RLIC | 0.277 | 0.140 | 0.002 | 0.551 | 0.048 | 0.400 | 0.000 | 130 | 98 |
| SCC | -0.056 | 0.139 | -0.328 | 0.217 | 0.690 | 0.947 | 0.000 | 130 | 98 |
| SCR | 0.067 | 0.172 | -0.269 | 0.404 | 0.694 | 0.947 | 32.259 | 130 | 98 |
| SFO | -0.016 | 0.139 | -0.290 | 0.257 | 0.906 | 0.947 | 0.000 | 130 | 98 |
| SLF | 0.213 | 0.140 | -0.060 | 0.487 | 0.126 | 0.450 | 0.000 | 130 | 98 |
| SS | 0.132 | 0.139 | -0.141 | 0.405 | 0.344 | 0.782 | 0.000 | 130 | 98 |
| UNC | 0.048 | 0.139 | -0.224 | 0.321 | 0.728 | 0.947 | 0.000 | 130 | 98 |
| AverageAD | 0.102 | 0.150 | -0.192 | 0.396 | 0.495 | 0.929 | 12.085 | 130 | 98 |

Table S50. Cohen’s d effect sizes after meta-analysis, for MD differences between patients with first episode MDD and controls **in adolescents only**. Age, sex, agexsex, age^2^, age^2^xsex and scansite included as covariates.

| Region | Cohen’s d | SE | CI LB | CI UB | P-value | FDR P-value | I^2^ | Controls | Patients |
| --- | --- | --- | --- | --- | --- | --- | --- | --- | --- |
| ACR | 0.005 | 0.139 | -0.268 | 0.278 | 0.970 | 0.970 | 0.000 | 130 | 98 |
| ALIC | -0.046 | 0.139 | -0.319 | 0.226 | 0.739 | 0.970 | 0.000 | 130 | 98 |
| BCC | 0.106 | 0.183 | -0.252 | 0.465 | 0.561 | 0.955 | 40.125 | 130 | 98 |
| CC | 0.084 | 0.140 | -0.189 | 0.358 | 0.546 | 0.955 | 0.005 | 130 | 98 |
| CGC | 0.158 | 0.167 | -0.169 | 0.486 | 0.343 | 0.955 | 28.554 | 130 | 98 |
| CGH | -0.127 | 0.229 | -0.575 | 0.321 | 0.579 | 0.955 | 61.238 | 130 | 98 |
| CR | 0.071 | 0.139 | -0.202 | 0.344 | 0.611 | 0.955 | 0.000 | 130 | 98 |
| CST | -0.146 | 0.257 | -0.649 | 0.357 | 0.569 | 0.955 | 69.085 | 130 | 98 |
| EC | -0.021 | 0.175 | -0.365 | 0.322 | 0.903 | 0.970 | 35.095 | 130 | 98 |
| FX | 0.314 | 0.203 | -0.084 | 0.712 | 0.122 | 0.955 | 50.309 | 130 | 98 |
| FXST | 0.084 | 0.141 | -0.193 | 0.361 | 0.552 | 0.955 | 2.249 | 130 | 98 |
| GCC | 0.105 | 0.139 | -0.168 | 0.378 | 0.452 | 0.955 | 0.000 | 130 | 98 |
| IC | -0.016 | 0.139 | -0.290 | 0.257 | 0.906 | 0.970 | 0.000 | 130 | 98 |
| IFO | 0.151 | 0.140 | -0.123 | 0.424 | 0.280 | 0.955 | 0.000 | 130 | 98 |
| PCR | 0.173 | 0.140 | -0.100 | 0.447 | 0.215 | 0.955 | 0.000 | 130 | 98 |
| PLIC | -0.228 | 0.139 | -0.501 | 0.045 | 0.102 | 0.955 | 0.000 | 130 | 98 |
| PTR | 0.255 | 0.140 | -0.019 | 0.529 | 0.068 | 0.955 | 0.000 | 130 | 98 |
| RLIC | 0.139 | 0.159 | -0.173 | 0.452 | 0.383 | 0.955 | 21.960 | 130 | 98 |
| SCC | -0.005 | 0.139 | -0.278 | 0.267 | 0.969 | 0.970 | 0.000 | 130 | 98 |
| SCR | 0.047 | 0.139 | -0.226 | 0.319 | 0.737 | 0.970 | 0.000 | 130 | 98 |
| SFO | 0.009 | 0.139 | -0.264 | 0.282 | 0.948 | 0.970 | 0.000 | 130 | 98 |
| SLF | 0.121 | 0.140 | -0.152 | 0.395 | 0.386 | 0.955 | 0.005 | 130 | 98 |
| SS | -0.039 | 0.168 | -0.369 | 0.291 | 0.817 | 0.970 | 29.922 | 130 | 98 |
| UNC | 0.126 | 0.139 | -0.147 | 0.399 | 0.366 | 0.955 | 0.000 | 130 | 98 |
| AverageMD | 0.072 | 0.221 | -0.360 | 0.505 | 0.743 | 0.970 | 58.414 | 130 | 98 |

Table S51. Cohen’s d effect sizes after meta-analysis, for RD differences between patients with first episode MDD and controls **in adolescents only**. Age, sex, agexsex, age^2^, age^2^xsex and scansite included as covariates.

| Region | Cohen’s d | SE | CI LB | CI UB | P-value | FDR P-value | I^2^ | Controls | Patients |
| --- | --- | --- | --- | --- | --- | --- | --- | --- | --- |
| ACR | 0.024 | 0.223 | -0.413 | 0.461 | 0.916 | 0.938 | 59.381 | 130 | 98 |
| ALIC | -0.059 | 0.266 | -0.580 | 0.462 | 0.824 | 0.938 | 71.085 | 130 | 98 |
| BCC | 0.230 | 0.152 | -0.067 | 0.528 | 0.129 | 0.938 | 13.919 | 130 | 98 |
| CC | 0.184 | 0.152 | -0.113 | 0.482 | 0.225 | 0.938 | 14.426 | 130 | 98 |
| CGC | -0.093 | 0.283 | -0.646 | 0.461 | 0.743 | 0.938 | 74.444 | 130 | 98 |
| CGH | -0.148 | 0.333 | -0.800 | 0.504 | 0.656 | 0.938 | 81.330 | 130 | 98 |
| CR | 0.050 | 0.233 | -0.406 | 0.506 | 0.831 | 0.938 | 62.659 | 130 | 98 |
| CST | 0.095 | 0.140 | -0.178 | 0.369 | 0.494 | 0.938 | 0.000 | 130 | 98 |
| EC | -0.184 | 0.301 | -0.774 | 0.407 | 0.542 | 0.938 | 77.340 | 130 | 98 |
| FX | 0.274 | 0.188 | -0.093 | 0.642 | 0.144 | 0.938 | 42.204 | 130 | 98 |
| FXST | -0.081 | 0.257 | -0.586 | 0.423 | 0.751 | 0.938 | 69.223 | 130 | 98 |
| GCC | 0.153 | 0.155 | -0.151 | 0.457 | 0.324 | 0.938 | 17.889 | 130 | 98 |
| IC | -0.091 | 0.183 | -0.450 | 0.267 | 0.618 | 0.938 | 40.085 | 130 | 98 |
| IFO | 0.181 | 0.140 | -0.093 | 0.455 | 0.196 | 0.938 | 0.000 | 130 | 98 |
| PCR | 0.140 | 0.177 | -0.207 | 0.486 | 0.429 | 0.938 | 35.960 | 130 | 98 |
| PLIC | -0.244 | 0.140 | -0.518 | 0.030 | 0.081 | 0.938 | 0.000 | 130 | 98 |
| PTR | 0.113 | 0.165 | -0.210 | 0.437 | 0.493 | 0.938 | 27.035 | 130 | 98 |
| RLIC | 0.015 | 0.195 | -0.366 | 0.397 | 0.938 | 0.938 | 47.038 | 130 | 98 |
| SCC | 0.058 | 0.139 | -0.215 | 0.331 | 0.677 | 0.938 | 0.000 | 130 | 98 |
| SCR | 0.035 | 0.242 | -0.439 | 0.509 | 0.884 | 0.938 | 65.363 | 130 | 98 |
| SFO | 0.051 | 0.219 | -0.377 | 0.480 | 0.815 | 0.938 | 57.730 | 130 | 98 |
| SLF | 0.046 | 0.159 | -0.266 | 0.358 | 0.773 | 0.938 | 21.700 | 130 | 98 |
| SS | -0.119 | 0.177 | -0.465 | 0.228 | 0.502 | 0.938 | 36.036 | 130 | 98 |
| UNC | 0.161 | 0.139 | -0.112 | 0.435 | 0.247 | 0.938 | 0.000 | 130 | 98 |
| AverageRD | 0.089 | 0.261 | -0.422 | 0.601 | 0.733 | 0.938 | 70.131 | 130 | 98 |

Table S52. Cohen’s d effect sizes after meta-analysis, for FA differences between patients with first episode MDD and patients with recurrent MDD **in adolescents only**. Age, sex, agexsex, age^2^, age^2^xsex and scansite included as covariates.

| Region | Cohen’s d | SE | CI LB | CI UB | P-value | FDR P-value | I^2^ | Controls | Patients |
| --- | --- | --- | --- | --- | --- | --- | --- | --- | --- |
| ACR | 0.019 | 0.148 | -0.271 | 0.308 | 0.899 | 0.899 | 0.000 | 81 | 109 |
| ALIC | 0.145 | 0.148 | -0.146 | 0.435 | 0.328 | 0.899 | 0.007 | 81 | 109 |
| BCC | -0.139 | 0.153 | -0.440 | 0.161 | 0.363 | 0.899 | 5.209 | 81 | 109 |
| CC | -0.093 | 0.148 | -0.383 | 0.196 | 0.528 | 0.899 | 0.000 | 81 | 109 |
| CGC | -0.068 | 0.148 | -0.357 | 0.222 | 0.645 | 0.899 | 0.000 | 81 | 109 |
| CGH | -0.083 | 0.203 | -0.481 | 0.316 | 0.685 | 0.899 | 42.340 | 81 | 109 |
| CR | 0.032 | 0.148 | -0.257 | 0.322 | 0.826 | 0.899 | 0.000 | 81 | 109 |
| CST | 0.028 | 0.148 | -0.262 | 0.317 | 0.851 | 0.899 | 0.000 | 81 | 109 |
| EC | -0.036 | 0.148 | -0.326 | 0.254 | 0.807 | 0.899 | 0.000 | 81 | 109 |
| FX | -0.267 | 0.285 | -0.825 | 0.291 | 0.348 | 0.899 | 69.522 | 81 | 109 |
| FXST | -0.142 | 0.195 | -0.523 | 0.240 | 0.466 | 0.899 | 37.649 | 81 | 109 |
| GCC | -0.054 | 0.148 | -0.343 | 0.236 | 0.716 | 0.899 | 0.000 | 81 | 109 |
| IC | 0.041 | 0.187 | -0.324 | 0.407 | 0.825 | 0.899 | 32.863 | 81 | 109 |
| IFO | -0.065 | 0.148 | -0.354 | 0.225 | 0.661 | 0.899 | 0.000 | 81 | 109 |
| PCR | 0.112 | 0.148 | -0.178 | 0.401 | 0.449 | 0.899 | 0.000 | 81 | 109 |
| PLIC | -0.079 | 0.189 | -0.449 | 0.291 | 0.676 | 0.899 | 34.273 | 81 | 109 |
| PTR | 0.079 | 0.158 | -0.232 | 0.389 | 0.620 | 0.899 | 10.546 | 81 | 109 |
| RLIC | 0.085 | 0.165 | -0.237 | 0.408 | 0.604 | 0.899 | 16.225 | 81 | 109 |
| SCC | -0.074 | 0.190 | -0.448 | 0.299 | 0.696 | 0.899 | 35.254 | 81 | 109 |
| SCR | -0.023 | 0.177 | -0.371 | 0.324 | 0.895 | 0.899 | 26.322 | 81 | 109 |
| SFO | 0.055 | 0.148 | -0.234 | 0.344 | 0.710 | 0.899 | 0.000 | 81 | 109 |
| SLF | -0.269 | 0.148 | -0.560 | 0.022 | 0.070 | 0.899 | 0.000 | 81 | 109 |
| SS | -0.284 | 0.189 | -0.654 | 0.086 | 0.133 | 0.899 | 33.570 | 81 | 109 |
| UNC | 0.184 | 0.148 | -0.106 | 0.473 | 0.215 | 0.899 | 0.000 | 81 | 109 |
| AverageFA | -0.097 | 0.208 | -0.506 | 0.312 | 0.642 | 0.899 | 45.162 | 81 | 109 |

Table S53. Cohen’s d effect sizes after meta-analysis, for AD differences between patients with first episode MDD and patients with recurrent MDD **in adolescents only**. Age, sex, agexsex, age^2^, age^2^xsex and scansite included as covariates.

| Region | Cohen’s d | SE | CI LB | CI UB | P-value | FDR P-value | I^2^ | Controls | Patients |
| --- | --- | --- | --- | --- | --- | --- | --- | --- | --- |
| ACR | 0.113 | 0.332 | -0.537 | 0.763 | 0.733 | 0.928 | 77.645 | 81 | 109 |
| ALIC | 0.242 | 0.166 | -0.083 | 0.567 | 0.145 | 0.928 | 16.726 | 81 | 109 |
| BCC | 0.027 | 0.148 | -0.263 | 0.316 | 0.856 | 0.928 | 0.000 | 81 | 109 |
| CC | 0.013 | 0.148 | -0.276 | 0.303 | 0.928 | 0.928 | 0.000 | 81 | 109 |
| CGC | 0.070 | 0.148 | -0.220 | 0.359 | 0.637 | 0.928 | 0.000 | 81 | 109 |
| CGH | -0.017 | 0.148 | -0.307 | 0.272 | 0.908 | 0.928 | 0.000 | 81 | 109 |
| CR | 0.096 | 0.247 | -0.388 | 0.580 | 0.697 | 0.928 | 60.347 | 81 | 109 |
| CST | 0.380 | 0.284 | -0.177 | 0.937 | 0.181 | 0.928 | 69.139 | 81 | 109 |
| EC | 0.253 | 0.148 | -0.038 | 0.544 | 0.088 | 0.928 | 0.000 | 81 | 109 |
| FX | -0.031 | 0.148 | -0.321 | 0.258 | 0.831 | 0.928 | 0.000 | 81 | 109 |
| FXST | 0.100 | 0.148 | -0.190 | 0.390 | 0.497 | 0.928 | 0.000 | 81 | 109 |
| GCC | 0.018 | 0.148 | -0.272 | 0.307 | 0.905 | 0.928 | 0.000 | 81 | 109 |
| IC | 0.164 | 0.148 | -0.126 | 0.454 | 0.268 | 0.928 | 0.000 | 81 | 109 |
| IFO | 0.059 | 0.152 | -0.238 | 0.357 | 0.697 | 0.928 | 4.009 | 81 | 109 |
| PCR | 0.038 | 0.148 | -0.251 | 0.328 | 0.796 | 0.928 | 0.000 | 81 | 109 |
| PLIC | 0.119 | 0.148 | -0.171 | 0.408 | 0.422 | 0.928 | 0.000 | 81 | 109 |
| PTR | 0.121 | 0.148 | -0.169 | 0.410 | 0.414 | 0.928 | 0.000 | 81 | 109 |
| RLIC | 0.102 | 0.148 | -0.188 | 0.392 | 0.490 | 0.928 | 0.000 | 81 | 109 |
| SCC | 0.016 | 0.148 | -0.274 | 0.306 | 0.914 | 0.928 | 0.005 | 81 | 109 |
| SCR | 0.144 | 0.148 | -0.146 | 0.434 | 0.331 | 0.928 | 0.000 | 81 | 109 |
| SFO | 0.231 | 0.195 | -0.152 | 0.614 | 0.237 | 0.928 | 37.739 | 81 | 109 |
| SLF | 0.097 | 0.148 | -0.192 | 0.387 | 0.511 | 0.928 | 0.000 | 81 | 109 |
| SS | 0.081 | 0.148 | -0.208 | 0.370 | 0.583 | 0.928 | 0.000 | 81 | 109 |
| UNC | 0.059 | 0.148 | -0.231 | 0.349 | 0.692 | 0.928 | 0.001 | 81 | 109 |
| AverageAD | 0.309 | 0.148 | 0.018 | 0.599 | 0.038 | 0.928 | 0.000 | 81 | 109 |

Table S54. Cohen’s d effect sizes after meta-analysis, for MD differences between patients with first episode MDD and patients with recurrent MDD **in adolescents only**. Age, sex, agexsex, age^2^, age^2^xsex and scansite included as covariates.

| Region | Cohen’s d | SE | CI LB | CI UB | P-value | FDR P-value | I^2^ | Controls | Patients |
| --- | --- | --- | --- | --- | --- | --- | --- | --- | --- |
| ACR | 0.123 | 0.299 | -0.463 | 0.710 | 0.680 | 0.754 | 72.640 | 81 | 109 |
| ALIC | 0.305 | 0.152 | 0.008 | 0.602 | 0.044 | 0.283 | 2.874 | 81 | 109 |
| BCC | 0.058 | 0.148 | -0.231 | 0.348 | 0.694 | 0.754 | 0.000 | 81 | 109 |
| CC | 0.143 | 0.148 | -0.147 | 0.433 | 0.333 | 0.522 | 0.000 | 81 | 109 |
| CGC | 0.218 | 0.148 | -0.072 | 0.509 | 0.141 | 0.418 | 0.000 | 81 | 109 |
| CGH | 0.303 | 0.314 | -0.312 | 0.918 | 0.334 | 0.522 | 74.756 | 81 | 109 |
| CR | 0.142 | 0.246 | -0.341 | 0.625 | 0.564 | 0.710 | 60.066 | 81 | 109 |
| CST | 0.191 | 0.208 | -0.216 | 0.599 | 0.357 | 0.525 | 44.593 | 81 | 109 |
| EC | 0.409 | 0.149 | 0.117 | 0.702 | 0.006 | 0.075 | 0.000 | 81 | 109 |
| FX | 0.070 | 0.148 | -0.219 | 0.359 | 0.635 | 0.754 | 0.000 | 81 | 109 |
| FXST | 0.202 | 0.148 | -0.087 | 0.492 | 0.171 | 0.418 | 0.000 | 81 | 109 |
| GCC | 0.109 | 0.148 | -0.181 | 0.399 | 0.462 | 0.642 | 0.000 | 81 | 109 |
| IC | 0.271 | 0.148 | -0.020 | 0.562 | 0.068 | 0.283 | 0.000 | 81 | 109 |
| IFO | 0.167 | 0.293 | -0.407 | 0.742 | 0.568 | 0.710 | 71.493 | 81 | 109 |
| PCR | 0.016 | 0.148 | -0.274 | 0.306 | 0.914 | 0.914 | 0.003 | 81 | 109 |
| PLIC | 0.275 | 0.149 | -0.016 | 0.566 | 0.064 | 0.283 | 0.000 | 81 | 109 |
| PTR | 0.148 | 0.148 | -0.142 | 0.438 | 0.318 | 0.522 | 0.000 | 81 | 109 |
| RLIC | 0.151 | 0.148 | -0.138 | 0.441 | 0.306 | 0.522 | 0.000 | 81 | 109 |
| SCC | 0.238 | 0.203 | -0.159 | 0.635 | 0.240 | 0.500 | 41.754 | 81 | 109 |
| SCR | 0.249 | 0.187 | -0.118 | 0.615 | 0.184 | 0.418 | 32.497 | 81 | 109 |
| SFO | 0.220 | 0.148 | -0.071 | 0.510 | 0.139 | 0.418 | 0.000 | 81 | 109 |
| SLF | 0.278 | 0.148 | -0.013 | 0.569 | 0.061 | 0.283 | 0.000 | 81 | 109 |
| SS | 0.243 | 0.180 | -0.110 | 0.595 | 0.177 | 0.418 | 27.493 | 81 | 109 |
| UNC | -0.053 | 0.279 | -0.601 | 0.494 | 0.848 | 0.883 | 68.654 | 81 | 109 |
| AverageMD | 0.436 | 0.149 | 0.143 | 0.729 | 0.004 | 0.075 | 0.000 | 81 | 109 |

Table S55. Cohen’s d effect sizes after meta-analysis, for RD differences between patients with first episode MDD and patients with recurrent MDD **in adolescents only**. Age, sex, agexsex, age^2^, age^2^xsex and scansite included as covariates.

| Region | Cohen’s d | SE | CI LB | CI UB | P-value | FDR P-value | I^2^ | Controls | Patients |
| --- | --- | --- | --- | --- | --- | --- | --- | --- | --- |
| ACR | 0.042 | 0.148 | -0.248 | 0.332 | 0.779 | 0.855 | 0.005 | 81 | 109 |
| ALIC | 0.234 | 0.148 | -0.057 | 0.524 | 0.115 | 0.359 | 0.000 | 81 | 109 |
| BCC | 0.047 | 0.148 | -0.243 | 0.337 | 0.750 | 0.855 | 0.000 | 81 | 109 |
| CC | 0.139 | 0.148 | -0.151 | 0.428 | 0.348 | 0.588 | 0.000 | 81 | 109 |
| CGC | 0.219 | 0.148 | -0.072 | 0.510 | 0.140 | 0.389 | 0.000 | 81 | 109 |
| CGH | 0.249 | 0.295 | -0.330 | 0.827 | 0.400 | 0.588 | 71.682 | 81 | 109 |
| CR | 0.076 | 0.148 | -0.214 | 0.366 | 0.606 | 0.797 | 0.000 | 81 | 109 |
| CST | 0.001 | 0.148 | -0.288 | 0.291 | 0.994 | 0.994 | 0.000 | 81 | 109 |
| EC | 0.392 | 0.149 | 0.100 | 0.684 | 0.009 | 0.113 | 0.000 | 81 | 109 |
| FX | 0.130 | 0.148 | -0.159 | 0.420 | 0.378 | 0.588 | 0.000 | 81 | 109 |
| FXST | 0.248 | 0.148 | -0.043 | 0.538 | 0.095 | 0.339 | 0.000 | 81 | 109 |
| GCC | 0.093 | 0.148 | -0.197 | 0.383 | 0.530 | 0.736 | 0.000 | 81 | 109 |
| IC | 0.249 | 0.148 | -0.041 | 0.540 | 0.092 | 0.339 | 0.000 | 81 | 109 |
| IFO | 0.052 | 0.191 | -0.323 | 0.426 | 0.787 | 0.855 | 35.405 | 81 | 109 |
| PCR | -0.012 | 0.148 | -0.302 | 0.277 | 0.933 | 0.972 | 0.000 | 81 | 109 |
| PLIC | 0.263 | 0.148 | -0.028 | 0.554 | 0.076 | 0.339 | 0.000 | 81 | 109 |
| PTR | 0.162 | 0.148 | -0.128 | 0.452 | 0.274 | 0.533 | 0.000 | 81 | 109 |
| RLIC | 0.133 | 0.148 | -0.157 | 0.423 | 0.369 | 0.588 | 0.000 | 81 | 109 |
| SCC | 0.290 | 0.148 | -0.001 | 0.581 | 0.051 | 0.319 | 0.000 | 81 | 109 |
| SCR | 0.205 | 0.166 | -0.120 | 0.530 | 0.216 | 0.491 | 16.759 | 81 | 109 |
| SFO | 0.161 | 0.148 | -0.129 | 0.450 | 0.277 | 0.533 | 0.000 | 81 | 109 |
| SLF | 0.316 | 0.149 | 0.024 | 0.607 | 0.034 | 0.283 | 0.000 | 81 | 109 |
| SS | 0.319 | 0.240 | -0.151 | 0.788 | 0.184 | 0.460 | 57.452 | 81 | 109 |
| UNC | -0.071 | 0.193 | -0.449 | 0.308 | 0.714 | 0.855 | 36.511 | 81 | 109 |
| AverageRD | 0.428 | 0.149 | 0.135 | 0.721 | 0.004 | 0.100 | 0.000 | 81 | 109 |

**6: Age of onset**

Table S56. Cohen’s d effect sizes after meta-analysis, for FA differences between adolescent age of onset MDD patients (age<22 years) and healthy controls **in adults only**. Age, sex, agexsex, age^2^, age^2^xsex and scansite included as covariates.

| Region | Cohen’s d | SE | CI LB | CI UB | P-value | FDR P-value | I^2^ | Controls | Patients |
| --- | --- | --- | --- | --- | --- | --- | --- | --- | --- |
| ACR | -0.203 | 0.076 | -0.352 | -0.054 | 0.007 | 0.175 | 0.732 | 869 | 251 |
| ALIC | -0.110 | 0.078 | -0.263 | 0.043 | 0.159 | 0.547 | 3.793 | 869 | 251 |
| BCC | -0.131 | 0.120 | -0.367 | 0.104 | 0.273 | 0.617 | 51.235 | 869 | 251 |
| CC | -0.091 | 0.110 | -0.305 | 0.124 | 0.407 | 0.668 | 42.230 | 869 | 251 |
| CGC | -0.103 | 0.110 | -0.319 | 0.114 | 0.352 | 0.629 | 42.657 | 869 | 251 |
| CGH | -0.073 | 0.103 | -0.274 | 0.129 | 0.481 | 0.668 | 35.287 | 869 | 251 |
| CR | -0.128 | 0.075 | -0.276 | 0.020 | 0.089 | 0.547 | 0.007 | 869 | 251 |
| CST | -0.056 | 0.107 | -0.267 | 0.154 | 0.600 | 0.688 | 39.924 | 869 | 251 |
| EC | -0.037 | 0.075 | -0.185 | 0.111 | 0.624 | 0.688 | 0.000 | 869 | 251 |
| FX | -0.085 | 0.085 | -0.252 | 0.083 | 0.321 | 0.617 | 13.892 | 869 | 251 |
| FXST | -0.099 | 0.138 | -0.369 | 0.171 | 0.473 | 0.668 | 62.712 | 869 | 251 |
| GCC | -0.141 | 0.086 | -0.309 | 0.027 | 0.100 | 0.547 | 14.335 | 869 | 251 |
| IC | -0.106 | 0.075 | -0.254 | 0.041 | 0.158 | 0.547 | 0.004 | 869 | 251 |
| IFO | 0.038 | 0.075 | -0.109 | 0.186 | 0.609 | 0.688 | 0.000 | 869 | 251 |
| PCR | -0.046 | 0.097 | -0.236 | 0.144 | 0.633 | 0.688 | 28.491 | 869 | 251 |
| PLIC | -0.093 | 0.075 | -0.241 | 0.055 | 0.216 | 0.600 | 0.000 | 869 | 251 |
| PTR | -0.205 | 0.126 | -0.451 | 0.042 | 0.103 | 0.547 | 55.154 | 869 | 251 |
| RLIC | -0.053 | 0.075 | -0.201 | 0.094 | 0.478 | 0.668 | 0.000 | 869 | 251 |
| SCC | 0.109 | 0.075 | -0.038 | 0.257 | 0.146 | 0.547 | 0.000 | 869 | 251 |
| SCR | -0.020 | 0.080 | -0.177 | 0.137 | 0.802 | 0.802 | 6.923 | 869 | 251 |
| SFO | -0.081 | 0.075 | -0.229 | 0.066 | 0.280 | 0.617 | 0.000 | 869 | 251 |
| SLF | -0.057 | 0.096 | -0.246 | 0.132 | 0.555 | 0.688 | 27.902 | 869 | 251 |
| SS | -0.123 | 0.122 | -0.361 | 0.115 | 0.312 | 0.617 | 52.316 | 869 | 251 |
| UNC | -0.025 | 0.075 | -0.172 | 0.122 | 0.741 | 0.772 | 0.000 | 869 | 251 |
| AverageFA | -0.102 | 0.075 | -0.250 | 0.046 | 0.175 | 0.547 | 0.014 | 869 | 251 |

Table S57. Cohen’s d effect sizes after meta-analysis, for AD differences between adolescent age of onset MDD patients (age<22 years) and healthy controls **in adults only**. Age, sex, agexsex, age^2^, age^2^xsex and scansite included as covariates.

| Region | Cohen’s d | SE | CI LB | CI UB | P-value | FDR P-value | I^2^ | Controls | Patients |
| --- | --- | --- | --- | --- | --- | --- | --- | --- | --- |
| ACR | -0.075 | 0.108 | -0.287 | 0.137 | 0.485 | 0.867 | 40.838 | 869 | 251 |
| ALIC | -0.038 | 0.075 | -0.186 | 0.109 | 0.610 | 0.867 | 0.000 | 869 | 251 |
| BCC | -0.046 | 0.075 | -0.194 | 0.101 | 0.536 | 0.867 | 0.000 | 869 | 251 |
| CC | -0.066 | 0.075 | -0.214 | 0.081 | 0.378 | 0.867 | 0.000 | 869 | 251 |
| CGC | -0.167 | 0.075 | -0.315 | -0.020 | 0.026 | 0.650 | 0.003 | 869 | 251 |
| CGH | -0.040 | 0.094 | -0.225 | 0.144 | 0.668 | 0.879 | 25.067 | 869 | 251 |
| CR | -0.017 | 0.075 | -0.165 | 0.130 | 0.818 | 0.921 | 0.000 | 869 | 251 |
| CST | 0.118 | 0.075 | -0.030 | 0.265 | 0.119 | 0.867 | 0.004 | 869 | 251 |
| EC | 0.011 | 0.075 | -0.136 | 0.158 | 0.884 | 0.921 | 0.000 | 869 | 251 |
| FX | 0.069 | 0.075 | -0.078 | 0.216 | 0.359 | 0.867 | 0.000 | 869 | 251 |
| FXST | 0.018 | 0.099 | -0.175 | 0.211 | 0.857 | 0.921 | 30.717 | 869 | 251 |
| GCC | -0.048 | 0.075 | -0.196 | 0.099 | 0.520 | 0.867 | 0.034 | 869 | 251 |
| IC | -0.047 | 0.096 | -0.235 | 0.141 | 0.624 | 0.867 | 27.670 | 869 | 251 |
| IFO | 0.115 | 0.075 | -0.032 | 0.263 | 0.126 | 0.867 | 0.000 | 869 | 251 |
| PCR | -0.071 | 0.075 | -0.219 | 0.076 | 0.341 | 0.867 | 0.000 | 869 | 251 |
| PLIC | 0.059 | 0.108 | -0.154 | 0.271 | 0.588 | 0.867 | 41.024 | 869 | 251 |
| PTR | -0.028 | 0.089 | -0.203 | 0.147 | 0.756 | 0.900 | 19.151 | 869 | 251 |
| RLIC | -0.088 | 0.096 | -0.276 | 0.100 | 0.358 | 0.867 | 27.749 | 869 | 251 |
| SCC | -0.055 | 0.089 | -0.229 | 0.119 | 0.535 | 0.867 | 18.714 | 869 | 251 |
| SCR | 0.087 | 0.079 | -0.068 | 0.242 | 0.270 | 0.867 | 5.070 | 869 | 251 |
| SFO | -0.052 | 0.075 | -0.199 | 0.096 | 0.492 | 0.867 | 0.000 | 869 | 251 |
| SLF | -0.094 | 0.084 | -0.259 | 0.070 | 0.261 | 0.867 | 12.255 | 869 | 251 |
| SS | 0.002 | 0.075 | -0.145 | 0.150 | 0.974 | 0.974 | 0.010 | 869 | 251 |
| UNC | 0.065 | 0.075 | -0.083 | 0.212 | 0.389 | 0.867 | 0.000 | 869 | 251 |
| AverageAD | -0.026 | 0.075 | -0.174 | 0.121 | 0.727 | 0.900 | 0.000 | 869 | 251 |

Table S58. Cohen’s d effect sizes after meta-analysis, for MD differences between adolescent age of onset MDD patients (age<22 years) and healthy controls **in adults only**. Age, sex, agexsex, age^2^, age^2^xsex and scansite included as covariates.

| Region | Cohen’s d | SE | CI LB | CI UB | P-value | FDR P-value | I^2^ | Controls | Patients |
| --- | --- | --- | --- | --- | --- | --- | --- | --- | --- |
| ACR | -0.028 | 0.075 | -0.175 | 0.120 | 0.713 | 0.938 | 0.001 | 869 | 251 |
| ALIC | -0.060 | 0.075 | -0.207 | 0.088 | 0.427 | 0.890 | 0.000 | 869 | 251 |
| BCC | 0.065 | 0.075 | -0.082 | 0.212 | 0.388 | 0.890 | 0.000 | 869 | 251 |
| CC | 0.003 | 0.075 | -0.144 | 0.151 | 0.964 | 0.977 | 0.009 | 869 | 251 |
| CGC | -0.075 | 0.075 | -0.222 | 0.073 | 0.321 | 0.890 | 0.016 | 869 | 251 |
| CGH | 0.004 | 0.121 | -0.232 | 0.240 | 0.974 | 0.977 | 51.427 | 869 | 251 |
| CR | 0.035 | 0.075 | -0.112 | 0.183 | 0.637 | 0.937 | 0.000 | 869 | 251 |
| CST | 0.114 | 0.125 | -0.131 | 0.359 | 0.362 | 0.890 | 54.698 | 869 | 251 |
| EC | -0.043 | 0.075 | -0.190 | 0.104 | 0.569 | 0.922 | 0.000 | 869 | 251 |
| FX | 0.014 | 0.075 | -0.133 | 0.162 | 0.849 | 0.977 | 0.000 | 869 | 251 |
| FXST | 0.133 | 0.132 | -0.125 | 0.392 | 0.313 | 0.890 | 59.220 | 869 | 251 |
| GCC | -0.029 | 0.075 | -0.177 | 0.118 | 0.697 | 0.938 | 0.005 | 869 | 251 |
| IC | -0.082 | 0.075 | -0.230 | 0.065 | 0.272 | 0.890 | 0.000 | 869 | 251 |
| IFO | -0.058 | 0.107 | -0.268 | 0.152 | 0.590 | 0.922 | 39.885 | 869 | 251 |
| PCR | 0.062 | 0.075 | -0.086 | 0.209 | 0.412 | 0.890 | 0.000 | 869 | 251 |
| PLIC | -0.047 | 0.081 | -0.207 | 0.112 | 0.563 | 0.922 | 8.515 | 869 | 251 |
| PTR | 0.133 | 0.075 | -0.015 | 0.280 | 0.079 | 0.890 | 0.001 | 869 | 251 |
| RLIC | -0.118 | 0.075 | -0.266 | 0.029 | 0.115 | 0.890 | 0.000 | 869 | 251 |
| SCC | -0.067 | 0.075 | -0.214 | 0.080 | 0.373 | 0.890 | 0.000 | 869 | 251 |
| SCR | 0.106 | 0.075 | -0.042 | 0.253 | 0.161 | 0.890 | 0.000 | 869 | 251 |
| SFO | 0.043 | 0.075 | -0.104 | 0.191 | 0.565 | 0.922 | 0.000 | 869 | 251 |
| SLF | -0.002 | 0.081 | -0.161 | 0.157 | 0.977 | 0.977 | 8.160 | 869 | 251 |
| SS | 0.012 | 0.075 | -0.135 | 0.160 | 0.869 | 0.977 | 0.000 | 869 | 251 |
| UNC | 0.115 | 0.085 | -0.051 | 0.281 | 0.174 | 0.890 | 12.680 | 869 | 251 |
| AverageMD | 0.005 | 0.075 | -0.142 | 0.153 | 0.943 | 0.977 | 0.000 | 869 | 251 |

Table S59. Cohen’s d effect sizes after meta-analysis, for RD differences between adolescent age of onset MDD patients (age<22 years) and healthy controls **in adults only**. Age, sex, agexsex, age^2^, age^2^xsex and scansite included as covariates.

| Region | Cohen’s d | SE | CI LB | CI UB | P-value | FDR P-value | I^2^ | Controls | Patients |
| --- | --- | --- | --- | --- | --- | --- | --- | --- | --- |
| ACR | 0.064 | 0.075 | -0.084 | 0.211 | 0.397 | 0.840 | 0.004 | 869 | 251 |
| ALIC | 0.029 | 0.075 | -0.118 | 0.177 | 0.699 | 0.884 | 0.009 | 869 | 251 |
| BCC | 0.074 | 0.080 | -0.082 | 0.231 | 0.351 | 0.840 | 6.403 | 869 | 251 |
| CC | 0.026 | 0.075 | -0.122 | 0.173 | 0.731 | 0.884 | 0.048 | 869 | 251 |
| CGC | 0.020 | 0.114 | -0.203 | 0.244 | 0.859 | 0.907 | 46.113 | 869 | 251 |
| CGH | 0.105 | 0.119 | -0.128 | 0.339 | 0.376 | 0.840 | 50.354 | 869 | 251 |
| CR | 0.100 | 0.075 | -0.047 | 0.248 | 0.183 | 0.840 | 0.009 | 869 | 251 |
| CST | 0.113 | 0.136 | -0.152 | 0.379 | 0.403 | 0.840 | 61.368 | 869 | 251 |
| EC | -0.024 | 0.075 | -0.172 | 0.124 | 0.750 | 0.884 | 0.000 | 869 | 251 |
| FX | 0.021 | 0.075 | -0.126 | 0.169 | 0.778 | 0.884 | 0.000 | 869 | 251 |
| FXST | 0.236 | 0.160 | -0.078 | 0.550 | 0.141 | 0.840 | 72.400 | 869 | 251 |
| GCC | 0.028 | 0.075 | -0.120 | 0.175 | 0.711 | 0.884 | 0.001 | 869 | 251 |
| IC | 0.012 | 0.075 | -0.136 | 0.159 | 0.875 | 0.907 | 0.015 | 869 | 251 |
| IFO | -0.061 | 0.092 | -0.241 | 0.118 | 0.504 | 0.884 | 22.128 | 869 | 251 |
| PCR | 0.103 | 0.075 | -0.044 | 0.251 | 0.171 | 0.840 | 0.010 | 869 | 251 |
| PLIC | 0.012 | 0.104 | -0.192 | 0.217 | 0.907 | 0.907 | 36.791 | 869 | 251 |
| PTR | 0.171 | 0.100 | -0.025 | 0.368 | 0.087 | 0.840 | 32.091 | 869 | 251 |
| RLIC | -0.039 | 0.075 | -0.186 | 0.109 | 0.606 | 0.884 | 0.000 | 869 | 251 |
| SCC | -0.083 | 0.075 | -0.230 | 0.064 | 0.270 | 0.840 | 0.001 | 869 | 251 |
| SCR | 0.108 | 0.075 | -0.039 | 0.256 | 0.150 | 0.840 | 0.002 | 869 | 251 |
| SFO | 0.078 | 0.075 | -0.069 | 0.226 | 0.298 | 0.840 | 0.003 | 869 | 251 |
| SLF | 0.074 | 0.101 | -0.123 | 0.272 | 0.461 | 0.884 | 33.120 | 869 | 251 |
| SS | 0.037 | 0.075 | -0.111 | 0.184 | 0.624 | 0.884 | 0.002 | 869 | 251 |
| UNC | 0.093 | 0.075 | -0.055 | 0.240 | 0.219 | 0.840 | 0.006 | 869 | 251 |
| AverageRD | 0.042 | 0.075 | -0.105 | 0.190 | 0.574 | 0.884 | 0.003 | 869 | 251 |

Table S60. Cohen’s d effect sizes after meta-analysis, for FA differences between adult age of onset MDD patients (age ≥ 22 years) and healthy controls **in adults only**. Age, sex, agexsex, age^2^, age^2^xsex and scansite included as covariates.

| Region | Cohen’s d | SE | CI LB | CI UB | P-value | FDR P-value | I^2^ | Controls | Patients |
| --- | --- | --- | --- | --- | --- | --- | --- | --- | --- |
| ACR | -0.204 | 0.099 | -0.397 | -0.010 | 0.039 | 0.077 | 43.116 | 853 | 399 |
| ALIC | -0.093 | 0.064 | -0.218 | 0.031 | 0.142 | 0.209 | 0.000 | 853 | 399 |
| BCC | -0.153 | 0.079 | -0.307 | 0.001 | 0.051 | 0.091 | 18.778 | 853 | 399 |
| CC | -0.201 | 0.087 | -0.371 | -0.032 | 0.020 | 0.050 | 28.888 | 853 | 399 |
| CGC | -0.162 | 0.064 | -0.287 | -0.036 | 0.011 | 0.041 | 0.000 | 853 | 399 |
| CGH | -0.135 | 0.064 | -0.260 | -0.010 | 0.034 | 0.077 | 0.000 | 853 | 399 |
| CR | -0.176 | 0.074 | -0.321 | -0.031 | 0.017 | 0.047 | 12.800 | 853 | 399 |
| CST | -0.154 | 0.113 | -0.376 | 0.068 | 0.174 | 0.242 | 56.474 | 853 | 399 |
| EC | -0.243 | 0.075 | -0.391 | -0.096 | 0.001 | 0.013 | 14.230 | 853 | 399 |
| FX | -0.069 | 0.064 | -0.194 | 0.056 | 0.276 | 0.329 | 0.000 | 853 | 399 |
| FXST | -0.110 | 0.089 | -0.286 | 0.065 | 0.217 | 0.271 | 32.809 | 853 | 399 |
| GCC | -0.189 | 0.074 | -0.334 | -0.044 | 0.011 | 0.041 | 12.835 | 853 | 399 |
| IC | -0.079 | 0.064 | -0.204 | 0.046 | 0.217 | 0.271 | 0.000 | 853 | 399 |
| IFO | -0.208 | 0.077 | -0.358 | -0.057 | 0.007 | 0.041 | 16.544 | 853 | 399 |
| PCR | -0.194 | 0.064 | -0.319 | -0.069 | 0.002 | 0.017 | 0.008 | 853 | 399 |
| PLIC | -0.020 | 0.064 | -0.145 | 0.105 | 0.752 | 0.783 | 0.000 | 853 | 399 |
| PTR | -0.097 | 0.118 | -0.329 | 0.135 | 0.412 | 0.448 | 60.109 | 853 | 399 |
| RLIC | -0.075 | 0.076 | -0.225 | 0.074 | 0.323 | 0.367 | 15.787 | 853 | 399 |
| SCC | -0.202 | 0.098 | -0.395 | -0.009 | 0.040 | 0.077 | 42.386 | 853 | 399 |
| SCR | -0.109 | 0.064 | -0.234 | 0.016 | 0.087 | 0.136 | 0.000 | 853 | 399 |
| SFO | -0.120 | 0.064 | -0.245 | 0.005 | 0.061 | 0.102 | 0.000 | 853 | 399 |
| SLF | -0.159 | 0.064 | -0.284 | -0.034 | 0.013 | 0.041 | 0.001 | 853 | 399 |
| SS | -0.216 | 0.064 | -0.341 | -0.091 | 0.001 | 0.013 | 0.004 | 853 | 399 |
| UNC | -0.028 | 0.141 | -0.304 | 0.248 | 0.841 | 0.841 | 72.320 | 853 | 399 |
| AverageFA | -0.158 | 0.064 | -0.283 | -0.033 | 0.013 | 0.041 | 0.000 | 853 | 399 |

Table S61. Cohen’s d effect sizes after meta-analysis, for AD differences between adult age of onset MDD patients (age ≥ 22 years) and healthy controls **in adults only**. Age, sex, agexsex, age^2^, age^2^xsex and scansite included as covariates.

| Region | Cohen’s d | SE | CI LB | CI UB | P-value | FDR P-value | I^2^ | Controls | Patients |
| --- | --- | --- | --- | --- | --- | --- | --- | --- | --- |
| ACR | -0.021 | 0.106 | -0.228 | 0.186 | 0.841 | 0.985 | 50.121 | 853 | 399 |
| ALIC | 0.001 | 0.083 | -0.162 | 0.165 | 0.990 | 0.997 | 25.251 | 853 | 399 |
| BCC | 0.031 | 0.118 | -0.201 | 0.263 | 0.792 | 0.985 | 60.007 | 853 | 399 |
| CC | 0.030 | 0.064 | -0.096 | 0.155 | 0.643 | 0.985 | 0.000 | 853 | 399 |
| CGC | -0.037 | 0.086 | -0.207 | 0.132 | 0.665 | 0.985 | 29.152 | 853 | 399 |
| CGH | -0.014 | 0.091 | -0.192 | 0.164 | 0.875 | 0.985 | 34.660 | 853 | 399 |
| CR | 0.023 | 0.091 | -0.156 | 0.202 | 0.802 | 0.985 | 35.045 | 853 | 399 |
| CST | 0.077 | 0.089 | -0.098 | 0.252 | 0.387 | 0.985 | 32.564 | 853 | 399 |
| EC | 0.032 | 0.113 | -0.190 | 0.254 | 0.776 | 0.985 | 56.408 | 853 | 399 |
| FX | 0.068 | 0.067 | -0.063 | 0.199 | 0.308 | 0.985 | 3.664 | 853 | 399 |
| FXST | 0.000 | 0.079 | -0.155 | 0.156 | 0.997 | 0.997 | 20.162 | 853 | 399 |
| GCC | -0.035 | 0.100 | -0.232 | 0.162 | 0.725 | 0.985 | 44.955 | 853 | 399 |
| IC | 0.026 | 0.095 | -0.160 | 0.212 | 0.782 | 0.985 | 39.369 | 853 | 399 |
| IFO | -0.023 | 0.101 | -0.221 | 0.175 | 0.818 | 0.985 | 45.899 | 853 | 399 |
| PCR | 0.008 | 0.064 | -0.118 | 0.133 | 0.906 | 0.985 | 0.005 | 853 | 399 |
| PLIC | 0.060 | 0.085 | -0.107 | 0.227 | 0.480 | 0.985 | 27.923 | 853 | 399 |
| PTR | 0.065 | 0.114 | -0.158 | 0.288 | 0.567 | 0.985 | 56.677 | 853 | 399 |
| RLIC | 0.022 | 0.104 | -0.181 | 0.225 | 0.832 | 0.985 | 48.464 | 853 | 399 |
| SCC | -0.016 | 0.064 | -0.141 | 0.109 | 0.805 | 0.985 | 0.000 | 853 | 399 |
| SCR | 0.092 | 0.074 | -0.053 | 0.237 | 0.213 | 0.985 | 12.813 | 853 | 399 |
| SFO | -0.037 | 0.077 | -0.187 | 0.113 | 0.628 | 0.985 | 16.429 | 853 | 399 |
| SLF | -0.016 | 0.064 | -0.141 | 0.109 | 0.801 | 0.985 | 0.000 | 853 | 399 |
| SS | -0.023 | 0.105 | -0.228 | 0.182 | 0.824 | 0.985 | 49.128 | 853 | 399 |
| UNC | 0.045 | 0.179 | -0.305 | 0.396 | 0.800 | 0.985 | 83.361 | 853 | 399 |
| AverageAD | 0.085 | 0.079 | -0.070 | 0.241 | 0.281 | 0.985 | 19.933 | 853 | 399 |

Table S62. Cohen’s d effect sizes after meta-analysis, for MD differences between adult age of onset MDD patients (age ≥ 22 years) and healthy controls **in adults only**. Age, sex, agexsex, age^2^, age^2^xsex and scansite included as covariates.

| Region | Cohen’s d | SE | CI LB | CI UB | P-value | FDR P-value | I^2^ | Controls | Patients |
| --- | --- | --- | --- | --- | --- | --- | --- | --- | --- |
| ACR | 0.093 | 0.064 | -0.032 | 0.218 | 0.145 | 0.385 | 0.005 | 853 | 399 |
| ALIC | 0.046 | 0.064 | -0.079 | 0.171 | 0.474 | 0.623 | 0.000 | 853 | 399 |
| BCC | 0.148 | 0.094 | -0.036 | 0.332 | 0.115 | 0.385 | 37.582 | 853 | 399 |
| CC | 0.129 | 0.068 | -0.005 | 0.263 | 0.059 | 0.385 | 5.523 | 853 | 399 |
| CGC | 0.087 | 0.064 | -0.038 | 0.212 | 0.172 | 0.391 | 0.000 | 853 | 399 |
| CGH | 0.044 | 0.064 | -0.081 | 0.169 | 0.489 | 0.623 | 0.000 | 853 | 399 |
| CR | 0.118 | 0.064 | -0.007 | 0.243 | 0.064 | 0.385 | 0.000 | 853 | 399 |
| CST | 0.104 | 0.086 | -0.065 | 0.273 | 0.228 | 0.438 | 28.731 | 853 | 399 |
| EC | 0.145 | 0.116 | -0.082 | 0.371 | 0.211 | 0.438 | 58.148 | 853 | 399 |
| FX | 0.043 | 0.064 | -0.082 | 0.168 | 0.498 | 0.623 | 0.000 | 853 | 399 |
| FXST | 0.094 | 0.064 | -0.031 | 0.219 | 0.139 | 0.385 | 0.000 | 853 | 399 |
| GCC | 0.105 | 0.064 | -0.020 | 0.230 | 0.100 | 0.385 | 0.000 | 853 | 399 |
| IC | 0.014 | 0.064 | -0.111 | 0.139 | 0.822 | 0.856 | 0.000 | 853 | 399 |
| IFO | 0.091 | 0.064 | -0.034 | 0.217 | 0.152 | 0.385 | 0.000 | 853 | 399 |
| PCR | 0.109 | 0.076 | -0.041 | 0.258 | 0.154 | 0.385 | 15.858 | 853 | 399 |
| PLIC | -0.018 | 0.064 | -0.143 | 0.107 | 0.775 | 0.842 | 0.000 | 853 | 399 |
| PTR | 0.068 | 0.134 | -0.195 | 0.331 | 0.612 | 0.729 | 69.298 | 853 | 399 |
| RLIC | 0.027 | 0.070 | -0.110 | 0.165 | 0.697 | 0.792 | 8.116 | 853 | 399 |
| SCC | 0.062 | 0.064 | -0.063 | 0.187 | 0.333 | 0.523 | 0.001 | 853 | 399 |
| SCR | 0.140 | 0.064 | 0.015 | 0.265 | 0.029 | 0.385 | 0.009 | 853 | 399 |
| SFO | 0.074 | 0.077 | -0.077 | 0.226 | 0.335 | 0.523 | 17.181 | 853 | 399 |
| SLF | 0.078 | 0.071 | -0.061 | 0.217 | 0.271 | 0.484 | 8.748 | 853 | 399 |
| SS | 0.073 | 0.091 | -0.105 | 0.252 | 0.421 | 0.619 | 34.677 | 853 | 399 |
| UNC | -0.016 | 0.145 | -0.300 | 0.268 | 0.912 | 0.912 | 73.965 | 853 | 399 |
| AverageMD | 0.133 | 0.075 | -0.014 | 0.281 | 0.077 | 0.385 | 14.760 | 853 | 399 |

Table S63. Cohen’s d effect sizes after meta-analysis, for RD differences between adult age of onset MDD patients (age ≥ 22 years) and healthy controls **in adults only**. Age, sex, agexsex, age^2^, age^2^xsex and scansite included as covariates.

| Region | Cohen’s d | SE | CI LB | CI UB | P-value | FDR P-value | I^2^ | Controls | Patients |
| --- | --- | --- | --- | --- | --- | --- | --- | --- | --- |
| ACR | 0.121 | 0.090 | -0.055 | 0.296 | 0.178 | 0.296 | 32.910 | 853 | 399 |
| ALIC | 0.094 | 0.064 | -0.031 | 0.219 | 0.139 | 0.248 | 0.000 | 853 | 399 |
| BCC | 0.131 | 0.081 | -0.029 | 0.291 | 0.108 | 0.238 | 22.456 | 853 | 399 |
| CC | 0.150 | 0.079 | -0.005 | 0.306 | 0.058 | 0.190 | 19.557 | 853 | 399 |
| CGC | 0.127 | 0.064 | 0.001 | 0.252 | 0.048 | 0.190 | 0.274 | 853 | 399 |
| CGH | 0.078 | 0.064 | -0.046 | 0.203 | 0.218 | 0.303 | 0.000 | 853 | 399 |
| CR | 0.142 | 0.064 | 0.016 | 0.267 | 0.027 | 0.190 | 0.008 | 853 | 399 |
| CST | 0.189 | 0.103 | -0.013 | 0.391 | 0.067 | 0.190 | 47.540 | 853 | 399 |
| EC | 0.161 | 0.124 | -0.083 | 0.404 | 0.197 | 0.296 | 63.966 | 853 | 399 |
| FX | 0.042 | 0.064 | -0.083 | 0.167 | 0.506 | 0.575 | 0.000 | 853 | 399 |
| FXST | 0.128 | 0.072 | -0.013 | 0.270 | 0.075 | 0.190 | 10.406 | 853 | 399 |
| GCC | 0.128 | 0.064 | 0.002 | 0.253 | 0.046 | 0.190 | 0.007 | 853 | 399 |
| IC | 0.060 | 0.064 | -0.065 | 0.185 | 0.345 | 0.431 | 0.000 | 853 | 399 |
| IFO | 0.154 | 0.097 | -0.037 | 0.344 | 0.114 | 0.238 | 41.491 | 853 | 399 |
| PCR | 0.127 | 0.099 | -0.068 | 0.322 | 0.201 | 0.296 | 43.968 | 853 | 399 |
| PLIC | 0.014 | 0.064 | -0.111 | 0.139 | 0.822 | 0.856 | 0.000 | 853 | 399 |
| PTR | 0.023 | 0.148 | -0.268 | 0.314 | 0.875 | 0.875 | 75.302 | 853 | 399 |
| RLIC | 0.049 | 0.064 | -0.076 | 0.174 | 0.441 | 0.525 | 0.008 | 853 | 399 |
| SCC | 0.213 | 0.105 | 0.008 | 0.418 | 0.042 | 0.190 | 48.556 | 853 | 399 |
| SCR | 0.159 | 0.064 | 0.034 | 0.284 | 0.012 | 0.190 | 0.000 | 853 | 399 |
| SFO | 0.113 | 0.064 | -0.012 | 0.238 | 0.076 | 0.190 | 0.004 | 853 | 399 |
| SLF | 0.115 | 0.075 | -0.033 | 0.262 | 0.128 | 0.246 | 14.342 | 853 | 399 |
| SS | 0.122 | 0.114 | -0.100 | 0.345 | 0.281 | 0.370 | 56.529 | 853 | 399 |
| UNC | -0.079 | 0.129 | -0.331 | 0.174 | 0.542 | 0.589 | 66.702 | 853 | 399 |
| AverageRD | 0.149 | 0.076 | 0.001 | 0.298 | 0.048 | 0.190 | 15.015 | 853 | 399 |

Table S64. Cohen’s d effect sizes after meta-analysis, for FA differences between adolescent age of onset MDD patients (age < 22 years) and adult age of onset MDD patients (age ≥ 22 years) **in adults only**. Age, sex, agexsex, age^2^, age^2^xsex and scansite included as covariates.

| Region | Cohen’s d | SE | CI LB | CI UB | P-value | FDR P-value | I^2^ | Controls | Patients |
| --- | --- | --- | --- | --- | --- | --- | --- | --- | --- |
| ACR | 0.115 | 0.092 | -0.065 | 0.294 | 0.210 | 0.924 | 0.000 | 197 | 334 |
| ALIC | 0.108 | 0.112 | -0.112 | 0.327 | 0.335 | 0.924 | 24.357 | 197 | 334 |
| BCC | 0.121 | 0.136 | -0.146 | 0.389 | 0.375 | 0.924 | 46.116 | 197 | 334 |
| CC | 0.058 | 0.117 | -0.172 | 0.288 | 0.622 | 0.924 | 29.626 | 197 | 334 |
| CGC | 0.013 | 0.119 | -0.221 | 0.247 | 0.914 | 0.924 | 31.412 | 197 | 334 |
| CGH | -0.029 | 0.150 | -0.323 | 0.264 | 0.845 | 0.924 | 55.012 | 197 | 334 |
| CR | 0.064 | 0.092 | -0.115 | 0.243 | 0.484 | 0.924 | 0.003 | 197 | 334 |
| CST | -0.049 | 0.091 | -0.228 | 0.130 | 0.593 | 0.924 | 0.001 | 197 | 334 |
| EC | -0.111 | 0.091 | -0.290 | 0.068 | 0.224 | 0.924 | 0.000 | 197 | 334 |
| FX | -0.021 | 0.097 | -0.211 | 0.169 | 0.827 | 0.924 | 6.788 | 197 | 334 |
| FXST | 0.014 | 0.092 | -0.165 | 0.193 | 0.878 | 0.924 | 0.004 | 197 | 334 |
| GCC | 0.074 | 0.091 | -0.106 | 0.253 | 0.421 | 0.924 | 0.001 | 197 | 334 |
| IC | 0.158 | 0.116 | -0.070 | 0.386 | 0.174 | 0.924 | 28.351 | 197 | 334 |
| IFO | -0.246 | 0.092 | -0.426 | -0.066 | 0.007 | 0.175 | 0.000 | 197 | 334 |
| PCR | -0.038 | 0.092 | -0.217 | 0.141 | 0.679 | 0.924 | 0.005 | 197 | 334 |
| PLIC | 0.157 | 0.096 | -0.031 | 0.344 | 0.101 | 0.924 | 5.110 | 197 | 334 |
| PTR | 0.063 | 0.125 | -0.181 | 0.308 | 0.612 | 0.924 | 36.868 | 197 | 334 |
| RLIC | 0.069 | 0.098 | -0.123 | 0.261 | 0.483 | 0.924 | 8.076 | 197 | 334 |
| SCC | -0.102 | 0.113 | -0.324 | 0.120 | 0.366 | 0.924 | 25.128 | 197 | 334 |
| SCR | 0.032 | 0.091 | -0.147 | 0.211 | 0.727 | 0.924 | 0.001 | 197 | 334 |
| SFO | 0.009 | 0.091 | -0.170 | 0.188 | 0.924 | 0.924 | 0.000 | 197 | 334 |
| SLF | -0.031 | 0.135 | -0.295 | 0.234 | 0.820 | 0.924 | 44.831 | 197 | 334 |
| SS | 0.026 | 0.091 | -0.153 | 0.205 | 0.778 | 0.924 | 0.000 | 197 | 334 |
| UNC | -0.044 | 0.231 | -0.496 | 0.409 | 0.850 | 0.924 | 81.071 | 197 | 334 |
| AverageFA | 0.032 | 0.091 | -0.147 | 0.211 | 0.729 | 0.924 | 0.007 | 197 | 334 |

Table S65. Cohen’s d effect sizes after meta-analysis, for AD differences between adolescent age of onset MDD patients (age < 22 years) and adult age of onset MDD patients (age ≥ 22 years) **in adults only**. Age, sex, agexsex, age^2^, age^2^xsex and scansite included as covariates.

| Region | Cohen’s d | SE | CI LB | CI UB | P-value | FDR P-value | I^2^ | Controls | Patients |
| --- | --- | --- | --- | --- | --- | --- | --- | --- | --- |
| ACR | 0.035 | 0.091 | -0.144 | 0.214 | 0.698 | 0.855 | 0.000 | 197 | 334 |
| ALIC | 0.182 | 0.116 | -0.045 | 0.408 | 0.116 | 0.464 | 27.747 | 197 | 334 |
| BCC | 0.144 | 0.134 | -0.119 | 0.407 | 0.283 | 0.708 | 44.465 | 197 | 334 |
| CC | 0.139 | 0.092 | -0.041 | 0.318 | 0.130 | 0.464 | 0.008 | 197 | 334 |
| CGC | 0.187 | 0.092 | 0.007 | 0.366 | 0.042 | 0.464 | 0.001 | 197 | 334 |
| CGH | 0.032 | 0.091 | -0.147 | 0.211 | 0.726 | 0.855 | 0.000 | 197 | 334 |
| CR | 0.043 | 0.091 | -0.136 | 0.222 | 0.639 | 0.855 | 0.000 | 197 | 334 |
| CST | -0.124 | 0.127 | -0.374 | 0.125 | 0.329 | 0.748 | 39.186 | 197 | 334 |
| EC | 0.029 | 0.091 | -0.150 | 0.208 | 0.752 | 0.855 | 0.009 | 197 | 334 |
| FX | -0.071 | 0.091 | -0.250 | 0.108 | 0.439 | 0.855 | 0.007 | 197 | 334 |
| FXST | -0.048 | 0.135 | -0.311 | 0.216 | 0.723 | 0.855 | 44.814 | 197 | 334 |
| GCC | 0.059 | 0.091 | -0.120 | 0.238 | 0.518 | 0.855 | 0.007 | 197 | 334 |
| IC | 0.112 | 0.091 | -0.067 | 0.291 | 0.221 | 0.691 | 0.000 | 197 | 334 |
| IFO | -0.247 | 0.138 | -0.518 | 0.024 | 0.074 | 0.464 | 47.397 | 197 | 334 |
| PCR | 0.015 | 0.118 | -0.216 | 0.247 | 0.897 | 0.934 | 30.544 | 197 | 334 |
| PLIC | 0.003 | 0.091 | -0.176 | 0.182 | 0.970 | 0.970 | 0.000 | 197 | 334 |
| PTR | 0.047 | 0.102 | -0.153 | 0.246 | 0.645 | 0.855 | 12.574 | 197 | 334 |
| RLIC | 0.168 | 0.091 | -0.011 | 0.348 | 0.066 | 0.464 | 0.000 | 197 | 334 |
| SCC | 0.148 | 0.091 | -0.031 | 0.328 | 0.104 | 0.464 | 0.000 | 197 | 334 |
| SCR | 0.054 | 0.091 | -0.124 | 0.233 | 0.551 | 0.855 | 0.000 | 197 | 334 |
| SFO | 0.099 | 0.091 | -0.080 | 0.279 | 0.277 | 0.708 | 0.000 | 197 | 334 |
| SLF | 0.069 | 0.113 | -0.152 | 0.290 | 0.542 | 0.855 | 24.996 | 197 | 334 |
| SS | 0.020 | 0.122 | -0.218 | 0.258 | 0.867 | 0.934 | 33.592 | 197 | 334 |
| UNC | -0.156 | 0.092 | -0.335 | 0.024 | 0.089 | 0.464 | 0.003 | 197 | 334 |
| AverageAD | 0.064 | 0.091 | -0.115 | 0.243 | 0.487 | 0.855 | 0.000 | 197 | 334 |

Table S66. Cohen’s d effect sizes after meta-analysis, for MD differences between adolescent age of onset MDD patients (age < 22 years) and adult age of onset MDD patients (age ≥ 22 years) **in adults only**. Age, sex, agexsex, age^2^, age^2^xsex and scansite included as covariates.

| Region | Cohen’s d | SE | CI LB | CI UB | P-value | FDR P-value | I^2^ | Controls | Patients |
| --- | --- | --- | --- | --- | --- | --- | --- | --- | --- |
| ACR | -0.032 | 0.091 | -0.211 | 0.147 | 0.724 | 0.957 | 0.000 | 197 | 334 |
| ALIC | 0.112 | 0.091 | -0.067 | 0.291 | 0.219 | 0.957 | 0.000 | 197 | 334 |
| BCC | -0.045 | 0.091 | -0.224 | 0.134 | 0.623 | 0.957 | 0.000 | 197 | 334 |
| CC | 0.015 | 0.091 | -0.164 | 0.194 | 0.871 | 0.957 | 0.000 | 197 | 334 |
| CGC | 0.036 | 0.091 | -0.143 | 0.215 | 0.694 | 0.957 | 0.000 | 197 | 334 |
| CGH | -0.046 | 0.139 | -0.319 | 0.227 | 0.739 | 0.957 | 48.311 | 197 | 334 |
| CR | -0.045 | 0.091 | -0.224 | 0.134 | 0.624 | 0.957 | 0.000 | 197 | 334 |
| CST | -0.157 | 0.091 | -0.336 | 0.022 | 0.086 | 0.957 | 0.000 | 197 | 334 |
| EC | 0.122 | 0.091 | -0.057 | 0.301 | 0.181 | 0.957 | 0.000 | 197 | 334 |
| FX | -0.014 | 0.122 | -0.253 | 0.226 | 0.911 | 0.957 | 34.401 | 197 | 334 |
| FXST | -0.056 | 0.125 | -0.301 | 0.190 | 0.657 | 0.957 | 37.235 | 197 | 334 |
| GCC | 0.009 | 0.091 | -0.169 | 0.188 | 0.919 | 0.957 | 0.000 | 197 | 334 |
| IC | 0.074 | 0.091 | -0.105 | 0.253 | 0.419 | 0.957 | 0.000 | 197 | 334 |
| IFO | -0.016 | 0.143 | -0.297 | 0.265 | 0.913 | 0.957 | 51.014 | 197 | 334 |
| PCR | -0.052 | 0.091 | -0.231 | 0.127 | 0.570 | 0.957 | 0.000 | 197 | 334 |
| PLIC | -0.024 | 0.091 | -0.203 | 0.155 | 0.796 | 0.957 | 0.000 | 197 | 334 |
| PTR | -0.042 | 0.091 | -0.221 | 0.137 | 0.645 | 0.957 | 0.000 | 197 | 334 |
| RLIC | 0.125 | 0.092 | -0.055 | 0.304 | 0.173 | 0.957 | 0.007 | 197 | 334 |
| SCC | 0.114 | 0.091 | -0.065 | 0.293 | 0.214 | 0.957 | 0.000 | 197 | 334 |
| SCR | -0.046 | 0.091 | -0.225 | 0.132 | 0.610 | 0.957 | 0.000 | 197 | 334 |
| SFO | 0.056 | 0.091 | -0.123 | 0.235 | 0.540 | 0.957 | 0.000 | 197 | 334 |
| SLF | -0.021 | 0.099 | -0.214 | 0.173 | 0.832 | 0.957 | 9.033 | 197 | 334 |
| SS | -0.022 | 0.091 | -0.201 | 0.157 | 0.807 | 0.957 | 0.005 | 197 | 334 |
| UNC | -0.192 | 0.165 | -0.516 | 0.131 | 0.244 | 0.957 | 62.618 | 197 | 334 |
| AverageMD | -0.004 | 0.091 | -0.182 | 0.175 | 0.969 | 0.969 | 0.000 | 197 | 334 |

Table S67. Cohen’s d effect sizes after meta-analysis, for RD differences between adolescent age of onset MDD patients (age < 22 years) and adult age of onset MDD patients (age ≥ 22 years) **in adults only**. Age, sex, agexsex, age^2^, age^2^xsex and scansite included as covariates.

| Region | Cohen’s d | SE | CI LB | CI UB | P-value | FDR P-value | I^2^ | Controls | Patients |
| --- | --- | --- | --- | --- | --- | --- | --- | --- | --- |
| ACR | -0.092 | 0.091 | -0.271 | 0.087 | 0.316 | 0.901 | 0.000 | 197 | 334 |
| ALIC | 0.029 | 0.091 | -0.150 | 0.208 | 0.750 | 0.901 | 0.000 | 197 | 334 |
| BCC | -0.098 | 0.106 | -0.305 | 0.110 | 0.356 | 0.901 | 17.210 | 197 | 334 |
| CC | -0.030 | 0.091 | -0.209 | 0.149 | 0.742 | 0.901 | 0.000 | 197 | 334 |
| CGC | -0.020 | 0.092 | -0.199 | 0.159 | 0.826 | 0.901 | 0.001 | 197 | 334 |
| CGH | -0.127 | 0.156 | -0.434 | 0.179 | 0.416 | 0.901 | 58.696 | 197 | 334 |
| CR | -0.073 | 0.091 | -0.252 | 0.106 | 0.422 | 0.901 | 0.000 | 197 | 334 |
| CST | -0.111 | 0.091 | -0.290 | 0.068 | 0.225 | 0.901 | 0.002 | 197 | 334 |
| EC | 0.126 | 0.091 | -0.053 | 0.306 | 0.167 | 0.901 | 0.000 | 197 | 334 |
| FX | -0.006 | 0.121 | -0.244 | 0.231 | 0.958 | 0.958 | 33.657 | 197 | 334 |
| FXST | -0.140 | 0.132 | -0.399 | 0.118 | 0.287 | 0.901 | 42.593 | 197 | 334 |
| GCC | -0.040 | 0.091 | -0.219 | 0.139 | 0.662 | 0.901 | 0.000 | 197 | 334 |
| IC | -0.020 | 0.091 | -0.199 | 0.159 | 0.829 | 0.901 | 0.000 | 197 | 334 |
| IFO | 0.111 | 0.091 | -0.069 | 0.290 | 0.226 | 0.901 | 0.000 | 197 | 334 |
| PCR | -0.040 | 0.091 | -0.220 | 0.139 | 0.658 | 0.901 | 0.006 | 197 | 334 |
| PLIC | -0.079 | 0.091 | -0.258 | 0.100 | 0.388 | 0.901 | 0.012 | 197 | 334 |
| PTR | -0.051 | 0.106 | -0.258 | 0.156 | 0.629 | 0.901 | 17.284 | 197 | 334 |
| RLIC | 0.006 | 0.102 | -0.195 | 0.206 | 0.957 | 0.958 | 13.054 | 197 | 334 |
| SCC | 0.110 | 0.092 | -0.069 | 0.289 | 0.229 | 0.901 | 0.004 | 197 | 334 |
| SCR | -0.048 | 0.091 | -0.227 | 0.131 | 0.602 | 0.901 | 0.000 | 197 | 334 |
| SFO | 0.020 | 0.091 | -0.159 | 0.199 | 0.828 | 0.901 | 0.000 | 197 | 334 |
| SLF | -0.069 | 0.120 | -0.305 | 0.166 | 0.565 | 0.901 | 32.416 | 197 | 334 |
| SS | -0.043 | 0.091 | -0.222 | 0.136 | 0.639 | 0.901 | 0.000 | 197 | 334 |
| UNC | -0.219 | 0.195 | -0.601 | 0.164 | 0.263 | 0.901 | 73.264 | 197 | 334 |
| AverageRD | -0.029 | 0.091 | -0.208 | 0.150 | 0.750 | 0.901 | 0.000 | 197 | 334 |

**7: Antidepressant use at time of scanning**

Table S68. Cohen’s d effect sizes after meta-analysis, for FA differences between antidepressant users and healthy controls **in adults only**. Age, sex, agexsex, age^2^, age^2^xsex and scansite included as covariates.

| Region | Cohen’s d | SE | CI LB | CI UB | P-value | FDR P-value | I^2^ | Controls | Patients |
| --- | --- | --- | --- | --- | --- | --- | --- | --- | --- |
| ACR | -0.170 | 0.062 | -0.293 | -0.048 | 0.006 | 0.125 | 0.000 | 848 | 406 |
| ALIC | -0.074 | 0.062 | -0.196 | 0.049 | 0.237 | 0.349 | 0.000 | 848 | 406 |
| BCC | -0.156 | 0.084 | -0.321 | 0.010 | 0.065 | 0.148 | 32.069 | 848 | 406 |
| CC | -0.136 | 0.072 | -0.278 | 0.006 | 0.061 | 0.148 | 15.383 | 848 | 406 |
| CGC | -0.123 | 0.062 | -0.245 | 0.000 | 0.049 | 0.148 | 0.000 | 848 | 406 |
| CGH | -0.036 | 0.062 | -0.158 | 0.087 | 0.569 | 0.625 | 0.001 | 848 | 406 |
| CR | -0.148 | 0.062 | -0.270 | -0.025 | 0.018 | 0.125 | 0.000 | 848 | 406 |
| CST | -0.144 | 0.125 | -0.389 | 0.101 | 0.251 | 0.349 | 67.328 | 848 | 406 |
| EC | -0.185 | 0.089 | -0.360 | -0.010 | 0.038 | 0.146 | 37.666 | 848 | 406 |
| FX | -0.043 | 0.076 | -0.193 | 0.107 | 0.575 | 0.625 | 21.201 | 848 | 406 |
| FXST | -0.082 | 0.107 | -0.291 | 0.127 | 0.442 | 0.526 | 55.121 | 848 | 406 |
| GCC | -0.160 | 0.063 | -0.284 | -0.036 | 0.011 | 0.125 | 1.111 | 848 | 406 |
| IC | -0.091 | 0.062 | -0.213 | 0.031 | 0.145 | 0.259 | 0.014 | 848 | 406 |
| IFO | -0.111 | 0.087 | -0.283 | 0.060 | 0.203 | 0.317 | 35.693 | 848 | 406 |
| PCR | -0.128 | 0.062 | -0.250 | -0.005 | 0.041 | 0.146 | 0.024 | 848 | 406 |
| PLIC | -0.050 | 0.062 | -0.172 | 0.072 | 0.421 | 0.526 | 0.000 | 848 | 406 |
| PTR | -0.214 | 0.092 | -0.395 | -0.034 | 0.020 | 0.125 | 40.825 | 848 | 406 |
| RLIC | -0.091 | 0.062 | -0.214 | 0.031 | 0.143 | 0.259 | 0.000 | 848 | 406 |
| SCC | -0.001 | 0.062 | -0.123 | 0.121 | 0.989 | 0.989 | 0.000 | 848 | 406 |
| SCR | -0.069 | 0.062 | -0.191 | 0.053 | 0.267 | 0.351 | 0.000 | 848 | 406 |
| SFO | -0.082 | 0.062 | -0.204 | 0.040 | 0.189 | 0.315 | 0.000 | 848 | 406 |
| SLF | -0.116 | 0.062 | -0.238 | 0.007 | 0.064 | 0.148 | 0.003 | 848 | 406 |
| SS | -0.220 | 0.123 | -0.462 | 0.022 | 0.074 | 0.154 | 66.284 | 848 | 406 |
| UNC | -0.028 | 0.116 | -0.255 | 0.199 | 0.808 | 0.842 | 61.825 | 848 | 406 |
| AverageFA | -0.129 | 0.062 | -0.251 | -0.006 | 0.039 | 0.146 | 0.000 | 848 | 406 |

Table S69. Cohen’s d effect sizes after meta-analysis, for AD differences between antidepressant users and healthy controls **in adults only**. Age, sex, agexsex, age^2^, age^2^xsex and scansite included as covariates.

| Region | Cohen’s d | SE | CI LB | CI UB | P-value | FDR P-value | I^2^ | Controls | Patients |
| --- | --- | --- | --- | --- | --- | --- | --- | --- | --- |
| ACR | -0.058 | 0.097 | -0.248 | 0.132 | 0.547 | 0.937 | 46.452 | 848 | 406 |
| ALIC | -0.035 | 0.097 | -0.224 | 0.154 | 0.717 | 0.937 | 46.034 | 848 | 406 |
| BCC | -0.036 | 0.072 | -0.176 | 0.105 | 0.618 | 0.937 | 13.961 | 848 | 406 |
| CC | -0.053 | 0.062 | -0.176 | 0.069 | 0.392 | 0.937 | 0.000 | 848 | 406 |
| CGC | -0.073 | 0.091 | -0.251 | 0.105 | 0.423 | 0.937 | 39.997 | 848 | 406 |
| CGH | 0.058 | 0.098 | -0.134 | 0.249 | 0.555 | 0.937 | 47.227 | 848 | 406 |
| CR | 0.036 | 0.065 | -0.090 | 0.163 | 0.575 | 0.937 | 3.470 | 848 | 406 |
| CST | 0.110 | 0.109 | -0.105 | 0.324 | 0.316 | 0.937 | 57.031 | 848 | 406 |
| EC | -0.008 | 0.102 | -0.208 | 0.191 | 0.934 | 0.937 | 51.188 | 848 | 406 |
| FX | -0.009 | 0.062 | -0.132 | 0.113 | 0.880 | 0.937 | 0.000 | 848 | 406 |
| FXST | 0.040 | 0.073 | -0.103 | 0.184 | 0.582 | 0.937 | 16.830 | 848 | 406 |
| GCC | -0.090 | 0.099 | -0.284 | 0.103 | 0.360 | 0.937 | 48.284 | 848 | 406 |
| IC | -0.028 | 0.099 | -0.221 | 0.165 | 0.775 | 0.937 | 48.269 | 848 | 406 |
| IFO | 0.037 | 0.093 | -0.145 | 0.218 | 0.690 | 0.937 | 41.925 | 848 | 406 |
| PCR | -0.046 | 0.062 | -0.169 | 0.076 | 0.456 | 0.937 | 0.000 | 848 | 406 |
| PLIC | 0.060 | 0.097 | -0.129 | 0.250 | 0.533 | 0.937 | 46.376 | 848 | 406 |
| PTR | -0.055 | 0.130 | -0.309 | 0.199 | 0.670 | 0.937 | 69.739 | 848 | 406 |
| RLIC | -0.056 | 0.082 | -0.217 | 0.105 | 0.497 | 0.937 | 29.384 | 848 | 406 |
| SCC | -0.085 | 0.062 | -0.207 | 0.037 | 0.174 | 0.937 | 0.000 | 848 | 406 |
| SCR | 0.120 | 0.062 | -0.003 | 0.242 | 0.055 | 0.937 | 0.000 | 848 | 406 |
| SFO | -0.013 | 0.067 | -0.145 | 0.118 | 0.843 | 0.937 | 7.296 | 848 | 406 |
| SLF | -0.066 | 0.062 | -0.188 | 0.056 | 0.289 | 0.937 | 0.000 | 848 | 406 |
| SS | -0.040 | 0.129 | -0.293 | 0.212 | 0.755 | 0.937 | 69.403 | 848 | 406 |
| UNC | 0.010 | 0.129 | -0.242 | 0.263 | 0.937 | 0.937 | 69.375 | 848 | 406 |
| AverageAD | 0.008 | 0.073 | -0.134 | 0.151 | 0.907 | 0.937 | 16.121 | 848 | 406 |

Table S70. Cohen’s d effect sizes after meta-analysis, for MD differences between antidepressant users and healthy controls **in adults only**. Age, sex, agexsex, age^2^, age^2^xsex and scansite included as covariates.

| Region | Cohen’s d | SE | CI LB | CI UB | P-value | FDR P-value | I^2^ | Controls | Patients |
| --- | --- | --- | --- | --- | --- | --- | --- | --- | --- |
| ACR | 0.029 | 0.062 | -0.094 | 0.151 | 0.647 | 0.985 | 0.000 | 848 | 406 |
| ALIC | -0.009 | 0.062 | -0.131 | 0.113 | 0.886 | 0.985 | 0.000 | 848 | 406 |
| BCC | 0.064 | 0.062 | -0.058 | 0.187 | 0.303 | 0.881 | 0.000 | 848 | 406 |
| CC | 0.017 | 0.062 | -0.105 | 0.139 | 0.784 | 0.985 | 0.000 | 848 | 406 |
| CGC | -0.005 | 0.062 | -0.127 | 0.118 | 0.941 | 0.985 | 0.006 | 848 | 406 |
| CGH | -0.004 | 0.076 | -0.153 | 0.145 | 0.958 | 0.985 | 20.753 | 848 | 406 |
| CR | 0.072 | 0.062 | -0.050 | 0.195 | 0.246 | 0.881 | 0.000 | 848 | 406 |
| CST | 0.097 | 0.117 | -0.132 | 0.326 | 0.406 | 0.881 | 62.567 | 848 | 406 |
| EC | 0.021 | 0.106 | -0.188 | 0.229 | 0.847 | 0.985 | 55.047 | 848 | 406 |
| FX | -0.054 | 0.062 | -0.177 | 0.068 | 0.382 | 0.881 | 0.000 | 848 | 406 |
| FXST | 0.050 | 0.062 | -0.073 | 0.172 | 0.425 | 0.881 | 0.001 | 848 | 406 |
| GCC | -0.001 | 0.062 | -0.123 | 0.121 | 0.985 | 0.985 | 0.014 | 848 | 406 |
| IC | -0.052 | 0.062 | -0.174 | 0.070 | 0.404 | 0.881 | 0.000 | 848 | 406 |
| IFO | -0.013 | 0.092 | -0.193 | 0.167 | 0.890 | 0.985 | 40.952 | 848 | 406 |
| PCR | 0.087 | 0.062 | -0.036 | 0.209 | 0.165 | 0.881 | 0.000 | 848 | 406 |
| PLIC | -0.073 | 0.062 | -0.196 | 0.049 | 0.240 | 0.881 | 0.000 | 848 | 406 |
| PTR | 0.097 | 0.128 | -0.155 | 0.348 | 0.451 | 0.881 | 69.090 | 848 | 406 |
| RLIC | -0.036 | 0.062 | -0.158 | 0.086 | 0.562 | 0.977 | 0.000 | 848 | 406 |
| SCC | -0.053 | 0.062 | -0.175 | 0.070 | 0.399 | 0.881 | 0.000 | 848 | 406 |
| SCR | 0.123 | 0.062 | 0.001 | 0.245 | 0.049 | 0.881 | 0.000 | 848 | 406 |
| SFO | 0.004 | 0.098 | -0.187 | 0.196 | 0.967 | 0.985 | 47.106 | 848 | 406 |
| SLF | 0.034 | 0.062 | -0.088 | 0.156 | 0.586 | 0.977 | 0.000 | 848 | 406 |
| SS | 0.067 | 0.062 | -0.055 | 0.189 | 0.282 | 0.881 | 0.000 | 848 | 406 |
| UNC | -0.017 | 0.134 | -0.280 | 0.246 | 0.899 | 0.985 | 71.952 | 848 | 406 |
| AverageMD | 0.046 | 0.062 | -0.076 | 0.168 | 0.458 | 0.881 | 0.000 | 848 | 406 |

Table S71. Cohen’s d effect sizes after meta-analysis, for RD differences between antidepressant users and healthy controls **in adults only**. Age, sex, agexsex, age^2^, age^2^xsex and scansite included as covariates.

| Region | Cohen’s d | SE | CI LB | CI UB | P-value | FDR P-value | I^2^ | Controls | Patients |
| --- | --- | --- | --- | --- | --- | --- | --- | --- | --- |
| ACR | 0.070 | 0.062 | -0.052 | 0.193 | 0.259 | 0.785 | 0.000 | 848 | 406 |
| ALIC | 0.049 | 0.062 | -0.073 | 0.171 | 0.434 | 0.785 | 0.000 | 848 | 406 |
| BCC | 0.065 | 0.062 | -0.057 | 0.187 | 0.297 | 0.785 | 0.000 | 848 | 406 |
| CC | 0.045 | 0.062 | -0.077 | 0.167 | 0.471 | 0.785 | 0.000 | 848 | 406 |
| CGC | 0.035 | 0.062 | -0.088 | 0.157 | 0.579 | 0.905 | 0.000 | 848 | 406 |
| CGH | 0.004 | 0.062 | -0.118 | 0.127 | 0.946 | 0.974 | 0.003 | 848 | 406 |
| CR | 0.105 | 0.062 | -0.017 | 0.227 | 0.092 | 0.767 | 0.000 | 848 | 406 |
| CST | 0.157 | 0.120 | -0.079 | 0.393 | 0.193 | 0.785 | 64.692 | 848 | 406 |
| EC | 0.037 | 0.118 | -0.193 | 0.268 | 0.752 | 0.965 | 63.188 | 848 | 406 |
| FX | -0.049 | 0.062 | -0.171 | 0.073 | 0.433 | 0.785 | 0.000 | 848 | 406 |
| FXST | 0.056 | 0.077 | -0.095 | 0.207 | 0.467 | 0.785 | 22.230 | 848 | 406 |
| GCC | 0.048 | 0.062 | -0.074 | 0.170 | 0.441 | 0.785 | 0.000 | 848 | 406 |
| IC | 0.024 | 0.062 | -0.099 | 0.146 | 0.706 | 0.965 | 0.001 | 848 | 406 |
| IFO | -0.004 | 0.111 | -0.222 | 0.214 | 0.974 | 0.974 | 58.769 | 848 | 406 |
| PCR | 0.111 | 0.062 | -0.011 | 0.233 | 0.075 | 0.767 | 0.000 | 848 | 406 |
| PLIC | -0.014 | 0.062 | -0.136 | 0.108 | 0.825 | 0.965 | 0.006 | 848 | 406 |
| PTR | 0.117 | 0.131 | -0.139 | 0.373 | 0.370 | 0.785 | 70.212 | 848 | 406 |
| RLIC | 0.009 | 0.062 | -0.114 | 0.131 | 0.889 | 0.966 | 0.008 | 848 | 406 |
| SCC | -0.012 | 0.062 | -0.134 | 0.110 | 0.849 | 0.965 | 0.000 | 848 | 406 |
| SCR | 0.124 | 0.062 | 0.002 | 0.247 | 0.046 | 0.767 | 0.000 | 848 | 406 |
| SFO | 0.023 | 0.095 | -0.162 | 0.208 | 0.808 | 0.965 | 43.903 | 848 | 406 |
| SLF | 0.071 | 0.062 | -0.051 | 0.193 | 0.254 | 0.785 | 0.000 | 848 | 406 |
| SS | 0.077 | 0.082 | -0.084 | 0.238 | 0.349 | 0.785 | 28.902 | 848 | 406 |
| UNC | -0.060 | 0.131 | -0.316 | 0.196 | 0.646 | 0.950 | 70.292 | 848 | 406 |
| AverageRD | 0.071 | 0.062 | -0.051 | 0.193 | 0.256 | 0.785 | 0.017 | 848 | 406 |

Table S72. Cohen’s d effect sizes after meta-analysis, for FA differences between non-antidepressant users and healthy controls **in adults only**. Age, sex, agexsex, age^2^, age^2^xsex and scansite included as covariates.

| Region | Cohen’s d | SE | CI LB | CI UB | P-value | FDR P-value | I^2^ | Controls | Patients |
| --- | --- | --- | --- | --- | --- | --- | --- | --- | --- |
| ACR | -0.237 | 0.113 | -0.459 | -0.016 | 0.035 | 0.109 | 45.969 | 962 | 288 |
| ALIC | -0.242 | 0.113 | -0.464 | -0.021 | 0.032 | 0.109 | 45.853 | 962 | 288 |
| BCC | -0.201 | 0.119 | -0.434 | 0.033 | 0.092 | 0.179 | 51.389 | 962 | 288 |
| CC | -0.196 | 0.119 | -0.429 | 0.038 | 0.100 | 0.179 | 51.103 | 962 | 288 |
| CGC | -0.289 | 0.163 | -0.609 | 0.031 | 0.077 | 0.179 | 73.934 | 962 | 288 |
| CGH | -0.247 | 0.097 | -0.437 | -0.058 | 0.010 | 0.042 | 27.930 | 962 | 288 |
| CR | -0.242 | 0.119 | -0.476 | -0.008 | 0.042 | 0.117 | 51.554 | 962 | 288 |
| CST | -0.117 | 0.089 | -0.292 | 0.057 | 0.188 | 0.233 | 18.216 | 962 | 288 |
| EC | -0.212 | 0.075 | -0.360 | -0.064 | 0.005 | 0.030 | 0.002 | 962 | 288 |
| FX | -0.241 | 0.087 | -0.412 | -0.069 | 0.006 | 0.030 | 15.682 | 962 | 288 |
| FXST | -0.274 | 0.076 | -0.422 | -0.126 | 0.000 | 0.000 | 0.000 | 962 | 288 |
| GCC | -0.145 | 0.108 | -0.356 | 0.066 | 0.177 | 0.233 | 40.810 | 962 | 288 |
| IC | -0.187 | 0.108 | -0.399 | 0.025 | 0.084 | 0.179 | 41.123 | 962 | 288 |
| IFO | -0.113 | 0.075 | -0.261 | 0.035 | 0.135 | 0.199 | 0.002 | 962 | 288 |
| PCR | -0.193 | 0.116 | -0.420 | 0.034 | 0.095 | 0.179 | 48.438 | 962 | 288 |
| PLIC | -0.096 | 0.119 | -0.329 | 0.137 | 0.420 | 0.457 | 51.170 | 962 | 288 |
| PTR | -0.060 | 0.100 | -0.256 | 0.137 | 0.551 | 0.574 | 32.432 | 962 | 288 |
| RLIC | -0.126 | 0.080 | -0.282 | 0.030 | 0.113 | 0.188 | 5.355 | 962 | 288 |
| SCC | -0.075 | 0.075 | -0.223 | 0.073 | 0.318 | 0.361 | 0.001 | 962 | 288 |
| SCR | -0.152 | 0.118 | -0.383 | 0.079 | 0.196 | 0.233 | 50.011 | 962 | 288 |
| SFO | -0.129 | 0.086 | -0.298 | 0.040 | 0.134 | 0.199 | 14.232 | 962 | 288 |
| SLF | -0.125 | 0.092 | -0.305 | 0.056 | 0.175 | 0.233 | 22.210 | 962 | 288 |
| SS | -0.221 | 0.075 | -0.369 | -0.073 | 0.003 | 0.030 | 0.000 | 962 | 288 |
| UNC | -0.029 | 0.075 | -0.177 | 0.118 | 0.698 | 0.698 | 0.002 | 962 | 288 |
| AverageFA | -0.272 | 0.099 | -0.465 | -0.079 | 0.006 | 0.030 | 30.149 | 962 | 288 |

Table S73. Cohen’s d effect sizes after meta-analysis, for AD differences between non-antidepressant users and healthy controls **in adults only**. Age, sex, agexsex, age^2^, age^2^xsex and scansite included as covariates.

| Region | Cohen’s d | SE | CI LB | CI UB | P-value | FDR P-value | I^2^ | Controls | Patients |
| --- | --- | --- | --- | --- | --- | --- | --- | --- | --- |
| ACR | -0.078 | 0.113 | -0.299 | 0.143 | 0.489 | 0.929 | 46.376 | 962 | 288 |
| ALIC | -0.016 | 0.082 | -0.176 | 0.144 | 0.849 | 0.929 | 8.105 | 962 | 288 |
| BCC | 0.107 | 0.097 | -0.083 | 0.296 | 0.271 | 0.929 | 28.412 | 962 | 288 |
| CC | 0.085 | 0.107 | -0.125 | 0.295 | 0.427 | 0.929 | 40.167 | 962 | 288 |
| CGC | -0.161 | 0.075 | -0.308 | -0.013 | 0.033 | 0.600 | 0.000 | 962 | 288 |
| CGH | -0.081 | 0.075 | -0.229 | 0.067 | 0.282 | 0.929 | 0.000 | 962 | 288 |
| CR | 0.019 | 0.075 | -0.128 | 0.167 | 0.799 | 0.929 | 0.000 | 962 | 288 |
| CST | 0.067 | 0.075 | -0.081 | 0.214 | 0.376 | 0.929 | 0.000 | 962 | 288 |
| EC | -0.075 | 0.075 | -0.222 | 0.073 | 0.321 | 0.929 | 0.000 | 962 | 288 |
| FX | 0.093 | 0.105 | -0.114 | 0.300 | 0.377 | 0.929 | 38.290 | 962 | 288 |
| FXST | -0.149 | 0.075 | -0.297 | -0.002 | 0.048 | 0.600 | 0.000 | 962 | 288 |
| GCC | 0.009 | 0.075 | -0.139 | 0.156 | 0.907 | 0.929 | 0.000 | 962 | 288 |
| IC | 0.008 | 0.095 | -0.177 | 0.194 | 0.929 | 0.929 | 25.746 | 962 | 288 |
| IFO | -0.052 | 0.076 | -0.200 | 0.096 | 0.488 | 0.929 | 0.000 | 962 | 288 |
| PCR | 0.038 | 0.084 | -0.126 | 0.202 | 0.647 | 0.929 | 11.018 | 962 | 288 |
| PLIC | 0.085 | 0.075 | -0.063 | 0.232 | 0.261 | 0.929 | 0.002 | 962 | 288 |
| PTR | 0.007 | 0.075 | -0.141 | 0.154 | 0.929 | 0.929 | 0.000 | 962 | 288 |
| RLIC | -0.032 | 0.128 | -0.282 | 0.218 | 0.804 | 0.929 | 57.791 | 962 | 288 |
| SCC | 0.052 | 0.104 | -0.151 | 0.255 | 0.616 | 0.929 | 36.720 | 962 | 288 |
| SCR | 0.107 | 0.075 | -0.040 | 0.255 | 0.154 | 0.929 | 0.000 | 962 | 288 |
| SFO | -0.036 | 0.075 | -0.184 | 0.112 | 0.632 | 0.929 | 0.000 | 962 | 288 |
| SLF | -0.043 | 0.076 | -0.193 | 0.106 | 0.569 | 0.929 | 1.146 | 962 | 288 |
| SS | -0.113 | 0.075 | -0.261 | 0.035 | 0.133 | 0.929 | 0.000 | 962 | 288 |
| UNC | 0.042 | 0.155 | -0.263 | 0.346 | 0.789 | 0.929 | 71.698 | 962 | 288 |
| AverageAD | 0.012 | 0.075 | -0.135 | 0.160 | 0.871 | 0.929 | 0.000 | 962 | 288 |

Table S74. Cohen’s d effect sizes after meta-analysis, for MD differences between non-antidepressant users and healthy controls **in adults only**. Age, sex, agexsex, age^2^, age^2^xsex and scansite included as covariates.

| Region | Cohen’s d | SE | CI LB | CI UB | P-value | FDR P-value | I^2^ | Controls | Patients |
| --- | --- | --- | --- | --- | --- | --- | --- | --- | --- |
| ACR | 0.032 | 0.101 | -0.165 | 0.229 | 0.749 | 0.749 | 33.263 | 962 | 288 |
| ALIC | 0.172 | 0.148 | -0.117 | 0.461 | 0.243 | 0.467 | 68.311 | 962 | 288 |
| BCC | 0.292 | 0.127 | 0.043 | 0.541 | 0.022 | 0.418 | 56.960 | 962 | 288 |
| CC | 0.247 | 0.129 | -0.005 | 0.499 | 0.055 | 0.418 | 58.250 | 962 | 288 |
| CGC | 0.058 | 0.075 | -0.090 | 0.206 | 0.441 | 0.525 | 0.003 | 962 | 288 |
| CGH | 0.112 | 0.082 | -0.049 | 0.274 | 0.173 | 0.418 | 9.224 | 962 | 288 |
| CR | 0.090 | 0.082 | -0.071 | 0.251 | 0.275 | 0.491 | 8.832 | 962 | 288 |
| CST | 0.114 | 0.075 | -0.033 | 0.262 | 0.129 | 0.418 | 0.005 | 962 | 288 |
| EC | 0.065 | 0.079 | -0.091 | 0.221 | 0.413 | 0.525 | 5.119 | 962 | 288 |
| FX | 0.100 | 0.075 | -0.048 | 0.248 | 0.184 | 0.418 | 0.003 | 962 | 288 |
| FXST | 0.132 | 0.085 | -0.035 | 0.298 | 0.121 | 0.418 | 12.244 | 962 | 288 |
| GCC | 0.129 | 0.110 | -0.086 | 0.345 | 0.238 | 0.467 | 43.110 | 962 | 288 |
| IC | 0.073 | 0.087 | -0.098 | 0.243 | 0.405 | 0.525 | 15.487 | 962 | 288 |
| IFO | 0.062 | 0.075 | -0.086 | 0.209 | 0.411 | 0.525 | 0.000 | 962 | 288 |
| PCR | 0.109 | 0.079 | -0.046 | 0.263 | 0.167 | 0.418 | 4.328 | 962 | 288 |
| PLIC | 0.069 | 0.075 | -0.078 | 0.217 | 0.357 | 0.525 | 0.000 | 962 | 288 |
| PTR | 0.051 | 0.091 | -0.126 | 0.228 | 0.574 | 0.624 | 20.215 | 962 | 288 |
| RLIC | 0.032 | 0.095 | -0.155 | 0.218 | 0.740 | 0.749 | 26.751 | 962 | 288 |
| SCC | 0.150 | 0.110 | -0.067 | 0.366 | 0.176 | 0.418 | 43.444 | 962 | 288 |
| SCR | 0.168 | 0.083 | 0.005 | 0.332 | 0.044 | 0.418 | 10.485 | 962 | 288 |
| SFO | 0.155 | 0.095 | -0.030 | 0.340 | 0.101 | 0.418 | 25.481 | 962 | 288 |
| SLF | 0.075 | 0.093 | -0.108 | 0.258 | 0.422 | 0.525 | 24.030 | 962 | 288 |
| SS | 0.071 | 0.075 | -0.076 | 0.219 | 0.342 | 0.525 | 0.000 | 962 | 288 |
| UNC | 0.083 | 0.142 | -0.196 | 0.362 | 0.558 | 0.624 | 66.146 | 962 | 288 |
| AverageMD | 0.143 | 0.089 | -0.031 | 0.317 | 0.106 | 0.418 | 17.911 | 962 | 288 |

Table S75. Cohen’s d effect sizes after meta-analysis, for RD differences between non-antidepressant users and healthy controls **in adults only**. Age, sex, agexsex, age^2^, age^2^xsex and scansite included as covariates.

| Region | Cohen’s d | SE | CI LB | CI UB | P-value | FDR P-value | I^2^ | Controls | Patients |
| --- | --- | --- | --- | --- | --- | --- | --- | --- | --- |
| ACR | 0.132 | 0.122 | -0.108 | 0.371 | 0.282 | 0.320 | 54.118 | 962 | 288 |
| ALIC | 0.261 | 0.163 | -0.059 | 0.581 | 0.110 | 0.172 | 74.192 | 962 | 288 |
| BCC | 0.280 | 0.132 | 0.021 | 0.539 | 0.034 | 0.121 | 60.344 | 962 | 288 |
| CC | 0.244 | 0.131 | -0.012 | 0.500 | 0.062 | 0.155 | 59.493 | 962 | 288 |
| CGC | 0.253 | 0.140 | -0.020 | 0.527 | 0.069 | 0.155 | 64.220 | 962 | 288 |
| CGH | 0.228 | 0.094 | 0.044 | 0.413 | 0.015 | 0.121 | 25.014 | 962 | 288 |
| CR | 0.188 | 0.106 | -0.020 | 0.395 | 0.076 | 0.155 | 39.029 | 962 | 288 |
| CST | 0.136 | 0.079 | -0.020 | 0.291 | 0.087 | 0.155 | 4.721 | 962 | 288 |
| EC | 0.138 | 0.077 | -0.013 | 0.290 | 0.073 | 0.155 | 2.202 | 962 | 288 |
| FX | 0.108 | 0.075 | -0.040 | 0.256 | 0.152 | 0.224 | 0.007 | 962 | 288 |
| FXST | 0.275 | 0.082 | 0.113 | 0.437 | 0.001 | 0.025 | 8.653 | 962 | 288 |
| GCC | 0.162 | 0.117 | -0.067 | 0.391 | 0.166 | 0.231 | 49.612 | 962 | 288 |
| IC | 0.175 | 0.100 | -0.022 | 0.372 | 0.082 | 0.155 | 32.373 | 962 | 288 |
| IFO | 0.123 | 0.075 | -0.025 | 0.271 | 0.102 | 0.170 | 0.000 | 962 | 288 |
| PCR | 0.178 | 0.086 | 0.009 | 0.347 | 0.039 | 0.122 | 14.190 | 962 | 288 |
| PLIC | 0.093 | 0.089 | -0.081 | 0.267 | 0.296 | 0.322 | 17.711 | 962 | 288 |
| PTR | 0.112 | 0.117 | -0.118 | 0.341 | 0.340 | 0.354 | 49.789 | 962 | 288 |
| RLIC | 0.093 | 0.076 | -0.057 | 0.242 | 0.224 | 0.274 | 1.150 | 962 | 288 |
| SCC | 0.121 | 0.101 | -0.077 | 0.319 | 0.230 | 0.274 | 32.725 | 962 | 288 |
| SCR | 0.202 | 0.095 | 0.017 | 0.388 | 0.032 | 0.121 | 25.238 | 962 | 288 |
| SFO | 0.204 | 0.095 | 0.017 | 0.390 | 0.032 | 0.121 | 26.031 | 962 | 288 |
| SLF | 0.135 | 0.109 | -0.079 | 0.349 | 0.217 | 0.274 | 42.580 | 962 | 288 |
| SS | 0.192 | 0.077 | 0.040 | 0.343 | 0.013 | 0.121 | 2.478 | 962 | 288 |
| UNC | 0.081 | 0.095 | -0.105 | 0.266 | 0.394 | 0.394 | 25.648 | 962 | 288 |
| AverageRD | 0.209 | 0.097 | 0.019 | 0.400 | 0.031 | 0.121 | 28.750 | 962 | 288 |

Table S76. Cohen’s d effect sizes after meta-analysis, for FA differences between non-antidepressant users and antidepressant users **in adults only**. Age, sex, agexsex, age^2^, age^2^xsex and scansite included as covariates.

| Region | Cohen’s d | SE | CI LB | CI UB | P-value | FDR P-value | I^2^ | Controls | Patients |
| --- | --- | --- | --- | --- | --- | --- | --- | --- | --- |
| ACR | 0.022 | 0.152 | -0.277 | 0.320 | 0.886 | 0.981 | 45.372 | 164 | 335 |
| ALIC | 0.058 | 0.162 | -0.259 | 0.376 | 0.719 | 0.981 | 51.348 | 164 | 335 |
| BCC | 0.033 | 0.212 | -0.382 | 0.448 | 0.876 | 0.981 | 71.209 | 164 | 335 |
| CC | -0.009 | 0.165 | -0.332 | 0.315 | 0.957 | 0.981 | 52.954 | 164 | 335 |
| CGC | 0.007 | 0.222 | -0.428 | 0.443 | 0.974 | 0.981 | 73.814 | 164 | 335 |
| CGH | 0.150 | 0.194 | -0.230 | 0.529 | 0.440 | 0.981 | 65.500 | 164 | 335 |
| CR | -0.033 | 0.173 | -0.373 | 0.306 | 0.847 | 0.981 | 57.111 | 164 | 335 |
| CST | -0.111 | 0.142 | -0.389 | 0.166 | 0.432 | 0.981 | 37.296 | 164 | 335 |
| EC | 0.004 | 0.156 | -0.302 | 0.309 | 0.981 | 0.981 | 47.564 | 164 | 335 |
| FX | 0.167 | 0.135 | -0.097 | 0.430 | 0.216 | 0.981 | 31.858 | 164 | 335 |
| FXST | 0.106 | 0.192 | -0.270 | 0.481 | 0.581 | 0.981 | 64.734 | 164 | 335 |
| GCC | -0.025 | 0.102 | -0.225 | 0.174 | 0.802 | 0.981 | 0.001 | 164 | 335 |
| IC | -0.116 | 0.233 | -0.572 | 0.340 | 0.619 | 0.981 | 76.028 | 164 | 335 |
| IFO | -0.028 | 0.102 | -0.228 | 0.171 | 0.780 | 0.981 | 0.000 | 164 | 335 |
| PCR | 0.026 | 0.181 | -0.330 | 0.382 | 0.886 | 0.981 | 60.834 | 164 | 335 |
| PLIC | -0.192 | 0.260 | -0.702 | 0.318 | 0.460 | 0.981 | 80.763 | 164 | 335 |
| PTR | -0.086 | 0.203 | -0.483 | 0.311 | 0.671 | 0.981 | 68.496 | 164 | 335 |
| RLIC | -0.120 | 0.188 | -0.489 | 0.249 | 0.524 | 0.981 | 63.404 | 164 | 335 |
| SCC | 0.009 | 0.102 | -0.190 | 0.208 | 0.931 | 0.981 | 0.000 | 164 | 335 |
| SCR | -0.131 | 0.119 | -0.364 | 0.103 | 0.273 | 0.981 | 17.378 | 164 | 335 |
| SFO | -0.102 | 0.119 | -0.334 | 0.130 | 0.389 | 0.981 | 16.961 | 164 | 335 |
| SLF | -0.037 | 0.194 | -0.418 | 0.344 | 0.848 | 0.981 | 65.728 | 164 | 335 |
| SS | 0.013 | 0.186 | -0.351 | 0.377 | 0.943 | 0.981 | 62.529 | 164 | 335 |
| UNC | 0.121 | 0.102 | -0.079 | 0.320 | 0.236 | 0.981 | 0.000 | 164 | 335 |
| AverageFA | 0.018 | 0.170 | -0.315 | 0.351 | 0.917 | 0.981 | 55.493 | 164 | 335 |

Table S77. Cohen’s d effect sizes after meta-analysis, for AD differences between non-antidepressant users and antidepressant users **in adults only**. Age, sex, agexsex, age^2^, age^2^xsex and scansite included as covariates.

| Region | Cohen’s d | SE | CI LB | CI UB | P-value | FDR P-value | I^2^ | Controls | Patients |
| --- | --- | --- | --- | --- | --- | --- | --- | --- | --- |
| ACR | -0.111 | 0.102 | -0.310 | 0.089 | 0.277 | 0.834 | 0.000 | 164 | 335 |
| ALIC | -0.057 | 0.138 | -0.327 | 0.213 | 0.678 | 0.848 | 34.563 | 164 | 335 |
| BCC | -0.130 | 0.210 | -0.543 | 0.282 | 0.537 | 0.834 | 70.896 | 164 | 335 |
| CC | -0.159 | 0.174 | -0.500 | 0.182 | 0.361 | 0.834 | 57.516 | 164 | 335 |
| CGC | -0.010 | 0.102 | -0.210 | 0.189 | 0.918 | 0.981 | 0.000 | 164 | 335 |
| CGH | 0.062 | 0.131 | -0.194 | 0.319 | 0.634 | 0.834 | 28.769 | 164 | 335 |
| CR | -0.098 | 0.102 | -0.297 | 0.101 | 0.336 | 0.834 | 0.000 | 164 | 335 |
| CST | -0.085 | 0.102 | -0.285 | 0.114 | 0.402 | 0.834 | 0.002 | 164 | 335 |
| EC | 0.091 | 0.123 | -0.150 | 0.331 | 0.459 | 0.834 | 21.191 | 164 | 335 |
| FX | -0.249 | 0.133 | -0.509 | 0.011 | 0.061 | 0.658 | 29.920 | 164 | 335 |
| FXST | 0.181 | 0.102 | -0.019 | 0.380 | 0.076 | 0.658 | 0.000 | 164 | 335 |
| GCC | -0.179 | 0.102 | -0.379 | 0.021 | 0.079 | 0.658 | 0.000 | 164 | 335 |
| IC | 0.002 | 0.102 | -0.197 | 0.202 | 0.981 | 0.981 | 0.000 | 164 | 335 |
| IFO | 0.095 | 0.102 | -0.105 | 0.294 | 0.352 | 0.834 | 0.000 | 164 | 335 |
| PCR | -0.137 | 0.145 | -0.421 | 0.147 | 0.344 | 0.834 | 39.891 | 164 | 335 |
| PLIC | -0.076 | 0.102 | -0.276 | 0.123 | 0.452 | 0.834 | 0.006 | 164 | 335 |
| PTR | 0.054 | 0.102 | -0.146 | 0.253 | 0.597 | 0.834 | 0.000 | 164 | 335 |
| RLIC | 0.087 | 0.138 | -0.182 | 0.357 | 0.525 | 0.834 | 34.358 | 164 | 335 |
| SCC | -0.099 | 0.175 | -0.443 | 0.245 | 0.573 | 0.834 | 58.158 | 164 | 335 |
| SCR | -0.060 | 0.104 | -0.264 | 0.144 | 0.566 | 0.834 | 2.672 | 164 | 335 |
| SFO | 0.023 | 0.102 | -0.177 | 0.222 | 0.824 | 0.936 | 0.000 | 164 | 335 |
| SLF | 0.004 | 0.102 | -0.196 | 0.203 | 0.972 | 0.981 | 0.000 | 164 | 335 |
| SS | 0.067 | 0.135 | -0.198 | 0.332 | 0.619 | 0.834 | 32.416 | 164 | 335 |
| UNC | -0.119 | 0.213 | -0.537 | 0.298 | 0.575 | 0.834 | 71.475 | 164 | 335 |
| AverageAD | -0.026 | 0.102 | -0.225 | 0.173 | 0.800 | 0.936 | 0.000 | 164 | 335 |

Table S78. Cohen’s d effect sizes after meta-analysis, for MD differences between non-antidepressant users and antidepressant users **in adults only**. Age, sex, agexsex, age^2^, age^2^xsex and scansite included as covariates.

| Region | Cohen’s d | SE | CI LB | CI UB | P-value | FDR P-value | I^2^ | Controls | Patients |
| --- | --- | --- | --- | --- | --- | --- | --- | --- | --- |
| ACR | -0.027 | 0.105 | -0.232 | 0.179 | 0.799 | 0.928 | 3.304 | 164 | 335 |
| ALIC | -0.121 | 0.175 | -0.465 | 0.222 | 0.488 | 0.928 | 58.142 | 164 | 335 |
| BCC | -0.287 | 0.193 | -0.665 | 0.091 | 0.137 | 0.685 | 65.180 | 164 | 335 |
| CC | -0.244 | 0.153 | -0.544 | 0.055 | 0.110 | 0.685 | 45.392 | 164 | 335 |
| CGC | -0.084 | 0.102 | -0.283 | 0.116 | 0.410 | 0.928 | 0.002 | 164 | 335 |
| CGH | -0.060 | 0.175 | -0.402 | 0.282 | 0.732 | 0.928 | 57.701 | 164 | 335 |
| CR | -0.029 | 0.123 | -0.270 | 0.211 | 0.812 | 0.928 | 21.282 | 164 | 335 |
| CST | -0.045 | 0.165 | -0.369 | 0.279 | 0.787 | 0.928 | 53.006 | 164 | 335 |
| EC | 0.056 | 0.143 | -0.224 | 0.336 | 0.696 | 0.928 | 38.784 | 164 | 335 |
| FX | -0.252 | 0.124 | -0.496 | -0.009 | 0.042 | 0.588 | 22.294 | 164 | 335 |
| FXST | 0.020 | 0.107 | -0.191 | 0.230 | 0.854 | 0.928 | 5.817 | 164 | 335 |
| GCC | -0.195 | 0.118 | -0.427 | 0.036 | 0.098 | 0.685 | 16.319 | 164 | 335 |
| IC | 0.051 | 0.102 | -0.148 | 0.251 | 0.613 | 0.928 | 0.000 | 164 | 335 |
| IFO | 0.037 | 0.141 | -0.239 | 0.314 | 0.791 | 0.928 | 37.057 | 164 | 335 |
| PCR | -0.031 | 0.126 | -0.277 | 0.215 | 0.803 | 0.928 | 24.017 | 164 | 335 |
| PLIC | 0.040 | 0.102 | -0.159 | 0.239 | 0.695 | 0.928 | 0.000 | 164 | 335 |
| PTR | 0.202 | 0.102 | 0.003 | 0.402 | 0.047 | 0.588 | 0.001 | 164 | 335 |
| RLIC | 0.095 | 0.102 | -0.104 | 0.295 | 0.349 | 0.928 | 0.000 | 164 | 335 |
| SCC | -0.106 | 0.102 | -0.306 | 0.093 | 0.296 | 0.928 | 0.000 | 164 | 335 |
| SCR | -0.015 | 0.134 | -0.278 | 0.248 | 0.912 | 0.950 | 31.911 | 164 | 335 |
| SFO | -0.027 | 0.135 | -0.291 | 0.238 | 0.844 | 0.928 | 32.215 | 164 | 335 |
| SLF | 0.100 | 0.102 | -0.099 | 0.300 | 0.324 | 0.928 | 0.000 | 164 | 335 |
| SS | 0.058 | 0.102 | -0.141 | 0.257 | 0.568 | 0.928 | 0.000 | 164 | 335 |
| UNC | -0.127 | 0.218 | -0.555 | 0.301 | 0.561 | 0.928 | 72.880 | 164 | 335 |
| AverageMD | -0.005 | 0.105 | -0.211 | 0.202 | 0.963 | 0.963 | 3.688 | 164 | 335 |

Table S79. Cohen’s d effect sizes after meta-analysis, for RD differences between non-antidepressant users and antidepressant users **in adults only**. Age, sex, agexsex, age^2^, age^2^xsex and scansite included as covariates.

| Region | Cohen’s d | SE | CI LB | CI UB | P-value | FDR P-value | I^2^ | Controls | Patients |
| --- | --- | --- | --- | --- | --- | --- | --- | --- | --- |
| ACR | -0.055 | 0.147 | -0.344 | 0.234 | 0.711 | 0.982 | 41.984 | 164 | 335 |
| ALIC | -0.069 | 0.184 | -0.430 | 0.292 | 0.708 | 0.982 | 62.157 | 164 | 335 |
| BCC | -0.229 | 0.211 | -0.642 | 0.185 | 0.278 | 0.982 | 70.949 | 164 | 335 |
| CC | -0.173 | 0.162 | -0.490 | 0.144 | 0.285 | 0.982 | 51.180 | 164 | 335 |
| CGC | -0.095 | 0.135 | -0.360 | 0.170 | 0.481 | 0.982 | 32.277 | 164 | 335 |
| CGH | -0.102 | 0.223 | -0.539 | 0.335 | 0.647 | 0.982 | 73.941 | 164 | 335 |
| CR | -0.032 | 0.150 | -0.327 | 0.263 | 0.831 | 0.982 | 44.051 | 164 | 335 |
| CST | -0.009 | 0.151 | -0.305 | 0.286 | 0.950 | 0.982 | 44.042 | 164 | 335 |
| EC | 0.004 | 0.164 | -0.317 | 0.325 | 0.982 | 0.982 | 52.254 | 164 | 335 |
| FX | -0.232 | 0.114 | -0.457 | -0.008 | 0.043 | 0.982 | 12.811 | 164 | 335 |
| FXST | -0.133 | 0.159 | -0.445 | 0.179 | 0.403 | 0.982 | 49.499 | 164 | 335 |
| GCC | -0.135 | 0.121 | -0.372 | 0.103 | 0.266 | 0.982 | 19.555 | 164 | 335 |
| IC | 0.030 | 0.166 | -0.296 | 0.356 | 0.855 | 0.982 | 53.562 | 164 | 335 |
| IFO | 0.057 | 0.102 | -0.143 | 0.256 | 0.576 | 0.982 | 0.009 | 164 | 335 |
| PCR | -0.043 | 0.134 | -0.305 | 0.220 | 0.751 | 0.982 | 31.326 | 164 | 335 |
| PLIC | 0.110 | 0.206 | -0.294 | 0.515 | 0.593 | 0.982 | 69.596 | 164 | 335 |
| PTR | 0.121 | 0.154 | -0.181 | 0.423 | 0.432 | 0.982 | 46.355 | 164 | 335 |
| RLIC | 0.015 | 0.131 | -0.242 | 0.273 | 0.909 | 0.982 | 28.987 | 164 | 335 |
| SCC | -0.051 | 0.102 | -0.250 | 0.148 | 0.614 | 0.982 | 0.000 | 164 | 335 |
| SCR | 0.022 | 0.143 | -0.258 | 0.301 | 0.879 | 0.982 | 38.394 | 164 | 335 |
| SFO | -0.009 | 0.154 | -0.310 | 0.292 | 0.953 | 0.982 | 46.181 | 164 | 335 |
| SLF | 0.107 | 0.121 | -0.131 | 0.345 | 0.377 | 0.982 | 19.640 | 164 | 335 |
| SS | -0.013 | 0.130 | -0.267 | 0.241 | 0.920 | 0.982 | 27.349 | 164 | 335 |
| UNC | -0.109 | 0.166 | -0.435 | 0.216 | 0.511 | 0.982 | 53.452 | 164 | 335 |
| AverageRD | -0.033 | 0.135 | -0.299 | 0.232 | 0.806 | 0.982 | 32.530 | 164 | 335 |

Table S80. Cohen’s d effect sizes after meta-analysis, for FA differences between antidepressant users and healthy controls **in adolescents only**. Age, sex, agexsex, age^2^, age^2^xsex and scansite included as covariates.

| Region | Cohen’s d | SE | CI LB | CI UB | P-value | FDR P-value | I^2^ | Controls | Patients |
| --- | --- | --- | --- | --- | --- | --- | --- | --- | --- |
| ACR | 0.097 | 0.233 | -0.360 | 0.554 | 0.676 | 0.987 | 63.195 | 138 | 128 |
| ALIC | -0.005 | 0.134 | -0.269 | 0.258 | 0.968 | 0.987 | 0.000 | 138 | 128 |
| BCC | -0.225 | 0.135 | -0.489 | 0.039 | 0.095 | 0.987 | 0.000 | 138 | 128 |
| CC | -0.121 | 0.135 | -0.385 | 0.143 | 0.368 | 0.987 | 0.000 | 138 | 128 |
| CGC | 0.278 | 0.307 | -0.324 | 0.880 | 0.366 | 0.987 | 78.376 | 138 | 128 |
| CGH | 0.194 | 0.151 | -0.102 | 0.491 | 0.199 | 0.987 | 16.792 | 138 | 128 |
| CR | 0.103 | 0.189 | -0.267 | 0.473 | 0.586 | 0.987 | 44.752 | 138 | 128 |
| CST | 0.072 | 0.185 | -0.290 | 0.435 | 0.697 | 0.987 | 42.665 | 138 | 128 |
| EC | 0.329 | 0.158 | 0.019 | 0.638 | 0.038 | 0.950 | 22.469 | 138 | 128 |
| FX | -0.046 | 0.248 | -0.531 | 0.440 | 0.854 | 0.987 | 66.922 | 138 | 128 |
| FXST | 0.002 | 0.135 | -0.262 | 0.266 | 0.987 | 0.987 | 0.000 | 138 | 128 |
| GCC | -0.014 | 0.135 | -0.279 | 0.250 | 0.915 | 0.987 | 0.000 | 138 | 128 |
| IC | 0.122 | 0.179 | -0.229 | 0.474 | 0.496 | 0.987 | 38.958 | 138 | 128 |
| IFO | 0.047 | 0.213 | -0.370 | 0.465 | 0.824 | 0.987 | 56.069 | 138 | 128 |
| PCR | 0.124 | 0.135 | -0.140 | 0.388 | 0.357 | 0.987 | 0.000 | 138 | 128 |
| PLIC | 0.130 | 0.196 | -0.254 | 0.513 | 0.508 | 0.987 | 48.138 | 138 | 128 |
| PTR | 0.080 | 0.148 | -0.211 | 0.371 | 0.592 | 0.987 | 14.332 | 138 | 128 |
| RLIC | 0.211 | 0.172 | -0.126 | 0.548 | 0.219 | 0.987 | 33.677 | 138 | 128 |
| SCC | 0.069 | 0.145 | -0.214 | 0.353 | 0.633 | 0.987 | 10.817 | 138 | 128 |
| SCR | 0.029 | 0.162 | -0.289 | 0.348 | 0.856 | 0.987 | 26.920 | 138 | 128 |
| SFO | 0.004 | 0.135 | -0.260 | 0.268 | 0.977 | 0.987 | 0.000 | 138 | 128 |
| SLF | 0.177 | 0.135 | -0.088 | 0.441 | 0.191 | 0.987 | 0.000 | 138 | 128 |
| SS | -0.011 | 0.135 | -0.275 | 0.253 | 0.937 | 0.987 | 0.000 | 138 | 128 |
| UNC | 0.110 | 0.140 | -0.164 | 0.384 | 0.431 | 0.987 | 5.600 | 138 | 128 |
| AverageFA | 0.011 | 0.135 | -0.253 | 0.275 | 0.937 | 0.987 | 0.000 | 138 | 128 |

Table S81. Cohen’s d effect sizes after meta-analysis, for AD differences between antidepressant users and healthy controls **in adolescents only**. Age, sex, agexsex, age^2^, age^2^xsex and scansite included as covariates.

| Region | Cohen’s d | SE | CI LB | CI UB | P-value | FDR P-value | I^2^ | Controls | Patients |
| --- | --- | --- | --- | --- | --- | --- | --- | --- | --- |
| ACR | 0.076 | 0.135 | -0.188 | 0.341 | 0.573 | 0.986 | 0.006 | 138 | 128 |
| ALIC | 0.135 | 0.135 | -0.130 | 0.399 | 0.319 | 0.986 | 0.008 | 138 | 128 |
| BCC | 0.018 | 0.135 | -0.246 | 0.282 | 0.893 | 0.986 | 0.000 | 138 | 128 |
| CC | 0.048 | 0.147 | -0.240 | 0.336 | 0.745 | 0.986 | 12.879 | 138 | 128 |
| CGC | 0.316 | 0.307 | -0.287 | 0.919 | 0.304 | 0.986 | 78.394 | 138 | 128 |
| CGH | 0.087 | 0.172 | -0.251 | 0.425 | 0.613 | 0.986 | 34.418 | 138 | 128 |
| CR | 0.099 | 0.187 | -0.267 | 0.466 | 0.595 | 0.986 | 43.577 | 138 | 128 |
| CST | 0.023 | 0.195 | -0.360 | 0.405 | 0.907 | 0.986 | 47.980 | 138 | 128 |
| EC | 0.251 | 0.284 | -0.305 | 0.807 | 0.377 | 0.986 | 74.803 | 138 | 128 |
| FX | 0.029 | 0.135 | -0.235 | 0.294 | 0.828 | 0.986 | 0.001 | 138 | 128 |
| FXST | 0.329 | 0.136 | 0.063 | 0.594 | 0.015 | 0.375 | 0.002 | 138 | 128 |
| GCC | 0.114 | 0.135 | -0.149 | 0.378 | 0.395 | 0.986 | 0.000 | 138 | 128 |
| IC | 0.084 | 0.296 | -0.495 | 0.663 | 0.777 | 0.986 | 76.772 | 138 | 128 |
| IFO | 0.268 | 0.209 | -0.141 | 0.677 | 0.199 | 0.986 | 54.081 | 138 | 128 |
| PCR | 0.140 | 0.238 | -0.326 | 0.605 | 0.557 | 0.986 | 64.687 | 138 | 128 |
| PLIC | -0.032 | 0.295 | -0.609 | 0.546 | 0.914 | 0.986 | 76.648 | 138 | 128 |
| PTR | 0.025 | 0.198 | -0.363 | 0.414 | 0.898 | 0.986 | 49.845 | 138 | 128 |
| RLIC | 0.219 | 0.226 | -0.224 | 0.662 | 0.334 | 0.986 | 60.795 | 138 | 128 |
| SCC | 0.006 | 0.209 | -0.403 | 0.415 | 0.978 | 0.986 | 54.459 | 138 | 128 |
| SCR | 0.004 | 0.245 | -0.476 | 0.485 | 0.986 | 0.986 | 66.664 | 138 | 128 |
| SFO | 0.229 | 0.256 | -0.272 | 0.730 | 0.371 | 0.986 | 69.153 | 138 | 128 |
| SLF | 0.171 | 0.264 | -0.346 | 0.688 | 0.516 | 0.986 | 71.079 | 138 | 128 |
| SS | 0.155 | 0.135 | -0.109 | 0.420 | 0.249 | 0.986 | 0.000 | 138 | 128 |
| UNC | 0.007 | 0.206 | -0.397 | 0.411 | 0.973 | 0.986 | 53.320 | 138 | 128 |
| AverageAD | 0.083 | 0.236 | -0.380 | 0.546 | 0.725 | 0.986 | 64.086 | 138 | 128 |

Table S82. Cohen’s d effect sizes after meta-analysis, for MD differences between antidepressant users and healthy controls **in adolescents only**. Age, sex, agexsex, age^2^, age^2^xsex and scansite included as covariates.

| Region | Cohen’s d | SE | CI LB | CI UB | P-value | FDR P-value | I^2^ | Controls | Patients |
| --- | --- | --- | --- | --- | --- | --- | --- | --- | --- |
| ACR | 0.014 | 0.135 | -0.250 | 0.277 | 0.919 | 0.919 | 0.000 | 138 | 128 |
| ALIC | 0.125 | 0.192 | -0.252 | 0.502 | 0.517 | 0.841 | 46.192 | 138 | 128 |
| BCC | 0.174 | 0.135 | -0.090 | 0.438 | 0.196 | 0.817 | 0.000 | 138 | 128 |
| CC | 0.148 | 0.135 | -0.116 | 0.411 | 0.273 | 0.841 | 0.000 | 138 | 128 |
| CGC | 0.313 | 0.135 | 0.048 | 0.578 | 0.021 | 0.388 | 0.000 | 138 | 128 |
| CGH | -0.043 | 0.135 | -0.307 | 0.221 | 0.751 | 0.894 | 0.000 | 138 | 128 |
| CR | 0.070 | 0.135 | -0.194 | 0.333 | 0.605 | 0.841 | 0.000 | 138 | 128 |
| CST | 0.138 | 0.158 | -0.171 | 0.448 | 0.381 | 0.841 | 23.175 | 138 | 128 |
| EC | 0.047 | 0.214 | -0.373 | 0.467 | 0.826 | 0.903 | 56.401 | 138 | 128 |
| FX | 0.132 | 0.135 | -0.133 | 0.396 | 0.329 | 0.841 | 0.002 | 138 | 128 |
| FXST | 0.256 | 0.189 | -0.115 | 0.627 | 0.177 | 0.817 | 44.719 | 138 | 128 |
| GCC | 0.123 | 0.135 | -0.141 | 0.386 | 0.362 | 0.841 | 0.000 | 138 | 128 |
| IC | 0.111 | 0.155 | -0.193 | 0.416 | 0.473 | 0.841 | 20.439 | 138 | 128 |
| IFO | 0.291 | 0.135 | 0.026 | 0.556 | 0.031 | 0.388 | 0.000 | 138 | 128 |
| PCR | 0.098 | 0.135 | -0.166 | 0.362 | 0.467 | 0.841 | 0.000 | 138 | 128 |
| PLIC | 0.025 | 0.135 | -0.240 | 0.290 | 0.854 | 0.903 | 0.000 | 138 | 128 |
| PTR | 0.065 | 0.169 | -0.266 | 0.397 | 0.699 | 0.874 | 32.354 | 138 | 128 |
| RLIC | 0.142 | 0.135 | -0.123 | 0.406 | 0.293 | 0.841 | 0.000 | 138 | 128 |
| SCC | 0.086 | 0.144 | -0.197 | 0.368 | 0.553 | 0.841 | 10.361 | 138 | 128 |
| SCR | 0.066 | 0.135 | -0.199 | 0.330 | 0.626 | 0.841 | 0.000 | 138 | 128 |
| SFO | 0.262 | 0.181 | -0.094 | 0.617 | 0.149 | 0.817 | 39.835 | 138 | 128 |
| SLF | 0.092 | 0.196 | -0.293 | 0.477 | 0.639 | 0.841 | 48.320 | 138 | 128 |
| SS | 0.193 | 0.135 | -0.071 | 0.456 | 0.153 | 0.817 | 0.000 | 138 | 128 |
| UNC | 0.023 | 0.137 | -0.246 | 0.292 | 0.867 | 0.903 | 2.698 | 138 | 128 |
| AverageMD | 0.117 | 0.176 | -0.228 | 0.463 | 0.506 | 0.841 | 37.056 | 138 | 128 |

Table S83. Cohen’s d effect sizes after meta-analysis, for RD differences between antidepressant users and healthy controls **in adolescents only**. Age, sex, agexsex, age^2^, age^2^xsex and scansite included as covariates.

| Region | Cohen’s d | SE | CI LB | CI UB | P-value | FDR P-value | I^2^ | Controls | Patients |
| --- | --- | --- | --- | --- | --- | --- | --- | --- | --- |
| ACR | -0.024 | 0.157 | -0.333 | 0.284 | 0.877 | 0.994 | 22.714 | 138 | 128 |
| ALIC | 0.174 | 0.143 | -0.107 | 0.455 | 0.225 | 0.994 | 8.608 | 138 | 128 |
| BCC | 0.216 | 0.135 | -0.048 | 0.480 | 0.108 | 0.994 | 0.000 | 138 | 128 |
| CC | 0.153 | 0.135 | -0.111 | 0.417 | 0.255 | 0.994 | 0.000 | 138 | 128 |
| CGC | -0.018 | 0.253 | -0.513 | 0.478 | 0.945 | 0.994 | 68.696 | 138 | 128 |
| CGH | -0.100 | 0.135 | -0.364 | 0.165 | 0.460 | 0.994 | 0.002 | 138 | 128 |
| CR | -0.004 | 0.150 | -0.297 | 0.290 | 0.981 | 0.994 | 15.838 | 138 | 128 |
| CST | 0.183 | 0.151 | -0.113 | 0.479 | 0.225 | 0.994 | 16.558 | 138 | 128 |
| EC | -0.058 | 0.184 | -0.419 | 0.302 | 0.751 | 0.994 | 41.954 | 138 | 128 |
| FX | 0.123 | 0.158 | -0.187 | 0.434 | 0.437 | 0.994 | 23.351 | 138 | 128 |
| FXST | 0.151 | 0.175 | -0.193 | 0.495 | 0.389 | 0.994 | 36.602 | 138 | 128 |
| GCC | 0.104 | 0.135 | -0.160 | 0.368 | 0.440 | 0.994 | 0.000 | 138 | 128 |
| IC | 0.040 | 0.218 | -0.387 | 0.468 | 0.854 | 0.994 | 57.995 | 138 | 128 |
| IFO | 0.124 | 0.179 | -0.227 | 0.475 | 0.489 | 0.994 | 38.819 | 138 | 128 |
| PCR | -0.001 | 0.135 | -0.265 | 0.263 | 0.994 | 0.994 | 0.000 | 138 | 128 |
| PLIC | -0.014 | 0.180 | -0.367 | 0.338 | 0.936 | 0.994 | 39.157 | 138 | 128 |
| PTR | 0.017 | 0.190 | -0.355 | 0.388 | 0.929 | 0.994 | 45.372 | 138 | 128 |
| RLIC | -0.046 | 0.188 | -0.414 | 0.321 | 0.805 | 0.994 | 44.167 | 138 | 128 |
| SCC | -0.041 | 0.183 | -0.399 | 0.317 | 0.823 | 0.994 | 41.443 | 138 | 128 |
| SCR | 0.018 | 0.135 | -0.246 | 0.283 | 0.892 | 0.994 | 0.000 | 138 | 128 |
| SFO | 0.176 | 0.135 | -0.089 | 0.441 | 0.192 | 0.994 | 0.005 | 138 | 128 |
| SLF | -0.054 | 0.180 | -0.408 | 0.300 | 0.764 | 0.994 | 39.399 | 138 | 128 |
| SS | 0.148 | 0.135 | -0.116 | 0.412 | 0.271 | 0.994 | 0.000 | 138 | 128 |
| UNC | -0.026 | 0.137 | -0.295 | 0.244 | 0.853 | 0.994 | 3.158 | 138 | 128 |
| AverageRD | 0.102 | 0.171 | -0.234 | 0.438 | 0.551 | 0.994 | 33.919 | 138 | 128 |

Table S84. Cohen’s d effect sizes after meta-analysis, for FA differences between non-antidepressant users and healthy controls **in adolescents only**. Age, sex, agexsex, age^2^, age^2^xsex and scansite included as covariates.

| Region | Cohen’s d | SE | CI LB | CI UB | P-value | FDR P-value | I^2^ | Controls | Patients |
| --- | --- | --- | --- | --- | --- | --- | --- | --- | --- |
| ACR | -0.165 | 0.195 | -0.547 | 0.217 | 0.397 | 0.709 | 63.908 | 156 | 188 |
| ALIC | -0.006 | 0.244 | -0.484 | 0.472 | 0.981 | 0.981 | 76.875 | 156 | 188 |
| BCC | -0.322 | 0.112 | -0.541 | -0.103 | 0.004 | 0.063 | 0.000 | 156 | 188 |
| CC | -0.313 | 0.112 | -0.532 | -0.094 | 0.005 | 0.063 | 0.000 | 156 | 188 |
| CGC | 0.065 | 0.183 | -0.293 | 0.423 | 0.720 | 0.857 | 59.215 | 156 | 188 |
| CGH | 0.093 | 0.201 | -0.301 | 0.486 | 0.644 | 0.857 | 66.027 | 156 | 188 |
| CR | -0.187 | 0.159 | -0.500 | 0.125 | 0.241 | 0.689 | 46.862 | 156 | 188 |
| CST | -0.183 | 0.111 | -0.401 | 0.036 | 0.101 | 0.631 | 0.000 | 156 | 188 |
| EC | 0.276 | 0.239 | -0.193 | 0.745 | 0.248 | 0.689 | 75.770 | 156 | 188 |
| FX | 0.027 | 0.111 | -0.191 | 0.245 | 0.807 | 0.877 | 0.000 | 156 | 188 |
| FXST | 0.081 | 0.192 | -0.296 | 0.457 | 0.675 | 0.857 | 62.837 | 156 | 188 |
| GCC | -0.331 | 0.160 | -0.643 | -0.018 | 0.038 | 0.317 | 46.328 | 156 | 188 |
| IC | 0.090 | 0.143 | -0.190 | 0.369 | 0.529 | 0.827 | 34.531 | 156 | 188 |
| IFO | -0.153 | 0.199 | -0.543 | 0.237 | 0.441 | 0.735 | 65.384 | 156 | 188 |
| PCR | -0.161 | 0.111 | -0.379 | 0.057 | 0.147 | 0.639 | 0.000 | 156 | 188 |
| PLIC | 0.157 | 0.111 | -0.061 | 0.375 | 0.158 | 0.639 | 0.000 | 156 | 188 |
| PTR | 0.017 | 0.111 | -0.201 | 0.235 | 0.877 | 0.914 | 0.002 | 156 | 188 |
| RLIC | 0.032 | 0.116 | -0.196 | 0.259 | 0.785 | 0.877 | 6.518 | 156 | 188 |
| SCC | -0.101 | 0.111 | -0.318 | 0.117 | 0.366 | 0.704 | 0.000 | 156 | 188 |
| SCR | -0.140 | 0.148 | -0.430 | 0.151 | 0.345 | 0.704 | 39.172 | 156 | 188 |
| SFO | -0.181 | 0.134 | -0.444 | 0.083 | 0.179 | 0.639 | 27.071 | 156 | 188 |
| SLF | -0.114 | 0.125 | -0.359 | 0.132 | 0.363 | 0.704 | 17.795 | 156 | 188 |
| SS | 0.178 | 0.195 | -0.204 | 0.559 | 0.362 | 0.704 | 63.864 | 156 | 188 |
| UNC | 0.062 | 0.154 | -0.240 | 0.364 | 0.688 | 0.857 | 42.941 | 156 | 188 |
| AverageFA | -0.100 | 0.188 | -0.469 | 0.269 | 0.595 | 0.857 | 61.434 | 156 | 188 |

Table S85. Cohen’s d effect sizes after meta-analysis, for AD differences between non-antidepressant users and healthy controls **in adolescents only**. Age, sex, agexsex, age^2^, age^2^xsex and scansite included as covariates.

| Region | Cohen’s d | SE | CI LB | CI UB | P-value | FDR P-value | I^2^ | Controls | Patients |
| --- | --- | --- | --- | --- | --- | --- | --- | --- | --- |
| ACR | 0.110 | 0.111 | -0.108 | 0.328 | 0.321 | 0.535 | 0.000 | 156 | 188 |
| ALIC | 0.161 | 0.112 | -0.058 | 0.380 | 0.149 | 0.373 | 0.267 | 156 | 188 |
| BCC | -0.113 | 0.165 | -0.436 | 0.211 | 0.495 | 0.619 | 50.172 | 156 | 188 |
| CC | -0.023 | 0.111 | -0.241 | 0.195 | 0.834 | 0.862 | 0.007 | 156 | 188 |
| CGC | 0.298 | 0.129 | 0.046 | 0.551 | 0.021 | 0.200 | 20.726 | 156 | 188 |
| CGH | -0.038 | 0.139 | -0.310 | 0.234 | 0.786 | 0.854 | 31.455 | 156 | 188 |
| CR | 0.142 | 0.111 | -0.076 | 0.360 | 0.203 | 0.423 | 0.000 | 156 | 188 |
| CST | -0.184 | 0.179 | -0.534 | 0.166 | 0.303 | 0.535 | 57.280 | 156 | 188 |
| EC | 0.252 | 0.111 | 0.034 | 0.471 | 0.024 | 0.200 | 0.000 | 156 | 188 |
| FX | 0.175 | 0.201 | -0.219 | 0.569 | 0.384 | 0.565 | 65.816 | 156 | 188 |
| FXST | 0.136 | 0.111 | -0.082 | 0.354 | 0.221 | 0.425 | 0.000 | 156 | 188 |
| GCC | 0.080 | 0.111 | -0.138 | 0.298 | 0.471 | 0.619 | 0.001 | 156 | 188 |
| IC | 0.169 | 0.111 | -0.049 | 0.388 | 0.129 | 0.372 | 0.008 | 156 | 188 |
| IFO | 0.085 | 0.111 | -0.133 | 0.303 | 0.444 | 0.617 | 0.000 | 156 | 188 |
| PCR | 0.186 | 0.111 | -0.032 | 0.405 | 0.095 | 0.372 | 0.000 | 156 | 188 |
| PLIC | 0.034 | 0.111 | -0.184 | 0.251 | 0.763 | 0.854 | 0.000 | 156 | 188 |
| PTR | 0.294 | 0.112 | 0.074 | 0.514 | 0.009 | 0.200 | 0.007 | 156 | 188 |
| RLIC | 0.178 | 0.117 | -0.051 | 0.406 | 0.128 | 0.372 | 7.121 | 156 | 188 |
| SCC | -0.019 | 0.111 | -0.237 | 0.198 | 0.862 | 0.862 | 0.000 | 156 | 188 |
| SCR | 0.057 | 0.111 | -0.161 | 0.274 | 0.610 | 0.726 | 0.000 | 156 | 188 |
| SFO | 0.100 | 0.111 | -0.118 | 0.318 | 0.369 | 0.565 | 0.000 | 156 | 188 |
| SLF | 0.228 | 0.111 | 0.010 | 0.446 | 0.041 | 0.205 | 0.000 | 156 | 188 |
| SS | 0.166 | 0.111 | -0.051 | 0.384 | 0.134 | 0.372 | 0.000 | 156 | 188 |
| UNC | 0.229 | 0.111 | 0.010 | 0.447 | 0.040 | 0.205 | 0.000 | 156 | 188 |
| AverageAD | 0.155 | 0.112 | -0.064 | 0.373 | 0.165 | 0.375 | 0.002 | 156 | 188 |

Table S86. Cohen’s d effect sizes after meta-analysis, for MD differences between non-antidepressant users and healthy controls **in adolescents only**. Age, sex, agexsex, age^2^, age^2^xsex and scansite included as covariates.

| Region | Cohen’s d | SE | CI LB | CI UB | P-value | FDR P-value | I^2^ | Controls | Patients |
| --- | --- | --- | --- | --- | --- | --- | --- | --- | --- |
| ACR | 0.222 | 0.125 | -0.022 | 0.466 | 0.075 | 0.186 | 16.730 | 156 | 188 |
| ALIC | 0.212 | 0.111 | -0.007 | 0.430 | 0.057 | 0.164 | 0.000 | 156 | 188 |
| BCC | 0.215 | 0.114 | -0.008 | 0.438 | 0.059 | 0.164 | 3.159 | 156 | 188 |
| CC | 0.232 | 0.114 | 0.009 | 0.455 | 0.042 | 0.161 | 3.372 | 156 | 188 |
| CGC | 0.205 | 0.134 | -0.058 | 0.468 | 0.127 | 0.212 | 26.844 | 156 | 188 |
| CGH | -0.093 | 0.228 | -0.540 | 0.354 | 0.684 | 0.684 | 73.660 | 156 | 188 |
| CR | 0.265 | 0.112 | 0.044 | 0.485 | 0.019 | 0.119 | 1.018 | 156 | 188 |
| CST | -0.074 | 0.172 | -0.412 | 0.264 | 0.667 | 0.684 | 54.309 | 156 | 188 |
| EC | 0.172 | 0.133 | -0.088 | 0.433 | 0.195 | 0.257 | 25.718 | 156 | 188 |
| FX | 0.209 | 0.157 | -0.099 | 0.517 | 0.184 | 0.256 | 44.972 | 156 | 188 |
| FXST | 0.176 | 0.112 | -0.043 | 0.395 | 0.114 | 0.204 | 0.003 | 156 | 188 |
| GCC | 0.277 | 0.116 | 0.049 | 0.504 | 0.017 | 0.119 | 6.134 | 156 | 188 |
| IC | 0.137 | 0.111 | -0.081 | 0.355 | 0.219 | 0.274 | 0.000 | 156 | 188 |
| IFO | 0.289 | 0.144 | 0.006 | 0.571 | 0.045 | 0.161 | 35.149 | 156 | 188 |
| PCR | 0.271 | 0.115 | 0.046 | 0.497 | 0.018 | 0.119 | 4.764 | 156 | 188 |
| PLIC | -0.089 | 0.111 | -0.307 | 0.128 | 0.421 | 0.478 | 0.000 | 156 | 188 |
| PTR | 0.284 | 0.134 | 0.022 | 0.546 | 0.034 | 0.161 | 25.767 | 156 | 188 |
| RLIC | 0.193 | 0.141 | -0.084 | 0.470 | 0.172 | 0.253 | 33.268 | 156 | 188 |
| SCC | 0.131 | 0.111 | -0.087 | 0.349 | 0.239 | 0.285 | 0.000 | 156 | 188 |
| SCR | 0.207 | 0.119 | -0.026 | 0.439 | 0.082 | 0.186 | 9.697 | 156 | 188 |
| SFO | 0.190 | 0.115 | -0.036 | 0.416 | 0.099 | 0.190 | 5.088 | 156 | 188 |
| SLF | 0.271 | 0.112 | 0.052 | 0.489 | 0.015 | 0.119 | 0.000 | 156 | 188 |
| SS | 0.096 | 0.164 | -0.225 | 0.417 | 0.557 | 0.605 | 49.719 | 156 | 188 |
| UNC | 0.207 | 0.124 | -0.035 | 0.450 | 0.094 | 0.190 | 15.809 | 156 | 188 |
| AverageMD | 0.252 | 0.143 | -0.028 | 0.531 | 0.078 | 0.253 | 34.354 | 156 | 188 |

Table S87. Cohen’s d effect sizes after meta-analysis, for RD differences between non-antidepressant users and healthy controls **in adolescents only**. Age, sex, agexsex, age^2^, age^2^xsex and scansite included as covariates.

| Region | Cohen’s d | SE | CI LB | CI UB | P-value | FDR P-value | I^2^ | Controls | Patients |
| --- | --- | --- | --- | --- | --- | --- | --- | --- | --- |
| ACR | 0.220 | 0.181 | -0.136 | 0.575 | 0.226 | 0.435 | 58.371 | 156 | 188 |
| ALIC | 0.136 | 0.198 | -0.252 | 0.525 | 0.491 | 0.682 | 65.038 | 156 | 188 |
| BCC | 0.308 | 0.112 | 0.089 | 0.527 | 0.006 | 0.075 | 0.000 | 156 | 188 |
| CC | 0.323 | 0.112 | 0.104 | 0.542 | 0.004 | 0.075 | 0.000 | 156 | 188 |
| CGC | 0.052 | 0.190 | -0.319 | 0.424 | 0.783 | 0.851 | 62.114 | 156 | 188 |
| CGH | -0.107 | 0.268 | -0.633 | 0.419 | 0.691 | 0.823 | 80.860 | 156 | 188 |
| CR | 0.257 | 0.163 | -0.061 | 0.576 | 0.113 | 0.298 | 48.630 | 156 | 188 |
| CST | 0.080 | 0.127 | -0.170 | 0.330 | 0.530 | 0.697 | 20.066 | 156 | 188 |
| EC | 0.045 | 0.204 | -0.355 | 0.444 | 0.825 | 0.859 | 67.092 | 156 | 188 |
| FX | 0.208 | 0.129 | -0.045 | 0.460 | 0.107 | 0.298 | 21.001 | 156 | 188 |
| FXST | 0.065 | 0.200 | -0.327 | 0.457 | 0.745 | 0.847 | 65.774 | 156 | 188 |
| GCC | 0.347 | 0.146 | 0.061 | 0.633 | 0.017 | 0.106 | 36.625 | 156 | 188 |
| IC | 0.091 | 0.126 | -0.155 | 0.337 | 0.470 | 0.682 | 18.235 | 156 | 188 |
| IFO | 0.301 | 0.187 | -0.065 | 0.667 | 0.107 | 0.298 | 60.530 | 156 | 188 |
| PCR | 0.278 | 0.113 | 0.058 | 0.499 | 0.013 | 0.106 | 1.228 | 156 | 188 |
| PLIC | -0.124 | 0.111 | -0.342 | 0.094 | 0.265 | 0.442 | 0.000 | 156 | 188 |
| PTR | 0.226 | 0.136 | -0.041 | 0.492 | 0.097 | 0.298 | 28.466 | 156 | 188 |
| RLIC | 0.153 | 0.131 | -0.104 | 0.409 | 0.244 | 0.436 | 23.702 | 156 | 188 |
| SCC | 0.173 | 0.111 | -0.045 | 0.392 | 0.119 | 0.298 | 0.000 | 156 | 188 |
| SCR | 0.211 | 0.171 | -0.124 | 0.546 | 0.218 | 0.435 | 53.524 | 156 | 188 |
| SFO | 0.158 | 0.187 | -0.209 | 0.525 | 0.398 | 0.622 | 60.995 | 156 | 188 |
| SLF | 0.215 | 0.148 | -0.074 | 0.504 | 0.145 | 0.330 | 38.441 | 156 | 188 |
| SS | -0.009 | 0.202 | -0.405 | 0.388 | 0.966 | 0.966 | 66.667 | 156 | 188 |
| UNC | 0.079 | 0.155 | -0.225 | 0.383 | 0.611 | 0.764 | 43.955 | 156 | 188 |
| AverageRD | 0.275 | 0.170 | -0.058 | 0.608 | 0.105 | 0.298 | 52.714 | 156 | 188 |

Table S88. Cohen’s d effect sizes after meta-analysis, for FA differences between non-antidepressant users and antidepressant users **in adolescents only**. Age, sex, agexsex, age^2^, age^2^xsex and scansite included as covariates.

| Region | Cohen’s d | SE | CI LB | CI UB | P-value | FDR P-value | I^2^ | Controls | Patients |
| --- | --- | --- | --- | --- | --- | --- | --- | --- | --- |
| ACR | 0.303 | 0.237 | -0.162 | 0.769 | 0.202 | 0.786 | 52.749 | 112 | 104 |
| ALIC | 0.034 | 0.145 | -0.249 | 0.318 | 0.812 | 0.967 | 0.000 | 112 | 104 |
| BCC | 0.090 | 0.145 | -0.194 | 0.374 | 0.537 | 0.967 | 0.005 | 112 | 104 |
| CC | 0.120 | 0.145 | -0.164 | 0.404 | 0.407 | 0.848 | 0.000 | 112 | 104 |
| CGC | 0.185 | 0.145 | -0.099 | 0.469 | 0.202 | 0.786 | 0.000 | 112 | 104 |
| CGH | -0.024 | 0.224 | -0.462 | 0.415 | 0.916 | 0.967 | 47.405 | 112 | 104 |
| CR | 0.209 | 0.209 | -0.200 | 0.618 | 0.317 | 0.848 | 40.873 | 112 | 104 |
| CST | 0.287 | 0.146 | 0.002 | 0.572 | 0.049 | 0.613 | 0.000 | 112 | 104 |
| EC | 0.020 | 0.288 | -0.544 | 0.584 | 0.945 | 0.967 | 67.427 | 112 | 104 |
| FX | 0.027 | 0.145 | -0.257 | 0.310 | 0.854 | 0.967 | 0.000 | 112 | 104 |
| FXST | -0.126 | 0.145 | -0.409 | 0.158 | 0.385 | 0.848 | 0.000 | 112 | 104 |
| GCC | 0.295 | 0.145 | 0.010 | 0.580 | 0.042 | 0.613 | 0.000 | 112 | 104 |
| IC | -0.107 | 0.305 | -0.704 | 0.491 | 0.726 | 0.967 | 70.554 | 112 | 104 |
| IFO | 0.090 | 0.183 | -0.269 | 0.449 | 0.623 | 0.967 | 27.182 | 112 | 104 |
| PCR | 0.195 | 0.145 | -0.089 | 0.479 | 0.178 | 0.786 | 0.000 | 112 | 104 |
| PLIC | -0.212 | 0.173 | -0.551 | 0.127 | 0.220 | 0.786 | 19.970 | 112 | 104 |
| PTR | 0.012 | 0.282 | -0.540 | 0.564 | 0.967 | 0.967 | 65.918 | 112 | 104 |
| RLIC | -0.102 | 0.487 | -1.058 | 0.853 | 0.834 | 0.967 | 88.146 | 112 | 104 |
| SCC | -0.053 | 0.259 | -0.561 | 0.455 | 0.838 | 0.967 | 59.975 | 112 | 104 |
| SCR | 0.008 | 0.145 | -0.276 | 0.292 | 0.957 | 0.967 | 0.004 | 112 | 104 |
| SFO | 0.126 | 0.145 | -0.158 | 0.410 | 0.384 | 0.848 | 0.000 | 112 | 104 |
| SLF | 0.029 | 0.145 | -0.254 | 0.313 | 0.840 | 0.967 | 0.000 | 112 | 104 |
| SS | -0.340 | 0.256 | -0.842 | 0.162 | 0.185 | 0.786 | 58.140 | 112 | 104 |
| UNC | -0.148 | 0.145 | -0.433 | 0.137 | 0.308 | 0.848 | 0.000 | 112 | 104 |
| AverageFA | -0.044 | 0.145 | -0.327 | 0.240 | 0.763 | 0.967 | 0.000 | 112 | 104 |

Table S89. Cohen’s d effect sizes after meta-analysis, for AD differences between non-antidepressant users and antidepressant users **in adolescents only**. Age, sex, agexsex, age^2^, age^2^xsex and scansite included as covariates.

| Region | Cohen’s d | SE | CI LB | CI UB | P-value | FDR P-value | I^2^ | Controls | Patients |
| --- | --- | --- | --- | --- | --- | --- | --- | --- | --- |
| ACR | 0.035 | 0.145 | -0.249 | 0.318 | 0.812 | 0.822 | 0.000 | 112 | 104 |
| ALIC | 0.160 | 0.193 | -0.219 | 0.539 | 0.409 | 0.530 | 32.999 | 112 | 104 |
| BCC | 0.362 | 0.255 | -0.138 | 0.862 | 0.156 | 0.467 | 58.042 | 112 | 104 |
| CC | 0.276 | 0.150 | -0.018 | 0.570 | 0.066 | 0.375 | 3.717 | 112 | 104 |
| CGC | 0.293 | 0.231 | -0.160 | 0.746 | 0.206 | 0.468 | 50.418 | 112 | 104 |
| CGH | 0.286 | 0.167 | -0.041 | 0.612 | 0.086 | 0.375 | 15.807 | 112 | 104 |
| CR | 0.164 | 0.158 | -0.146 | 0.474 | 0.301 | 0.491 | 10.186 | 112 | 104 |
| CST | 0.390 | 0.242 | -0.085 | 0.865 | 0.107 | 0.382 | 54.067 | 112 | 104 |
| EC | 0.427 | 0.202 | 0.032 | 0.822 | 0.034 | 0.283 | 36.571 | 112 | 104 |
| FX | 0.108 | 0.145 | -0.176 | 0.393 | 0.455 | 0.530 | 0.002 | 112 | 104 |
| FXST | 0.246 | 0.145 | -0.038 | 0.530 | 0.090 | 0.375 | 0.000 | 112 | 104 |
| GCC | 0.071 | 0.145 | -0.214 | 0.355 | 0.627 | 0.682 | 0.002 | 112 | 104 |
| IC | 0.350 | 0.359 | -0.353 | 1.053 | 0.330 | 0.491 | 78.445 | 112 | 104 |
| IFO | 0.181 | 0.187 | -0.186 | 0.547 | 0.334 | 0.491 | 29.236 | 112 | 104 |
| PCR | 0.169 | 0.145 | -0.115 | 0.453 | 0.242 | 0.491 | 0.000 | 112 | 104 |
| PLIC | 0.231 | 0.317 | -0.390 | 0.853 | 0.466 | 0.530 | 72.838 | 112 | 104 |
| PTR | 0.154 | 0.145 | -0.130 | 0.437 | 0.288 | 0.491 | 0.000 | 112 | 104 |
| RLIC | 0.390 | 0.460 | -0.512 | 1.291 | 0.397 | 0.530 | 86.623 | 112 | 104 |
| SCC | 0.374 | 0.146 | 0.089 | 0.660 | 0.010 | 0.238 | 0.006 | 112 | 104 |
| SCR | 0.190 | 0.145 | -0.094 | 0.474 | 0.190 | 0.468 | 0.000 | 112 | 104 |
| SFO | 0.450 | 0.326 | -0.190 | 1.089 | 0.168 | 0.467 | 73.794 | 112 | 104 |
| SLF | 0.248 | 0.225 | -0.193 | 0.688 | 0.270 | 0.491 | 47.894 | 112 | 104 |
| SS | 0.222 | 0.282 | -0.331 | 0.776 | 0.431 | 0.530 | 65.832 | 112 | 104 |
| UNC | -0.032 | 0.144 | -0.316 | 0.251 | 0.822 | 0.822 | 0.000 | 112 | 104 |
| AverageAD | 0.342 | 0.146 | 0.057 | 0.628 | 0.019 | 0.238 | 0.000 | 112 | 104 |

Table S90. Cohen’s d effect sizes after meta-analysis, for MD differences between non-antidepressant users and antidepressant users **in adolescents only**. Age, sex, agexsex, age^2^, age^2^xsex and scansite included as covariates.

| Region | Cohen’s d | SE | CI LB | CI UB | P-value | FDR P-value | I^2^ | Controls | Patients |
| --- | --- | --- | --- | --- | --- | --- | --- | --- | --- |
| ACR | -0.108 | 0.145 | -0.391 | 0.176 | 0.457 | 0.672 | 0.002 | 112 | 104 |
| ALIC | 0.119 | 0.155 | -0.185 | 0.424 | 0.442 | 0.672 | 8.089 | 112 | 104 |
| BCC | 0.024 | 0.145 | -0.259 | 0.308 | 0.868 | 0.886 | 0.000 | 112 | 104 |
| CC | 0.039 | 0.145 | -0.244 | 0.322 | 0.787 | 0.886 | 0.000 | 112 | 104 |
| CGC | 0.230 | 0.145 | -0.054 | 0.514 | 0.113 | 0.310 | 0.000 | 112 | 104 |
| CGH | 0.302 | 0.145 | 0.017 | 0.587 | 0.038 | 0.310 | 0.000 | 112 | 104 |
| CR | -0.028 | 0.145 | -0.311 | 0.256 | 0.848 | 0.886 | 0.000 | 112 | 104 |
| CST | 0.379 | 0.270 | -0.150 | 0.907 | 0.161 | 0.310 | 62.071 | 112 | 104 |
| EC | 0.251 | 0.145 | -0.033 | 0.536 | 0.083 | 0.310 | 0.000 | 112 | 104 |
| FX | 0.073 | 0.145 | -0.211 | 0.357 | 0.616 | 0.856 | 0.010 | 112 | 104 |
| FXST | 0.299 | 0.145 | 0.014 | 0.584 | 0.040 | 0.310 | 0.000 | 112 | 104 |
| GCC | -0.131 | 0.145 | -0.414 | 0.153 | 0.366 | 0.654 | 0.000 | 112 | 104 |
| IC | 0.329 | 0.224 | -0.111 | 0.768 | 0.143 | 0.310 | 46.988 | 112 | 104 |
| IFO | -0.021 | 0.145 | -0.305 | 0.263 | 0.886 | 0.886 | 0.005 | 112 | 104 |
| PCR | -0.025 | 0.144 | -0.308 | 0.258 | 0.862 | 0.886 | 0.000 | 112 | 104 |
| PLIC | 0.306 | 0.179 | -0.044 | 0.657 | 0.087 | 0.310 | 23.645 | 112 | 104 |
| PTR | 0.093 | 0.226 | -0.350 | 0.537 | 0.680 | 0.875 | 48.641 | 112 | 104 |
| RLIC | 0.207 | 0.145 | -0.078 | 0.492 | 0.154 | 0.310 | 0.217 | 112 | 104 |
| SCC | 0.211 | 0.145 | -0.073 | 0.495 | 0.144 | 0.310 | 0.000 | 112 | 104 |
| SCR | 0.112 | 0.145 | -0.172 | 0.396 | 0.440 | 0.672 | 0.001 | 112 | 104 |
| SFO | 0.382 | 0.262 | -0.132 | 0.897 | 0.145 | 0.310 | 60.281 | 112 | 104 |
| SLF | 0.224 | 0.145 | -0.060 | 0.508 | 0.122 | 0.310 | 0.000 | 112 | 104 |
| SS | 0.312 | 0.145 | 0.028 | 0.597 | 0.032 | 0.310 | 0.000 | 112 | 104 |
| UNC | 0.056 | 0.145 | -0.228 | 0.340 | 0.700 | 0.875 | 0.003 | 112 | 104 |
| AverageMD | 0.252 | 0.145 | -0.033 | 0.536 | 0.083 | 0.310 | 0.000 | 112 | 104 |

Table S91. Cohen’s d effect sizes after meta-analysis, for RD differences between non-antidepressant users and antidepressant users **in adolescents only**. Age, sex, agexsex, age^2^, age^2^xsex and scansite included as covariates.

| Region | Cohen’s d | SE | CI LB | CI UB | P-value | FDR P-value | I^2^ | Controls | Patients |
| --- | --- | --- | --- | --- | --- | --- | --- | --- | --- |
| ACR | -0.197 | 0.203 | -0.595 | 0.200 | 0.330 | 0.745 | 37.970 | 112 | 104 |
| ALIC | 0.041 | 0.145 | -0.242 | 0.324 | 0.776 | 0.808 | 0.000 | 112 | 104 |
| BCC | -0.091 | 0.145 | -0.375 | 0.193 | 0.530 | 0.745 | 0.000 | 112 | 104 |
| CC | -0.088 | 0.145 | -0.372 | 0.195 | 0.542 | 0.745 | 0.000 | 112 | 104 |
| CGC | 0.078 | 0.175 | -0.266 | 0.421 | 0.657 | 0.808 | 22.292 | 112 | 104 |
| CGH | 0.263 | 0.146 | -0.022 | 0.548 | 0.071 | 0.592 | 0.005 | 112 | 104 |
| CR | -0.119 | 0.145 | -0.403 | 0.165 | 0.412 | 0.745 | 0.002 | 112 | 104 |
| CST | 0.259 | 0.305 | -0.339 | 0.856 | 0.396 | 0.745 | 70.131 | 112 | 104 |
| EC | 0.084 | 0.145 | -0.200 | 0.368 | 0.561 | 0.745 | 0.000 | 112 | 104 |
| FX | 0.043 | 0.145 | -0.241 | 0.327 | 0.764 | 0.808 | 0.001 | 112 | 104 |
| FXST | 0.230 | 0.145 | -0.054 | 0.514 | 0.112 | 0.679 | 0.000 | 112 | 104 |
| GCC | -0.208 | 0.145 | -0.492 | 0.076 | 0.152 | 0.679 | 0.000 | 112 | 104 |
| IC | 0.239 | 0.268 | -0.287 | 0.765 | 0.372 | 0.745 | 61.883 | 112 | 104 |
| IFO | -0.124 | 0.145 | -0.407 | 0.160 | 0.393 | 0.745 | 0.000 | 112 | 104 |
| PCR | -0.112 | 0.145 | -0.395 | 0.172 | 0.440 | 0.745 | 0.000 | 112 | 104 |
| PLIC | 0.266 | 0.146 | -0.019 | 0.552 | 0.067 | 0.592 | 0.002 | 112 | 104 |
| PTR | 0.043 | 0.273 | -0.491 | 0.578 | 0.874 | 0.874 | 63.685 | 112 | 104 |
| RLIC | 0.247 | 0.431 | -0.598 | 1.093 | 0.566 | 0.745 | 84.845 | 112 | 104 |
| SCC | 0.060 | 0.161 | -0.255 | 0.376 | 0.707 | 0.808 | 12.200 | 112 | 104 |
| SCR | 0.077 | 0.225 | -0.365 | 0.519 | 0.733 | 0.808 | 47.981 | 112 | 104 |
| SFO | 0.146 | 0.150 | -0.148 | 0.440 | 0.331 | 0.745 | 3.808 | 112 | 104 |
| SLF | 0.165 | 0.158 | -0.144 | 0.475 | 0.295 | 0.745 | 10.106 | 112 | 104 |
| SS | 0.298 | 0.146 | 0.013 | 0.583 | 0.041 | 0.592 | 0.002 | 112 | 104 |
| UNC | 0.195 | 0.275 | -0.343 | 0.734 | 0.477 | 0.745 | 63.670 | 112 | 104 |
| AverageRD | 0.202 | 0.145 | -0.082 | 0.487 | 0.163 | 0.679 | 0.000 | 112 | 104 |

**8: Severity**

Table S92. Beta’s from linear regression analyses examining the association between FA values and severity of symptoms at study inclusion measured by the BDI-II **in adults only**. Age, sex, agexsex, age^2^, age^2^xsex and scansite included as covariates.

| Region | Beta | SE | CI LB | CI UB | P-value | FDR P-value | I^2^ | N |
| --- | --- | --- | --- | --- | --- | --- | --- | --- |
| ACR | 0.000313 | 0.000187 | -0.000053 | 0.000679 | 0.093 | 0.883 | 34.109 | 477 |
| ALIC | 0.000088 | 0.000139 | -0.000184 | 0.000360 | 0.528 | 0.975 | 19.424 | 477 |
| BCC | 0.000020 | 0.000165 | -0.000304 | 0.000344 | 0.904 | 0.975 | 0.000 | 477 |
| CC | 0.000006 | 0.000123 | -0.000235 | 0.000246 | 0.963 | 0.975 | 0.276 | 477 |
| CGC | 0.000029 | 0.000261 | -0.000482 | 0.000539 | 0.913 | 0.975 | 41.845 | 477 |
| CGH | -0.000359 | 0.000326 | -0.000997 | 0.000279 | 0.269 | 0.975 | 50.839 | 477 |
| CR | 0.000165 | 0.000131 | -0.000091 | 0.000420 | 0.208 | 0.975 | 17.340 | 477 |
| CST | -0.000346 | 0.000502 | -0.001331 | 0.000638 | 0.491 | 0.975 | 81.332 | 477 |
| EC | 0.000018 | 0.000202 | -0.000378 | 0.000414 | 0.928 | 0.975 | 56.521 | 477 |
| FX | -0.000419 | 0.000259 | -0.000926 | 0.000089 | 0.106 | 0.883 | 0.000 | 477 |
| FXST | -0.000389 | 0.000214 | -0.000809 | 0.000031 | 0.069 | 0.883 | 31.235 | 477 |
| GCC | -0.000075 | 0.000236 | -0.000537 | 0.000388 | 0.752 | 0.975 | 47.584 | 477 |
| IC | -0.000019 | 0.000096 | -0.000206 | 0.000169 | 0.847 | 0.975 | 0.000 | 477 |
| IFO | -0.000330 | 0.000487 | -0.001285 | 0.000625 | 0.498 | 0.975 | 78.024 | 477 |
| PCR | 0.000054 | 0.000112 | -0.000167 | 0.000274 | 0.633 | 0.975 | 0.000 | 477 |
| PLIC | -0.000032 | 0.000124 | -0.000276 | 0.000211 | 0.794 | 0.975 | 0.000 | 477 |
| PTR | -0.000131 | 0.000133 | -0.000392 | 0.000130 | 0.326 | 0.975 | 0.000 | 477 |
| RLIC | -0.000129 | 0.000118 | -0.000361 | 0.000103 | 0.276 | 0.975 | 0.000 | 477 |
| SCC | 0.000055 | 0.000099 | -0.000139 | 0.000249 | 0.576 | 0.975 | 0.000 | 477 |
| SCR | 0.000208 | 0.000215 | -0.000214 | 0.000631 | 0.333 | 0.975 | 53.738 | 477 |
| SFO | 0.000122 | 0.000365 | -0.000593 | 0.000837 | 0.738 | 0.975 | 71.573 | 477 |
| SLF | 0.000045 | 0.000114 | -0.000178 | 0.000267 | 0.695 | 0.975 | 0.000 | 477 |
| SS | 0.000050 | 0.000119 | -0.000183 | 0.000282 | 0.677 | 0.975 | 0.000 | 477 |
| UNC | -0.000023 | 0.000232 | -0.000477 | 0.000431 | 0.921 | 0.975 | 5.682 | 477 |
| AverageFA | 0.000003 | 0.000078 | -0.000151 | 0.000156 | 0.975 | 0.975 | 5.459 | 477 |

Table S93. Beta’s from linear regression analyses examining the association between AD values and severity of symptoms at study inclusion measured by the BDI-II **in adults only**. Age, sex, agexsex, age^2^, age^2^xsex and scansite included as covariates.

| Region | Beta | SE | CI LB | CI UB | P-value | FDR P-value | I^2^ | N |
| --- | --- | --- | --- | --- | --- | --- | --- | --- |
| ACR | 0.00000029 | 0.00000031 | -0.00000032 | 0.00000090 | 0.348 | 0.725 | 36.477 | 477 |
| ALIC | 0.00000003 | 0.00000032 | -0.00000060 | 0.00000066 | 0.930 | 0.997 | 47.114 | 477 |
| BCC | 0.00000048 | 0.00000026 | -0.00000004 | 0.00000099 | 0.068 | 0.350 | 12.703 | 477 |
| CC | 0.00000027 | 0.00000034 | -0.00000039 | 0.00000094 | 0.420 | 0.775 | 53.539 | 477 |
| CGC | 0.00000064 | 0.00000034 | -0.00000002 | 0.00000129 | 0.059 | 0.350 | 28.360 | 477 |
| CGH | -0.00000002 | 0.00000035 | -0.00000070 | 0.00000066 | 0.957 | 0.997 | 19.360 | 477 |
| CR | 0.00000025 | 0.00000020 | -0.00000013 | 0.00000064 | 0.193 | 0.603 | 11.586 | 477 |
| CST | 0.00000003 | 0.00000036 | -0.00000068 | 0.00000073 | 0.944 | 0.997 | 25.842 | 477 |
| EC | 0.00000009 | 0.00000016 | -0.00000022 | 0.00000040 | 0.581 | 0.854 | 6.381 | 477 |
| FX | -0.00000142 | 0.00000151 | -0.00000438 | 0.00000154 | 0.347 | 0.725 | 38.919 | 477 |
| FXST | 0.00000012 | 0.00000048 | -0.00000082 | 0.00000107 | 0.798 | 0.997 | 73.742 | 477 |
| GCC | -0.00000010 | 0.00000028 | -0.00000065 | 0.00000045 | 0.715 | 0.993 | 12.223 | 477 |
| IC | -0.00000002 | 0.00000030 | -0.00000060 | 0.00000057 | 0.955 | 0.997 | 49.780 | 477 |
| IFO | 0.00000065 | 0.00000029 | 0.00000008 | 0.00000121 | 0.024 | 0.300 | 18.934 | 477 |
| PCR | 0.00000033 | 0.00000018 | -0.00000003 | 0.00000069 | 0.070 | 0.350 | 0.000 | 477 |
| PLIC | 0.00000005 | 0.00000049 | -0.00000092 | 0.00000101 | 0.923 | 0.997 | 66.114 | 477 |
| PTR | 0.00000023 | 0.00000030 | -0.00000035 | 0.00000082 | 0.434 | 0.775 | 32.276 | 477 |
| RLIC | -0.00000025 | 0.00000020 | -0.00000065 | 0.00000015 | 0.221 | 0.614 | 5.631 | 477 |
| SCC | 0.00000026 | 0.00000038 | -0.00000048 | 0.00000101 | 0.487 | 0.812 | 44.846 | 477 |
| SCR | 0.00000027 | 0.00000018 | -0.00000009 | 0.00000063 | 0.137 | 0.571 | 0.000 | 477 |
| SFO | 0.00000000 | 0.00000022 | -0.00000044 | 0.00000044 | 1.000 | 1.000 | 0.000 | 477 |
| SLF | 0.00000021 | 0.00000019 | -0.00000017 | 0.00000058 | 0.283 | 0.708 | 14.784 | 477 |
| SS | 0.00000014 | 0.00000024 | -0.00000033 | 0.00000061 | 0.560 | 0.854 | 18.056 | 477 |
| UNC | 0.00000108 | 0.00000036 | 0.00000037 | 0.00000178 | 0.003 | 0.075 | 0.000 | 477 |
| AverageAD | 0.00000016 | 0.00000012 | -0.00000007 | 0.00000040 | 0.175 | 0.603 | 15.709 | 477 |

Table S94. Beta’s from linear regression analyses examining the association between MD values and severity of symptoms at study inclusion measured by the BDI-II **in adults only**. Age, sex, agexsex, age^2^, age^2^xsex and scansite included as covariates.

| Region | Beta | SE | CI LB | CI UB | P-value | FDR P-value | I^2^ | N |
| --- | --- | --- | --- | --- | --- | --- | --- | --- |
| ACR | -0.00000001 | 0.00000031 | -0.00000061 | 0.00000059 | 0.975 | 0.975 | 61.795 | 477 |
| ALIC | -0.00000006 | 0.00000012 | -0.00000029 | 0.00000017 | 0.620 | 0.902 | 2.768 | 477 |
| BCC | 0.00000011 | 0.00000018 | -0.00000024 | 0.00000047 | 0.541 | 0.902 | 0.000 | 477 |
| CC | 0.00000005 | 0.00000015 | -0.00000025 | 0.00000035 | 0.745 | 0.902 | 1.767 | 477 |
| CGC | 0.00000016 | 0.00000015 | -0.00000014 | 0.00000046 | 0.297 | 0.902 | 9.121 | 477 |
| CGH | 0.00000025 | 0.00000020 | -0.00000014 | 0.00000064 | 0.207 | 0.902 | 15.588 | 477 |
| CR | 0.00000005 | 0.00000020 | -0.00000034 | 0.00000044 | 0.794 | 0.902 | 41.998 | 477 |
| CST | 0.00000013 | 0.00000024 | -0.00000034 | 0.00000061 | 0.586 | 0.902 | 41.516 | 477 |
| EC | 0.00000003 | 0.00000011 | -0.00000019 | 0.00000025 | 0.794 | 0.902 | 0.000 | 477 |
| FX | -0.00000023 | 0.00000112 | -0.00000243 | 0.00000197 | 0.838 | 0.908 | 11.894 | 477 |
| FXST | 0.00000017 | 0.00000013 | -0.00000009 | 0.00000043 | 0.191 | 0.902 | 14.567 | 477 |
| GCC | 0.00000003 | 0.00000017 | -0.00000031 | 0.00000037 | 0.872 | 0.908 | 0.000 | 477 |
| IC | -0.00000008 | 0.00000010 | -0.00000027 | 0.00000012 | 0.441 | 0.902 | 0.000 | 477 |
| IFO | 0.00000043 | 0.00000034 | -0.00000025 | 0.00000111 | 0.213 | 0.902 | 78.643 | 477 |
| PCR | 0.00000013 | 0.00000013 | -0.00000012 | 0.00000038 | 0.301 | 0.902 | 0.000 | 477 |
| PLIC | -0.00000004 | 0.00000013 | -0.00000028 | 0.00000021 | 0.761 | 0.902 | 0.000 | 477 |
| PTR | 0.00000016 | 0.00000016 | -0.00000015 | 0.00000047 | 0.316 | 0.902 | 11.768 | 477 |
| RLIC | -0.00000012 | 0.00000011 | -0.00000033 | 0.00000010 | 0.298 | 0.902 | 0.000 | 477 |
| SCC | -0.00000005 | 0.00000016 | -0.00000037 | 0.00000027 | 0.775 | 0.902 | 15.737 | 477 |
| SCR | 0.00000011 | 0.00000010 | -0.00000009 | 0.00000031 | 0.284 | 0.902 | 0.000 | 477 |
| SFO | 0.00000005 | 0.00000015 | -0.00000023 | 0.00000034 | 0.718 | 0.902 | 0.000 | 477 |
| SLF | 0.00000003 | 0.00000010 | -0.00000016 | 0.00000023 | 0.737 | 0.902 | 0.000 | 477 |
| SS | 0.00000009 | 0.00000016 | -0.00000023 | 0.00000040 | 0.585 | 0.902 | 20.731 | 477 |
| UNC | 0.00000022 | 0.00000024 | -0.00000025 | 0.00000070 | 0.353 | 0.902 | 0.000 | 477 |
| AverageMD | 0.00000005 | 0.00000009 | -0.00000013 | 0.00000024 | 0.562 | 0.902 | 0.000 | 477 |

Table S95. Beta’s from linear regression analyses examining the association between RD values and severity of symptoms at study inclusion measured by the BDI-II **in adults only**. Age, sex, agexsex, age^2^, age^2^xsex and scansite included as covariates.

| Region | Beta | SE | CI LB | CI UB | P-value | FDR P-value | I^2^ | N |
| --- | --- | --- | --- | --- | --- | --- | --- | --- |
| ACR | -0.00000020 | 0.00000029 | -0.00000077 | 0.00000037 | 0.498 | 0.980 | 52.755 | 477 |
| ALIC | -0.00000007 | 0.00000013 | -0.00000032 | 0.00000018 | 0.575 | 0.980 | 4.812 | 477 |
| BCC | 0.00000004 | 0.00000022 | -0.00000038 | 0.00000047 | 0.839 | 0.980 | 0.000 | 477 |
| CC | 0.00000000 | 0.00000017 | -0.00000032 | 0.00000033 | 0.980 | 0.980 | 0.000 | 477 |
| CGC | 0.00000004 | 0.00000017 | -0.00000029 | 0.00000036 | 0.828 | 0.980 | 0.868 | 477 |
| CGH | 0.00000037 | 0.00000023 | -0.00000008 | 0.00000082 | 0.110 | 0.980 | 18.732 | 477 |
| CR | -0.00000012 | 0.00000024 | -0.00000059 | 0.00000036 | 0.629 | 0.980 | 53.225 | 477 |
| CST | 0.00000016 | 0.00000024 | -0.00000031 | 0.00000064 | 0.504 | 0.980 | 24.443 | 477 |
| EC | 0.00000004 | 0.00000013 | -0.00000021 | 0.00000028 | 0.775 | 0.980 | 0.453 | 477 |
| FX | 0.00000013 | 0.00000107 | -0.00000197 | 0.00000223 | 0.906 | 0.980 | 5.302 | 477 |
| FXST | 0.00000032 | 0.00000014 | 0.00000004 | 0.00000060 | 0.027 | 0.675 | 1.994 | 477 |
| GCC | -0.00000002 | 0.00000018 | -0.00000038 | 0.00000034 | 0.911 | 0.980 | 9.148 | 477 |
| IC | -0.00000004 | 0.00000010 | -0.00000024 | 0.00000017 | 0.724 | 0.980 | 0.000 | 477 |
| IFO | 0.00000042 | 0.00000045 | -0.00000047 | 0.00000130 | 0.356 | 0.980 | 81.838 | 477 |
| PCR | 0.00000002 | 0.00000014 | -0.00000025 | 0.00000030 | 0.870 | 0.980 | 0.000 | 477 |
| PLIC | 0.00000001 | 0.00000013 | -0.00000024 | 0.00000027 | 0.931 | 0.980 | 2.776 | 477 |
| PTR | 0.00000016 | 0.00000015 | -0.00000013 | 0.00000046 | 0.270 | 0.980 | 0.000 | 477 |
| RLIC | 0.00000003 | 0.00000013 | -0.00000023 | 0.00000028 | 0.835 | 0.980 | 0.000 | 477 |
| SCC | -0.00000012 | 0.00000014 | -0.00000039 | 0.00000015 | 0.382 | 0.980 | 0.000 | 477 |
| SCR | -0.00000001 | 0.00000022 | -0.00000043 | 0.00000042 | 0.974 | 0.980 | 52.034 | 477 |
| SFO | -0.00000007 | 0.00000026 | -0.00000058 | 0.00000045 | 0.799 | 0.980 | 47.407 | 477 |
| SLF | -0.00000001 | 0.00000012 | -0.00000025 | 0.00000022 | 0.908 | 0.980 | 0.000 | 477 |
| SS | 0.00000021 | 0.00000020 | -0.00000019 | 0.00000060 | 0.309 | 0.980 | 42.332 | 477 |
| UNC | 0.00000008 | 0.00000028 | -0.00000046 | 0.00000062 | 0.773 | 0.980 | 0.000 | 477 |
| AverageRD | 0.00000006 | 0.00000010 | -0.00000014 | 0.00000026 | 0.533 | 0.980 | 0.000 | 477 |

Table S96. Beta’s from linear regression analyses examining the association between FA values and severity of symptoms at study inclusion measured by the HDRS **in adults only**. Age, sex, agexsex, age^2^, age^2^xsex and scansite included as covariates.

| Region | Beta | SE | CI LB | CI UB | P-value | FDR P-value | I^2^ | N |
| --- | --- | --- | --- | --- | --- | --- | --- | --- |
| ACR | -0.000185 | 0.000177 | -0.000532 | 0.000162 | 0.297 | 0.825 | 0.000 | 603 |
| ALIC | -0.000103 | 0.000162 | -0.000420 | 0.000214 | 0.525 | 0.866 | 0.000 | 603 |
| BCC | -0.000020 | 0.000240 | -0.000489 | 0.000450 | 0.934 | 0.990 | 0.000 | 603 |
| CC | -0.000019 | 0.000178 | -0.000368 | 0.000330 | 0.916 | 0.990 | 0.000 | 603 |
| CGC | -0.000116 | 0.000238 | -0.000582 | 0.000350 | 0.627 | 0.871 | 0.000 | 603 |
| CGH | -0.000694 | 0.000280 | -0.001243 | -0.000144 | 0.013 | 0.325 | 0.000 | 603 |
| CR | -0.000221 | 0.000146 | -0.000507 | 0.000065 | 0.129 | 0.616 | 0.000 | 603 |
| CST | 0.000057 | 0.000266 | -0.000464 | 0.000577 | 0.831 | 0.989 | 0.000 | 603 |
| EC | -0.000193 | 0.000146 | -0.000479 | 0.000093 | 0.186 | 0.616 | 0.000 | 603 |
| FX | -0.000108 | 0.000414 | -0.000919 | 0.000703 | 0.794 | 0.989 | 0.000 | 603 |
| FXST | 0.000006 | 0.000226 | -0.000436 | 0.000448 | 0.979 | 0.990 | 0.000 | 603 |
| GCC | -0.000167 | 0.000207 | -0.000572 | 0.000238 | 0.420 | 0.866 | 0.000 | 603 |
| IC | -0.000120 | 0.000148 | -0.000410 | 0.000170 | 0.417 | 0.866 | 0.000 | 603 |
| IFO | 0.000420 | 0.000776 | -0.001101 | 0.001940 | 0.589 | 0.866 | 81.289 | 603 |
| PCR | -0.000159 | 0.000172 | -0.000495 | 0.000178 | 0.355 | 0.866 | 0.000 | 603 |
| PLIC | 0.000002 | 0.000193 | -0.000376 | 0.000381 | 0.990 | 0.990 | 0.000 | 603 |
| PTR | -0.000385 | 0.000204 | -0.000785 | 0.000014 | 0.059 | 0.616 | 0.000 | 603 |
| RLIC | -0.000241 | 0.000187 | -0.000607 | 0.000125 | 0.197 | 0.616 | 0.000 | 603 |
| SCC | 0.000040 | 0.000154 | -0.000263 | 0.000343 | 0.795 | 0.989 | 0.000 | 603 |
| SCR | -0.000227 | 0.000163 | -0.000546 | 0.000092 | 0.164 | 0.616 | 0.000 | 603 |
| SFO | -0.000316 | 0.000227 | -0.000760 | 0.000128 | 0.163 | 0.616 | 0.000 | 603 |
| SLF | -0.000263 | 0.000161 | -0.000579 | 0.000053 | 0.103 | 0.616 | 0.000 | 603 |
| SS | 0.000151 | 0.000232 | -0.000303 | 0.000606 | 0.514 | 0.866 | 19.417 | 603 |
| UNC | -0.000168 | 0.000305 | -0.000766 | 0.000429 | 0.581 | 0.866 | 0.000 | 603 |
| AverageFA | -0.000080 | 0.000109 | -0.000293 | 0.000134 | 0.463 | 0.866 | 0.000 | 603 |

Table S97. Beta’s from linear regression analyses examining the association between AD values and severity of symptoms at study inclusion measured by the HDRS **in adults only**. Age, sex, agexsex, age^2^, age^2^xsex and scansite included as covariates.

| Region | Beta | SE | CI LB | CI UB | P-value | FDR P-value | I^2^ | N |
| --- | --- | --- | --- | --- | --- | --- | --- | --- |
| ACR | -0.00001038 | 0.00005200 | -0.00011231 | 0.00009154 | 0.842 | 0.960 | 99.993 | 603 |
| ALIC | -0.00000019 | 0.00000040 | -0.00000097 | 0.00000059 | 0.641 | 0.960 | 10.729 | 603 |
| BCC | -0.00000215 | 0.00003042 | -0.00006177 | 0.00005748 | 0.944 | 0.960 | 99.974 | 603 |
| CC | -0.00000078 | 0.00001559 | -0.00003133 | 0.00002978 | 0.960 | 0.960 | 99.917 | 603 |
| CGC | -0.00000139 | 0.00002151 | -0.00004355 | 0.00004076 | 0.948 | 0.960 | 99.942 | 603 |
| CGH | -0.00000104 | 0.00000053 | -0.00000208 | -0.00000001 | 0.049 | 0.417 | 0.000 | 603 |
| CR | -0.00000523 | 0.00003310 | -0.00007011 | 0.00005964 | 0.874 | 0.960 | 99.988 | 603 |
| CST | -0.00000107 | 0.00000052 | -0.00000209 | -0.00000004 | 0.041 | 0.417 | 2.993 | 603 |
| EC | -0.00001045 | 0.00004483 | -0.00009832 | 0.00007742 | 0.816 | 0.960 | 99.995 | 603 |
| FX | -0.00000166 | 0.00000180 | -0.00000519 | 0.00000187 | 0.356 | 0.960 | 0.000 | 603 |
| FXST | -0.00000020 | 0.00000036 | -0.00000090 | 0.00000051 | 0.587 | 0.960 | 0.000 | 603 |
| GCC | -0.00000170 | 0.00002442 | -0.00004956 | 0.00004616 | 0.945 | 0.960 | 99.945 | 603 |
| IC | -0.00000035 | 0.00000042 | -0.00000117 | 0.00000047 | 0.407 | 0.960 | 18.914 | 603 |
| IFO | 0.00000084 | 0.00000043 | 0.00000000 | 0.00000167 | 0.050 | 0.417 | 0.000 | 603 |
| PCR | -0.00000360 | 0.00003225 | -0.00006680 | 0.00005961 | 0.911 | 0.960 | 99.982 | 603 |
| PLIC | -0.00000069 | 0.00000358 | -0.00000770 | 0.00000633 | 0.848 | 0.960 | 98.227 | 603 |
| PTR | -0.00001536 | 0.00006796 | -0.00014857 | 0.00011784 | 0.821 | 0.960 | 99.995 | 603 |
| RLIC | -0.00000023 | 0.00000059 | -0.00000137 | 0.00000092 | 0.700 | 0.960 | 34.301 | 603 |
| SCC | 0.00000013 | 0.00000041 | -0.00000066 | 0.00000093 | 0.740 | 0.960 | 0.000 | 603 |
| SCR | -0.00000032 | 0.00000039 | -0.00000108 | 0.00000043 | 0.402 | 0.960 | 11.357 | 603 |
| SFO | 0.00000016 | 0.00000062 | -0.00000106 | 0.00000138 | 0.800 | 0.960 | 22.960 | 603 |
| SLF | -0.00000297 | 0.00002727 | -0.00005643 | 0.00005048 | 0.913 | 0.960 | 99.985 | 603 |
| SS | -0.00000507 | 0.00004233 | -0.00008803 | 0.00007789 | 0.905 | 0.960 | 99.989 | 603 |
| UNC | 0.00000025 | 0.00000060 | -0.00000094 | 0.00000143 | 0.683 | 0.960 | 0.000 | 603 |
| AverageAD | -0.00000383 | 0.00002612 | -0.00005503 | 0.00004737 | 0.883 | 0.960 | 99.992 | 603 |

Table S98. Beta’s from linear regression analyses examining the association between MD values and severity of symptoms at study inclusion measured by the HDRS **in adults only**. Age, sex, agexsex, age^2^, age^2^xsex and scansite included as covariates.

| Region | Beta | SE | CI LB | CI UB | P-value | FDR P-value | I^2^ | N |
| --- | --- | --- | --- | --- | --- | --- | --- | --- |
| ACR | -0.00001023 | 0.00005202 | -0.00011218 | 0.00009172 | 0.844 | 0.965 | 99.996 | 603 |
| ALIC | -0.00000008 | 0.00000019 | -0.00000046 | 0.00000030 | 0.694 | 0.965 | 0.000 | 603 |
| BCC | -0.00000248 | 0.00003038 | -0.00006201 | 0.00005706 | 0.935 | 0.965 | 99.979 | 603 |
| CC | -0.00000103 | 0.00001554 | -0.00003149 | 0.00002942 | 0.947 | 0.965 | 99.944 | 603 |
| CGC | -0.00000104 | 0.00002155 | -0.00004329 | 0.00004120 | 0.961 | 0.965 | 99.980 | 603 |
| CGH | 0.00000014 | 0.00000041 | -0.00000066 | 0.00000095 | 0.730 | 0.965 | 17.024 | 603 |
| CR | -0.00000510 | 0.00003311 | -0.00007000 | 0.00005980 | 0.878 | 0.965 | 99.993 | 603 |
| CST | -0.00000058 | 0.00000038 | -0.00000132 | 0.00000016 | 0.123 | 0.965 | 5.976 | 603 |
| EC | -0.00001065 | 0.00004481 | -0.00009848 | 0.00007718 | 0.812 | 0.965 | 99.997 | 603 |
| FX | -0.00000212 | 0.00000166 | -0.00000538 | 0.00000114 | 0.203 | 0.965 | 0.000 | 603 |
| FXST | -0.00000017 | 0.00000020 | -0.00000057 | 0.00000023 | 0.397 | 0.965 | 0.000 | 603 |
| GCC | -0.00000186 | 0.00002440 | -0.00004968 | 0.00004596 | 0.939 | 0.965 | 99.967 | 603 |
| IC | -0.00000007 | 0.00000018 | -0.00000042 | 0.00000027 | 0.679 | 0.965 | 0.000 | 603 |
| IFO | -0.00000001 | 0.00000023 | -0.00000047 | 0.00000044 | 0.959 | 0.965 | 0.000 | 603 |
| PCR | -0.00000363 | 0.00003224 | -0.00006682 | 0.00005956 | 0.910 | 0.965 | 99.991 | 603 |
| PLIC | -0.00000014 | 0.00000314 | -0.00000629 | 0.00000601 | 0.965 | 0.965 | 99.328 | 603 |
| PTR | -0.00001578 | 0.00006792 | -0.00014890 | 0.00011734 | 0.816 | 0.965 | 99.998 | 603 |
| RLIC | -0.00000016 | 0.00000020 | -0.00000055 | 0.00000024 | 0.440 | 0.965 | 0.000 | 603 |
| SCC | -0.00000013 | 0.00000025 | -0.00000062 | 0.00000036 | 0.608 | 0.965 | 0.000 | 603 |
| SCR | -0.00000007 | 0.00000018 | -0.00000043 | 0.00000029 | 0.697 | 0.965 | 0.000 | 603 |
| SFO | -0.00000007 | 0.00000027 | -0.00000061 | 0.00000046 | 0.783 | 0.965 | 0.000 | 603 |
| SLF | -0.00000304 | 0.00002726 | -0.00005648 | 0.00005040 | 0.911 | 0.965 | 99.993 | 603 |
| SS | -0.00000570 | 0.00004226 | -0.00008853 | 0.00007712 | 0.893 | 0.965 | 99.995 | 603 |
| UNC | -0.00000035 | 0.00000057 | -0.00000146 | 0.00000076 | 0.533 | 0.965 | 12.650 | 603 |
| AverageMD | -0.00000391 | 0.00002611 | -0.00005510 | 0.00004727 | 0.881 | 0.965 | 99.993 | 603 |

Table S99. Beta’s from linear regression analyses examining the association between RD values and severity of symptoms at study inclusion measured by the HDRS **in adults only**. Age, sex, agexsex, age^2^, age^2^xsex and scansite included as covariates.

| Region | Beta | SE | CI LB | CI UB | P-value | FDR P-value | I^2^ | N |
| --- | --- | --- | --- | --- | --- | --- | --- | --- |
| ACR | -0.00001005 | 0.00005203 | -0.00011203 | 0.00009194 | 0.847 | 0.972 | 99.996 | 603 |
| ALIC | 0.00000003 | 0.00000020 | -0.00000036 | 0.00000042 | 0.883 | 0.972 | 0.000 | 603 |
| BCC | -0.00000257 | 0.00003037 | -0.00006208 | 0.00005695 | 0.933 | 0.972 | 99.970 | 603 |
| CC | -0.00000109 | 0.00001553 | -0.00003153 | 0.00002935 | 0.944 | 0.972 | 99.930 | 603 |
| CGC | -0.00000078 | 0.00002160 | -0.00004311 | 0.00004155 | 0.971 | 0.972 | 99.972 | 603 |
| CGH | 0.00000065 | 0.00000044 | -0.00000021 | 0.00000152 | 0.137 | 0.972 | 16.252 | 603 |
| CR | -0.00000495 | 0.00003313 | -0.00006988 | 0.00005998 | 0.881 | 0.972 | 99.992 | 603 |
| CST | -0.00000034 | 0.00000035 | -0.00000103 | 0.00000035 | 0.339 | 0.972 | 0.000 | 603 |
| EC | -0.00001069 | 0.00004481 | -0.00009852 | 0.00007713 | 0.811 | 0.972 | 99.996 | 603 |
| FX | -0.00000233 | 0.00000168 | -0.00000562 | 0.00000095 | 0.164 | 0.972 | 0.000 | 603 |
| FXST | -0.00000013 | 0.00000026 | -0.00000063 | 0.00000037 | 0.616 | 0.972 | 0.149 | 603 |
| GCC | -0.00000179 | 0.00002441 | -0.00004964 | 0.00004606 | 0.942 | 0.972 | 99.963 | 603 |
| IC | 0.00000007 | 0.00000019 | -0.00000029 | 0.00000044 | 0.692 | 0.972 | 0.000 | 603 |
| IFO | -0.00000022 | 0.00000030 | -0.00000080 | 0.00000036 | 0.463 | 0.972 | 0.000 | 603 |
| PCR | -0.00000358 | 0.00003225 | -0.00006678 | 0.00005963 | 0.912 | 0.972 | 99.990 | 603 |
| PLIC | 0.00000010 | 0.00000294 | -0.00000566 | 0.00000586 | 0.972 | 0.972 | 99.146 | 603 |
| PTR | -0.00001587 | 0.00006791 | -0.00014896 | 0.00011723 | 0.815 | 0.972 | 99.997 | 603 |
| RLIC | 0.00000006 | 0.00000024 | -0.00000040 | 0.00000052 | 0.806 | 0.972 | 0.000 | 603 |
| SCC | -0.00000009 | 0.00000026 | -0.00000059 | 0.00000041 | 0.729 | 0.972 | 0.000 | 603 |
| SCR | 0.00000003 | 0.00000024 | -0.00000044 | 0.00000050 | 0.910 | 0.972 | 9.076 | 603 |
| SFO | -0.00000004 | 0.00000029 | -0.00000060 | 0.00000052 | 0.892 | 0.972 | 0.000 | 603 |
| SLF | -0.00000301 | 0.00002727 | -0.00005645 | 0.00005044 | 0.912 | 0.972 | 99.991 | 603 |
| SS | -0.00000595 | 0.00004223 | -0.00008872 | 0.00007683 | 0.888 | 0.972 | 99.994 | 603 |
| UNC | -0.00000007 | 0.00000054 | -0.00000112 | 0.00000099 | 0.902 | 0.972 | 2.750 | 603 |
| AverageRD | -0.00000388 | 0.00002612 | -0.00005507 | 0.00004730 | 0.882 | 0.972 | 99.992 | 603 |

Table S100. Beta’s from linear regression analyses examining the association between FA values and severity of symptoms at study inclusion measured by the BDI-II **in adolescents only**. Age, sex, agexsex, age^2^, age^2^xsex and scansite included as covariates.

| Region | Beta | SE | CI LB | CI UB | P-value | FDR P-value | I^2^ | N |
| --- | --- | --- | --- | --- | --- | --- | --- | --- |
| ACR | -0.000193 | 0.000186 | -0.000557 | 0.000171 | 0.300 | 0.450 | 11.702 | 168 |
| ALIC | -0.000161 | 0.000158 | -0.000470 | 0.000147 | 0.306 | 0.450 | 0.000 | 168 |
| BCC | -0.000299 | 0.000320 | -0.000926 | 0.000329 | 0.351 | 0.462 | 35.071 | 168 |
| CC | -0.000281 | 0.000216 | -0.000704 | 0.000143 | 0.194 | 0.450 | 27.723 | 168 |
| CGC | -0.000289 | 0.000241 | -0.000761 | 0.000183 | 0.230 | 0.450 | 0.000 | 168 |
| CGH | -0.000172 | 0.000382 | -0.000920 | 0.000576 | 0.653 | 0.764 | 30.705 | 168 |
| CR | -0.000169 | 0.000148 | -0.000459 | 0.000122 | 0.255 | 0.450 | 0.000 | 168 |
| CST | -0.000556 | 0.000272 | -0.001089 | -0.000024 | 0.041 | 0.450 | 0.000 | 168 |
| EC | -0.000048 | 0.000161 | -0.000363 | 0.000266 | 0.764 | 0.764 | 0.000 | 168 |
| FX | 0.000102 | 0.000334 | -0.000553 | 0.000757 | 0.760 | 0.764 | 0.475 | 168 |
| FXST | -0.000287 | 0.000235 | -0.000748 | 0.000175 | 0.223 | 0.450 | 0.000 | 168 |
| GCC | -0.000340 | 0.000303 | -0.000935 | 0.000254 | 0.262 | 0.450 | 68.913 | 168 |
| IC | -0.000148 | 0.000144 | -0.000429 | 0.000134 | 0.304 | 0.450 | 0.000 | 168 |
| IFO | 0.000258 | 0.000269 | -0.000269 | 0.000785 | 0.338 | 0.462 | 0.000 | 168 |
| PCR | -0.000071 | 0.000183 | -0.000429 | 0.000287 | 0.696 | 0.764 | 0.000 | 168 |
| PLIC | -0.000068 | 0.000198 | -0.000456 | 0.000321 | 0.733 | 0.764 | 13.933 | 168 |
| PTR | -0.000204 | 0.000198 | -0.000593 | 0.000184 | 0.303 | 0.450 | 0.000 | 168 |
| RLIC | -0.000250 | 0.000187 | -0.000616 | 0.000117 | 0.182 | 0.450 | 0.000 | 168 |
| SCC | -0.000172 | 0.000146 | -0.000458 | 0.000114 | 0.238 | 0.450 | 0.000 | 168 |
| SCR | -0.000109 | 0.000172 | -0.000446 | 0.000228 | 0.527 | 0.659 | 0.000 | 168 |
| SFO | -0.000327 | 0.000184 | -0.000688 | 0.000034 | 0.076 | 0.450 | 0.000 | 168 |
| SLF | -0.000219 | 0.000170 | -0.000553 | 0.000114 | 0.197 | 0.450 | 0.000 | 168 |
| SS | -0.000250 | 0.000207 | -0.000656 | 0.000156 | 0.227 | 0.450 | 0.000 | 168 |
| UNC | -0.000474 | 0.000288 | -0.001039 | 0.000091 | 0.100 | 0.450 | 0.000 | 168 |
| AverageFA | -0.000114 | 0.000104 | -0.000318 | 0.000090 | 0.275 | 0.450 | 0.000 | 168 |

Table S101. Beta’s from linear regression analyses examining the association between AD values and severity of symptoms at study inclusion measured by the BDI-II **in adolescents only**. Age, sex, agexsex, age^2^, age^2^xsex and scansite included as covariates.

| Region | Beta | SE | CI LB | CI UB | P-value | FDR P-value | I^2^ | N |
| --- | --- | --- | --- | --- | --- | --- | --- | --- |
| ACR | -0.00000010 | 0.00000048 | -0.00000104 | 0.00000085 | 0.839 | 0.957 | 57.247 | 168 |
| ALIC | -0.00000029 | 0.00000026 | -0.00000079 | 0.00000021 | 0.254 | 0.957 | 0.000 | 168 |
| BCC | 0.00000013 | 0.00000040 | -0.00000065 | 0.00000091 | 0.738 | 0.957 | 36.793 | 168 |
| CC | -0.00000019 | 0.00000036 | -0.00000088 | 0.00000051 | 0.600 | 0.957 | 27.706 | 168 |
| CGC | -0.00000035 | 0.00000060 | -0.00000152 | 0.00000082 | 0.558 | 0.957 | 60.051 | 168 |
| CGH | 0.00000015 | 0.00000043 | -0.00000070 | 0.00000099 | 0.736 | 0.957 | 0.000 | 168 |
| CR | -0.00000005 | 0.00000031 | -0.00000065 | 0.00000056 | 0.880 | 0.957 | 43.354 | 168 |
| CST | -0.00000079 | 0.00000043 | -0.00000163 | 0.00000005 | 0.065 | 0.957 | 0.000 | 168 |
| EC | 0.00000008 | 0.00000042 | -0.00000074 | 0.00000089 | 0.852 | 0.957 | 72.621 | 168 |
| FX | -0.00000020 | 0.00000247 | -0.00000505 | 0.00000464 | 0.935 | 0.974 | 65.240 | 168 |
| FXST | -0.00000037 | 0.00000034 | -0.00000104 | 0.00000030 | 0.282 | 0.957 | 0.000 | 168 |
| GCC | -0.00000039 | 0.00000046 | -0.00000129 | 0.00000052 | 0.403 | 0.957 | 45.214 | 168 |
| IC | -0.00000026 | 0.00000023 | -0.00000071 | 0.00000018 | 0.247 | 0.957 | 0.000 | 168 |
| IFO | 0.00000001 | 0.00000035 | -0.00000068 | 0.00000069 | 0.988 | 0.988 | 6.534 | 168 |
| PCR | 0.00000013 | 0.00000041 | -0.00000068 | 0.00000094 | 0.749 | 0.957 | 41.041 | 168 |
| PLIC | -0.00000036 | 0.00000032 | -0.00000099 | 0.00000027 | 0.268 | 0.957 | 13.858 | 168 |
| PTR | 0.00000012 | 0.00000034 | -0.00000055 | 0.00000078 | 0.725 | 0.957 | 0.000 | 168 |
| RLIC | -0.00000031 | 0.00000045 | -0.00000119 | 0.00000056 | 0.482 | 0.957 | 48.726 | 168 |
| SCC | -0.00000028 | 0.00000039 | -0.00000105 | 0.00000049 | 0.475 | 0.957 | 10.299 | 168 |
| SCR | -0.00000013 | 0.00000023 | -0.00000057 | 0.00000031 | 0.571 | 0.957 | 0.000 | 168 |
| SFO | -0.00000027 | 0.00000032 | -0.00000090 | 0.00000037 | 0.411 | 0.957 | 4.940 | 168 |
| SLF | -0.00000016 | 0.00000038 | -0.00000090 | 0.00000059 | 0.681 | 0.957 | 59.600 | 168 |
| SS | -0.00000013 | 0.00000063 | -0.00000136 | 0.00000110 | 0.838 | 0.957 | 65.376 | 168 |
| UNC | 0.00000036 | 0.00000065 | -0.00000091 | 0.00000163 | 0.580 | 0.957 | 35.849 | 168 |
| AverageAD | -0.00000006 | 0.00000016 | -0.00000038 | 0.00000026 | 0.717 | 0.957 | 11.735 | 168 |

Table S102. Beta’s from linear regression analyses examining the association between MD values and severity of symptoms at study inclusion measured by the BDI-II **in adolescents only**. Age, sex, agexsex, age^2^, age^2^xsex and scansite included as covariates.

| Region | Beta | SE | CI LB | CI UB | P-value | FDR P-value | I^2^ | N |
| --- | --- | --- | --- | --- | --- | --- | --- | --- |
| ACR | 0.00000000 | 0.00000019 | -0.00000037 | 0.00000037 | 0.981 | 0.981 | 0.000 | 168 |
| ALIC | -0.00000006 | 0.00000013 | -0.00000031 | 0.00000018 | 0.606 | 0.981 | 0.000 | 168 |
| BCC | 0.00000021 | 0.00000027 | -0.00000032 | 0.00000075 | 0.435 | 0.981 | 30.566 | 168 |
| CC | 0.00000013 | 0.00000023 | -0.00000032 | 0.00000058 | 0.565 | 0.981 | 36.144 | 168 |
| CGC | 0.00000013 | 0.00000019 | -0.00000025 | 0.00000051 | 0.511 | 0.981 | 10.610 | 168 |
| CGH | 0.00000017 | 0.00000024 | -0.00000029 | 0.00000064 | 0.462 | 0.981 | 23.288 | 168 |
| CR | 0.00000002 | 0.00000016 | -0.00000029 | 0.00000032 | 0.904 | 0.981 | 0.000 | 168 |
| CST | 0.00000005 | 0.00000047 | -0.00000087 | 0.00000096 | 0.922 | 0.981 | 44.319 | 168 |
| EC | 0.00000016 | 0.00000023 | -0.00000028 | 0.00000061 | 0.473 | 0.981 | 71.030 | 168 |
| FX | 0.00000059 | 0.00000122 | -0.00000180 | 0.00000298 | 0.627 | 0.981 | 18.248 | 168 |
| FXST | 0.00000016 | 0.00000016 | -0.00000015 | 0.00000047 | 0.320 | 0.981 | 0.000 | 168 |
| GCC | 0.00000012 | 0.00000045 | -0.00000076 | 0.00000100 | 0.785 | 0.981 | 79.333 | 168 |
| IC | -0.00000002 | 0.00000014 | -0.00000029 | 0.00000024 | 0.878 | 0.981 | 0.000 | 168 |
| IFO | -0.00000008 | 0.00000018 | -0.00000043 | 0.00000026 | 0.637 | 0.981 | 7.630 | 168 |
| PCR | 0.00000005 | 0.00000019 | -0.00000032 | 0.00000043 | 0.780 | 0.981 | 2.730 | 168 |
| PLIC | -0.00000010 | 0.00000016 | -0.00000042 | 0.00000022 | 0.542 | 0.981 | 0.000 | 168 |
| PTR | 0.00000014 | 0.00000020 | -0.00000025 | 0.00000053 | 0.476 | 0.981 | 0.000 | 168 |
| RLIC | 0.00000006 | 0.00000016 | -0.00000027 | 0.00000038 | 0.735 | 0.981 | 0.000 | 168 |
| SCC | -0.00000001 | 0.00000015 | -0.00000031 | 0.00000029 | 0.942 | 0.981 | 0.000 | 168 |
| SCR | 0.00000001 | 0.00000014 | -0.00000026 | 0.00000028 | 0.961 | 0.981 | 0.000 | 168 |
| SFO | 0.00000010 | 0.00000021 | -0.00000031 | 0.00000050 | 0.640 | 0.981 | 14.542 | 168 |
| SLF | 0.00000001 | 0.00000016 | -0.00000031 | 0.00000033 | 0.931 | 0.981 | 0.000 | 168 |
| SS | 0.00000007 | 0.00000021 | -0.00000034 | 0.00000048 | 0.746 | 0.981 | 7.715 | 168 |
| UNC | 0.00000028 | 0.00000029 | -0.00000030 | 0.00000085 | 0.343 | 0.981 | 6.500 | 168 |
| AverageMD | 0.00000006 | 0.00000012 | -0.00000017 | 0.00000030 | 0.588 | 0.981 | 0.000 | 168 |

Table S103. Beta’s from linear regression analyses examining the association between RD values and severity of symptoms at study inclusion measured by the BDI-II **in adolescents only**. Age, sex, agexsex, age^2^, age^2^xsex and scansite included as covariates.

| Region | Beta | SE | CI LB | CI UB | P-value | FDR P-value | I^2^ | N |
| --- | --- | --- | --- | --- | --- | --- | --- | --- |
| ACR | 0.00000015 | 0.00000020 | -0.00000025 | 0.00000055 | 0.450 | 0.635 | 3.863 | 168 |
| ALIC | 0.00000004 | 0.00000013 | -0.00000022 | 0.00000030 | 0.771 | 0.803 | 0.000 | 168 |
| BCC | 0.00000041 | 0.00000051 | -0.00000058 | 0.00000141 | 0.415 | 0.635 | 58.555 | 168 |
| CC | 0.00000031 | 0.00000030 | -0.00000028 | 0.00000090 | 0.305 | 0.635 | 43.135 | 168 |
| CGC | 0.00000016 | 0.00000023 | -0.00000029 | 0.00000061 | 0.488 | 0.635 | 1.594 | 168 |
| CGH | 0.00000013 | 0.00000028 | -0.00000042 | 0.00000069 | 0.638 | 0.693 | 20.962 | 168 |
| CR | 0.00000015 | 0.00000017 | -0.00000018 | 0.00000048 | 0.382 | 0.635 | 0.000 | 168 |
| CST | 0.00000023 | 0.00000033 | -0.00000042 | 0.00000088 | 0.483 | 0.635 | 16.047 | 168 |
| EC | 0.00000013 | 0.00000021 | -0.00000028 | 0.00000055 | 0.536 | 0.635 | 41.652 | 168 |
| FX | 0.00000065 | 0.00000109 | -0.00000148 | 0.00000278 | 0.552 | 0.635 | 17.011 | 168 |
| FXST | 0.00000029 | 0.00000020 | -0.00000010 | 0.00000069 | 0.146 | 0.635 | 6.972 | 168 |
| GCC | 0.00000036 | 0.00000047 | -0.00000056 | 0.00000129 | 0.440 | 0.635 | 79.160 | 168 |
| IC | 0.00000008 | 0.00000014 | -0.00000020 | 0.00000037 | 0.559 | 0.635 | 0.000 | 168 |
| IFO | -0.00000020 | 0.00000022 | -0.00000063 | 0.00000023 | 0.362 | 0.635 | 0.000 | 168 |
| PCR | 0.00000013 | 0.00000020 | -0.00000026 | 0.00000052 | 0.508 | 0.635 | 0.000 | 168 |
| PLIC | 0.00000004 | 0.00000018 | -0.00000031 | 0.00000040 | 0.812 | 0.812 | 0.000 | 168 |
| PTR | 0.00000022 | 0.00000019 | -0.00000015 | 0.00000059 | 0.247 | 0.635 | 0.000 | 168 |
| RLIC | 0.00000023 | 0.00000019 | -0.00000014 | 0.00000060 | 0.232 | 0.635 | 0.000 | 168 |
| SCC | 0.00000010 | 0.00000016 | -0.00000022 | 0.00000042 | 0.559 | 0.635 | 0.000 | 168 |
| SCR | 0.00000010 | 0.00000016 | -0.00000021 | 0.00000041 | 0.523 | 0.635 | 0.000 | 168 |
| SFO | 0.00000020 | 0.00000019 | -0.00000017 | 0.00000057 | 0.291 | 0.635 | 7.509 | 168 |
| SLF | 0.00000015 | 0.00000018 | -0.00000019 | 0.00000050 | 0.391 | 0.635 | 0.000 | 168 |
| SS | 0.00000019 | 0.00000022 | -0.00000023 | 0.00000062 | 0.365 | 0.635 | 0.000 | 168 |
| UNC | 0.00000044 | 0.00000030 | -0.00000014 | 0.00000102 | 0.139 | 0.635 | 0.000 | 168 |
| AverageRD | 0.00000014 | 0.00000012 | -0.00000011 | 0.00000038 | 0.267 | 0.635 | 4.902 | 168 |

Table S104. Beta’s from linear regression analyses examining the association between FA values and severity of symptoms at study inclusion measured by the HDRS **in adolescents only**. Age, sex, agexsex, age^2^, age^2^xsex and scansite included as covariates.

| Region | Beta | SE | CI LB | CI UB | P-value | FDR P-value | I^2^ | N |
| --- | --- | --- | --- | --- | --- | --- | --- | --- |
| ACR | 0.000141 | 0.000336 | -0.000516 | 0.000799 | 0.674 | 0.856 | 13.481 | 191 |
| ALIC | -0.000295 | 0.000470 | -0.001217 | 0.000627 | 0.531 | 0.856 | 45.251 | 191 |
| BCC | 0.000396 | 0.000277 | -0.000148 | 0.000940 | 0.153 | 0.771 | 0.000 | 191 |
| CC | 0.000194 | 0.000412 | -0.000613 | 0.001000 | 0.638 | 0.856 | 30.611 | 191 |
| CGC | 0.000632 | 0.000462 | -0.000274 | 0.001537 | 0.171 | 0.771 | 11.126 | 191 |
| CGH | 0.000751 | 0.000405 | -0.000043 | 0.001546 | 0.064 | 0.771 | 0.000 | 191 |
| CR | 0.000121 | 0.000334 | -0.000534 | 0.000776 | 0.717 | 0.856 | 24.165 | 191 |
| CST | -0.000439 | 0.001223 | -0.002836 | 0.001958 | 0.719 | 0.856 | 69.996 | 191 |
| EC | 0.000313 | 0.000225 | -0.000128 | 0.000754 | 0.164 | 0.771 | 0.000 | 191 |
| FX | 0.001218 | 0.000456 | 0.000325 | 0.002111 | 0.008 | 0.200 | 0.000 | 191 |
| FXST | 0.000251 | 0.000360 | -0.000455 | 0.000956 | 0.487 | 0.856 | 0.000 | 191 |
| GCC | -0.000163 | 0.000676 | -0.001487 | 0.001162 | 0.810 | 0.920 | 66.006 | 191 |
| IC | 0.000105 | 0.000282 | -0.000448 | 0.000658 | 0.711 | 0.856 | 20.618 | 191 |
| IFO | 0.000053 | 0.000433 | -0.000796 | 0.000901 | 0.903 | 0.972 | 0.000 | 191 |
| PCR | -0.000029 | 0.000398 | -0.000809 | 0.000750 | 0.941 | 0.972 | 19.700 | 191 |
| PLIC | 0.000258 | 0.000228 | -0.000188 | 0.000704 | 0.256 | 0.856 | 0.000 | 191 |
| PTR | 0.000209 | 0.000347 | -0.000470 | 0.000889 | 0.546 | 0.856 | 0.000 | 191 |
| RLIC | 0.000343 | 0.000258 | -0.000164 | 0.000849 | 0.185 | 0.771 | 0.000 | 191 |
| SCC | -0.000218 | 0.000536 | -0.001269 | 0.000833 | 0.684 | 0.856 | 66.529 | 191 |
| SCR | 0.000252 | 0.000307 | -0.000350 | 0.000854 | 0.412 | 0.856 | 11.615 | 191 |
| SFO | -0.000311 | 0.000364 | -0.001024 | 0.000402 | 0.393 | 0.856 | 15.033 | 191 |
| SLF | -0.000021 | 0.000587 | -0.001170 | 0.001129 | 0.972 | 0.972 | 62.395 | 191 |
| SS | 0.000111 | 0.000292 | -0.000463 | 0.000684 | 0.705 | 0.856 | 0.000 | 191 |
| UNC | -0.000251 | 0.000492 | -0.001216 | 0.000713 | 0.610 | 0.856 | 0.000 | 191 |
| AverageFA | 0.000143 | 0.000284 | -0.000414 | 0.000700 | 0.614 | 0.856 | 43.435 | 191 |

Table S105. Beta’s from linear regression analyses examining the association between AD values and severity of symptoms at study inclusion measured by the HDRS **in adolescents only**. Age, sex, agexsex, age^2^, age^2^xsex and scansite included as covariates.

| Region | Beta | SE | CI LB | CI UB | P-value | FDR P-value | I^2^ | N |
| --- | --- | --- | --- | --- | --- | --- | --- | --- |
| ACR | -0.00000091 | 0.00000061 | -0.00000212 | 0.00000029 | 0.137 | 0.893 | 0.000 | 191 |
| ALIC | -0.00000123 | 0.00000067 | -0.00000254 | 0.00000008 | 0.065 | 0.813 | 0.000 | 191 |
| BCC | -0.00000032 | 0.00000084 | -0.00000196 | 0.00000132 | 0.706 | 0.893 | 6.541 | 191 |
| CC | -0.00000021 | 0.00000070 | -0.00000157 | 0.00000115 | 0.764 | 0.893 | 0.000 | 191 |
| CGC | -0.00000068 | 0.00000161 | -0.00000383 | 0.00000247 | 0.673 | 0.893 | 71.619 | 191 |
| CGH | 0.00000140 | 0.00000142 | -0.00000138 | 0.00000418 | 0.323 | 0.893 | 0.000 | 191 |
| CR | -0.00000049 | 0.00000045 | -0.00000136 | 0.00000039 | 0.275 | 0.893 | 0.000 | 191 |
| CST | -0.00000025 | 0.00000120 | -0.00000261 | 0.00000211 | 0.833 | 0.905 | 0.000 | 191 |
| EC | 0.00000001 | 0.00000044 | -0.00000085 | 0.00000086 | 0.987 | 0.987 | 1.824 | 191 |
| FX | -0.00000091 | 0.00000186 | -0.00000455 | 0.00000274 | 0.625 | 0.893 | 0.000 | 191 |
| FXST | -0.00000032 | 0.00000071 | -0.00000172 | 0.00000107 | 0.649 | 0.893 | 6.020 | 191 |
| GCC | -0.00000161 | 0.00000068 | -0.00000294 | -0.00000028 | 0.018 | 0.450 | 0.000 | 191 |
| IC | -0.00000051 | 0.00000050 | -0.00000149 | 0.00000048 | 0.315 | 0.893 | 0.000 | 191 |
| IFO | 0.00000048 | 0.00000089 | -0.00000126 | 0.00000223 | 0.588 | 0.893 | 0.000 | 191 |
| PCR | 0.00000036 | 0.00000069 | -0.00000100 | 0.00000171 | 0.605 | 0.893 | 0.615 | 191 |
| PLIC | -0.00000147 | 0.00000125 | -0.00000392 | 0.00000097 | 0.237 | 0.893 | 80.428 | 191 |
| PTR | 0.00000028 | 0.00000096 | -0.00000160 | 0.00000217 | 0.768 | 0.893 | 0.000 | 191 |
| RLIC | 0.00000033 | 0.00000084 | -0.00000132 | 0.00000197 | 0.697 | 0.893 | 19.402 | 191 |
| SCC | 0.00000143 | 0.00000104 | -0.00000061 | 0.00000346 | 0.170 | 0.893 | 0.000 | 191 |
| SCR | -0.00000016 | 0.00000042 | -0.00000098 | 0.00000066 | 0.697 | 0.893 | 0.000 | 191 |
| SFO | -0.00000089 | 0.00000167 | -0.00000415 | 0.00000238 | 0.595 | 0.893 | 59.350 | 191 |
| SLF | 0.00000076 | 0.00000136 | -0.00000191 | 0.00000342 | 0.579 | 0.893 | 87.148 | 191 |
| SS | 0.00000015 | 0.00000118 | -0.00000215 | 0.00000246 | 0.897 | 0.934 | 48.959 | 191 |
| UNC | 0.00000202 | 0.00000215 | -0.00000220 | 0.00000623 | 0.348 | 0.893 | 42.709 | 191 |
| AverageAD | 0.00000009 | 0.00000034 | -0.00000058 | 0.00000076 | 0.786 | 0.893 | 0.000 | 191 |

Table S106. Beta’s from linear regression analyses examining the association between MD values and severity of symptoms at study inclusion measured by the HDRS **in adolescents only**. Age, sex, agexsex, age^2^, age^2^xsex and scansite included as covariates.

| Region | Beta | SE | CI LB | CI UB | P-value | FDR P-value | I^2^ | N |
| --- | --- | --- | --- | --- | --- | --- | --- | --- |
| ACR | -0.00000045 | 0.00000064 | -0.00000170 | 0.00000080 | 0.484 | 0.929 | 45.425 | 191 |
| ALIC | 0.00000007 | 0.00000047 | -0.00000084 | 0.00000098 | 0.877 | 0.929 | 22.957 | 191 |
| BCC | -0.00000029 | 0.00000094 | -0.00000214 | 0.00000156 | 0.761 | 0.929 | 29.682 | 191 |
| CC | 0.00000010 | 0.00000072 | -0.00000131 | 0.00000151 | 0.892 | 0.929 | 18.274 | 191 |
| CGC | 0.00000033 | 0.00000049 | -0.00000063 | 0.00000128 | 0.502 | 0.929 | 2.434 | 191 |
| CGH | 0.00000135 | 0.00000095 | -0.00000050 | 0.00000321 | 0.153 | 0.929 | 25.247 | 191 |
| CR | -0.00000021 | 0.00000052 | -0.00000122 | 0.00000080 | 0.686 | 0.929 | 42.656 | 191 |
| CST | -0.00000038 | 0.00000095 | -0.00000224 | 0.00000148 | 0.688 | 0.929 | 0.000 | 191 |
| EC | 0.00000034 | 0.00000066 | -0.00000095 | 0.00000163 | 0.601 | 0.929 | 75.999 | 191 |
| FX | -0.00000151 | 0.00000181 | -0.00000506 | 0.00000204 | 0.404 | 0.929 | 0.000 | 191 |
| FXST | 0.00000010 | 0.00000050 | -0.00000089 | 0.00000108 | 0.848 | 0.929 | 16.584 | 191 |
| GCC | -0.00000061 | 0.00000144 | -0.00000343 | 0.00000221 | 0.673 | 0.929 | 74.350 | 191 |
| IC | 0.00000012 | 0.00000035 | -0.00000057 | 0.00000081 | 0.737 | 0.929 | 8.472 | 191 |
| IFO | 0.00000157 | 0.00000085 | -0.00000010 | 0.00000324 | 0.066 | 0.929 | 27.536 | 191 |
| PCR | 0.00000037 | 0.00000055 | -0.00000071 | 0.00000146 | 0.498 | 0.929 | 2.311 | 191 |
| PLIC | -0.00000036 | 0.00000032 | -0.00000099 | 0.00000027 | 0.265 | 0.929 | 0.000 | 191 |
| PTR | 0.00000057 | 0.00000051 | -0.00000042 | 0.00000157 | 0.260 | 0.929 | 0.000 | 191 |
| RLIC | 0.00000048 | 0.00000050 | -0.00000050 | 0.00000146 | 0.339 | 0.929 | 13.766 | 191 |
| SCC | 0.00000076 | 0.00000074 | -0.00000070 | 0.00000221 | 0.308 | 0.929 | 8.338 | 191 |
| SCR | -0.00000007 | 0.00000041 | -0.00000088 | 0.00000073 | 0.857 | 0.929 | 41.117 | 191 |
| SFO | 0.00000010 | 0.00000121 | -0.00000227 | 0.00000247 | 0.935 | 0.935 | 67.846 | 191 |
| SLF | 0.00000011 | 0.00000044 | -0.00000076 | 0.00000097 | 0.809 | 0.929 | 39.842 | 191 |
| SS | 0.00000060 | 0.00000099 | -0.00000134 | 0.00000254 | 0.545 | 0.929 | 65.721 | 191 |
| UNC | 0.00000049 | 0.00000118 | -0.00000183 | 0.00000281 | 0.681 | 0.929 | 8.821 | 191 |
| AverageMD | 0.00000021 | 0.00000033 | -0.00000044 | 0.00000085 | 0.529 | 0.929 | 0.000 | 191 |

Table S107. Beta’s from linear regression analyses examining the association between RD values and severity of symptoms at study inclusion measured by the HDRS **in adolescents only**. Age, sex, agexsex, age^2^, age^2^xsex and scansite included as covariates.

| Region | Beta | SE | CI LB | CI UB | P-value | FDR P-value | I^2^ | N |
| --- | --- | --- | --- | --- | --- | --- | --- | --- |
| ACR | -0.00000034 | 0.00000069 | -0.00000169 | 0.00000100 | 0.617 | 0.976 | 48.880 | 191 |
| ALIC | 0.00000037 | 0.00000053 | -0.00000068 | 0.00000141 | 0.491 | 0.976 | 30.370 | 191 |
| BCC | -0.00000054 | 0.00000128 | -0.00000305 | 0.00000198 | 0.675 | 0.976 | 33.851 | 191 |
| CC | -0.00000012 | 0.00000100 | -0.00000207 | 0.00000183 | 0.902 | 0.976 | 34.815 | 191 |
| CGC | 0.00000039 | 0.00000061 | -0.00000081 | 0.00000158 | 0.526 | 0.976 | 0.000 | 191 |
| CGH | 0.00000006 | 0.00000084 | -0.00000159 | 0.00000170 | 0.947 | 0.976 | 0.000 | 191 |
| CR | -0.00000016 | 0.00000062 | -0.00000138 | 0.00000106 | 0.799 | 0.976 | 49.197 | 191 |
| CST | -0.00000015 | 0.00000113 | -0.00000237 | 0.00000206 | 0.891 | 0.976 | 15.670 | 191 |
| EC | 0.00000002 | 0.00000055 | -0.00000105 | 0.00000109 | 0.976 | 0.976 | 42.396 | 191 |
| FX | -0.00000177 | 0.00000187 | -0.00000543 | 0.00000190 | 0.345 | 0.976 | 0.000 | 191 |
| FXST | -0.00000019 | 0.00000061 | -0.00000140 | 0.00000101 | 0.753 | 0.976 | 7.860 | 191 |
| GCC | -0.00000024 | 0.00000182 | -0.00000380 | 0.00000332 | 0.896 | 0.976 | 77.434 | 191 |
| IC | 0.00000014 | 0.00000041 | -0.00000066 | 0.00000095 | 0.728 | 0.976 | 8.532 | 191 |
| IFO | 0.00000119 | 0.00000076 | -0.00000030 | 0.00000268 | 0.118 | 0.976 | 0.000 | 191 |
| PCR | 0.00000042 | 0.00000073 | -0.00000101 | 0.00000185 | 0.562 | 0.976 | 15.031 | 191 |
| PLIC | -0.00000032 | 0.00000038 | -0.00000107 | 0.00000042 | 0.399 | 0.976 | 0.000 | 191 |
| PTR | 0.00000055 | 0.00000061 | -0.00000065 | 0.00000174 | 0.368 | 0.976 | 0.000 | 191 |
| RLIC | 0.00000029 | 0.00000049 | -0.00000067 | 0.00000125 | 0.555 | 0.976 | 0.000 | 191 |
| SCC | 0.00000082 | 0.00000054 | -0.00000023 | 0.00000187 | 0.127 | 0.976 | 0.000 | 191 |
| SCR | -0.00000015 | 0.00000048 | -0.00000110 | 0.00000079 | 0.748 | 0.976 | 40.641 | 191 |
| SFO | 0.00000035 | 0.00000090 | -0.00000142 | 0.00000211 | 0.700 | 0.976 | 36.803 | 191 |
| SLF | 0.00000011 | 0.00000050 | -0.00000086 | 0.00000108 | 0.826 | 0.976 | 43.367 | 191 |
| SS | 0.00000039 | 0.00000082 | -0.00000121 | 0.00000199 | 0.636 | 0.976 | 34.606 | 191 |
| UNC | 0.00000091 | 0.00000107 | -0.00000119 | 0.00000302 | 0.395 | 0.976 | 0.000 | 191 |
| AverageRD | 0.00000020 | 0.00000037 | -0.00000053 | 0.00000093 | 0.590 | 0.976 | 0.000 | 191 |

**9. UK biobank**

Table S108 . Cohen’s d effect sizes for FA differences between patients and controls for UK biobank. Age, sex, agexsex, age^2^, age^2^xsex, included as covariates.

| Region | Cohen’s d | SE | CI LB | CI UB | P-value | FDR P-value | Controls | Patients |
| --- | --- | --- | --- | --- | --- | --- | --- | --- |
| ACR | -0.08 | 0.05 | -0.19 | 0.02 | 0.15 | 0.257 | 1060 | 540 |
| ALIC | -0.05 | 0.05 | -0.16 | 0.05 | 0.37 | 0.467 | 1060 | 540 |
| BCC | -0.04 | 0.05 | -0.14 | 0.07 | 0.51 | 0.583 | 1060 | 540 |
| CC | -0.08 | 0.05 | -0.19 | 0.02 | 0.15 | 0.257 | 1060 | 540 |
| CGC | -0.09 | 0.05 | -0.19 | 0.01 | 0.11 | 0.257 | 1060 | 540 |
| CGH | -0.01 | 0.05 | -0.12 | 0.09 | 0.83 | 0.830 | 1060 | 540 |
| CR | -0.08 | 0.05 | -0.19 | 0.02 | 0.15 | 0.257 | 1060 | 540 |
| CST | -0.02 | 0.05 | -0.13 | 0.08 | 0.68 | 0.742 | 1060 | 540 |
| EC | -0.06 | 0.05 | -0.16 | 0.04 | 0.31 | 0.438 | 1060 | 540 |
| FX | -0.13 | 0.05 | -0.23 | -0.02 | 0.03 | 0.180 | 1060 | 540 |
| FXST | -0.09 | 0.05 | -0.19 | 0.02 | 0.12 | 0.257 | 1060 | 540 |
| GCC | -0.11 | 0.05 | -0.21 | -0.00 | 0.07 | 0.240 | 1060 | 540 |
| IC | -0.04 | 0.05 | -0.15 | 0.06 | 0.46 | 0.552 | 1060 | 540 |
| IFO | -0.15 | 0.05 | -0.26 | -0.05 | 0.01 | 0.180 | 1060 | 540 |
| PCR | -0.05 | 0.05 | -0.16 | 0.05 | 0.35 | 0.467 | 1060 | 540 |
| PLIC | 0.02 | 0.05 | -0.08 | 0.12 | 0.73 | 0.762 | 1060 | 540 |
| PTR | -0.13 | 0.05 | -0.24 | -0.03 | 0.02 | 0.180 | 1060 | 540 |
| RLIC | -0.07 | 0.05 | -0.18 | 0.03 | 0.20 | 0.320 | 1060 | 540 |
| SCC | -0.10 | 0.05 | -0.20 | 0.00 | 0.08 | 0.240 | 1060 | 540 |
| SCR | -0.07 | 0.05 | -0.17 | 0.03 | 0.23 | 0.345 | 1060 | 540 |
| SFO | -0.11 | 0.05 | -0.21 | -0.01 | 0.06 | 0.240 | 1060 | 540 |
| SLF | -0.10 | 0.05 | -0.20 | 0.00 | 0.08 | 0.240 | 1060 | 540 |
| SS | -0.10 | 0.05 | -0.20 | 0.01 | 0.09 | 0.240 | 1060 | 540 |
| UNC | -0.13 | 0.05 | -0.23 | -0.02 | 0.03 | 0.180 | 1060 | 540 |

Table S109 . Cohen’s d effect sizes for AD differences between patients and controls for UK biobank. Age, sex, agexsex, age^2^, age^2^xsex, included as covariates.

| Region | Cohen’s d | SE | CI LB | CI UB | P-value | FDR P-value | Controls | Patients |
| --- | --- | --- | --- | --- | --- | --- | --- | --- |
| ACR | 0.11 | 0.05 | 0.00 | 0.21 | 0.06 | 0.288 | 1060 | 540 |
| ALIC | 0.13 | 0.05 | 0.03 | 0.24 | 0.02 | 0.240 | 1060 | 540 |
| BCC | -0.02 | 0.05 | -0.12 | 0.08 | 0.71 | 0.897 | 1060 | 540 |
| CC | -0.03 | 0.05 | -0.14 | 0.07 | 0.54 | 0.880 | 1060 | 540 |
| CGC | -0.03 | 0.05 | -0.13 | 0.08 | 0.63 | 0.880 | 1060 | 540 |
| CGH | 0.03 | 0.05 | -0.07 | 0.14 | 0.57 | 0.880 | 1060 | 540 |
| CR | 0.10 | 0.05 | -0.00 | 0.20 | 0.08 | 0.320 | 1060 | 540 |
| CST | -0.00 | 0.05 | -0.10 | 0.10 | 1.00 | 1.000 | 1060 | 540 |
| EC | 0.08 | 0.05 | -0.02 | 0.19 | 0.16 | 0.456 | 1060 | 540 |
| FX | 0.06 | 0.05 | -0.04 | 0.16 | 0.31 | 0.620 | 1060 | 540 |
| FXST | 0.03 | 0.05 | -0.08 | 0.13 | 0.63 | 0.880 | 1060 | 540 |
| GCC | 0.01 | 0.05 | -0.09 | 0.11 | 0.85 | 0.971 | 1060 | 540 |
| IC | 0.11 | 0.05 | 0.01 | 0.21 | 0.06 | 0.288 | 1060 | 540 |
| IFO | -0.00 | 0.05 | -0.11 | 0.10 | 0.97 | 1.000 | 1060 | 540 |
| PCR | 0.06 | 0.05 | -0.04 | 0.17 | 0.28 | 0.611 | 1060 | 540 |
| PLIC | 0.12 | 0.05 | 0.02 | 0.22 | 0.04 | 0.288 | 1060 | 540 |
| PTR | 0.05 | 0.05 | -0.05 | 0.16 | 0.36 | 0.665 | 1060 | 540 |
| RLIC | 0.02 | 0.05 | -0.09 | 0.12 | 0.78 | 0.936 | 1060 | 540 |
| SCC | -0.07 | 0.05 | -0.18 | 0.03 | 0.19 | 0.456 | 1060 | 540 |
| SCR | 0.08 | 0.05 | -0.02 | 0.19 | 0.15 | 0.456 | 1060 | 540 |
| SFO | 0.13 | 0.05 | 0.03 | 0.24 | 0.02 | 0.240 | 1060 | 540 |
| SLF | 0.08 | 0.05 | -0.03 | 0.18 | 0.18 | 0.456 | 1060 | 540 |
| SS | 0.03 | 0.05 | -0.08 | 0.13 | 0.66 | 0.880 | 1060 | 540 |
| UNC | 0.01 | 0.05 | -0.10 | 0.11 | 0.91 | 0.993 | 1060 | 540 |

Table S110 . Cohen’s d effect sizes for MD differences between patients and controls for UK biobank. Age, sex, agexsex, age^2^, age^2^xsex, included as covariates.

| Region | Cohen’s d | SE | CI LB | CI UB | P-value | FDR P-value | Controls | Patients |
| --- | --- | --- | --- | --- | --- | --- | --- | --- |
| ACR | 0.12 | 0.05 | 0.02 | 0.23 | 0.03 | 0.120 | 1060 | 540 |
| ALIC | 0.13 | 0.05 | 0.03 | 0.23 | 0.02 | 0.120 | 1060 | 540 |
| BCC | 0.01 | 0.05 | -0.09 | 0.11 | 0.88 | 0.929 | 1060 | 540 |
| CC | 0.03 | 0.05 | -0.07 | 0.14 | 0.55 | 0.629 | 1060 | 540 |
| CGC | 0.05 | 0.05 | -0.06 | 0.15 | 0.43 | 0.516 | 1060 | 540 |
| CGH | 0.06 | 0.05 | -0.05 | 0.16 | 0.33 | 0.417 | 1060 | 540 |
| CR | 0.11 | 0.05 | 0.01 | 0.22 | 0.05 | 0.133 | 1060 | 540 |
| CST | 0.00 | 0.05 | -0.10 | 0.10 | 0.99 | 0.990 | 1060 | 540 |
| EC | 0.09 | 0.05 | -0.02 | 0.19 | 0.13 | 0.260 | 1060 | 540 |
| FX | 0.10 | 0.05 | -0.00 | 0.20 | 0.08 | 0.175 | 1060 | 540 |
| FXST | 0.07 | 0.05 | -0.04 | 0.17 | 0.24 | 0.360 | 1060 | 540 |
| GCC | 0.08 | 0.05 | -0.02 | 0.19 | 0.15 | 0.277 | 1060 | 540 |
| IC | 0.12 | 0.05 | 0.01 | 0.22 | 0.04 | 0.120 | 1060 | 540 |
| IFO | 0.12 | 0.05 | 0.02 | 0.22 | 0.04 | 0.120 | 1060 | 540 |
| PCR | 0.06 | 0.05 | -0.04 | 0.17 | 0.27 | 0.373 | 1060 | 540 |
| PLIC | 0.11 | 0.05 | 0.00 | 0.21 | 0.06 | 0.144 | 1060 | 540 |
| PTR | 0.12 | 0.05 | 0.01 | 0.22 | 0.04 | 0.120 | 1060 | 540 |
| RLIC | 0.06 | 0.05 | -0.04 | 0.17 | 0.28 | 0.373 | 1060 | 540 |
| SCC | 0.01 | 0.05 | -0.10 | 0.11 | 0.89 | 0.929 | 1060 | 540 |
| SCR | 0.12 | 0.05 | 0.01 | 0.22 | 0.04 | 0.120 | 1060 | 540 |
| SFO | 0.16 | 0.05 | 0.05 | 0.26 | 0.01 | 0.120 | 1060 | 540 |
| SLF | 0.12 | 0.05 | 0.02 | 0.22 | 0.04 | 0.120 | 1060 | 540 |
| SS | 0.08 | 0.05 | -0.03 | 0.18 | 0.19 | 0.326 | 1060 | 540 |
| UNC | 0.07 | 0.05 | -0.03 | 0.17 | 0.23 | 0.360 | 1060 | 540 |

Table S111. Cohen’s d effect sizes for RD differences between patients and controls for UK biobank. Age, sex, agexsex, age^2^, age^2^xsex, included as covariates.

| Region | Cohen’s d | SE | CI LB | CI UB | P-value | FDR P-value | Controls | Patients |
| --- | --- | --- | --- | --- | --- | --- | --- | --- |
| ACR | 0.12 | 0.05 | 0.01 | 0.22 | 0.04 | 0.150 | 1060 | 540 |
| ALIC | 0.10 | 0.05 | -0.00 | 0.20 | 0.09 | 0.196 | 1060 | 540 |
| BCC | 0.02 | 0.05 | -0.08 | 0.13 | 0.67 | 0.710 | 1060 | 540 |
| CC | 0.07 | 0.05 | -0.03 | 0.17 | 0.23 | 0.291 | 1060 | 540 |
| CGC | 0.08 | 0.05 | -0.02 | 0.19 | 0.15 | 0.240 | 1060 | 540 |
| CGH | 0.05 | 0.05 | -0.05 | 0.16 | 0.36 | 0.411 | 1060 | 540 |
| CR | 0.11 | 0.05 | 0.01 | 0.21 | 0.05 | 0.150 | 1060 | 540 |
| CST | 0.00 | 0.05 | -0.10 | 0.11 | 0.98 | 0.980 | 1060 | 540 |
| EC | 0.08 | 0.05 | -0.02 | 0.18 | 0.17 | 0.240 | 1060 | 540 |
| FX | 0.11 | 0.05 | 0.01 | 0.22 | 0.05 | 0.150 | 1060 | 540 |
| FXST | 0.08 | 0.05 | -0.03 | 0.18 | 0.18 | 0.240 | 1060 | 540 |
| GCC | 0.10 | 0.05 | 0.00 | 0.21 | 0.07 | 0.187 | 1060 | 540 |
| IC | 0.08 | 0.05 | -0.02 | 0.19 | 0.15 | 0.240 | 1060 | 540 |
| IFO | 0.16 | 0.05 | 0.06 | 0.27 | 0.00 | 0.000 | 1060 | 540 |
| PCR | 0.06 | 0.05 | -0.04 | 0.16 | 0.30 | 0.360 | 1060 | 540 |
| PLIC | 0.02 | 0.05 | -0.08 | 0.13 | 0.68 | 0.710 | 1060 | 540 |
| PTR | 0.13 | 0.05 | 0.03 | 0.24 | 0.02 | 0.150 | 1060 | 540 |
| RLIC | 0.08 | 0.05 | -0.03 | 0.18 | 0.18 | 0.240 | 1060 | 540 |
| SCC | 0.08 | 0.05 | -0.03 | 0.18 | 0.17 | 0.240 | 1060 | 540 |
| SCR | 0.12 | 0.05 | 0.02 | 0.22 | 0.04 | 0.150 | 1060 | 540 |
| SFO | 0.15 | 0.05 | 0.05 | 0.26 | 0.01 | 0.120 | 1060 | 540 |
| SLF | 0.12 | 0.05 | 0.02 | 0.22 | 0.04 | 0.150 | 1060 | 540 |
| SS | 0.09 | 0.05 | -0.01 | 0.19 | 0.12 | 0.240 | 1060 | 540 |
| UNC | 0.10 | 0.05 | -0.01 | 0.20 | 0.09 | 0.196 | 1060 | 540 |

**10. Effect of MDD diagnosis: alternative FDR-correction**

Table S112. Cohen’s d effect sizes after meta-analysis, for FA differences between patients and controls **in adults only**. Results are FDR-corrected for the total number of tests (4 metrics x 25 ROIs). Age, sex, agexsex, age^2^, age^2^xsex and scansite included as covariates.

| Region | Cohen’s d | SE | CI LB | CI UB | P-value | FDR P-value | I^2^ | Controls | Patients |
| --- | --- | --- | --- | --- | --- | --- | --- | --- | --- |
| ACR | -0.253 | 0.079 | -0.408 | -0.098 | 0.001 | 0.020 | 54.984 | 1265 | 921 |
| ALIC | -0.232 | 0.080 | -0.388 | -0.076 | 0.004 | 0.025 | 55.406 | 1265 | 921 |
| BCC | -0.243 | 0.080 | -0.401 | -0.086 | 0.002 | 0.022 | 56.195 | 1265 | 921 |
| CC | -0.249 | 0.080 | -0.406 | -0.092 | 0.002 | 0.022 | 55.820 | 1265 | 921 |
| CGC | -0.165 | 0.053 | -0.268 | -0.062 | 0.002 | 0.022 | 11.516 | 1265 | 921 |
| CGH | -0.068 | 0.046 | -0.157 | 0.022 | 0.138 | 0.234 | 0.000 | 1265 | 921 |
| CR | -0.250 | 0.080 | -0.407 | -0.093 | 0.002 | 0.022 | 55.811 | 1265 | 921 |
| CST | -0.101 | 0.062 | -0.221 | 0.020 | 0.101 | 0.208 | 28.658 | 1265 | 921 |
| EC | -0.160 | 0.067 | -0.291 | -0.029 | 0.016 | 0.070 | 37.605 | 1265 | 921 |
| FX | -0.083 | 0.049 | -0.179 | 0.012 | 0.087 | 0.193 | 5.409 | 1265 | 921 |
| FXST | -0.183 | 0.062 | -0.305 | -0.061 | 0.003 | 0.025 | 29.984 | 1265 | 921 |
| GCC | -0.247 | 0.077 | -0.398 | -0.095 | 0.001 | 0.020 | 52.666 | 1265 | 921 |
| IC | -0.229 | 0.085 | -0.395 | -0.063 | 0.007 | 0.039 | 60.650 | 1265 | 921 |
| IFO | -0.121 | 0.048 | -0.214 | -0.027 | 0.012 | 0.055 | 3.601 | 1265 | 921 |
| PCR | -0.202 | 0.071 | -0.341 | -0.063 | 0.004 | 0.025 | 44.138 | 1265 | 921 |
| PLIC | -0.154 | 0.089 | -0.329 | 0.021 | 0.084 | 0.191 | 64.810 | 1265 | 921 |
| PTR | -0.135 | 0.086 | -0.303 | 0.033 | 0.116 | 0.215 | 61.851 | 1265 | 921 |
| RLIC | -0.151 | 0.079 | -0.305 | 0.003 | 0.054 | 0.142 | 54.283 | 1265 | 921 |
| SCC | -0.130 | 0.063 | -0.254 | -0.006 | 0.039 | 0.118 | 31.342 | 1265 | 921 |
| SCR | -0.197 | 0.086 | -0.366 | -0.029 | 0.022 | 0.081 | 61.930 | 1265 | 921 |
| SFO | -0.232 | 0.064 | -0.357 | -0.107 | 0.000 | 0.000 | 32.299 | 1265 | 921 |
| SLF | -0.169 | 0.081 | -0.327 | -0.011 | 0.037 | 0.116 | 56.865 | 1265 | 921 |
| SS | -0.230 | 0.080 | -0.387 | -0.073 | 0.004 | 0.025 | 56.069 | 1265 | 921 |
| UNC | -0.120 | 0.088 | -0.291 | 0.052 | 0.172 | 0.265 | 63.560 | 1265 | 921 |
| AverageFA | -0.258 | 0.073 | -0.403 | -0.114 | 0.000 | 0.000 | 47.560 | 1265 | 921 |

Table S113. Cohen’s d effect sizes after meta-analysis, for AD differences between patients and controls **in adults only**. Results are FDR-corrected for the total number of tests (4 metrics x 25 ROIs). Age, sex, agexsex, age^2^, age^2^xsex and scansite included as covariates.

| Region | Cohen’s d | SE | CI LB | CI UB | P-value | FDR P-value | I^2^ | Controls | Patients |
| --- | --- | --- | --- | --- | --- | --- | --- | --- | --- |
| ACR | -0.002 | 0.100 | -0.198 | 0.194 | 0.984 | 0.984 | 72.857 | 1265 | 921 |
| ALIC | 0.083 | 0.101 | -0.115 | 0.281 | 0.411 | 0.511 | 73.242 | 1265 | 921 |
| BCC | 0.101 | 0.099 | -0.093 | 0.296 | 0.306 | 0.414 | 72.078 | 1265 | 921 |
| CC | 0.095 | 0.104 | -0.110 | 0.299 | 0.366 | 0.463 | 75.131 | 1265 | 921 |
| CGC | -0.027 | 0.094 | -0.211 | 0.158 | 0.778 | 0.819 | 68.886 | 1265 | 921 |
| CGH | 0.069 | 0.088 | -0.104 | 0.241 | 0.433 | 0.522 | 63.992 | 1265 | 921 |
| CR | 0.053 | 0.093 | -0.130 | 0.236 | 0.572 | 0.615 | 68.391 | 1265 | 921 |
| CST | 0.102 | 0.087 | -0.068 | 0.272 | 0.240 | 0.343 | 62.841 | 1265 | 921 |
| EC | 0.089 | 0.097 | -0.101 | 0.278 | 0.358 | 0.463 | 70.597 | 1265 | 921 |
| FX | 0.141 | 0.086 | -0.028 | 0.309 | 0.102 | 0.208 | 61.952 | 1265 | 921 |
| FXST | 0.065 | 0.089 | -0.110 | 0.240 | 0.466 | 0.537 | 65.066 | 1265 | 921 |
| GCC | 0.038 | 0.101 | -0.159 | 0.235 | 0.706 | 0.751 | 73.030 | 1265 | 921 |
| IC | 0.095 | 0.096 | -0.093 | 0.284 | 0.321 | 0.428 | 70.213 | 1265 | 921 |
| IFO | 0.058 | 0.096 | -0.130 | 0.245 | 0.545 | 0.612 | 69.753 | 1265 | 921 |
| PCR | 0.076 | 0.095 | -0.109 | 0.262 | 0.421 | 0.513 | 69.284 | 1265 | 921 |
| PLIC | 0.174 | 0.087 | 0.004 | 0.344 | 0.045 | 0.132 | 62.797 | 1265 | 921 |
| PTR | 0.127 | 0.092 | -0.053 | 0.308 | 0.167 | 0.265 | 67.504 | 1265 | 921 |
| RLIC | 0.073 | 0.101 | -0.124 | 0.271 | 0.467 | 0.537 | 73.152 | 1265 | 921 |
| SCC | 0.125 | 0.097 | -0.066 | 0.315 | 0.200 | 0.294 | 70.877 | 1265 | 921 |
| SCR | 0.139 | 0.085 | -0.029 | 0.306 | 0.104 | 0.208 | 61.516 | 1265 | 921 |
| SFO | 0.067 | 0.105 | -0.140 | 0.273 | 0.526 | 0.598 | 75.530 | 1265 | 921 |
| SLF | 0.102 | 0.093 | -0.080 | 0.284 | 0.272 | 0.383 | 67.946 | 1265 | 921 |
| SS | 0.051 | 0.090 | -0.126 | 0.228 | 0.572 | 0.615 | 65.983 | 1265 | 921 |
| UNC | 0.066 | 0.114 | -0.158 | 0.290 | 0.564 | 0.615 | 79.609 | 1265 | 921 |
| AverageAD | 0.123 | 0.086 | -0.046 | 0.293 | 0.154 | 0.252 | 62.693 | 1265 | 921 |

Table S114. Cohen’s d effect sizes after meta-analysis, for MD differences between patients and controls **in adults only**. Results are FDR-corrected for the total number of tests (4 metrics x 25 ROIs). Age, sex, agexsex, age^2^, age^2^xsex and scansite included as covariates.

| Region | Cohen’s d | SE | CI LB | CI UB | P-value | FDR P-value | I^2^ | Controls | Patients |
| --- | --- | --- | --- | --- | --- | --- | --- | --- | --- |
| ACR | 0.065 | 0.088 | -0.107 | 0.236 | 0.459 | 0.537 | 63.620 | 1265 | 921 |
| ALIC | 0.124 | 0.100 | -0.073 | 0.320 | 0.217 | 0.314 | 72.639 | 1265 | 921 |
| BCC | 0.192 | 0.092 | 0.012 | 0.371 | 0.036 | 0.116 | 66.786 | 1265 | 921 |
| CC | 0.170 | 0.091 | -0.009 | 0.349 | 0.062 | 0.155 | 66.547 | 1265 | 921 |
| CGC | 0.085 | 0.083 | -0.078 | 0.248 | 0.306 | 0.414 | 59.337 | 1265 | 921 |
| CGH | 0.140 | 0.073 | -0.002 | 0.283 | 0.054 | 0.142 | 47.001 | 1265 | 921 |
| CR | 0.120 | 0.078 | -0.032 | 0.272 | 0.122 | 0.222 | 53.301 | 1265 | 921 |
| CST | 0.104 | 0.073 | -0.039 | 0.246 | 0.154 | 0.252 | 46.829 | 1265 | 921 |
| EC | 0.148 | 0.084 | -0.017 | 0.313 | 0.078 | 0.186 | 60.384 | 1265 | 921 |
| FX | 0.123 | 0.078 | -0.031 | 0.277 | 0.116 | 0.215 | 54.034 | 1265 | 921 |
| FXST | 0.180 | 0.075 | 0.033 | 0.327 | 0.017 | 0.071 | 50.051 | 1265 | 921 |
| GCC | 0.102 | 0.077 | -0.049 | 0.253 | 0.184 | 0.279 | 52.506 | 1265 | 921 |
| IC | 0.104 | 0.079 | -0.051 | 0.258 | 0.189 | 0.282 | 54.668 | 1265 | 921 |
| IFO | 0.093 | 0.058 | -0.020 | 0.206 | 0.106 | 0.208 | 21.769 | 1265 | 921 |
| PCR | 0.143 | 0.067 | 0.012 | 0.275 | 0.033 | 0.114 | 38.082 | 1265 | 921 |
| PLIC | 0.105 | 0.076 | -0.044 | 0.255 | 0.166 | 0.265 | 51.329 | 1265 | 921 |
| PTR | 0.111 | 0.071 | -0.027 | 0.250 | 0.115 | 0.215 | 43.877 | 1265 | 921 |
| RLIC | 0.075 | 0.073 | -0.069 | 0.218 | 0.306 | 0.414 | 47.523 | 1265 | 921 |
| SCC | 0.143 | 0.082 | -0.018 | 0.304 | 0.083 | 0.191 | 58.472 | 1265 | 921 |
| SCR | 0.192 | 0.067 | 0.062 | 0.323 | 0.004 | 0.025 | 37.170 | 1265 | 921 |
| SFO | 0.164 | 0.083 | 0.001 | 0.328 | 0.048 | 0.137 | 59.449 | 1265 | 921 |
| SLF | 0.126 | 0.076 | -0.023 | 0.276 | 0.098 | 0.208 | 51.613 | 1265 | 921 |
| SS | 0.120 | 0.064 | -0.005 | 0.246 | 0.061 | 0.155 | 33.189 | 1265 | 921 |
| UNC | 0.052 | 0.089 | -0.123 | 0.226 | 0.563 | 0.615 | 64.948 | 1265 | 921 |
| AverageMD | 0.176 | 0.075 | 0.029 | 0.324 | 0.019 | 0.076 | 50.183 | 1265 | 921 |

Table S115. Cohen’s d effect sizes after meta-analysis, for RD differences between patients and controls **in adults only**. Results are FDR-corrected for the total number of tests (4 metrics x 25 ROIs). Age, sex, agexsex, age^2^, age^2^xsex and scansite included as covariates.

| Region | Cohen’s d | SE | CI LB | CI UB | P-value | FDR P-value | I^2^ | Controls | Patients |
| --- | --- | --- | --- | --- | --- | --- | --- | --- | --- |
| ACR | 0.092 | 0.068 | -0.040 | 0.225 | 0.172 | 0.265 | 0.172 | 1265 | 921 |
| ALIC | 0.128 | 0.061 | 0.008 | 0.249 | 0.036 | 0.116 | 0.036 | 1265 | 921 |
| BCC | 0.174 | 0.067 | 0.043 | 0.304 | 0.009 | 0.043 | 0.009 | 1265 | 921 |
| CC | 0.095 | 0.062 | -0.027 | 0.216 | 0.127 | 0.223 | 0.127 | 1265 | 921 |
| CGC | 0.139 | 0.071 | -0.001 | 0.279 | 0.051 | 0.142 | 0.051 | 1265 | 921 |
| CGH | 0.154 | 0.059 | 0.038 | 0.271 | 0.009 | 0.043 | 0.009 | 1265 | 921 |
| CR | 0.122 | 0.046 | 0.032 | 0.211 | 0.008 | 0.042 | 0.008 | 1265 | 921 |
| CST | 0.091 | 0.061 | -0.028 | 0.210 | 0.136 | 0.234 | 0.136 | 1265 | 921 |
| EC | 0.158 | 0.071 | 0.019 | 0.297 | 0.025 | 0.089 | 0.025 | 1265 | 921 |
| FX | 0.118 | 0.073 | -0.025 | 0.260 | 0.106 | 0.208 | 0.106 | 1265 | 921 |
| FXST | 0.176 | 0.061 | 0.058 | 0.295 | 0.004 | 0.025 | 0.004 | 1265 | 921 |
| GCC | 0.060 | 0.078 | -0.094 | 0.213 | 0.445 | 0.530 | 0.445 | 1265 | 921 |
| IC | 0.057 | 0.063 | -0.066 | 0.179 | 0.366 | 0.463 | 0.366 | 1265 | 921 |
| IFO | 0.062 | 0.066 | -0.067 | 0.191 | 0.345 | 0.454 | 0.345 | 1265 | 921 |
| PCR | 0.096 | 0.063 | -0.027 | 0.220 | 0.127 | 0.223 | 0.127 | 1265 | 921 |
| PLIC | 0.014 | 0.085 | -0.153 | 0.181 | 0.873 | 0.891 | 0.873 | 1265 | 921 |
| PTR | 0.026 | 0.098 | -0.166 | 0.217 | 0.793 | 0.826 | 0.793 | 1265 | 921 |
| RLIC | 0.007 | 0.055 | -0.101 | 0.114 | 0.905 | 0.914 | 0.905 | 1265 | 921 |
| SCC | 0.083 | 0.101 | -0.116 | 0.281 | 0.414 | 0.511 | 0.414 | 1265 | 921 |
| SCR | 0.158 | 0.046 | 0.068 | 0.247 | 0.001 | 0.020 | 0.001 | 1265 | 921 |
| SFO | 0.129 | 0.046 | 0.039 | 0.218 | 0.005 | 0.029 | 0.005 | 1265 | 921 |
| SLF | 0.082 | 0.046 | -0.007 | 0.172 | 0.072 | 0.176 | 0.072 | 1265 | 921 |
| SS | 0.106 | 0.046 | 0.016 | 0.195 | 0.020 | 0.077 | 0.020 | 1265 | 921 |
| UNC | -0.013 | 0.081 | -0.172 | 0.145 | 0.868 | 0.891 | 0.868 | 1265 | 921 |
| AverageRD | 0.152 | 0.051 | 0.052 | 0.253 | 0.003 | 0.025 | 0.003 | 1265 | 921 |

Table S116. Cohen’s d effect sizes after meta-analysis, for FA differences between patients and controls **in adolescents only**. Results are FDR-corrected for the total number of tests (4 metrics x 25 ROIs). Age, sex, agexsex, age^2^, age^2^xsex and scansite included as covariates.

| Region | Cohen’s d | SE | CI LB | CI UB | P-value | FDR P-value | I^2^ | Controls | Patients |
| --- | --- | --- | --- | --- | --- | --- | --- | --- | --- |
| ACR | -0.040 | 0.141 | -0.316 | 0.235 | 0.773 | 0.889 | 55.139 | 290 | 372 |
| ALIC | 0.064 | 0.160 | -0.250 | 0.377 | 0.691 | 0.880 | 65.133 | 290 | 372 |
| BCC | -0.220 | 0.090 | -0.397 | -0.043 | 0.015 | 0.427 | 0.000 | 290 | 372 |
| CC | -0.195 | 0.090 | -0.372 | -0.018 | 0.031 | 0.427 | 0.000 | 290 | 372 |
| CGC | 0.068 | 0.143 | -0.211 | 0.347 | 0.634 | 0.880 | 56.353 | 290 | 372 |
| CGH | 0.098 | 0.135 | -0.165 | 0.362 | 0.465 | 0.775 | 51.107 | 290 | 372 |
| CR | -0.046 | 0.127 | -0.296 | 0.203 | 0.716 | 0.880 | 45.724 | 290 | 372 |
| CST | -0.051 | 0.110 | -0.266 | 0.165 | 0.645 | 0.880 | 28.635 | 290 | 372 |
| EC | 0.254 | 0.157 | -0.053 | 0.561 | 0.104 | 0.530 | 63.436 | 290 | 372 |
| FX | 0.019 | 0.098 | -0.172 | 0.210 | 0.847 | 0.918 | 11.427 | 290 | 372 |
| FXST | 0.041 | 0.134 | -0.221 | 0.303 | 0.760 | 0.889 | 50.207 | 290 | 372 |
| GCC | -0.170 | 0.113 | -0.392 | 0.053 | 0.134 | 0.569 | 32.256 | 290 | 372 |
| IC | 0.126 | 0.113 | -0.097 | 0.348 | 0.268 | 0.632 | 32.091 | 290 | 372 |
| IFO | -0.041 | 0.147 | -0.328 | 0.247 | 0.782 | 0.889 | 58.632 | 290 | 372 |
| PCR | -0.029 | 0.090 | -0.206 | 0.148 | 0.750 | 0.889 | 0.000 | 290 | 372 |
| PLIC | 0.156 | 0.090 | -0.021 | 0.333 | 0.084 | 0.500 | 0.000 | 290 | 372 |
| PTR | 0.048 | 0.102 | -0.152 | 0.248 | 0.636 | 0.880 | 17.924 | 290 | 372 |
| RLIC | 0.072 | 0.090 | -0.105 | 0.249 | 0.426 | 0.775 | 0.000 | 290 | 372 |
| SCC | -0.048 | 0.090 | -0.225 | 0.128 | 0.592 | 0.870 | 0.000 | 290 | 372 |
| SCR | -0.059 | 0.118 | -0.289 | 0.172 | 0.617 | 0.880 | 37.020 | 290 | 372 |
| SFO | -0.022 | 0.117 | -0.251 | 0.208 | 0.854 | 0.918 | 36.151 | 290 | 372 |
| SLF | 0.015 | 0.103 | -0.188 | 0.218 | 0.885 | 0.918 | 20.215 | 290 | 372 |
| SS | 0.035 | 0.117 | -0.194 | 0.264 | 0.767 | 0.889 | 36.024 | 290 | 372 |
| UNC | 0.065 | 0.090 | -0.112 | 0.242 | 0.473 | 0.775 | 0.000 | 290 | 372 |
| AverageFA | -0.020 | 0.135 | -0.284 | 0.244 | 0.882 | 0.918 | 51.141 | 290 | 372 |

Table S117. Cohen’s d effect sizes after meta-analysis, for AD differences between patients and controls **in adolescents only**. Results are FDR-corrected for the total number of tests (4 metrics x 25 ROIs). Age, sex, agexsex, age^2^, age^2^xsex and scansite included as covariates.

| Region | Cohen’s d | SE | CI LB | CI UB | P-value | FDR P-value | I^2^ | Controls | Patients |
| --- | --- | --- | --- | --- | --- | --- | --- | --- | --- |
| ACR | 0.085 | 0.090 | -0.091 | 0.262 | 0.343 | 0.686 | 0.000 | 290 | 372 |
| ALIC | 0.162 | 0.091 | -0.015 | 0.340 | 0.073 | 0.500 | 0.007 | 290 | 372 |
| BCC | -0.038 | 0.090 | -0.215 | 0.139 | 0.672 | 0.880 | 0.002 | 290 | 372 |
| CC | -0.004 | 0.090 | -0.181 | 0.173 | 0.967 | 0.977 | 0.007 | 290 | 372 |
| CGC | 0.205 | 0.127 | -0.043 | 0.454 | 0.106 | 0.530 | 44.994 | 290 | 372 |
| CGH | 0.035 | 0.099 | -0.158 | 0.229 | 0.722 | 0.880 | 13.828 | 290 | 372 |
| CR | 0.093 | 0.093 | -0.088 | 0.274 | 0.315 | 0.669 | 3.681 | 290 | 372 |
| CST | -0.104 | 0.115 | -0.330 | 0.122 | 0.368 | 0.722 | 34.420 | 290 | 372 |
| EC | 0.189 | 0.110 | -0.026 | 0.404 | 0.085 | 0.500 | 27.886 | 290 | 372 |
| FX | 0.092 | 0.122 | -0.148 | 0.332 | 0.453 | 0.775 | 40.931 | 290 | 372 |
| FXST | 0.180 | 0.090 | 0.003 | 0.358 | 0.046 | 0.427 | 0.000 | 290 | 372 |
| GCC | 0.098 | 0.090 | -0.079 | 0.275 | 0.278 | 0.632 | 0.000 | 290 | 372 |
| IC | 0.129 | 0.097 | -0.061 | 0.318 | 0.183 | 0.579 | 9.860 | 290 | 372 |
| IFO | 0.099 | 0.090 | -0.078 | 0.276 | 0.273 | 0.632 | 0.000 | 290 | 372 |
| PCR | 0.094 | 0.144 | -0.189 | 0.377 | 0.515 | 0.805 | 57.539 | 290 | 372 |
| PLIC | 0.006 | 0.107 | -0.204 | 0.215 | 0.957 | 0.977 | 24.671 | 290 | 372 |
| PTR | 0.132 | 0.153 | -0.168 | 0.432 | 0.390 | 0.750 | 61.882 | 290 | 372 |
| RLIC | 0.156 | 0.090 | -0.021 | 0.333 | 0.084 | 0.500 | 0.008 | 290 | 372 |
| SCC | -0.013 | 0.090 | -0.189 | 0.164 | 0.890 | 0.918 | 0.006 | 290 | 372 |
| SCR | 0.000 | 0.105 | -0.206 | 0.206 | 1.000 | 1.000 | 22.566 | 290 | 372 |
| SFO | 0.116 | 0.090 | -0.061 | 0.294 | 0.197 | 0.579 | 0.007 | 290 | 372 |
| SLF | 0.140 | 0.122 | -0.099 | 0.379 | 0.251 | 0.632 | 40.776 | 290 | 372 |
| SS | 0.125 | 0.090 | -0.052 | 0.302 | 0.165 | 0.569 | 0.000 | 290 | 372 |
| UNC | 0.134 | 0.136 | -0.133 | 0.400 | 0.326 | 0.669 | 51.848 | 290 | 372 |
| AverageAD | 0.126 | 0.090 | -0.052 | 0.303 | 0.165 | 0.569 | 0.005 | 290 | 372 |

Table S118. Cohen’s d effect sizes after meta-analysis, for MD differences between patients and controls **in adolescents only**. Results are FDR-corrected for the total number of tests (4 metrics x 25 ROIs). Age, sex, agexsex, age^2^, age^2^xsex and scansite included as covariates.

| Region | Cohen’s d | SE | CI LB | CI UB | P-value | FDR P-value | I^2^ | Controls | Patients |
| --- | --- | --- | --- | --- | --- | --- | --- | --- | --- |
| ACR | 0.116 | 0.101 | -0.082 | 0.315 | 0.251 | 0.632 | 17.086 | 290 | 372 |
| ALIC | 0.149 | 0.090 | -0.028 | 0.326 | 0.099 | 0.530 | 0.003 | 290 | 372 |
| BCC | 0.179 | 0.090 | 0.003 | 0.356 | 0.047 | 0.427 | 0.000 | 290 | 372 |
| CC | 0.183 | 0.090 | 0.006 | 0.360 | 0.043 | 0.427 | 0.000 | 290 | 372 |
| CGC | 0.188 | 0.090 | 0.011 | 0.365 | 0.038 | 0.427 | 0.000 | 290 | 372 |
| CGH | -0.034 | 0.143 | -0.314 | 0.246 | 0.811 | 0.901 | 56.466 | 290 | 372 |
| CR | 0.145 | 0.098 | -0.048 | 0.338 | 0.140 | 0.569 | 12.856 | 290 | 372 |
| CST | -0.042 | 0.112 | -0.262 | 0.177 | 0.705 | 0.880 | 31.227 | 290 | 372 |
| EC | 0.048 | 0.129 | -0.204 | 0.301 | 0.707 | 0.880 | 46.763 | 290 | 372 |
| FX | 0.146 | 0.108 | -0.065 | 0.358 | 0.175 | 0.579 | 25.630 | 290 | 372 |
| FXST | 0.150 | 0.107 | -0.059 | 0.359 | 0.160 | 0.569 | 24.180 | 290 | 372 |
| GCC | 0.227 | 0.090 | 0.049 | 0.404 | 0.012 | 0.427 | 0.000 | 290 | 372 |
| IC | 0.092 | 0.090 | -0.085 | 0.269 | 0.309 | 0.669 | 0.000 | 290 | 372 |
| IFO | 0.215 | 0.107 | 0.005 | 0.425 | 0.044 | 0.427 | 24.527 | 290 | 372 |
| PCR | 0.168 | 0.097 | -0.023 | 0.359 | 0.084 | 0.500 | 11.216 | 290 | 372 |
| PLIC | -0.062 | 0.090 | -0.239 | 0.115 | 0.492 | 0.794 | 0.000 | 290 | 372 |
| PTR | 0.157 | 0.121 | -0.080 | 0.395 | 0.194 | 0.579 | 40.113 | 290 | 372 |
| RLIC | 0.145 | 0.098 | -0.048 | 0.337 | 0.141 | 0.569 | 12.613 | 290 | 372 |
| SCC | 0.088 | 0.090 | -0.089 | 0.265 | 0.328 | 0.669 | 0.000 | 290 | 372 |
| SCR | 0.114 | 0.101 | -0.083 | 0.311 | 0.258 | 0.632 | 16.273 | 290 | 372 |
| SFO | 0.136 | 0.097 | -0.054 | 0.326 | 0.161 | 0.569 | 10.933 | 290 | 372 |
| SLF | 0.180 | 0.096 | -0.008 | 0.367 | 0.061 | 0.500 | 8.582 | 290 | 372 |
| SS | 0.135 | 0.106 | -0.073 | 0.343 | 0.204 | 0.583 | 23.629 | 290 | 372 |
| UNC | 0.152 | 0.106 | -0.057 | 0.360 | 0.154 | 0.569 | 24.077 | 290 | 372 |
| AverageMD | 0.156 | 0.107 | -0.053 | 0.365 | 0.144 | 0.569 | 24.469 | 290 | 372 |

Table S119. Cohen’s d effect sizes after meta-analysis, for RD differences between patients and controls **in adolescents only**. Results are FDR-corrected for the total number of tests (4 metrics x 25 ROIs). Age, sex, agexsex, age^2^, age^2^xsex and scansite included as covariates.

| Region | Cohen’s d | SE | CI LB | CI UB | P-value | FDR P-value | I^2^ | Controls | Patients |
| --- | --- | --- | --- | --- | --- | --- | --- | --- | --- |
| ACR | 0.089 | 0.136 | -0.178 | 0.355 | 0.514 | 0.805 | 52.055 | 290 | 372 |
| ALIC | 0.036 | 0.150 | -0.257 | 0.329 | 0.808 | 0.901 | 60.163 | 290 | 372 |
| BCC | 0.209 | 0.090 | 0.032 | 0.386 | 0.020 | 0.427 | 0.000 | 290 | 372 |
| CC | 0.213 | 0.090 | 0.035 | 0.390 | 0.019 | 0.427 | 0.000 | 290 | 372 |
| CGC | 0.029 | 0.139 | -0.245 | 0.302 | 0.838 | 0.918 | 54.434 | 290 | 372 |
| CGH | -0.062 | 0.172 | -0.399 | 0.275 | 0.719 | 0.880 | 69.874 | 290 | 372 |
| CR | 0.098 | 0.131 | -0.159 | 0.355 | 0.456 | 0.775 | 48.693 | 290 | 372 |
| CST | 0.055 | 0.105 | -0.151 | 0.261 | 0.600 | 0.870 | 22.509 | 290 | 372 |
| EC | -0.053 | 0.154 | -0.354 | 0.249 | 0.732 | 0.882 | 62.377 | 290 | 372 |
| FX | 0.143 | 0.108 | -0.069 | 0.354 | 0.186 | 0.579 | 25.529 | 290 | 372 |
| FXST | 0.062 | 0.150 | -0.233 | 0.356 | 0.681 | 0.880 | 60.500 | 290 | 372 |
| GCC | 0.228 | 0.110 | 0.012 | 0.444 | 0.038 | 0.427 | 28.512 | 290 | 372 |
| IC | 0.016 | 0.109 | -0.198 | 0.231 | 0.880 | 0.918 | 27.611 | 290 | 372 |
| IFO | 0.162 | 0.145 | -0.121 | 0.446 | 0.262 | 0.632 | 57.429 | 290 | 372 |
| PCR | 0.111 | 0.101 | -0.087 | 0.310 | 0.272 | 0.632 | 17.317 | 290 | 372 |
| PLIC | -0.099 | 0.090 | -0.276 | 0.078 | 0.272 | 0.632 | 0.000 | 290 | 372 |
| PTR | 0.099 | 0.126 | -0.147 | 0.346 | 0.429 | 0.775 | 44.262 | 290 | 372 |
| RLIC | 0.061 | 0.103 | -0.140 | 0.263 | 0.552 | 0.824 | 19.498 | 290 | 372 |
| SCC | 0.073 | 0.094 | -0.111 | 0.256 | 0.438 | 0.775 | 5.877 | 290 | 372 |
| SCR | 0.094 | 0.130 | -0.161 | 0.349 | 0.470 | 0.775 | 47.825 | 290 | 372 |
| SFO | 0.052 | 0.143 | -0.228 | 0.332 | 0.716 | 0.880 | 56.368 | 290 | 372 |
| SLF | 0.093 | 0.114 | -0.129 | 0.316 | 0.411 | 0.775 | 32.349 | 290 | 372 |
| SS | 0.078 | 0.127 | -0.172 | 0.327 | 0.540 | 0.824 | 45.583 | 290 | 372 |
| UNC | 0.063 | 0.104 | -0.141 | 0.266 | 0.545 | 0.824 | 20.647 | 290 | 372 |
| AverageRD | 0.132 | 0.132 | -0.127 | 0.392 | 0.318 | 0.669 | 49.624 | 290 | 372 |

Table S120. Cohen’s d effect sizes after meta-analysis, for FA differences between patients and controls **between age 10 and 20**. Age, sex, agexsex, age^2^, age^2^xsex and scansite included as covariates.

| Region | Cohen’s d | SE | CI LB | CI UB | P-value | FDR P-value | I^2^ | Controls | Patients |
| --- | --- | --- | --- | --- | --- | --- | --- | --- | --- |
| ACR | -0.092 | 0.266 | -0.614 | 0.430 | 0.730 | 0.949 | 77.360 | 113 | 262 |
| ALIC | 0.083 | 0.332 | -0.568 | 0.734 | 0.802 | 0.949 | 85.277 | 113 | 262 |
| BCC | -0.241 | 0.120 | -0.477 | -0.005 | 0.045 | 0.442 | 0.000 | 113 | 262 |
| CC | -0.257 | 0.121 | -0.493 | -0.020 | 0.033 | 0.442 | 0.000 | 113 | 262 |
| CGC | 0.158 | 0.234 | -0.301 | 0.618 | 0.500 | 0.949 | 70.901 | 113 | 262 |
| CGH | 0.106 | 0.270 | -0.422 | 0.635 | 0.693 | 0.949 | 77.862 | 113 | 262 |
| CR | -0.161 | 0.223 | -0.597 | 0.276 | 0.471 | 0.949 | 67.823 | 113 | 262 |
| CST | -0.233 | 0.120 | -0.469 | 0.003 | 0.053 | 0.442 | 0.000 | 113 | 262 |
| EC | 0.297 | 0.318 | -0.327 | 0.920 | 0.351 | 0.949 | 83.856 | 113 | 262 |
| FX | -0.018 | 0.120 | -0.254 | 0.217 | 0.879 | 0.949 | 0.000 | 113 | 262 |
| FXST | 0.059 | 0.264 | -0.458 | 0.576 | 0.824 | 0.949 | 76.855 | 113 | 262 |
| GCC | -0.225 | 0.177 | -0.571 | 0.122 | 0.204 | 0.900 | 49.429 | 113 | 262 |
| IC | 0.063 | 0.208 | -0.345 | 0.471 | 0.763 | 0.949 | 63.115 | 113 | 262 |
| IFO | -0.017 | 0.226 | -0.460 | 0.425 | 0.939 | 0.949 | 68.737 | 113 | 262 |
| PCR | -0.149 | 0.120 | -0.384 | 0.087 | 0.216 | 0.900 | 0.000 | 113 | 262 |
| PLIC | 0.031 | 0.172 | -0.306 | 0.368 | 0.857 | 0.949 | 46.895 | 113 | 262 |
| PTR | 0.084 | 0.202 | -0.311 | 0.479 | 0.676 | 0.949 | 60.655 | 113 | 262 |
| RLIC | -0.008 | 0.120 | -0.243 | 0.228 | 0.949 | 0.949 | 0.000 | 113 | 262 |
| SCC | -0.149 | 0.120 | -0.385 | 0.086 | 0.215 | 0.900 | 0.000 | 113 | 262 |
| SCR | -0.175 | 0.192 | -0.551 | 0.201 | 0.362 | 0.949 | 56.971 | 113 | 262 |
| SFO | -0.047 | 0.207 | -0.453 | 0.358 | 0.820 | 0.949 | 62.776 | 113 | 262 |
| SLF | -0.105 | 0.138 | -0.376 | 0.166 | 0.449 | 0.949 | 20.929 | 113 | 262 |
| SS | 0.040 | 0.195 | -0.342 | 0.422 | 0.838 | 0.949 | 58.037 | 113 | 262 |
| UNC | -0.090 | 0.120 | -0.326 | 0.145 | 0.451 | 0.949 | 0.005 | 113 | 262 |
| AverageFA | -0.175 | 0.339 | -0.839 | 0.489 | 0.606 | 0.949 | 77.657 | 77 | 194 |

Table S121. Cohen’s d effect sizes after meta-analysis, for AD differences between patients and controls **between age 10 and 20**. Age, sex, agexsex, age^2^, age^2^xsex and scansite included as covariates.

| Region | Cohen’s d | SE | CI LB | CI UB | P-value | FDR P-value | I^2^ | Controls | Patients |
| --- | --- | --- | --- | --- | --- | --- | --- | --- | --- |
| ACR | 0.160 | 0.120 | -0.076 | 0.395 | 0.184 | 0.354 | 0.000 | 113 | 262 |
| ALIC | 0.242 | 0.169 | -0.089 | 0.572 | 0.152 | 0.345 | 44.091 | 113 | 262 |
| BCC | -0.013 | 0.120 | -0.248 | 0.222 | 0.912 | 0.914 | 0.000 | 113 | 262 |
| CC | 0.030 | 0.120 | -0.205 | 0.266 | 0.800 | 0.909 | 0.000 | 113 | 262 |
| CGC | 0.433 | 0.149 | 0.141 | 0.725 | 0.004 | 0.067 | 29.274 | 113 | 262 |
| CGH | -0.033 | 0.227 | -0.478 | 0.411 | 0.883 | 0.914 | 69.220 | 113 | 262 |
| CR | 0.187 | 0.120 | -0.049 | 0.423 | 0.120 | 0.300 | 0.000 | 113 | 262 |
| CST | -0.254 | 0.133 | -0.515 | 0.007 | 0.057 | 0.250 | 15.198 | 113 | 262 |
| EC | 0.327 | 0.122 | 0.087 | 0.566 | 0.008 | 0.067 | 1.889 | 113 | 262 |
| FX | 0.250 | 0.241 | -0.222 | 0.723 | 0.299 | 0.467 | 72.062 | 113 | 262 |
| FXST | 0.136 | 0.173 | -0.204 | 0.476 | 0.433 | 0.601 | 47.600 | 113 | 262 |
| GCC | 0.101 | 0.120 | -0.134 | 0.337 | 0.399 | 0.587 | 0.000 | 113 | 262 |
| IC | 0.221 | 0.121 | -0.015 | 0.458 | 0.067 | 0.250 | 0.004 | 113 | 262 |
| IFO | 0.103 | 0.142 | -0.175 | 0.381 | 0.466 | 0.613 | 24.171 | 113 | 262 |
| PCR | 0.218 | 0.120 | -0.018 | 0.453 | 0.070 | 0.250 | 0.000 | 113 | 262 |
| PLIC | 0.021 | 0.198 | -0.367 | 0.410 | 0.914 | 0.914 | 59.772 | 113 | 262 |
| PTR | 0.330 | 0.121 | 0.092 | 0.568 | 0.007 | 0.067 | 0.007 | 113 | 262 |
| RLIC | 0.210 | 0.120 | -0.026 | 0.446 | 0.081 | 0.253 | 0.000 | 113 | 262 |
| SCC | 0.031 | 0.120 | -0.205 | 0.266 | 0.798 | 0.909 | 0.000 | 113 | 262 |
| SCR | 0.077 | 0.120 | -0.159 | 0.312 | 0.523 | 0.654 | 0.000 | 113 | 262 |
| SFO | 0.188 | 0.120 | -0.048 | 0.424 | 0.118 | 0.300 | 0.000 | 113 | 262 |
| SLF | 0.258 | 0.120 | 0.022 | 0.494 | 0.032 | 0.200 | 0.000 | 113 | 262 |
| SS | 0.140 | 0.120 | -0.096 | 0.375 | 0.246 | 0.410 | 0.000 | 113 | 262 |
| UNC | 0.164 | 0.120 | -0.071 | 0.400 | 0.172 | 0.354 | 0.000 | 113 | 262 |
| AverageAD | 0.206 | 0.167 | -0.122 | 0.534 | 0.218 | 0.389 | 16.414 | 77 | 194 |

Table S122. Cohen’s d effect sizes after meta-analysis, for MD differences between patients and controls **between age 10 and 20**. Age, sex, agexsex, age^2^, age^2^xsex and scansite included as covariates.

| Region | Cohen’s d | SE | CI LB | CI UB | P-value | FDR P-value | I^2^ | Controls | Patients |
| --- | --- | --- | --- | --- | --- | --- | --- | --- | --- |
| ACR | 0.202 | 0.160 | -0.112 | 0.517 | 0.207 | 0.301 | 39.111 | 113 | 262 |
| ALIC | 0.181 | 0.120 | -0.055 | 0.417 | 0.133 | 0.285 | 0.000 | 113 | 262 |
| BCC | 0.195 | 0.120 | -0.040 | 0.431 | 0.104 | 0.285 | 0.000 | 113 | 262 |
| CC | 0.211 | 0.126 | -0.037 | 0.458 | 0.095 | 0.285 | 7.197 | 113 | 262 |
| CGC | 0.222 | 0.141 | -0.055 | 0.498 | 0.116 | 0.285 | 23.223 | 113 | 262 |
| CGH | -0.110 | 0.307 | -0.713 | 0.492 | 0.720 | 0.769 | 83.045 | 113 | 262 |
| CR | 0.262 | 0.136 | -0.004 | 0.528 | 0.054 | 0.254 | 17.629 | 113 | 262 |
| CST | -0.126 | 0.140 | -0.401 | 0.149 | 0.368 | 0.438 | 22.771 | 113 | 262 |
| EC | 0.192 | 0.159 | -0.119 | 0.503 | 0.226 | 0.301 | 37.968 | 113 | 262 |
| FX | 0.279 | 0.213 | -0.137 | 0.696 | 0.189 | 0.301 | 64.164 | 113 | 262 |
| FXST | 0.129 | 0.176 | -0.217 | 0.474 | 0.466 | 0.530 | 49.155 | 113 | 262 |
| GCC | 0.214 | 0.154 | -0.087 | 0.515 | 0.163 | 0.301 | 34.123 | 113 | 262 |
| IC | 0.148 | 0.120 | -0.088 | 0.383 | 0.219 | 0.301 | 0.000 | 113 | 262 |
| IFO | 0.196 | 0.167 | -0.132 | 0.523 | 0.241 | 0.301 | 43.677 | 113 | 262 |
| PCR | 0.261 | 0.136 | -0.006 | 0.528 | 0.056 | 0.254 | 18.283 | 113 | 262 |
| PLIC | -0.035 | 0.120 | -0.271 | 0.200 | 0.769 | 0.769 | 0.000 | 113 | 262 |
| PTR | 0.278 | 0.137 | 0.010 | 0.546 | 0.042 | 0.254 | 18.616 | 113 | 262 |
| RLIC | 0.226 | 0.121 | -0.011 | 0.462 | 0.061 | 0.254 | 0.000 | 113 | 262 |
| SCC | 0.143 | 0.120 | -0.093 | 0.379 | 0.235 | 0.301 | 0.000 | 113 | 262 |
| SCR | 0.256 | 0.124 | 0.014 | 0.499 | 0.038 | 0.254 | 3.850 | 113 | 262 |
| SFO | 0.221 | 0.148 | -0.070 | 0.511 | 0.137 | 0.285 | 29.566 | 113 | 262 |
| SLF | 0.287 | 0.121 | 0.050 | 0.524 | 0.017 | 0.254 | 0.000 | 113 | 262 |
| SS | 0.072 | 0.220 | -0.359 | 0.502 | 0.745 | 0.769 | 67.027 | 113 | 262 |
| UNC | 0.174 | 0.149 | -0.117 | 0.466 | 0.241 | 0.301 | 30.378 | 113 | 262 |
| AverageMD | 0.340 | 0.206 | -0.063 | 0.743 | 0.098 | 0.285 | 41.343 | 77 | 194 |

Table S123. Cohen’s d effect sizes after meta-analysis, for RD differences between patients and controls **between age 10 and 20**. Age, sex, agexsex, age^2^, age^2^xsex and scansite included as covariates.

| Region | Cohen’s d | SE | CI LB | CI UB | P-value | FDR P-value | I^2^ | Controls | Patients |
| --- | --- | --- | --- | --- | --- | --- | --- | --- | --- |
| ACR | 0.173 | 0.242 | -0.300 | 0.647 | 0.473 | 0.802 | 72.565 | 113 | 262 |
| ALIC | -0.009 | 0.278 | -0.554 | 0.536 | 0.975 | 0.987 | 79.176 | 113 | 262 |
| BCC | 0.237 | 0.120 | 0.001 | 0.473 | 0.049 | 0.638 | 0.000 | 113 | 262 |
| CC | 0.252 | 0.130 | -0.003 | 0.507 | 0.053 | 0.638 | 11.567 | 113 | 262 |
| CGC | -0.040 | 0.240 | -0.510 | 0.430 | 0.868 | 0.987 | 72.293 | 113 | 262 |
| CGH | -0.148 | 0.360 | -0.853 | 0.557 | 0.681 | 0.946 | 87.480 | 113 | 262 |
| CR | 0.227 | 0.212 | -0.189 | 0.643 | 0.285 | 0.659 | 64.513 | 113 | 262 |
| CST | 0.028 | 0.123 | -0.213 | 0.269 | 0.822 | 0.987 | 3.668 | 113 | 262 |
| EC | 0.016 | 0.270 | -0.512 | 0.545 | 0.952 | 0.987 | 77.896 | 113 | 262 |
| FX | 0.269 | 0.184 | -0.092 | 0.630 | 0.144 | 0.638 | 52.645 | 113 | 262 |
| FXST | 0.031 | 0.252 | -0.462 | 0.524 | 0.901 | 0.987 | 74.623 | 113 | 262 |
| GCC | 0.223 | 0.193 | -0.155 | 0.601 | 0.247 | 0.659 | 57.220 | 113 | 262 |
| IC | 0.045 | 0.160 | -0.269 | 0.360 | 0.777 | 0.987 | 39.247 | 113 | 262 |
| IFO | 0.155 | 0.209 | -0.253 | 0.564 | 0.456 | 0.802 | 63.407 | 113 | 262 |
| PCR | 0.224 | 0.148 | -0.067 | 0.515 | 0.132 | 0.638 | 29.911 | 113 | 262 |
| PLIC | -0.078 | 0.120 | -0.314 | 0.157 | 0.515 | 0.805 | 0.000 | 113 | 262 |
| PTR | 0.124 | 0.176 | -0.221 | 0.470 | 0.481 | 0.802 | 49.260 | 113 | 262 |
| RLIC | 0.141 | 0.133 | -0.120 | 0.403 | 0.290 | 0.659 | 15.761 | 113 | 262 |
| SCC | 0.153 | 0.120 | -0.083 | 0.389 | 0.204 | 0.638 | 0.000 | 113 | 262 |
| SCR | 0.268 | 0.197 | -0.117 | 0.654 | 0.172 | 0.638 | 58.736 | 113 | 262 |
| SFO | 0.134 | 0.252 | -0.360 | 0.628 | 0.595 | 0.875 | 74.729 | 113 | 262 |
| SLF | 0.221 | 0.152 | -0.077 | 0.519 | 0.146 | 0.638 | 32.902 | 113 | 262 |
| SS | 0.004 | 0.257 | -0.500 | 0.509 | 0.987 | 0.987 | 75.855 | 113 | 262 |
| UNC | 0.112 | 0.130 | -0.143 | 0.366 | 0.391 | 0.802 | 11.954 | 113 | 262 |
| AverageRD | 0.351 | 0.266 | -0.171 | 0.874 | 0.187 | 0.638 | 64.204 | 77 | 194 |

Table S124. Cohen’s d effect sizes after meta-analysis, for FA differences between patients and controls **between age 20 and 30**. Age, sex, agexsex, age^2^, age^2^xsex and scansite included as covariates.

| Region | Cohen’s d | SE | CI LB | CI UB | P-value | FDR P-value | I^2^ | Controls | Patients |
| --- | --- | --- | --- | --- | --- | --- | --- | --- | --- |
| ACR | -0.082 | 0.077 | -0.233 | 0.069 | 0.287 | 0.738 | 0.000 | 616 | 271 |
| ALIC | -0.081 | 0.077 | -0.232 | 0.070 | 0.295 | 0.738 | 0.000 | 616 | 271 |
| BCC | -0.128 | 0.144 | -0.410 | 0.153 | 0.372 | 0.845 | 66.194 | 616 | 271 |
| CC | -0.077 | 0.143 | -0.357 | 0.204 | 0.592 | 0.914 | 65.877 | 616 | 271 |
| CGC | -0.115 | 0.077 | -0.267 | 0.036 | 0.136 | 0.738 | 0.007 | 616 | 271 |
| CGH | -0.133 | 0.077 | -0.284 | 0.019 | 0.086 | 0.717 | 0.003 | 616 | 271 |
| CR | -0.037 | 0.077 | -0.188 | 0.114 | 0.632 | 0.914 | 0.000 | 616 | 271 |
| CST | 0.018 | 0.077 | -0.133 | 0.169 | 0.816 | 0.927 | 0.002 | 616 | 271 |
| EC | -0.093 | 0.077 | -0.245 | 0.058 | 0.227 | 0.738 | 0.004 | 616 | 271 |
| FX | -0.096 | 0.077 | -0.248 | 0.055 | 0.213 | 0.738 | 0.006 | 616 | 271 |
| FXST | -0.163 | 0.077 | -0.315 | -0.012 | 0.035 | 0.717 | 0.014 | 616 | 271 |
| GCC | -0.048 | 0.109 | -0.262 | 0.165 | 0.658 | 0.914 | 42.496 | 616 | 271 |
| IC | -0.040 | 0.077 | -0.191 | 0.111 | 0.604 | 0.914 | 0.000 | 616 | 271 |
| IFO | -0.098 | 0.088 | -0.271 | 0.074 | 0.263 | 0.738 | 17.115 | 616 | 271 |
| PCR | 0.008 | 0.105 | -0.197 | 0.213 | 0.938 | 0.987 | 37.888 | 616 | 271 |
| PLIC | 0.025 | 0.077 | -0.127 | 0.176 | 0.749 | 0.927 | 0.000 | 616 | 271 |
| PTR | -0.064 | 0.077 | -0.216 | 0.087 | 0.406 | 0.846 | 0.012 | 616 | 271 |
| RLIC | -0.055 | 0.077 | -0.206 | 0.097 | 0.478 | 0.914 | 0.000 | 616 | 271 |
| SCC | 0.041 | 0.113 | -0.181 | 0.264 | 0.715 | 0.927 | 46.626 | 616 | 271 |
| SCR | 0.001 | 0.077 | -0.150 | 0.152 | 0.987 | 0.987 | 0.000 | 616 | 271 |
| SFO | -0.134 | 0.077 | -0.285 | 0.018 | 0.084 | 0.717 | 0.000 | 616 | 271 |
| SLF | -0.003 | 0.077 | -0.155 | 0.148 | 0.966 | 0.987 | 0.020 | 616 | 271 |
| SS | -0.064 | 0.099 | -0.258 | 0.130 | 0.520 | 0.914 | 31.774 | 616 | 271 |
| UNC | -0.024 | 0.093 | -0.206 | 0.159 | 0.799 | 0.927 | 24.286 | 616 | 271 |
| AverageFA | -0.104 | 0.083 | -0.267 | 0.060 | 0.213 | 0.738 | 0.574 | 504 | 241 |

Table S125. Cohen’s d effect sizes after meta-analysis, for AD differences between patients and controls **between age 20 and 30**. Age, sex, agexsex, age^2^, age^2^xsex and scansite included as covariates.

| Region | Cohen’s d | SE | CI LB | CI UB | P-value | FDR P-value | I^2^ | Controls | Patients |
| --- | --- | --- | --- | --- | --- | --- | --- | --- | --- |
| ACR | 0.031 | 0.168 | -0.299 | 0.360 | 0.856 | 0.982 | 75.341 | 616 | 271 |
| ALIC | 0.089 | 0.139 | -0.183 | 0.361 | 0.521 | 0.982 | 63.843 | 616 | 271 |
| BCC | -0.003 | 0.095 | -0.188 | 0.183 | 0.978 | 0.994 | 26.338 | 616 | 271 |
| CC | 0.011 | 0.136 | -0.255 | 0.277 | 0.935 | 0.994 | 62.184 | 616 | 271 |
| CGC | -0.033 | 0.077 | -0.184 | 0.118 | 0.668 | 0.982 | 0.003 | 616 | 271 |
| CGH | 0.040 | 0.096 | -0.148 | 0.228 | 0.677 | 0.982 | 27.987 | 616 | 271 |
| CR | 0.071 | 0.144 | -0.211 | 0.353 | 0.622 | 0.982 | 66.240 | 616 | 271 |
| CST | 0.121 | 0.099 | -0.073 | 0.315 | 0.221 | 0.982 | 31.739 | 616 | 271 |
| EC | 0.042 | 0.115 | -0.183 | 0.266 | 0.716 | 0.982 | 47.649 | 616 | 271 |
| FX | 0.130 | 0.077 | -0.021 | 0.281 | 0.092 | 0.982 | 0.000 | 616 | 271 |
| FXST | 0.090 | 0.077 | -0.061 | 0.242 | 0.242 | 0.982 | 0.000 | 616 | 271 |
| GCC | 0.058 | 0.143 | -0.223 | 0.339 | 0.685 | 0.982 | 66.086 | 616 | 271 |
| IC | 0.053 | 0.142 | -0.224 | 0.331 | 0.707 | 0.982 | 65.225 | 616 | 271 |
| IFO | 0.061 | 0.077 | -0.090 | 0.213 | 0.428 | 0.982 | 0.000 | 616 | 271 |
| PCR | 0.036 | 0.100 | -0.161 | 0.232 | 0.721 | 0.982 | 33.320 | 616 | 271 |
| PLIC | 0.114 | 0.136 | -0.153 | 0.381 | 0.401 | 0.982 | 62.395 | 616 | 271 |
| PTR | 0.073 | 0.099 | -0.121 | 0.266 | 0.463 | 0.982 | 31.683 | 616 | 271 |
| RLIC | 0.001 | 0.122 | -0.238 | 0.239 | 0.994 | 0.994 | 53.200 | 616 | 271 |
| SCC | 0.035 | 0.178 | -0.314 | 0.384 | 0.844 | 0.982 | 77.963 | 616 | 271 |
| SCR | 0.121 | 0.132 | -0.137 | 0.380 | 0.358 | 0.982 | 59.780 | 616 | 271 |
| SFO | 0.018 | 0.077 | -0.133 | 0.170 | 0.812 | 0.982 | 0.336 | 616 | 271 |
| SLF | 0.058 | 0.121 | -0.179 | 0.296 | 0.631 | 0.982 | 52.995 | 616 | 271 |
| SS | 0.067 | 0.082 | -0.094 | 0.228 | 0.414 | 0.982 | 8.311 | 616 | 271 |
| UNC | 0.021 | 0.123 | -0.221 | 0.263 | 0.864 | 0.982 | 54.459 | 616 | 271 |
| AverageAD | 0.068 | 0.105 | -0.138 | 0.274 | 0.519 | 0.982 | 33.839 | 504 | 241 |

Table S126. Cohen’s d effect sizes after meta-analysis, for MD differences between patients and controls **between age 20 and 30**. Age, sex, agexsex, age^2^, age^2^xsex and scansite included as covariates.

| Region | Cohen’s d | SE | CI LB | CI UB | P-value | FDR P-value | I^2^ | Controls | Patients |
| --- | --- | --- | --- | --- | --- | --- | --- | --- | --- |
| ACR | -0.073 | 0.149 | -0.364 | 0.218 | 0.623 | 0.926 | 68.339 | 616 | 271 |
| ALIC | 0.000 | 0.127 | -0.249 | 0.248 | 0.997 | 0.997 | 56.831 | 616 | 271 |
| BCC | 0.102 | 0.118 | -0.130 | 0.334 | 0.387 | 0.926 | 50.684 | 616 | 271 |
| CC | 0.072 | 0.154 | -0.229 | 0.373 | 0.641 | 0.926 | 70.423 | 616 | 271 |
| CGC | 0.015 | 0.077 | -0.136 | 0.166 | 0.846 | 0.960 | 0.011 | 616 | 271 |
| CGH | 0.186 | 0.126 | -0.060 | 0.432 | 0.139 | 0.845 | 55.565 | 616 | 271 |
| CR | -0.005 | 0.137 | -0.274 | 0.263 | 0.968 | 0.997 | 62.723 | 616 | 271 |
| CST | 0.015 | 0.100 | -0.182 | 0.211 | 0.883 | 0.960 | 33.268 | 616 | 271 |
| EC | 0.068 | 0.105 | -0.137 | 0.273 | 0.515 | 0.926 | 38.117 | 616 | 271 |
| FX | 0.130 | 0.077 | -0.022 | 0.281 | 0.093 | 0.845 | 0.008 | 616 | 271 |
| FXST | 0.160 | 0.077 | 0.008 | 0.312 | 0.039 | 0.845 | 0.009 | 616 | 271 |
| GCC | 0.032 | 0.155 | -0.272 | 0.336 | 0.838 | 0.960 | 71.082 | 616 | 271 |
| IC | -0.018 | 0.103 | -0.219 | 0.183 | 0.862 | 0.960 | 35.883 | 616 | 271 |
| IFO | 0.123 | 0.089 | -0.052 | 0.298 | 0.169 | 0.845 | 19.207 | 616 | 271 |
| PCR | 0.074 | 0.125 | -0.171 | 0.318 | 0.555 | 0.926 | 55.242 | 616 | 271 |
| PLIC | -0.055 | 0.080 | -0.211 | 0.102 | 0.493 | 0.926 | 4.582 | 616 | 271 |
| PTR | 0.136 | 0.109 | -0.079 | 0.350 | 0.215 | 0.896 | 42.660 | 616 | 271 |
| RLIC | 0.052 | 0.102 | -0.148 | 0.251 | 0.610 | 0.926 | 34.977 | 616 | 271 |
| SCC | 0.062 | 0.159 | -0.250 | 0.373 | 0.698 | 0.926 | 72.393 | 616 | 271 |
| SCR | 0.045 | 0.118 | -0.187 | 0.277 | 0.704 | 0.926 | 50.559 | 616 | 271 |
| SFO | 0.127 | 0.085 | -0.039 | 0.292 | 0.134 | 0.845 | 11.898 | 616 | 271 |
| SLF | 0.059 | 0.111 | -0.158 | 0.277 | 0.593 | 0.926 | 44.209 | 616 | 271 |
| SS | 0.097 | 0.088 | -0.075 | 0.268 | 0.270 | 0.926 | 16.565 | 616 | 271 |
| UNC | 0.176 | 0.184 | -0.183 | 0.536 | 0.337 | 0.926 | 79.245 | 616 | 271 |
| AverageMD | 0.064 | 0.100 | -0.132 | 0.261 | 0.522 | 0.926 | 27.877 | 504 | 241 |

Table S127. Cohen’s d effect sizes after meta-analysis, for RD differences between patients and controls **between age 20 and 30**. Age, sex, agexsex, age^2^, age^2^xsex and scansite included as covariates.

| Region | Cohen’s d | SE | CI LB | CI UB | P-value | FDR P-value | I^2^ | Controls | Patients |
| --- | --- | --- | --- | --- | --- | --- | --- | --- | --- |
| ACR | -0.055 | 0.130 | -0.309 | 0.200 | 0.674 | 0.892 | 58.626 | 616 | 271 |
| ALIC | 0.038 | 0.077 | -0.113 | 0.189 | 0.623 | 0.892 | 0.005 | 616 | 271 |
| BCC | 0.116 | 0.113 | -0.105 | 0.337 | 0.304 | 0.760 | 45.979 | 616 | 271 |
| CC | 0.068 | 0.137 | -0.200 | 0.336 | 0.618 | 0.892 | 62.803 | 616 | 271 |
| CGC | 0.058 | 0.092 | -0.122 | 0.238 | 0.526 | 0.892 | 22.437 | 616 | 271 |
| CGH | 0.213 | 0.114 | -0.011 | 0.436 | 0.062 | 0.517 | 46.706 | 616 | 271 |
| CR | -0.004 | 0.104 | -0.208 | 0.201 | 0.972 | 0.972 | 37.849 | 616 | 271 |
| CST | -0.010 | 0.077 | -0.161 | 0.142 | 0.900 | 0.972 | 0.000 | 616 | 271 |
| EC | 0.093 | 0.077 | -0.058 | 0.244 | 0.229 | 0.642 | 0.006 | 616 | 271 |
| FX | 0.129 | 0.077 | -0.022 | 0.280 | 0.095 | 0.594 | 0.000 | 616 | 271 |
| FXST | 0.179 | 0.077 | 0.027 | 0.330 | 0.021 | 0.517 | 0.005 | 616 | 271 |
| GCC | 0.020 | 0.129 | -0.232 | 0.273 | 0.876 | 0.972 | 58.185 | 616 | 271 |
| IC | 0.019 | 0.077 | -0.132 | 0.171 | 0.801 | 0.972 | 0.000 | 616 | 271 |
| IFO | 0.155 | 0.104 | -0.049 | 0.359 | 0.136 | 0.642 | 37.344 | 616 | 271 |
| PCR | 0.064 | 0.130 | -0.190 | 0.319 | 0.619 | 0.892 | 58.553 | 616 | 271 |
| PLIC | -0.037 | 0.077 | -0.188 | 0.114 | 0.631 | 0.892 | 0.000 | 616 | 271 |
| PTR | 0.150 | 0.118 | -0.082 | 0.382 | 0.204 | 0.642 | 50.415 | 616 | 271 |
| RLIC | 0.062 | 0.077 | -0.089 | 0.214 | 0.419 | 0.873 | 0.000 | 616 | 271 |
| SCC | 0.010 | 0.145 | -0.273 | 0.294 | 0.944 | 0.972 | 66.630 | 616 | 271 |
| SCR | 0.032 | 0.077 | -0.119 | 0.183 | 0.678 | 0.892 | 0.009 | 616 | 271 |
| SFO | 0.164 | 0.086 | -0.005 | 0.332 | 0.057 | 0.517 | 13.983 | 616 | 271 |
| SLF | 0.008 | 0.077 | -0.143 | 0.160 | 0.916 | 0.972 | 0.002 | 616 | 271 |
| SS | 0.117 | 0.096 | -0.072 | 0.305 | 0.225 | 0.642 | 28.167 | 616 | 271 |
| UNC | 0.175 | 0.146 | -0.112 | 0.462 | 0.231 | 0.642 | 67.247 | 616 | 271 |
| AverageRD | 0.077 | 0.089 | -0.098 | 0.253 | 0.389 | 0.873 | 11.748 | 504 | 241 |

Table S128. Cohen’s d effect sizes after meta-analysis, for FA differences between patients and controls **between age 30 and 40**. Age, sex, agexsex, age^2^, age^2^xsex and scansite included as covariates.

| Region | Cohen’s d | SE | CI LB | CI UB | P-value | FDR P-value | I^2^ | Controls | Patients |
| --- | --- | --- | --- | --- | --- | --- | --- | --- | --- |
| ACR | -0.407 | 0.177 | -0.754 | -0.061 | 0.021 | 0.070 | 43.527 | 182 | 119 |
| ALIC | -0.352 | 0.122 | -0.592 | -0.112 | 0.004 | 0.050 | 0.000 | 182 | 119 |
| BCC | -0.146 | 0.122 | -0.385 | 0.092 | 0.230 | 0.303 | 0.000 | 182 | 119 |
| CC | -0.228 | 0.122 | -0.467 | 0.011 | 0.061 | 0.102 | 0.000 | 182 | 119 |
| CGC | -0.174 | 0.122 | -0.413 | 0.065 | 0.154 | 0.226 | 0.000 | 182 | 119 |
| CGH | 0.039 | 0.122 | -0.200 | 0.277 | 0.751 | 0.751 | 0.000 | 182 | 119 |
| CR | -0.433 | 0.170 | -0.767 | -0.100 | 0.011 | 0.060 | 39.236 | 182 | 119 |
| CST | -0.209 | 0.259 | -0.717 | 0.299 | 0.420 | 0.457 | 73.350 | 182 | 119 |
| EC | -0.106 | 0.164 | -0.427 | 0.215 | 0.517 | 0.539 | 36.492 | 182 | 119 |
| FX | 0.180 | 0.122 | -0.059 | 0.419 | 0.139 | 0.217 | 0.000 | 182 | 119 |
| FXST | -0.216 | 0.231 | -0.669 | 0.236 | 0.349 | 0.436 | 66.583 | 182 | 119 |
| GCC | -0.273 | 0.122 | -0.513 | -0.034 | 0.025 | 0.070 | 0.000 | 182 | 119 |
| IC | -0.309 | 0.122 | -0.548 | -0.069 | 0.012 | 0.060 | 0.000 | 182 | 119 |
| IFO | -0.271 | 0.123 | -0.512 | -0.031 | 0.027 | 0.070 | 0.005 | 182 | 119 |
| PCR | -0.430 | 0.123 | -0.671 | -0.189 | 0.000 | 0.000 | 0.000 | 182 | 119 |
| PLIC | -0.271 | 0.128 | -0.521 | -0.021 | 0.034 | 0.077 | 5.645 | 182 | 119 |
| PTR | -0.354 | 0.175 | -0.696 | -0.012 | 0.043 | 0.090 | 42.478 | 182 | 119 |
| RLIC | -0.100 | 0.122 | -0.339 | 0.138 | 0.409 | 0.457 | 0.000 | 182 | 119 |
| SCC | -0.238 | 0.122 | -0.478 | 0.002 | 0.052 | 0.100 | 0.005 | 182 | 119 |
| SCR | -0.313 | 0.167 | -0.641 | 0.015 | 0.061 | 0.102 | 38.163 | 182 | 119 |
| SFO | -0.268 | 0.122 | -0.508 | -0.028 | 0.028 | 0.070 | 0.004 | 182 | 119 |
| SLF | -0.154 | 0.122 | -0.392 | 0.085 | 0.206 | 0.286 | 0.000 | 182 | 119 |
| SS | -0.103 | 0.122 | -0.342 | 0.137 | 0.401 | 0.457 | 0.008 | 182 | 119 |
| UNC | -0.325 | 0.122 | -0.565 | -0.085 | 0.008 | 0.060 | 0.000 | 182 | 119 |
| AverageFA | -0.339 | 0.140 | -0.614 | -0.065 | 0.015 | 0.063 | 0.000 | 152 | 85 |

Table S129. Cohen’s d effect sizes after meta-analysis, for AD differences between patients and controls **between age 30 and 40**. Age, sex, agexsex, age^2^, age^2^xsex and scansite included as covariates.

| Region | Cohen’s d | SE | CI LB | CI UB | P-value | FDR P-value | I^2^ | Controls | Patients |
| --- | --- | --- | --- | --- | --- | --- | --- | --- | --- |
| ACR | 0.168 | 0.128 | -0.083 | 0.419 | 0.189 | 0.646 | 5.917 | 182 | 119 |
| ALIC | 0.205 | 0.272 | -0.328 | 0.738 | 0.451 | 0.646 | 75.917 | 182 | 119 |
| BCC | 0.197 | 0.176 | -0.149 | 0.542 | 0.264 | 0.646 | 43.852 | 182 | 119 |
| CC | 0.145 | 0.184 | -0.215 | 0.506 | 0.429 | 0.646 | 48.391 | 182 | 119 |
| CGC | 0.157 | 0.193 | -0.222 | 0.535 | 0.417 | 0.646 | 53.535 | 182 | 119 |
| CGH | 0.211 | 0.122 | -0.028 | 0.450 | 0.083 | 0.646 | 0.000 | 182 | 119 |
| CR | 0.164 | 0.122 | -0.075 | 0.403 | 0.179 | 0.646 | 0.000 | 182 | 119 |
| CST | -0.086 | 0.190 | -0.458 | 0.287 | 0.652 | 0.741 | 51.692 | 182 | 119 |
| EC | 0.091 | 0.122 | -0.147 | 0.330 | 0.453 | 0.646 | 0.000 | 182 | 119 |
| FX | 0.100 | 0.145 | -0.184 | 0.383 | 0.491 | 0.646 | 22.274 | 182 | 119 |
| FXST | 0.116 | 0.122 | -0.122 | 0.354 | 0.340 | 0.646 | 0.000 | 182 | 119 |
| GCC | 0.104 | 0.193 | -0.274 | 0.482 | 0.590 | 0.702 | 53.132 | 182 | 119 |
| IC | 0.040 | 0.149 | -0.253 | 0.333 | 0.789 | 0.858 | 26.151 | 182 | 119 |
| IFO | 0.118 | 0.129 | -0.135 | 0.371 | 0.360 | 0.646 | 7.263 | 182 | 119 |
| PCR | -0.121 | 0.122 | -0.360 | 0.118 | 0.321 | 0.646 | 0.000 | 182 | 119 |
| PLIC | 0.227 | 0.153 | -0.073 | 0.527 | 0.138 | 0.646 | 28.178 | 182 | 119 |
| PTR | 0.124 | 0.122 | -0.114 | 0.363 | 0.307 | 0.646 | 0.000 | 182 | 119 |
| RLIC | 0.088 | 0.122 | -0.151 | 0.326 | 0.472 | 0.646 | 0.000 | 182 | 119 |
| SCC | 0.006 | 0.122 | -0.233 | 0.245 | 0.959 | 0.959 | 0.000 | 182 | 119 |
| SCR | 0.279 | 0.122 | 0.039 | 0.519 | 0.023 | 0.575 | 0.005 | 182 | 119 |
| SFO | 0.208 | 0.213 | -0.209 | 0.624 | 0.329 | 0.646 | 60.764 | 182 | 119 |
| SLF | 0.067 | 0.122 | -0.171 | 0.305 | 0.582 | 0.702 | 0.000 | 182 | 119 |
| SS | 0.190 | 0.122 | -0.049 | 0.428 | 0.120 | 0.646 | 0.000 | 182 | 119 |
| UNC | -0.022 | 0.141 | -0.299 | 0.255 | 0.876 | 0.913 | 19.376 | 182 | 119 |
| AverageAD | 0.251 | 0.184 | -0.110 | 0.613 | 0.173 | 0.646 | 34.055 | 152 | 85 |

Table S130. Cohen’s d effect sizes after meta-analysis, for MD differences between patients and controls **between age 30 and 40**. Age, sex, agexsex, age^2^, age^2^xsex and scansite included as covariates.

| Region | Cohen’s d | SE | CI LB | CI UB | P-value | FDR P-value | I^2^ | Controls | Patients |
| --- | --- | --- | --- | --- | --- | --- | --- | --- | --- |
| ACR | 0.259 | 0.169 | -0.072 | 0.591 | 0.125 | 0.330 | 39.190 | 182 | 119 |
| ALIC | 0.252 | 0.218 | -0.175 | 0.679 | 0.247 | 0.441 | 62.056 | 182 | 119 |
| BCC | 0.178 | 0.122 | -0.061 | 0.418 | 0.145 | 0.330 | 0.002 | 182 | 119 |
| CC | 0.157 | 0.122 | -0.082 | 0.396 | 0.197 | 0.410 | 0.000 | 182 | 119 |
| CGC | 0.123 | 0.154 | -0.179 | 0.425 | 0.425 | 0.523 | 30.131 | 182 | 119 |
| CGH | 0.123 | 0.122 | -0.116 | 0.361 | 0.313 | 0.522 | 0.000 | 182 | 119 |
| CR | 0.284 | 0.123 | 0.044 | 0.525 | 0.020 | 0.144 | 0.004 | 182 | 119 |
| CST | -0.059 | 0.122 | -0.298 | 0.181 | 0.631 | 0.644 | 0.005 | 182 | 119 |
| EC | 0.148 | 0.122 | -0.091 | 0.387 | 0.225 | 0.433 | 0.000 | 182 | 119 |
| FX | -0.056 | 0.122 | -0.295 | 0.182 | 0.644 | 0.644 | 0.000 | 182 | 119 |
| FXST | 0.345 | 0.123 | 0.104 | 0.586 | 0.005 | 0.125 | 0.001 | 182 | 119 |
| GCC | 0.095 | 0.122 | -0.144 | 0.333 | 0.438 | 0.523 | 0.000 | 182 | 119 |
| IC | 0.087 | 0.122 | -0.151 | 0.326 | 0.472 | 0.523 | 0.000 | 182 | 119 |
| IFO | 0.184 | 0.221 | -0.249 | 0.617 | 0.405 | 0.523 | 63.489 | 182 | 119 |
| PCR | 0.247 | 0.122 | 0.008 | 0.486 | 0.042 | 0.210 | 0.000 | 182 | 119 |
| PLIC | 0.169 | 0.205 | -0.232 | 0.570 | 0.409 | 0.523 | 57.779 | 182 | 119 |
| PTR | 0.279 | 0.122 | 0.039 | 0.519 | 0.023 | 0.144 | 0.001 | 182 | 119 |
| RLIC | 0.086 | 0.122 | -0.153 | 0.324 | 0.481 | 0.523 | 0.000 | 182 | 119 |
| SCC | 0.091 | 0.122 | -0.147 | 0.330 | 0.454 | 0.523 | 0.000 | 182 | 119 |
| SCR | 0.379 | 0.163 | 0.059 | 0.698 | 0.020 | 0.144 | 34.487 | 182 | 119 |
| SFO | 0.398 | 0.262 | -0.116 | 0.912 | 0.129 | 0.330 | 73.454 | 182 | 119 |
| SLF | 0.108 | 0.122 | -0.130 | 0.346 | 0.374 | 0.523 | 0.000 | 182 | 119 |
| SS | 0.212 | 0.123 | -0.028 | 0.452 | 0.084 | 0.330 | 0.000 | 182 | 119 |
| UNC | 0.192 | 0.122 | -0.047 | 0.431 | 0.115 | 0.330 | 0.000 | 182 | 119 |
| AverageMD | 0.206 | 0.140 | -0.068 | 0.480 | 0.140 | 0.330 | 0.000 | 152 | 85 |

Table S131. Cohen’s d effect sizes after meta-analysis, for RD differences between patients and controls **between age 30 and 40**. Age, sex, agexsex, age^2^, age^2^xsex and scansite included as covariates.

| Region | Cohen’s d | SE | CI LB | CI UB | P-value | FDR P-value | I^2^ | Controls | Patients |
| --- | --- | --- | --- | --- | --- | --- | --- | --- | --- |
| ACR | 0.248 | 0.173 | -0.090 | 0.586 | 0.151 | 0.258 | 41.358 | 182 | 119 |
| ALIC | 0.275 | 0.123 | 0.034 | 0.516 | 0.025 | 0.156 | 0.002 | 182 | 119 |
| BCC | 0.175 | 0.122 | -0.064 | 0.414 | 0.152 | 0.258 | 0.000 | 182 | 119 |
| CC | 0.189 | 0.122 | -0.050 | 0.428 | 0.121 | 0.258 | 0.000 | 182 | 119 |
| CGC | 0.166 | 0.122 | -0.073 | 0.405 | 0.175 | 0.258 | 0.000 | 182 | 119 |
| CGH | 0.092 | 0.122 | -0.147 | 0.330 | 0.451 | 0.513 | 0.000 | 182 | 119 |
| CR | 0.292 | 0.123 | 0.051 | 0.533 | 0.017 | 0.142 | 0.004 | 182 | 119 |
| CST | -0.023 | 0.279 | -0.569 | 0.523 | 0.934 | 0.934 | 77.039 | 182 | 119 |
| EC | 0.177 | 0.122 | -0.062 | 0.416 | 0.147 | 0.258 | 0.000 | 182 | 119 |
| FX | -0.053 | 0.122 | -0.292 | 0.186 | 0.662 | 0.690 | 0.000 | 182 | 119 |
| FXST | 0.439 | 0.233 | -0.018 | 0.896 | 0.060 | 0.244 | 66.623 | 182 | 119 |
| GCC | 0.162 | 0.122 | -0.078 | 0.401 | 0.186 | 0.258 | 0.000 | 182 | 119 |
| IC | 0.180 | 0.122 | -0.059 | 0.419 | 0.141 | 0.258 | 0.000 | 182 | 119 |
| IFO | 0.231 | 0.141 | -0.045 | 0.508 | 0.100 | 0.258 | 17.882 | 182 | 119 |
| PCR | 0.347 | 0.122 | 0.107 | 0.586 | 0.005 | 0.125 | 0.000 | 182 | 119 |
| PLIC | 0.123 | 0.230 | -0.328 | 0.573 | 0.593 | 0.645 | 66.478 | 182 | 119 |
| PTR | 0.313 | 0.232 | -0.141 | 0.767 | 0.176 | 0.258 | 66.493 | 182 | 119 |
| RLIC | 0.096 | 0.122 | -0.142 | 0.334 | 0.431 | 0.513 | 0.000 | 182 | 119 |
| SCC | 0.165 | 0.122 | -0.074 | 0.404 | 0.175 | 0.258 | 0.000 | 182 | 119 |
| SCR | 0.310 | 0.123 | 0.068 | 0.551 | 0.012 | 0.142 | 0.000 | 182 | 119 |
| SFO | 0.353 | 0.195 | -0.030 | 0.736 | 0.071 | 0.244 | 52.635 | 182 | 119 |
| SLF | 0.117 | 0.122 | -0.121 | 0.356 | 0.335 | 0.419 | 0.000 | 182 | 119 |
| SS | 0.131 | 0.122 | -0.109 | 0.370 | 0.284 | 0.374 | 0.003 | 182 | 119 |
| UNC | 0.251 | 0.122 | 0.011 | 0.490 | 0.040 | 0.200 | 0.000 | 182 | 119 |
| AverageRD | 0.247 | 0.140 | -0.027 | 0.520 | 0.078 | 0.244 | 0.000 | 152 | 85 |

Table S132. Cohen’s d effect sizes after meta-analysis, for FA differences between patients and controls **between age 40 and 50**. Age, sex, agexsex, age^2^, age^2^xsex and scansite included as covariates.

| Region | Cohen’s d | SE | CI LB | CI UB | P-value | FDR P-value | I^2^ | Controls | Patients |
| --- | --- | --- | --- | --- | --- | --- | --- | --- | --- |
| ACR | -0.246 | 0.268 | -0.772 | 0.279 | 0.358 | 0.613 | 73.308 | 164 | 100 |
| ALIC | -0.378 | 0.176 | -0.723 | -0.033 | 0.032 | 0.295 | 38.502 | 164 | 100 |
| BCC | -0.231 | 0.394 | -1.003 | 0.541 | 0.557 | 0.771 | 87.429 | 164 | 100 |
| CC | -0.237 | 0.368 | -0.958 | 0.484 | 0.520 | 0.765 | 85.646 | 164 | 100 |
| CGC | 0.028 | 0.134 | -0.235 | 0.291 | 0.834 | 0.899 | 0.000 | 164 | 100 |
| CGH | -0.057 | 0.134 | -0.320 | 0.206 | 0.673 | 0.789 | 0.000 | 164 | 100 |
| CR | -0.279 | 0.273 | -0.814 | 0.256 | 0.307 | 0.613 | 74.183 | 164 | 100 |
| CST | -0.136 | 0.141 | -0.413 | 0.141 | 0.337 | 0.613 | 8.868 | 164 | 100 |
| EC | 0.025 | 0.143 | -0.255 | 0.304 | 0.863 | 0.899 | 10.048 | 164 | 100 |
| FX | -0.014 | 0.252 | -0.507 | 0.479 | 0.955 | 0.955 | 70.109 | 164 | 100 |
| FXST | -0.332 | 0.225 | -0.773 | 0.108 | 0.139 | 0.434 | 62.053 | 164 | 100 |
| GCC | -0.153 | 0.315 | -0.770 | 0.464 | 0.627 | 0.784 | 80.635 | 164 | 100 |
| IC | -0.455 | 0.240 | -0.925 | 0.015 | 0.058 | 0.295 | 66.119 | 164 | 100 |
| IFO | -0.053 | 0.134 | -0.316 | 0.210 | 0.694 | 0.789 | 0.000 | 164 | 100 |
| PCR | -0.153 | 0.135 | -0.417 | 0.111 | 0.256 | 0.613 | 0.000 | 164 | 100 |
| PLIC | -0.413 | 0.251 | -0.905 | 0.078 | 0.099 | 0.354 | 69.154 | 164 | 100 |
| PTR | -0.416 | 0.136 | -0.682 | -0.150 | 0.002 | 0.050 | 0.001 | 164 | 100 |
| RLIC | -0.358 | 0.213 | -0.775 | 0.058 | 0.092 | 0.354 | 57.554 | 164 | 100 |
| SCC | -0.218 | 0.178 | -0.567 | 0.131 | 0.222 | 0.613 | 40.624 | 164 | 100 |
| SCR | -0.322 | 0.331 | -0.972 | 0.328 | 0.332 | 0.613 | 82.316 | 164 | 100 |
| SFO | -0.183 | 0.214 | -0.602 | 0.236 | 0.392 | 0.613 | 58.498 | 164 | 100 |
| SLF | -0.521 | 0.271 | -1.052 | 0.010 | 0.055 | 0.295 | 73.160 | 164 | 100 |
| SS | -0.254 | 0.135 | -0.519 | 0.010 | 0.059 | 0.295 | 0.000 | 164 | 100 |
| UNC | -0.073 | 0.135 | -0.337 | 0.190 | 0.586 | 0.771 | 0.014 | 164 | 100 |
| AverageFA | -0.134 | 0.153 | -0.434 | 0.167 | 0.383 | 0.613 | 0.000 | 142 | 69 |

Table S133. Cohen’s d effect sizes after meta-analysis, for AD differences between patients and controls **between age 40 and 50**. Age, sex, agexsex, age^2^, age^2^xsex and scansite included as covariates.

| Region | Cohen’s d | SE | CI LB | CI UB | P-value | FDR P-value | I^2^ | Controls | Patients |
| --- | --- | --- | --- | --- | --- | --- | --- | --- | --- |
| ACR | 0.381 | 0.315 | -0.236 | 0.998 | 0.226 | 0.682 | 80.335 | 164 | 100 |
| ALIC | 0.283 | 0.341 | -0.385 | 0.952 | 0.406 | 0.693 | 83.327 | 164 | 100 |
| BCC | 0.249 | 0.314 | -0.366 | 0.863 | 0.428 | 0.693 | 80.377 | 164 | 100 |
| CC | 0.184 | 0.391 | -0.582 | 0.951 | 0.638 | 0.693 | 87.316 | 164 | 100 |
| CGC | 0.359 | 0.336 | -0.299 | 1.017 | 0.285 | 0.682 | 82.677 | 164 | 100 |
| CGH | 0.209 | 0.154 | -0.094 | 0.511 | 0.176 | 0.682 | 22.086 | 164 | 100 |
| CR | 0.353 | 0.308 | -0.251 | 0.957 | 0.253 | 0.682 | 79.548 | 164 | 100 |
| CST | 0.383 | 0.143 | 0.104 | 0.662 | 0.007 | 0.175 | 8.505 | 164 | 100 |
| EC | 0.205 | 0.358 | -0.497 | 0.907 | 0.567 | 0.693 | 84.909 | 164 | 100 |
| FX | 0.219 | 0.332 | -0.431 | 0.870 | 0.509 | 0.693 | 82.506 | 164 | 100 |
| FXST | 0.236 | 0.475 | -0.696 | 1.168 | 0.620 | 0.693 | 91.237 | 164 | 100 |
| GCC | 0.221 | 0.433 | -0.628 | 1.071 | 0.610 | 0.693 | 89.600 | 164 | 100 |
| IC | 0.336 | 0.324 | -0.299 | 0.971 | 0.300 | 0.682 | 81.493 | 164 | 100 |
| IFO | 0.335 | 0.311 | -0.273 | 0.944 | 0.280 | 0.682 | 79.874 | 164 | 100 |
| PCR | 0.290 | 0.369 | -0.433 | 1.013 | 0.432 | 0.693 | 85.683 | 164 | 100 |
| PLIC | 0.428 | 0.268 | -0.097 | 0.953 | 0.110 | 0.682 | 72.807 | 164 | 100 |
| PTR | 0.238 | 0.425 | -0.594 | 1.071 | 0.575 | 0.693 | 89.163 | 164 | 100 |
| RLIC | 0.213 | 0.425 | -0.620 | 1.046 | 0.617 | 0.693 | 89.156 | 164 | 100 |
| SCC | 0.151 | 0.420 | -0.672 | 0.973 | 0.719 | 0.749 | 88.930 | 164 | 100 |
| SCR | 0.334 | 0.277 | -0.209 | 0.878 | 0.228 | 0.682 | 74.856 | 164 | 100 |
| SFO | 0.218 | 0.342 | -0.452 | 0.888 | 0.524 | 0.693 | 83.482 | 164 | 100 |
| SLF | 0.225 | 0.391 | -0.541 | 0.990 | 0.565 | 0.693 | 87.236 | 164 | 100 |
| SS | 0.404 | 0.331 | -0.245 | 1.054 | 0.222 | 0.682 | 82.187 | 164 | 100 |
| UNC | 0.430 | 0.278 | -0.115 | 0.974 | 0.122 | 0.682 | 74.732 | 164 | 100 |
| AverageAD | -0.026 | 0.153 | -0.326 | 0.274 | 0.864 | 0.864 | 0.000 | 142 | 69 |

Table S134. Cohen’s d effect sizes after meta-analysis, for MD differences between patients and controls **between age 40 and 50**. Age, sex, agexsex, age^2^, age^2^xsex and scansite included as covariates.

| Region | Cohen’s d | SE | CI LB | CI UB | P-value | FDR P-value | I^2^ | Controls | Patients |
| --- | --- | --- | --- | --- | --- | --- | --- | --- | --- |
| ACR | 0.384 | 0.268 | -0.140 | 0.909 | 0.151 | 0.296 | 72.897 | 164 | 100 |
| ALIC | 0.309 | 0.171 | -0.025 | 0.644 | 0.070 | 0.254 | 35.171 | 164 | 100 |
| BCC | 0.312 | 0.286 | -0.250 | 0.873 | 0.277 | 0.407 | 76.469 | 164 | 100 |
| CC | 0.264 | 0.285 | -0.295 | 0.823 | 0.355 | 0.467 | 76.340 | 164 | 100 |
| CGC | 0.357 | 0.297 | -0.225 | 0.938 | 0.229 | 0.358 | 77.921 | 164 | 100 |
| CGH | 0.165 | 0.135 | -0.098 | 0.429 | 0.219 | 0.358 | 0.000 | 164 | 100 |
| CR | 0.421 | 0.243 | -0.055 | 0.897 | 0.083 | 0.259 | 67.046 | 164 | 100 |
| CST | 0.263 | 0.135 | -0.001 | 0.527 | 0.051 | 0.254 | 0.000 | 164 | 100 |
| EC | 0.244 | 0.263 | -0.272 | 0.760 | 0.354 | 0.467 | 72.364 | 164 | 100 |
| FX | 0.235 | 0.316 | -0.385 | 0.854 | 0.458 | 0.484 | 80.735 | 164 | 100 |
| FXST | 0.196 | 0.241 | -0.277 | 0.668 | 0.417 | 0.484 | 67.220 | 164 | 100 |
| GCC | 0.187 | 0.290 | -0.381 | 0.756 | 0.518 | 0.518 | 77.255 | 164 | 100 |
| IC | 0.333 | 0.213 | -0.085 | 0.751 | 0.119 | 0.280 | 57.911 | 164 | 100 |
| IFO | 0.210 | 0.169 | -0.122 | 0.542 | 0.215 | 0.358 | 34.614 | 164 | 100 |
| PCR | 0.367 | 0.203 | -0.031 | 0.766 | 0.071 | 0.254 | 53.660 | 164 | 100 |
| PLIC | 0.381 | 0.247 | -0.103 | 0.866 | 0.123 | 0.280 | 68.340 | 164 | 100 |
| PTR | 0.335 | 0.135 | 0.070 | 0.600 | 0.013 | 0.254 | 0.000 | 164 | 100 |
| RLIC | 0.170 | 0.233 | -0.286 | 0.626 | 0.465 | 0.484 | 64.852 | 164 | 100 |
| SCC | 0.228 | 0.262 | -0.285 | 0.741 | 0.385 | 0.481 | 72.057 | 164 | 100 |
| SCR | 0.478 | 0.236 | 0.016 | 0.939 | 0.043 | 0.254 | 64.857 | 164 | 100 |
| SFO | 0.388 | 0.193 | 0.008 | 0.767 | 0.045 | 0.254 | 48.797 | 164 | 100 |
| SLF | 0.374 | 0.224 | -0.066 | 0.813 | 0.095 | 0.264 | 61.632 | 164 | 100 |
| SS | 0.323 | 0.227 | -0.121 | 0.767 | 0.154 | 0.296 | 62.650 | 164 | 100 |
| UNC | 0.319 | 0.157 | 0.011 | 0.626 | 0.042 | 0.254 | 23.753 | 164 | 100 |
| AverageMD | 0.116 | 0.153 | -0.184 | 0.416 | 0.449 | 0.484 | 0.000 | 142 | 69 |

Table S135. Cohen’s d effect sizes after meta-analysis, for RD differences between patients and controls **between age 40 and 50**. Age, sex, agexsex, age^2^, age^2^xsex and scansite included as covariates.

| Region | Cohen’s d | SE | CI LB | CI UB | P-value | FDR P-value | I^2^ | Controls | Patients |
| --- | --- | --- | --- | --- | --- | --- | --- | --- | --- |
| ACR | 0.235 | 0.156 | -0.071 | 0.541 | 0.132 | 0.419 | 23.549 | 164 | 100 |
| ALIC | 0.192 | 0.134 | -0.072 | 0.455 | 0.154 | 0.428 | 0.000 | 164 | 100 |
| BCC | 0.153 | 0.220 | -0.279 | 0.584 | 0.488 | 0.718 | 60.857 | 164 | 100 |
| CC | -0.053 | 0.134 | -0.316 | 0.210 | 0.695 | 0.827 | 0.000 | 164 | 100 |
| CGC | 0.206 | 0.212 | -0.210 | 0.621 | 0.332 | 0.692 | 57.757 | 164 | 100 |
| CGH | 0.097 | 0.134 | -0.167 | 0.360 | 0.472 | 0.718 | 0.000 | 164 | 100 |
| CR | 0.269 | 0.139 | -0.002 | 0.541 | 0.052 | 0.410 | 4.407 | 164 | 100 |
| CST | 0.222 | 0.135 | -0.042 | 0.486 | 0.099 | 0.413 | 0.000 | 164 | 100 |
| EC | 0.152 | 0.135 | -0.113 | 0.417 | 0.262 | 0.655 | 0.721 | 164 | 100 |
| FX | 0.238 | 0.302 | -0.354 | 0.830 | 0.431 | 0.718 | 78.926 | 164 | 100 |
| FXST | 0.084 | 0.143 | -0.195 | 0.364 | 0.556 | 0.772 | 10.195 | 164 | 100 |
| GCC | -0.202 | 0.135 | -0.466 | 0.062 | 0.134 | 0.419 | 0.000 | 164 | 100 |
| IC | 0.028 | 0.212 | -0.389 | 0.444 | 0.897 | 0.897 | 58.222 | 164 | 100 |
| IFO | -0.035 | 0.134 | -0.298 | 0.228 | 0.794 | 0.827 | 0.000 | 164 | 100 |
| PCR | 0.135 | 0.135 | -0.128 | 0.399 | 0.315 | 0.692 | 0.000 | 164 | 100 |
| PLIC | -0.114 | 0.332 | -0.765 | 0.536 | 0.730 | 0.827 | 82.542 | 164 | 100 |
| PTR | 0.073 | 0.267 | -0.450 | 0.597 | 0.784 | 0.827 | 73.370 | 164 | 100 |
| RLIC | -0.053 | 0.176 | -0.398 | 0.292 | 0.764 | 0.827 | 39.715 | 164 | 100 |
| SCC | -0.220 | 0.313 | -0.834 | 0.394 | 0.482 | 0.718 | 80.375 | 164 | 100 |
| SCR | 0.392 | 0.204 | -0.008 | 0.791 | 0.055 | 0.410 | 53.733 | 164 | 100 |
| SFO | 0.270 | 0.154 | -0.031 | 0.572 | 0.079 | 0.410 | 21.467 | 164 | 100 |
| SLF | 0.271 | 0.135 | 0.007 | 0.535 | 0.045 | 0.410 | 0.000 | 164 | 100 |
| SS | 0.234 | 0.135 | -0.030 | 0.498 | 0.082 | 0.410 | 0.000 | 164 | 100 |
| UNC | 0.061 | 0.134 | -0.202 | 0.324 | 0.651 | 0.827 | 0.000 | 164 | 100 |
| AverageRD | 0.116 | 0.153 | -0.184 | 0.417 | 0.447 | 0.718 | 0.000 | 142 | 69 |

Table S136. Cohen’s d effect sizes after meta-analysis, for FA differences between patients and controls **between age 50 and 60**. Age, sex, agexsex, age^2^, age^2^xsex and scansite included as covariates.

| Region | Cohen’s d | SE | CI LB | CI UB | P-value | FDR P-value | I^2^ | Controls | Patients |
| --- | --- | --- | --- | --- | --- | --- | --- | --- | --- |
| ACR | -0.434 | 0.326 | -1.072 | 0.204 | 0.182 | 0.545 | 85.264 | 171 | 124 |
| ALIC | -0.249 | 0.368 | -0.970 | 0.473 | 0.499 | 0.693 | 88.556 | 171 | 124 |
| BCC | -0.346 | 0.270 | -0.874 | 0.182 | 0.199 | 0.545 | 78.830 | 171 | 124 |
| CC | -0.355 | 0.301 | -0.944 | 0.234 | 0.238 | 0.545 | 82.901 | 171 | 124 |
| CGC | -0.239 | 0.158 | -0.550 | 0.071 | 0.131 | 0.545 | 39.886 | 171 | 124 |
| CGH | -0.057 | 0.134 | -0.320 | 0.205 | 0.668 | 0.793 | 16.456 | 171 | 124 |
| CR | -0.460 | 0.338 | -1.122 | 0.203 | 0.174 | 0.545 | 86.265 | 171 | 124 |
| CST | 0.087 | 0.219 | -0.343 | 0.516 | 0.692 | 0.793 | 68.626 | 171 | 124 |
| EC | -0.144 | 0.122 | -0.383 | 0.096 | 0.240 | 0.545 | 0.000 | 171 | 124 |
| FX | 0.047 | 0.122 | -0.193 | 0.286 | 0.702 | 0.793 | 0.000 | 171 | 124 |
| FXST | 0.044 | 0.128 | -0.207 | 0.296 | 0.730 | 0.793 | 9.332 | 171 | 124 |
| GCC | -0.548 | 0.231 | -1.001 | -0.096 | 0.018 | 0.450 | 70.689 | 171 | 124 |
| IC | -0.260 | 0.365 | -0.976 | 0.455 | 0.476 | 0.693 | 88.373 | 171 | 124 |
| IFO | -0.133 | 0.122 | -0.373 | 0.107 | 0.277 | 0.560 | 0.000 | 171 | 124 |
| PCR | -0.436 | 0.332 | -1.086 | 0.215 | 0.189 | 0.545 | 85.799 | 171 | 124 |
| PLIC | -0.264 | 0.377 | -1.002 | 0.473 | 0.482 | 0.693 | 89.033 | 171 | 124 |
| PTR | -0.230 | 0.174 | -0.572 | 0.111 | 0.186 | 0.545 | 50.253 | 171 | 124 |
| RLIC | -0.247 | 0.261 | -0.760 | 0.265 | 0.344 | 0.614 | 77.656 | 171 | 124 |
| SCC | -0.016 | 0.312 | -0.627 | 0.595 | 0.959 | 0.959 | 84.317 | 171 | 124 |
| SCR | -0.402 | 0.288 | -0.966 | 0.163 | 0.163 | 0.545 | 81.328 | 171 | 124 |
| SFO | -0.184 | 0.174 | -0.525 | 0.158 | 0.291 | 0.560 | 50.268 | 171 | 124 |
| SLF | -0.284 | 0.403 | -1.075 | 0.506 | 0.481 | 0.693 | 90.383 | 171 | 124 |
| SS | -0.734 | 0.351 | -1.422 | -0.045 | 0.037 | 0.463 | 86.784 | 171 | 124 |
| UNC | -0.172 | 0.437 | -1.028 | 0.685 | 0.694 | 0.793 | 91.798 | 171 | 124 |
| AverageFA | -0.042 | 0.180 | -0.395 | 0.312 | 0.818 | 0.852 | 31.084 | 123 | 82 |

Table S137. Cohen’s d effect sizes after meta-analysis, for AD differences between patients and controls **between age 50 and 60**. Age, sex, agexsex, age^2^, age^2^xsex and scansite included as covariates.

| Region | Cohen’s d | SE | CI LB | CI UB | P-value | FDR P-value | I^2^ | Controls | Patients |
| --- | --- | --- | --- | --- | --- | --- | --- | --- | --- |
| ACR | 0.505 | 0.203 | 0.107 | 0.903 | 0.013 | 0.206 | 62.487 | 171 | 124 |
| ALIC | 0.377 | 0.299 | -0.210 | 0.964 | 0.208 | 0.400 | 82.715 | 171 | 124 |
| BCC | 0.482 | 0.319 | -0.142 | 1.107 | 0.130 | 0.325 | 84.529 | 171 | 124 |
| CC | 0.438 | 0.308 | -0.166 | 1.043 | 0.155 | 0.352 | 83.581 | 171 | 124 |
| CGC | 0.480 | 0.225 | 0.040 | 0.921 | 0.033 | 0.206 | 69.294 | 171 | 124 |
| CGH | 0.444 | 0.293 | -0.130 | 1.018 | 0.130 | 0.325 | 81.860 | 171 | 124 |
| CR | 0.482 | 0.259 | -0.027 | 0.990 | 0.063 | 0.315 | 76.871 | 171 | 124 |
| CST | 0.349 | 0.407 | -0.449 | 1.146 | 0.391 | 0.444 | 90.508 | 171 | 124 |
| EC | 0.473 | 0.222 | 0.038 | 0.908 | 0.033 | 0.206 | 68.570 | 171 | 124 |
| FX | 0.247 | 0.273 | -0.289 | 0.783 | 0.366 | 0.444 | 79.521 | 171 | 124 |
| FXST | 0.338 | 0.337 | -0.322 | 0.998 | 0.316 | 0.436 | 86.321 | 171 | 124 |
| GCC | 0.394 | 0.261 | -0.117 | 0.906 | 0.130 | 0.325 | 77.299 | 171 | 124 |
| IC | 0.367 | 0.378 | -0.373 | 1.108 | 0.331 | 0.436 | 89.033 | 171 | 124 |
| IFO | 0.390 | 0.171 | 0.056 | 0.725 | 0.022 | 0.206 | 47.633 | 171 | 124 |
| PCR | 0.341 | 0.307 | -0.261 | 0.942 | 0.267 | 0.415 | 83.586 | 171 | 124 |
| PLIC | 0.393 | 0.366 | -0.324 | 1.111 | 0.282 | 0.415 | 88.309 | 171 | 124 |
| PTR | 0.231 | 0.329 | -0.414 | 0.877 | 0.482 | 0.482 | 85.791 | 171 | 124 |
| RLIC | 0.304 | 0.407 | -0.493 | 1.101 | 0.454 | 0.482 | 90.538 | 171 | 124 |
| SCC | 0.321 | 0.371 | -0.405 | 1.048 | 0.386 | 0.444 | 88.652 | 171 | 124 |
| SCR | 0.468 | 0.296 | -0.111 | 1.048 | 0.113 | 0.325 | 82.157 | 171 | 124 |
| SFO | 0.498 | 0.303 | -0.096 | 1.092 | 0.100 | 0.325 | 82.948 | 171 | 124 |
| SLF | 0.257 | 0.352 | -0.432 | 0.946 | 0.465 | 0.482 | 87.477 | 171 | 124 |
| SS | 0.344 | 0.295 | -0.235 | 0.922 | 0.244 | 0.415 | 82.278 | 171 | 124 |
| UNC | 0.399 | 0.298 | -0.184 | 0.982 | 0.180 | 0.375 | 82.462 | 171 | 124 |
| AverageAD | 0.225 | 0.202 | -0.172 | 0.622 | 0.266 | 0.415 | 44.936 | 123 | 82 |

Table S138. Cohen’s d effect sizes after meta-analysis, for MD differences between patients and controls **between age 50 and 60**. Age, sex, agexsex, age^2^, age^2^xsex and scansite included as covariates.

| Region | Cohen’s d | SE | CI LB | CI UB | P-value | FDR P-value | I^2^ | Controls | Patients |
| --- | --- | --- | --- | --- | --- | --- | --- | --- | --- |
| ACR | 0.399 | 0.183 | 0.040 | 0.757 | 0.029 | 0.170 | 54.277 | 171 | 124 |
| ALIC | 0.265 | 0.323 | -0.368 | 0.897 | 0.412 | 0.626 | 85.192 | 171 | 124 |
| BCC | 0.482 | 0.284 | -0.074 | 1.039 | 0.089 | 0.298 | 80.627 | 171 | 124 |
| CC | 0.423 | 0.265 | -0.097 | 0.942 | 0.111 | 0.298 | 77.953 | 171 | 124 |
| CGC | 0.472 | 0.206 | 0.069 | 0.875 | 0.022 | 0.170 | 63.412 | 171 | 124 |
| CGH | 0.378 | 0.258 | -0.127 | 0.884 | 0.142 | 0.323 | 76.836 | 171 | 124 |
| CR | 0.396 | 0.225 | -0.045 | 0.837 | 0.079 | 0.298 | 69.664 | 171 | 124 |
| CST | 0.087 | 0.342 | -0.584 | 0.757 | 0.800 | 0.800 | 86.885 | 171 | 124 |
| EC | 0.505 | 0.169 | 0.174 | 0.837 | 0.003 | 0.038 | 45.786 | 171 | 124 |
| FX | 0.193 | 0.254 | -0.306 | 0.692 | 0.448 | 0.626 | 76.497 | 171 | 124 |
| FXST | 0.325 | 0.285 | -0.233 | 0.883 | 0.253 | 0.487 | 81.010 | 171 | 124 |
| GCC | 0.389 | 0.130 | 0.134 | 0.644 | 0.003 | 0.038 | 10.141 | 171 | 124 |
| IC | 0.180 | 0.339 | -0.484 | 0.844 | 0.595 | 0.729 | 86.587 | 171 | 124 |
| IFO | 0.044 | 0.122 | -0.196 | 0.283 | 0.721 | 0.779 | 0.000 | 171 | 124 |
| PCR | 0.256 | 0.220 | -0.176 | 0.687 | 0.245 | 0.487 | 68.639 | 171 | 124 |
| PLIC | 0.131 | 0.349 | -0.553 | 0.815 | 0.707 | 0.779 | 87.385 | 171 | 124 |
| PTR | 0.117 | 0.178 | -0.233 | 0.466 | 0.513 | 0.675 | 52.717 | 171 | 124 |
| RLIC | 0.224 | 0.297 | -0.359 | 0.807 | 0.451 | 0.626 | 82.685 | 171 | 124 |
| SCC | 0.163 | 0.322 | -0.468 | 0.794 | 0.612 | 0.729 | 85.202 | 171 | 124 |
| SCR | 0.413 | 0.265 | -0.106 | 0.933 | 0.119 | 0.298 | 77.990 | 171 | 124 |
| SFO | 0.354 | 0.218 | -0.074 | 0.782 | 0.105 | 0.298 | 67.927 | 171 | 124 |
| SLF | 0.306 | 0.363 | -0.405 | 1.018 | 0.399 | 0.626 | 88.196 | 171 | 124 |
| SS | 0.362 | 0.171 | 0.027 | 0.697 | 0.034 | 0.170 | 47.787 | 171 | 124 |
| UNC | 0.059 | 0.184 | -0.301 | 0.419 | 0.748 | 0.779 | 55.457 | 171 | 124 |
| AverageMD | 0.190 | 0.188 | -0.179 | 0.559 | 0.312 | 0.557 | 36.332 | 123 | 82 |

Table S139. Cohen’s d effect sizes after meta-analysis, for RD differences between patients and controls **between age 50 and 60**. Age, sex, agexsex, age^2^, age^2^xsex and scansite included as covariates.

| Region | Cohen’s d | SE | CI LB | CI UB | P-value | FDR P-value | I^2^ | Controls | Patients |
| --- | --- | --- | --- | --- | --- | --- | --- | --- | --- |
| ACR | 0.247 | 0.126 | -0.001 | 0.494 | 0.050 | 0.222 | 5.420 | 171 | 124 |
| ALIC | 0.119 | 0.193 | -0.258 | 0.497 | 0.536 | 0.705 | 59.403 | 171 | 124 |
| BCC | 0.286 | 0.164 | -0.035 | 0.608 | 0.081 | 0.225 | 43.709 | 171 | 124 |
| CC | 0.043 | 0.122 | -0.196 | 0.283 | 0.724 | 0.787 | 0.012 | 171 | 124 |
| CGC | 0.280 | 0.125 | 0.035 | 0.524 | 0.025 | 0.222 | 3.048 | 171 | 124 |
| CGH | 0.304 | 0.142 | 0.025 | 0.582 | 0.033 | 0.222 | 25.103 | 171 | 124 |
| CR | 0.221 | 0.137 | -0.047 | 0.490 | 0.107 | 0.243 | 19.942 | 171 | 124 |
| CST | 0.012 | 0.202 | -0.383 | 0.407 | 0.951 | 0.991 | 62.969 | 171 | 124 |
| EC | 0.450 | 0.133 | 0.188 | 0.712 | 0.001 | 0.025 | 13.899 | 171 | 124 |
| FX | 0.157 | 0.238 | -0.310 | 0.624 | 0.510 | 0.705 | 73.304 | 171 | 124 |
| FXST | 0.244 | 0.169 | -0.086 | 0.575 | 0.147 | 0.306 | 46.880 | 171 | 124 |
| GCC | 0.095 | 0.252 | -0.400 | 0.589 | 0.708 | 0.787 | 76.241 | 171 | 124 |
| IC | -0.092 | 0.122 | -0.332 | 0.147 | 0.450 | 0.703 | 0.000 | 171 | 124 |
| IFO | -0.135 | 0.213 | -0.552 | 0.283 | 0.528 | 0.705 | 66.780 | 171 | 124 |
| PCR | 0.001 | 0.154 | -0.299 | 0.302 | 0.993 | 0.993 | 36.489 | 171 | 124 |
| PLIC | -0.275 | 0.151 | -0.571 | 0.021 | 0.068 | 0.222 | 33.631 | 171 | 124 |
| PTR | -0.173 | 0.184 | -0.534 | 0.188 | 0.348 | 0.588 | 55.615 | 171 | 124 |
| RLIC | -0.048 | 0.122 | -0.288 | 0.191 | 0.694 | 0.787 | 0.000 | 171 | 124 |
| SCC | -0.438 | 0.208 | -0.846 | -0.029 | 0.036 | 0.222 | 64.461 | 171 | 124 |
| SCR | 0.279 | 0.154 | -0.024 | 0.582 | 0.071 | 0.222 | 36.622 | 171 | 124 |
| SFO | 0.114 | 0.122 | -0.126 | 0.353 | 0.353 | 0.588 | 0.000 | 171 | 124 |
| SLF | 0.105 | 0.236 | -0.357 | 0.567 | 0.657 | 0.787 | 72.803 | 171 | 124 |
| SS | 0.258 | 0.135 | -0.005 | 0.522 | 0.055 | 0.222 | 16.932 | 171 | 124 |
| UNC | -0.251 | 0.155 | -0.554 | 0.052 | 0.105 | 0.243 | 36.850 | 171 | 124 |
| AverageRD | 0.170 | 0.173 | -0.170 | 0.510 | 0.327 | 0.588 | 25.144 | 123 | 82 |

Table S140. Cohen’s d effect sizes after meta-analysis, for FA differences between patients and controls **in the age range of UK biobank (>42 years of age)**. Age, sex, agexsex, age^2^, age^2^xsex and scansite included as covariates.

| Region | Cohen’s d | SE | CI LB | CI UB | P-value | FDR P-value | I^2^ | Controls | Patients |
| --- | --- | --- | --- | --- | --- | --- | --- | --- | --- |
| ACR | -0.391 | 0.176 | -0.736 | -0.046 | 0.026 | 0.102 | 77.477 | 391 | 348 |
| ALIC | -0.364 | 0.177 | -0.711 | -0.016 | 0.040 | 0.102 | 77.842 | 391 | 348 |
| BCC | -0.316 | 0.159 | -0.628 | -0.004 | 0.047 | 0.102 | 72.615 | 391 | 348 |
| CC | -0.318 | 0.162 | -0.636 | -0.001 | 0.049 | 0.102 | 73.482 | 391 | 348 |
| CGC | -0.230 | 0.091 | -0.409 | -0.052 | 0.012 | 0.095 | 21.851 | 391 | 348 |
| CGH | -0.093 | 0.085 | -0.260 | 0.074 | 0.274 | 0.361 | 13.135 | 391 | 348 |
| CR | -0.344 | 0.173 | -0.682 | -0.005 | 0.047 | 0.102 | 76.708 | 391 | 348 |
| CST | -0.069 | 0.112 | -0.289 | 0.151 | 0.539 | 0.561 | 45.906 | 391 | 348 |
| EC | -0.186 | 0.148 | -0.476 | 0.105 | 0.210 | 0.339 | 68.604 | 391 | 348 |
| FX | -0.031 | 0.090 | -0.207 | 0.146 | 0.735 | 0.735 | 20.707 | 391 | 348 |
| FXST | -0.101 | 0.100 | -0.297 | 0.096 | 0.316 | 0.376 | 33.754 | 391 | 348 |
| GCC | -0.333 | 0.163 | -0.651 | -0.014 | 0.041 | 0.102 | 73.665 | 391 | 348 |
| IC | -0.392 | 0.168 | -0.721 | -0.064 | 0.019 | 0.095 | 75.200 | 391 | 348 |
| IFO | -0.082 | 0.078 | -0.234 | 0.070 | 0.292 | 0.365 | 0.012 | 391 | 348 |
| PCR | -0.283 | 0.140 | -0.558 | -0.008 | 0.044 | 0.102 | 64.714 | 391 | 348 |
| PLIC | -0.314 | 0.177 | -0.660 | 0.033 | 0.076 | 0.136 | 77.781 | 391 | 348 |
| PTR | -0.347 | 0.109 | -0.560 | -0.134 | 0.001 | 0.013 | 41.922 | 391 | 348 |
| RLIC | -0.353 | 0.146 | -0.640 | -0.067 | 0.016 | 0.095 | 67.365 | 391 | 348 |
| SCC | -0.146 | 0.123 | -0.387 | 0.095 | 0.235 | 0.346 | 54.781 | 391 | 348 |
| SCR | -0.224 | 0.182 | -0.580 | 0.132 | 0.217 | 0.339 | 79.097 | 391 | 348 |
| SFO | -0.137 | 0.120 | -0.371 | 0.098 | 0.253 | 0.351 | 52.122 | 391 | 348 |
| SLF | -0.348 | 0.183 | -0.707 | 0.010 | 0.057 | 0.110 | 79.231 | 391 | 348 |
| SS | -0.575 | 0.153 | -0.874 | -0.275 | 0.000 | 0.001 | 69.388 | 391 | 348 |
| UNC | -0.139 | 0.222 | -0.574 | 0.296 | 0.532 | 0.561 | 86.088 | 391 | 348 |
| AverageFA | -0.144 | 0.161 | -0.460 | 0.172 | 0.373 | 0.424 | 0.000 | 158 | 51 |

Table S141. Cohen’s d effect sizes after meta-analysis, for AD differences between patients and controls **in the age range of UK biobank (>42 years of age)**. Age, sex, agexsex, age^2^, age^2^xsex and scansite included as covariates.

| Region | Cohen’s d | SE | CI LB | CI UB | P-value | FDR P-value | I^2^ | Controls | Patients |
| --- | --- | --- | --- | --- | --- | --- | --- | --- | --- |
| ACR | 0.180 | 0.199 | -0.211 | 0.570 | 0.368 | 0.551 | 82.686 | 391 | 348 |
| ALIC | 0.169 | 0.190 | -0.204 | 0.542 | 0.375 | 0.551 | 80.997 | 391 | 348 |
| BCC | 0.259 | 0.199 | -0.130 | 0.648 | 0.192 | 0.551 | 82.496 | 391 | 348 |
| CC | 0.197 | 0.209 | -0.213 | 0.607 | 0.346 | 0.551 | 84.266 | 391 | 348 |
| CGC | 0.147 | 0.197 | -0.239 | 0.533 | 0.455 | 0.632 | 82.298 | 391 | 348 |
| CGH | 0.159 | 0.178 | -0.189 | 0.507 | 0.370 | 0.551 | 78.184 | 391 | 348 |
| CR | 0.226 | 0.188 | -0.143 | 0.596 | 0.230 | 0.551 | 80.571 | 391 | 348 |
| CST | 0.420 | 0.161 | 0.104 | 0.736 | 0.009 | 0.225 | 73.021 | 391 | 348 |
| EC | 0.100 | 0.229 | -0.350 | 0.549 | 0.664 | 0.755 | 86.946 | 391 | 348 |
| FX | 0.188 | 0.159 | -0.124 | 0.501 | 0.238 | 0.551 | 72.898 | 391 | 348 |
| FXST | 0.205 | 0.197 | -0.180 | 0.590 | 0.297 | 0.551 | 82.178 | 391 | 348 |
| GCC | 0.172 | 0.191 | -0.202 | 0.546 | 0.367 | 0.551 | 81.106 | 391 | 348 |
| IC | 0.124 | 0.213 | -0.294 | 0.541 | 0.561 | 0.689 | 84.845 | 391 | 348 |
| IFO | 0.240 | 0.179 | -0.111 | 0.592 | 0.180 | 0.551 | 78.485 | 391 | 348 |
| PCR | 0.182 | 0.203 | -0.215 | 0.580 | 0.369 | 0.551 | 83.303 | 391 | 348 |
| PLIC | 0.188 | 0.197 | -0.197 | 0.574 | 0.339 | 0.551 | 82.198 | 391 | 348 |
| PTR | 0.046 | 0.227 | -0.398 | 0.490 | 0.838 | 0.886 | 86.673 | 391 | 348 |
| RLIC | 0.044 | 0.237 | -0.420 | 0.509 | 0.851 | 0.886 | 87.797 | 391 | 348 |
| SCC | 0.124 | 0.223 | -0.313 | 0.560 | 0.579 | 0.689 | 86.167 | 391 | 348 |
| SCR | 0.281 | 0.170 | -0.052 | 0.614 | 0.098 | 0.551 | 75.983 | 391 | 348 |
| SFO | 0.223 | 0.192 | -0.153 | 0.598 | 0.245 | 0.551 | 81.190 | 391 | 348 |
| SLF | 0.132 | 0.207 | -0.273 | 0.537 | 0.523 | 0.688 | 83.942 | 391 | 348 |
| SS | -0.020 | 0.255 | -0.520 | 0.480 | 0.937 | 0.937 | 89.495 | 391 | 348 |
| UNC | 0.257 | 0.251 | -0.235 | 0.749 | 0.305 | 0.551 | 89.039 | 391 | 348 |
| AverageAD | 0.260 | 0.162 | -0.057 | 0.577 | 0.107 | 0.551 | 0.000 | 158 | 51 |

Table S142. Cohen’s d effect sizes after meta-analysis, for MD differences between patients and controls **in the age range of UK biobank (>42 years of age)**. Age, sex, agexsex, age^2^, age^2^xsex and scansite included as covariates.

| Region | Cohen’s d | SE | CI LB | CI UB | P-value | FDR P-value | I^2^ | Controls | Patients |
| --- | --- | --- | --- | --- | --- | --- | --- | --- | --- |
| ACR | 0.228 | 0.123 | -0.012 | 0.468 | 0.063 | 0.300 | 54.228 | 391 | 348 |
| ALIC | 0.216 | 0.132 | -0.043 | 0.475 | 0.102 | 0.300 | 60.467 | 391 | 348 |
| BCC | 0.280 | 0.164 | -0.042 | 0.601 | 0.088 | 0.300 | 74.225 | 391 | 348 |
| CC | 0.234 | 0.152 | -0.063 | 0.531 | 0.123 | 0.308 | 69.855 | 391 | 348 |
| CGC | 0.227 | 0.141 | -0.050 | 0.504 | 0.108 | 0.300 | 65.320 | 391 | 348 |
| CGH | 0.181 | 0.129 | -0.072 | 0.435 | 0.160 | 0.309 | 58.727 | 391 | 348 |
| CR | 0.234 | 0.122 | -0.006 | 0.474 | 0.056 | 0.300 | 53.998 | 391 | 348 |
| CST | 0.347 | 0.148 | 0.056 | 0.637 | 0.019 | 0.238 | 68.228 | 391 | 348 |
| EC | 0.198 | 0.179 | -0.152 | 0.548 | 0.268 | 0.411 | 78.399 | 391 | 348 |
| FX | 0.153 | 0.146 | -0.134 | 0.440 | 0.296 | 0.411 | 67.826 | 391 | 348 |
| FXST | 0.176 | 0.134 | -0.086 | 0.438 | 0.189 | 0.315 | 61.405 | 391 | 348 |
| GCC | 0.205 | 0.094 | 0.020 | 0.390 | 0.030 | 0.250 | 26.329 | 391 | 348 |
| IC | 0.152 | 0.140 | -0.124 | 0.427 | 0.280 | 0.411 | 65.068 | 391 | 348 |
| IFO | 0.115 | 0.078 | -0.037 | 0.267 | 0.137 | 0.309 | 0.004 | 391 | 348 |
| PCR | 0.162 | 0.116 | -0.066 | 0.390 | 0.163 | 0.309 | 49.487 | 391 | 348 |
| PLIC | 0.131 | 0.140 | -0.144 | 0.406 | 0.350 | 0.432 | 65.026 | 391 | 348 |
| PTR | 0.068 | 0.120 | -0.167 | 0.303 | 0.572 | 0.622 | 52.186 | 391 | 348 |
| RLIC | 0.070 | 0.148 | -0.220 | 0.360 | 0.635 | 0.661 | 68.548 | 391 | 348 |
| SCC | 0.124 | 0.145 | -0.159 | 0.408 | 0.391 | 0.444 | 67.112 | 391 | 348 |
| SCR | 0.277 | 0.114 | 0.053 | 0.501 | 0.015 | 0.238 | 47.520 | 391 | 348 |
| SFO | 0.130 | 0.129 | -0.123 | 0.383 | 0.315 | 0.414 | 58.844 | 391 | 348 |
| SLF | 0.207 | 0.152 | -0.091 | 0.504 | 0.173 | 0.309 | 70.022 | 391 | 348 |
| SS | 0.134 | 0.148 | -0.155 | 0.424 | 0.363 | 0.432 | 68.333 | 391 | 348 |
| UNC | 0.022 | 0.144 | -0.259 | 0.303 | 0.878 | 0.878 | 66.564 | 391 | 348 |
| AverageMD | 0.275 | 0.162 | -0.042 | 0.591 | 0.089 | 0.300 | 0.000 | 158 | 51 |

Table S143. Cohen’s d effect sizes after meta-analysis, for RD differences between patients and controls **in the age range of UK biobank (>42 years of age)**. Age, sex, agexsex, age^2^, age^2^xsex and scansite included as covariates.

| Region | Cohen’s d | SE | CI LB | CI UB | P-value | FDR P-value | I^2^ | Controls | Patients |
| --- | --- | --- | --- | --- | --- | --- | --- | --- | --- |
| ACR | 0.157 | 0.078 | 0.005 | 0.310 | 0.043 | 0.280 | 0.004 | 391 | 348 |
| ALIC | 0.135 | 0.078 | -0.017 | 0.287 | 0.082 | 0.293 | 0.004 | 391 | 348 |
| BCC | 0.174 | 0.100 | -0.021 | 0.370 | 0.081 | 0.293 | 33.071 | 391 | 348 |
| CC | 0.005 | 0.097 | -0.186 | 0.196 | 0.956 | 0.956 | 30.309 | 391 | 348 |
| CGC | 0.160 | 0.078 | 0.008 | 0.312 | 0.039 | 0.280 | 0.000 | 391 | 348 |
| CGH | 0.149 | 0.078 | -0.003 | 0.301 | 0.055 | 0.280 | 0.006 | 391 | 348 |
| CR | 0.122 | 0.078 | -0.030 | 0.274 | 0.117 | 0.325 | 0.002 | 391 | 348 |
| CST | 0.237 | 0.110 | 0.021 | 0.453 | 0.032 | 0.280 | 43.775 | 391 | 348 |
| EC | 0.193 | 0.141 | -0.083 | 0.469 | 0.171 | 0.389 | 65.119 | 391 | 348 |
| FX | 0.131 | 0.136 | -0.135 | 0.397 | 0.333 | 0.595 | 62.614 | 391 | 348 |
| FXST | 0.094 | 0.077 | -0.058 | 0.246 | 0.224 | 0.456 | 0.000 | 391 | 348 |
| GCC | -0.025 | 0.137 | -0.293 | 0.243 | 0.857 | 0.938 | 63.325 | 391 | 348 |
| IC | 0.014 | 0.114 | -0.208 | 0.237 | 0.900 | 0.938 | 47.278 | 391 | 348 |
| IFO | -0.039 | 0.128 | -0.290 | 0.213 | 0.762 | 0.938 | 58.309 | 391 | 348 |
| PCR | -0.024 | 0.120 | -0.260 | 0.212 | 0.842 | 0.938 | 52.737 | 391 | 348 |
| PLIC | -0.075 | 0.143 | -0.356 | 0.206 | 0.601 | 0.865 | 66.592 | 391 | 348 |
| PTR | -0.080 | 0.194 | -0.461 | 0.301 | 0.681 | 0.896 | 81.836 | 391 | 348 |
| RLIC | -0.061 | 0.123 | -0.302 | 0.181 | 0.623 | 0.865 | 54.972 | 391 | 348 |
| SCC | -0.161 | 0.185 | -0.525 | 0.202 | 0.384 | 0.623 | 79.998 | 391 | 348 |
| SCR | 0.148 | 0.077 | -0.004 | 0.300 | 0.056 | 0.280 | 0.000 | 391 | 348 |
| SFO | -0.012 | 0.077 | -0.164 | 0.140 | 0.874 | 0.938 | 0.000 | 391 | 348 |
| SLF | 0.092 | 0.078 | -0.060 | 0.244 | 0.237 | 0.456 | 0.007 | 391 | 348 |
| SS | 0.124 | 0.147 | -0.164 | 0.412 | 0.399 | 0.623 | 68.023 | 391 | 348 |
| UNC | -0.217 | 0.135 | -0.482 | 0.049 | 0.110 | 0.325 | 62.322 | 391 | 348 |
| AverageRD | 0.236 | 0.161 | -0.081 | 0.552 | 0.144 | 0.360 | 0.000 | 158 | 51 |
